# Electrophilic Reagents for the Direct Incorporation of Uncommon SCF_2_CF_2_H and SCF_2_CF_3_ Motifs

**DOI:** 10.1021/acs.joc.2c01038

**Published:** 2022-08-09

**Authors:** Jordi Mestre, Miguel Bernús, Sergio Castillón, Omar Boutureira

**Affiliations:** Departament de Química Analítica i Química Orgànica, Universitat Rovira i Virgili, C/ Marcel·lí Domingo 1, 43007 Tarragona, Spain

## Abstract

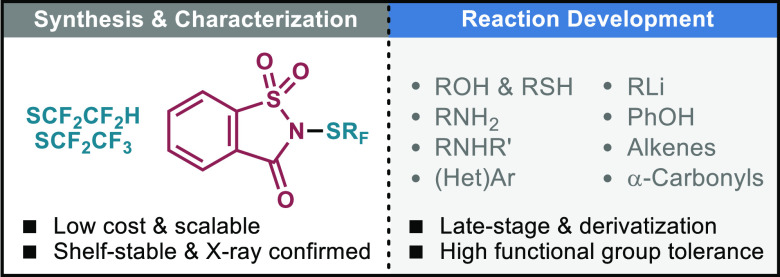

The introduction of fluoroalkylthioether groups has attracted the attention of the drug-discovery community given the special physicochemical and pharmacokinetic features they confer to bioactive compounds, yet these are often limited to standard SCF_3_ and SCF_2_H moieties. Herein, two saccharin-based electrophilic reagents have been disclosed for the incorporation of uncommon SCF_2_CF_2_H and SCF_2_CF_3_ motifs. Their reactivity performance, multigram-scale preparation, and divergent derivatization have been thoroughly investigated with a variety of nucleophiles, including natural products and pharmaceuticals.

## Introduction

**Figure 1:**
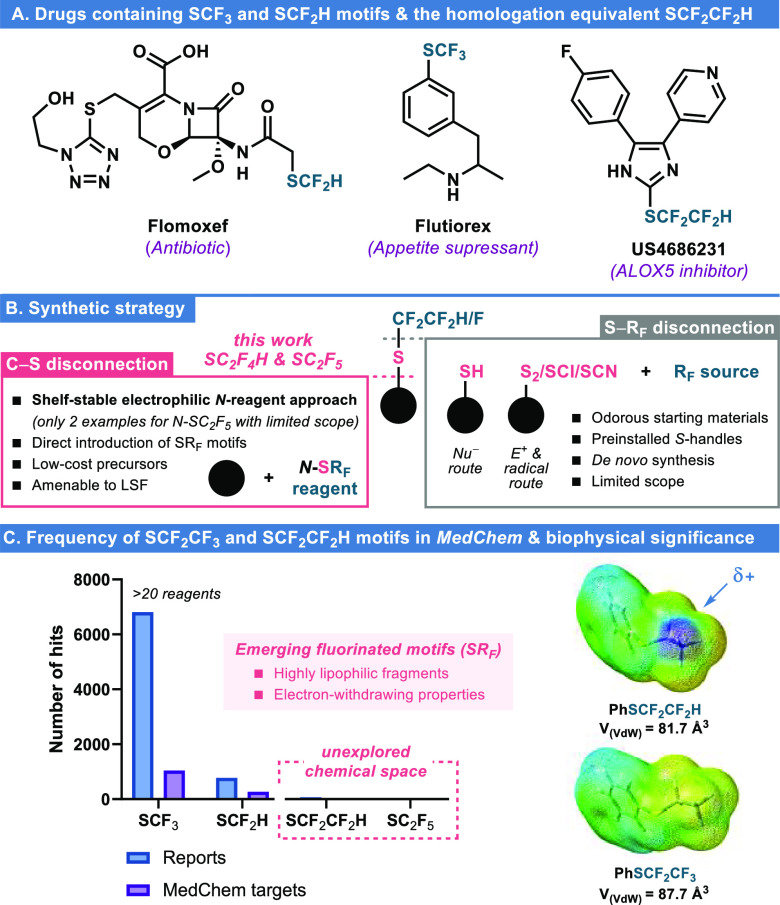
(A) Selected drugs containing fluoroalkylthioether groups. (B) Synthetic strategy and disconnections to R–SR_F_ motifs. (C) Reports on the installation of selected SR_F_ motifs and *MedChem* targets. Biophysical significance of SCF_2_CF_3_ and SCF_2_CF_2_H motifs.^10,11^ Hits obtained with the Reaxys database.

The introduction of fluoroalkyl motifs has been a cornerstone in synthetic, medicinal, and crop chemistry by virtue of the fine-tuning optimization of physicochemical properties of the modified compounds.^1^ Over the last few years, the so-called *fluorinated emerging motifs*(2) have entered this arena to find structural alternatives to the most exploited CF_3_ and F substituents. In this immense scenario, thiofluoroalkyl motifs (SR_F_) occupy a privileged position since the association of fluoroalkyl chains with sulfur results in a powerful combination.^3^ The high electronegativity induced by the fluorine atoms combined with the electronic density of the chalcogen, renders highly lipophilic fragments.^4^ In medicinal chemistry, these attributes are interesting as they lead to more metabolically stable and higher cell-membrane/blood–brain barrier-permeable ingredients, thus increasing the bioavailability of drug candidates.^5^ Fluoroalkyl modified thioethers not only show outstanding Hansch lipophilicity (e.g., CF_3_, 0.88 vs SCF_3_, 1.44)^6^ but also serve as pivotal groups to access other appreciated derivatives, including fluorinated sulfones, sulfonamides, and sulfoximines.^7^ Collectively, these groups exhibit unique properties and represent new avenues for the development of improved bioactive compounds (Figure 1A). Classically, SR_F_ motifs have been prepared by fluoroalkylation of SH, S_2_, SCl, or SCN moieties via S–R_F_ disconnection (Figure 1B, right panel).^8^ However, this strategy is not amenable to late-stage functionalization as it requires a preinstalled sulfur handle in the parent molecule. For this reason, fluoroalkylthiolating reagents (and other direct, *one-pot* protocols) have emerged as a power alternative for the direct modification of target compounds via C–S disconnection (Figure 1B, left panel).^9^

In recent years, most of the vast number of reports describing nucleophilic, electrophilic, radical, or oxidative fluoroalkylthiolating agents/protocols are limited to the introduction of SCF_3_,^12^ followed in number by SCF_2_H.^13,14^ Despite recent advances in the field, drug development comprising other polyfluorinated ethyl congeners is virtually absent (Figure 1C, left panel). Compared to the SCF_3_ motif, SCF_2_CF_2_H and SCF_2_CF_3_ fragments confer a larger van der Waals volume (81.7 and 87.7 Å^3^, respectively vs 58.3 Å^3^ for SCF_3_).^10^ Thus, higher lipophilicity is expected because of the increase in fluorination degree, although subtle differences in polarity may arise due to the uncommon fluorination patterns (Figure 1C, right panel).^15^ Alike the CF_2_H group,^16^ examination of the electrostatic potential surface of PhSCF_2_CF_2_H indicates a terminal electropositive region, suggesting the capability of this group to act as a hydrogen bond donor (Supporting information (SI), Figures S5 and S6). Besides a few *one-pot* nucleophilic/radical methods,^17^ to the best of our knowledge, only two *N*-electrophilic sulfenamide reagents have been disclosed by Billard for the introduction of the SCF_2_CF_3_ motif. However, the electrophilic reactivity shown is limited to the modification of two examples of activated aromatics (phenol and 1,3-dimethoxybenzene), ethynyl lithium, and Grignard nucleophiles.^18^ On the other hand, although mechanistically different to the prototypical *N*-electrophilic reagents, the in situ-generated ^–^SC_2_F_5_ anion from either sulfenamide reagents by Billard^19^ or from benzothiazolium reagents by Hopkinson^20^ enabled the formal incorporation of the SCF_2_CF_3_ motif via nucleophilic substitution of halides, tosylates/mesylates, or alcohols, respectively. Concerning the other potentially valuable SCF_2_CF_2_H fragment, and although recent efforts have been undertaken toward the development of tetrafluoroethylation protocols,^21,22^ direct transfer of tetrafluoroethylthioether units still remains uncharted.^23^

## Results and Discussion

### Reagent Design and Development

**Figure 2:**
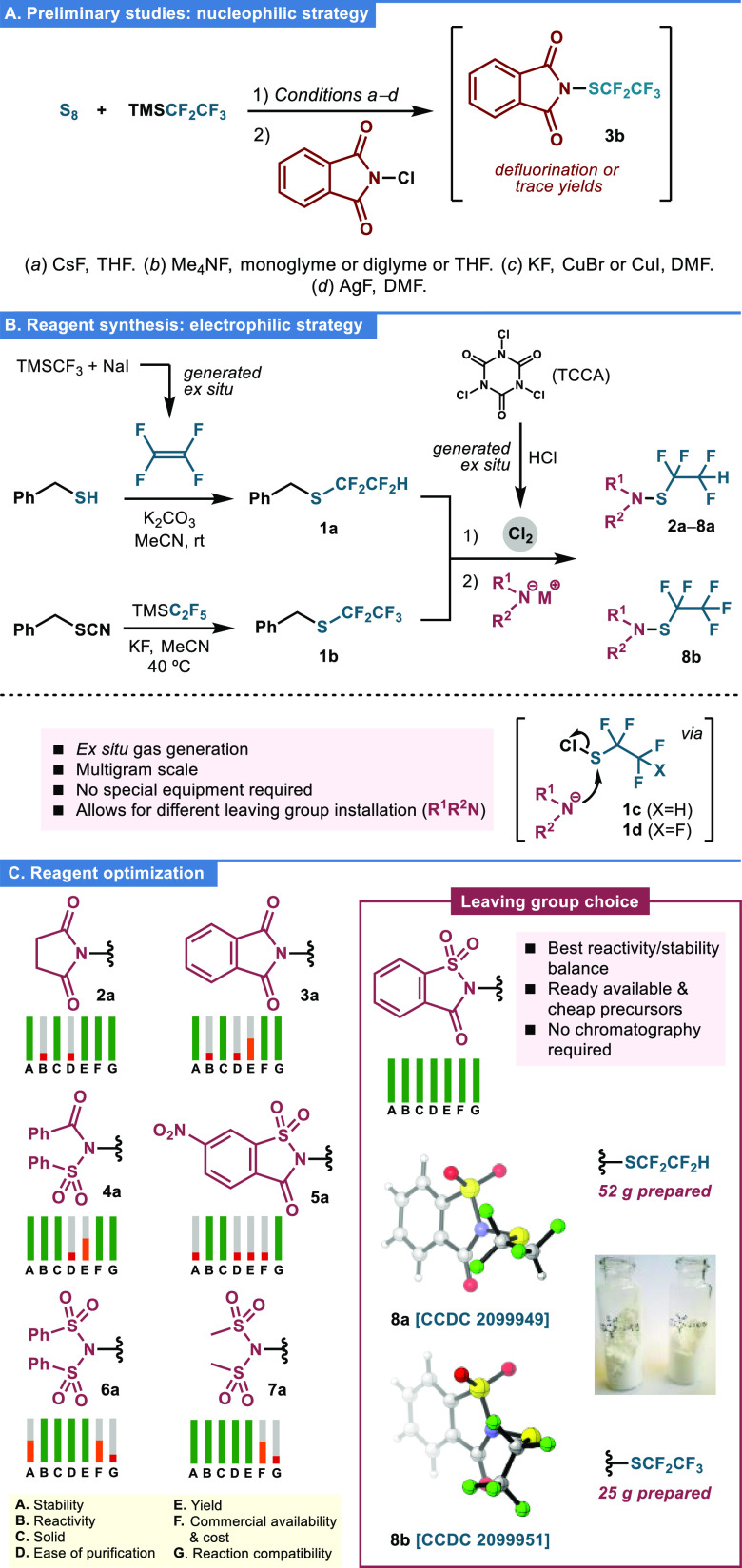
(A) Preliminary attempts for the preparation of SCF_2_CF_3_ reagent **3b** using the standard nucleophilic route. (B) *Umpolung* (electrophilic) route to SCF_2_CF_2_H **2a**–**8a** and SCF_2_CF_3_**8b** reagents. (C) Reagent optimization. See the SI for details. TMS = trimethylsilyl, TCCA = trichloroisocyanuric acid.

Willing to develop electrophilic reagents able to transfer the aforementioned thiofluoroalkyl chains, we turned our attention to imide- and sulfonamide-based scaffolds. Typically, the N–S–R_F_ triad in these reagents is constructed by a general nucleophilic approach from either thiolate salts and *N*-Cl compounds (for SCF_3_)^24^ or AgCF_2_H and *N*-SCl precursors (for SCF_2_H).^13^ However, longer fluoroalkyl thiols show very low stability due to α-fluoride elimination processes.^25^ Thus, all our first attempts using the in situ-generated M^+^^–^SC_2_F_5_, (M^+^ = Ag^+^, Cu^+^, NMe_4_^+^)^26^ were unsuccessful (Figure 2A). In view of these results, we decided to adjust the synthetic strategy using electrophilic ^+^SR_F_ synthons for the preparation of the final electrophilic reagents (Figure 2B).^27^ Thus, chlorination of readily available 1,1,2,2-tetrafluoroethyl **1a** and pentafluoroethyl **1b** benzyl thioethers gave access to key sulfenyl chlorides **1c,d**,^28^ which reacted with various imide, and sulfonamide salts to render a family of *N*-reagents **2a**–**8a**, **8b**, featuring succinimide, phthalimide, saccharine, and sulfonamides as representative leaving groups. Importantly, this synthetic protocol uses cheap and widely available starting materials, making it suitable for scaling-up reactions (up to 52 g of **8a** prepared). The choice of the optimal reagent was based on a balance between synthetic yield, reactivity, stability, and cost (SI, Table S1). Saccharine-SCF_2_CF_2_H **8a** and SCF_2_CF_3_**8b** exhibited the best overall results (Figure 2C). Both reagents showed robust stability not only in the solid state but also in solution as demonstrated by differential scanning calorimetry (DSC) and thermogravimetric (TGA) analyses as well as solvent stability studies (SI, Figures S1–S3).

### Reaction Scope

**Scheme 1:**
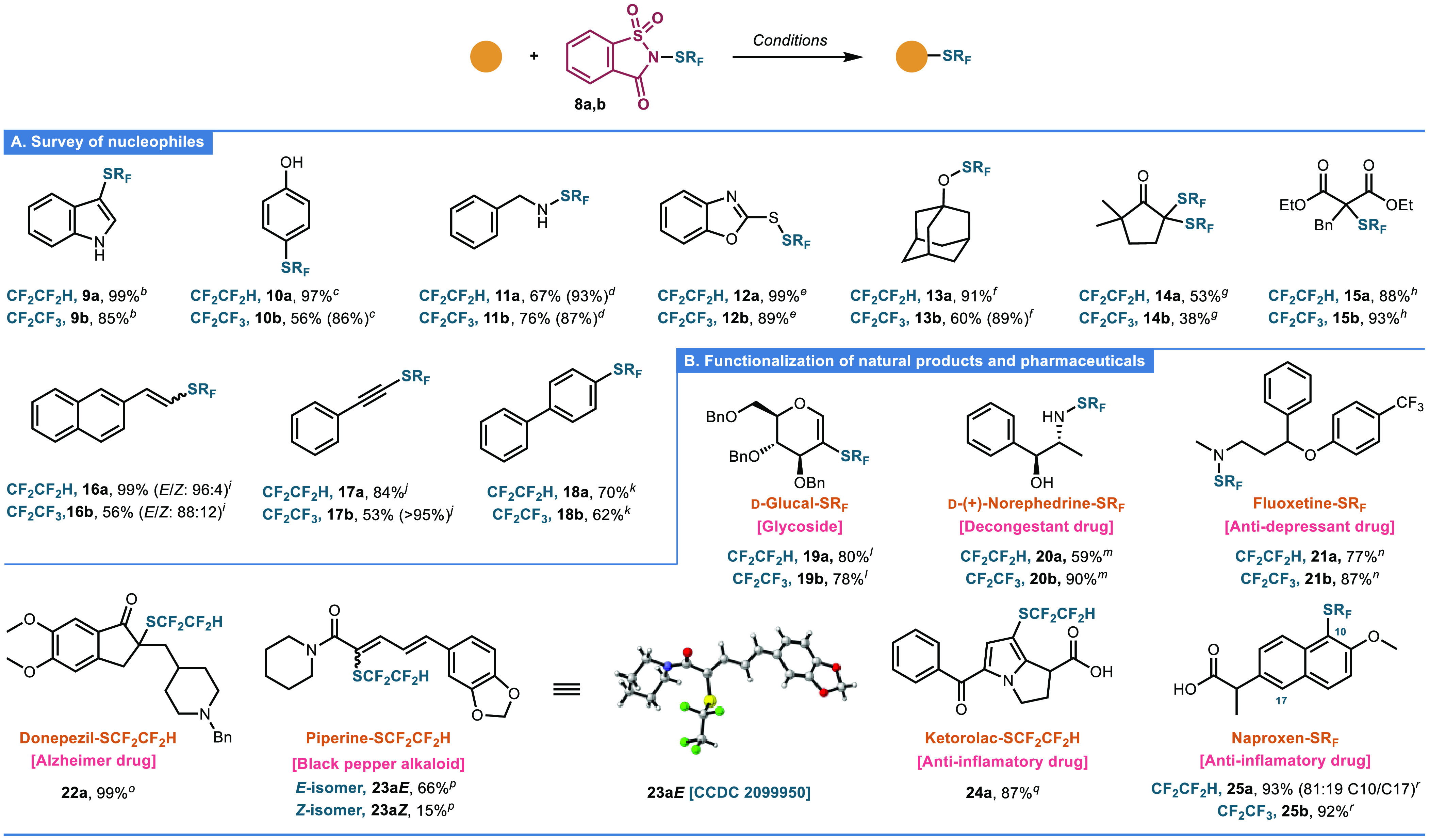
(A) Scope of Nucleophiles and (B) Functionalization of Natural Products and Pharmaceuticals Conditions: *^b^*1*H*-Indole (1.0 equiv), **8a**,**b** (1.1 equiv), CH_2_Cl_2_, 40 °C. ^*c*^PhOH (1.0 equiv), **8a,b** (1.2 equiv), TfOH (1.0 equiv), CH_2_Cl_2_, rt. ^*d*^BnNH_2_ (1.0 equiv), **8a,b** (1.1 equiv), CH_2_Cl_2_, rt. ^*e*^2-Mercaptobenzoxazole (1.0 equiv), **8a**,**b** (1.1 equiv), CH_2_Cl_2_, rt. ^*f*^Adamantol (1.0 equiv), **8a**,**b** (1.3 equiv), Et_3_N (2.5 equiv), CH_2_Cl_2_, rt. ^*g*^(i) 2,2-Dimethylcyclopentan-1-one (1.0 equiv), KHMDS (1.2); (ii) **8a**,**b** (2.5 equiv), THF, −78 °C. ^*h*^(i) Diethyl 2-benzylmalonate (1.0 equiv), NaH (3 equiv); (ii) **8a**,**b** (1.7 equiv), THF, rt. ^*i*^(i) 2-Vinylnaphthalene (1.0 equiv), TMSCl (3 equiv), **8a**,**b** (2.2 equiv); (ii) DBU (6 equiv), MeCN, rt. ^*j*^(*i*) Phenylacetlylene (1.0 equiv), *n*-BuLi (1.1 equiv); (ii) **8a**,**b** (1.2 equiv), THF, −78 °C. ^*k*^(*i*) 4-Bromo-1,1′-biphenyl (1.0 equiv), *n*-BuLi (1.1 equiv); (ii) **8a**,**b** (1.2 equiv), THF, −78 °C. ^*l*^(*i*) Tri-*O*-benzyl-d-glucal (1.0 equiv), 3 Å MS, TMSCl (3 equiv), **8a,b** (2.2 equiv); (ii) DBU (6 equiv), MeCN, rt. ^*m*^(1*S*,2*R*)-(+)-Norephedrine (1.0 equiv), **8a**,**b** (3 equiv), CH_2_Cl_2_, rt. ^*n*^Fluoxetine (1.0 equiv), **8a**,**b** (1.5 equiv), Et_3_N (1.1 equiv), CH_2_Cl_2_, rt. ^*o*^(i) Donepezil (1.0 equiv), KHMDS (1.3 equiv); (ii) **8a** (1.3 equiv), THF, −78 °C. ^*p*^Piperine (1.0 equiv), **8a** (2.2 equiv), TMSCl (1.2 equiv), CH_2_Cl_2_, rt. ^*q*^Ketorolac (1.0 equiv), **8a** (2.0 equiv), TMSCl (2 equiv), CH_2_Cl_2_, rt. *^r^rac*-Naproxen (1.0 equiv), **8a**,**b** (1.5 equiv), TfOH (1.2 equiv), CHCl_3_, 40 °C (for **25a**) or 70 °C (for **25b**). Isolated yields given. Yields in parenthesis were determined by ^19^F NMR using 1,4-difluorobenzene (DFB) as internal standard (see the SI for details). MS = molecular sieves, TfOH = trifluoromethanesulfonic acid, HMDS = hexamethyldisilazane, THF = tetrahydrofuran, TMS = trimethylsilyl, DBU = 1,8-diazabicyclo(5.4.0)undec-7-ene.

With the optimal reagents in hand, their applicability was first evaluated with representative nucleophiles (Scheme 1A). First, a preliminary solvent compatibility study of **8a** with *N*-H indole demonstrated that solvents of different nature (chlorinated, aprotic polar, and aprotic nonpolar solvents) do not substantially affect the performance of the reaction with yields of **9a** up to >95% (SI, Figure S4). Thus, reaction of *N*-H indole in CH_2_Cl_2_ with **8a**,**b** afforded **9a** (99%) and **9b** (85%) after heating at 40 °C for 1 or 24 h, respectively. Reaction with phenol required the addition of TfOH as a promoter and afforded **10a** (97%) and **10b** (86%). Next, we assayed the suitability of other nucleophiles to afford N–, O–, and S–SR_F_ bonds.^29^ Thus, reaction with benzylamine gave the desired products **11a** (93%) and **11b** (87%) after 1 h at room temperature, while reaction with 2-mercaptobenzoxazole afforded instantaneously disulfides **12a** (99%) and **12b** (89%). Unlike phenol, which required a protic acid that activates the electrophilic reagent, preliminary results with alcoholic nucleophiles indicate the necessity of an exogenous base (e.g., Et_3_N) to deprotonate the hydroxyl moiety and deliver the desired products. Thus, adamantol derivatives **13a** (91%) and **13b** (89%) were obtained after 1 h at room temperature, using Et_3_N as a base. Reactions with the preformed enolate of 2,2-dimethylcyclopentanone afforded the double substitution products **14a** (53%) and **14b** (38%). Attempts to selectively obtain the monosubstituted product were unsuccessful due to the increased reactivity of the monosubstituted intermediate. Treatment of diethyl benzylmalonate with sodium hydride (NaH) and subsequent reaction with **8a**,**b** afforded **15a** and **15b** in 88 and 93% yield, respectively. Alkenes are also suitable nucleophiles as demonstrated with 2-vinylnaphthalene, using an addition/elimination sequence that afforded *E*/*Z* mixtures (up to 96:4) of vinylic SCF_2_CF_2_H **16a** (99%) and SCF_2_CF_3_**16b** (56%). While treatment of phenylacetylene with **8a** in the presence of CuBr failed to deliver the desired product,^18^ reaction of the alkyne with *n*-BuLi and subsequent reaction with **8a**,**b** rendered **17a** (84%) and **17b** (>95%). Similarly, generation of the organolithium intermediate from 4-bromobiphenyl by lithium–bromine exchange afforded **18a** (70%) and **18b** (62%) after subsequent reaction with **8a,b**.^30^

Next, having demonstrated the versatility of our reagents with model nucleophiles, we aimed to evaluate their efficiency for the direct/late-stage modification of natural products and pharmaceuticals (Scheme 1B).^31^ First, the aforementioned addition/elimination protocol also worked well for the benzyl-protected d-glucal to afford **19a** (80%) and **19b** (78%).^32^ Interestingly, despite the large volume of SCF_2_CF_3_ and SCF_2_CF_2_H groups, they have less impact on the conformation of 2-substituted-d-glucals than their alkyl (e.g., CF_2_CF_3_, CF_3_) counterparts as indicated by the analysis of diagnostic coupling constants ^3^*J*_3,4_ = 4–4.6 Hz and ^3^*J*_4,5_ = 5–5.8 Hz (intermediate conformation deformed toward the ^5^H_4_) (SI, Figure S7).^33,34^ (+)-Norephedrine was chemoselectively *N*-modified to **20a** (59%) and **20b** (90%) under mild reaction conditions without competitive *O*-substitution. The secondary amine of fluoxetine (Prozac) also reacted successfully to deliver **21a,b** in 77% and 87% yield, respectively. Donepezil, a drug used in the treatment of Alzheimer’s disease, was reacted with potassium bis(trimethylsilyl)amide (KHMDS) to generate the enolate that subsequently reacted with **8a** to afford **22a** in an excellent 99% yield. Similarly to 2-vinylnaphthalene and d-glucal, the use of the same addition/elimination protocol with piperine (black pepper alkaloid) and **8a** in the presence of trimethylsilyl chloride (TMSCl) as a promoter, afforded **23a** as a separable mixture of *E*/*Z*-isomers **23a*E*** (66%) and **23a*Z*** (15%), resulting from the modification of the conjugated diene system as determined by NMR and X-ray (for the *E*-isomer) analysis. Reaction of **8a** with ketorolac, an anti-inflammatory agent, afforded **24a** (87%) with the exclusive modification of the pyrrole moiety, thus demonstrating the compatibility of our reagent **8a** with carboxylic acids. Finally, when naproxen was reacted with **8a** and TfOH as a promoter, **25a** (93%) was obtained as an 81:19 mixture of C10/C17 regioisomers. In contrast, reaction with **8b** afforded **25b** (92%) as the sole C10-isomer.

### Large Scale and Derivatization

**Figure 3:**
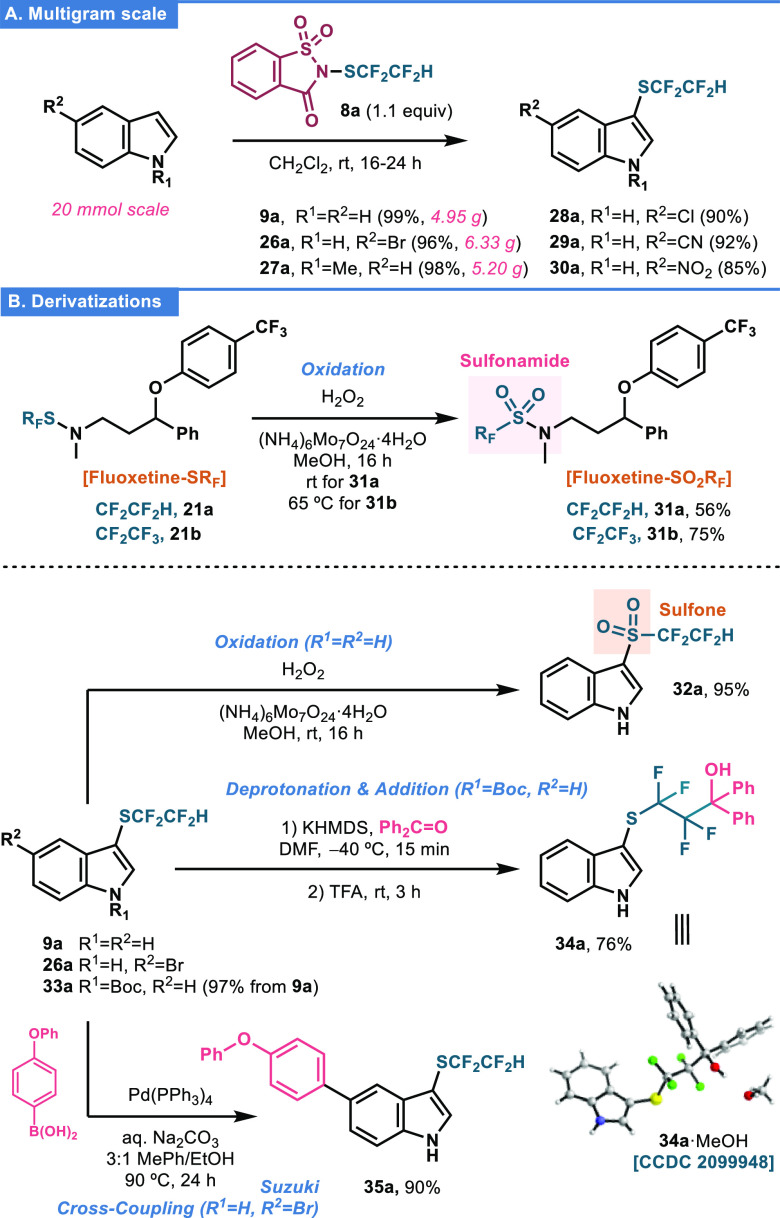
(A) Multigram-scale preparation of tetrafluoroethylthio indoles and (B) derivatization reactions. See the SI for details. Boc, *tert*-butoxycarbonyl; DMF, *N*,*N*-dimethylformamide; HMDS, hexamethyldisilazane; TFA, trifluoroacetic acid.

Next, multigram-scale reactions (20 mmol) with a series of unprotected and *N*-Me-protected indoles afforded gram amounts of the corresponding SCF_2_CF_2_H-analogues **9a**, **26a**, and **27a** with yields up to 99% (Figure 3A). Notably, reaction crudes are substantially clean and only excess of **8a** and saccharine are observed, which indeed can be simply removed by sequential washings with aqueous Na_2_CO_3_. In addition, reactions with 5-substituted indoles bearing Cl, CN, and NO_2_ moieties afforded analogues **28a**–**30a** (up to 92%) that further demonstrate the functional group compatibility/tolerance of our reagents.

Because the 1,1,2,2-tetrafluoroethylthio moiety represents an interesting platform for accessing other compounds, various derivatization reactions were evaluated (Figure 3B). First, sulfenamide fluoxetine derivatives **21a,b** were oxidized to sulfonamides **31a** (56%) and **31b** (75%) using H_2_O_2_ and a molybdenum catalyst. This methodology represents an overall workable strategy to obtain uncommon, fluorinated sulfonamides (Figure 3B, upper panel). Noteworthily, the same oxidation conditions could be applied to the oxidation of thioether **9a** to the corresponding sulfone **32a** (95%) (Figure 3B, lower panel). Next, after *N*-Boc protection of indole **9a** to **33a** (Boc_2_O, Et_3_N, CH_2_Cl_2_, rt, 16 h, 97%), the SCF_2_CF_2_H moiety of product **33a** was deprotonated with KHMDS and the resulting carbanion quenched with benzophenone. Finally, *N*-Boc removal with TFA gave access to CF_2_CF_2_-bridged **34a** in 76% yield. This strategy serves as a proof of concept for the functionalization with electrophiles of terminal SCF_2_CF_2_H-containing compounds.^22^ Finally, Suzuki cross-coupling of 5-bromoindole **26a** with an aryl boronic acid partner smoothly afforded **35a** in an excellent 90% yield, thus demonstrating group compatibility with Pd-catalyzed transformations.

## Conclusions

In summary, two new reagents for the direct introduction of uncommon SCF_2_CF_2_H and SCF_2_CF_3_ motifs have been disclosed. These electrophilic agents are synthetized in three steps from simple and readily available starting materials and can be obtained in a multigram scale. Electrophilic introduction has proven successful in a range of different nucleophiles, including amines, alcohols, thiols, electron-rich (hetero)aromatics, phenols, ketones, 1,3-diesters, and alkenes as well as organolithium alkyne and arene derivatives. The robustness of the transformation, including its operational/purification simplicity has been further demonstrated with a range of complex structures, including blockbuster drugs and natural products. Multigram-scale reactions and product derivatization to sulfones, sulfonamides, and deprotonation of SCF_2_CF_2_H-addition to electrophiles as well as orthogonal metal-mediated reactions have also been demonstrated. We expect our findings will provide new opportunities in drug and agrochemical discovery by expanding the toolbox of reagents for the introduction of new fluorinated motifs into natural products and active principal ingredients.

## Experimental Section

### General Remarks

Proton (^1^H NMR), carbon (^13^C{^1^H}) NMR), and fluorine (^19^F NMR) nuclear magnetic resonance spectra were recorded on a Varian Mercury spectrometer or a Bruker Avance Ultrashield (400 MHz for ^1^H), (100.6 MHz for ^13^C{^1^H}), and (376.5 MHz for ^19^F). Spectra were fully assigned using COSY, HSQC, HMBC, and NOESY. All chemical shifts are quoted on the δ scale in parts per million (ppm) using the residual solvent as internal standard (^1^H NMR: CDCl_3_ = 7.26, CD_2_Cl_2_ = 5.32, CD_3_OD = 3.31 and (^13^C{^1^H} NMR: CDCl_3_ = 77.16, CD_2_Cl_2_ = 54.0, CD_3_OD = 49.0). Coupling constants (*J*) are reported in Hz with the following splitting abbreviations: s = singlet, d = doublet, t = triplet, q = quartet, and app = apparent. Infrared (IR) spectra were recorded on a FTIR–ATR spectrophotometer. Absorption maxima (ν_max_) are reported in wavenumbers (cm^–1^). High-resolution mass spectra (HRMS) were recorded on an LC–MS system (UHPLC 1290 Infinity II Series coupled to a qTOF/MS 6550 Series, both Agilent Technologies (Agilent Technologies). For the ionization, an ESI operating on positive or negative ionization or an APCI operating on positive or negative ionization was used. Water and methanol with 0.05% formic acid were used as mobile phases. The quadrupole time of flight mass spectrometer (qTOF) operated in high-resolution MS scan mode between 100–1000 *m*/*z*. For GC–HRMS mass determination the compounds were directly analyzed by gas chromatography coupled to high-resolution mass spectrometry (7200 GC–qTOF from Agilent Technologies). For ionization, electron impact ionization was used. The chromatographic column was a 5HP–MS from Agilent and carried gas was He. The quadrupole time of flight mass spectrometer (qTOF) operated in high-resolution MS scan mode between 100–600 *m*/*z*. Nominal and exact *m*/*z* values are reported in Daltons. Thin layer chromatography (TLC) was carried out using commercial backed sheets coated with 60 Å F_254_ silica gel. Visualization of the silica plates was achieved using a UV lamp (λ_max_ = 254 nm), 6% H_2_SO_4_ in EtOH, cerium molybdate, and/or potassium permanganate staining solutions. Flash column chromatography was carried out using silica gel 60 Å CC (230–400 mesh). Mobile phases are reported in relative composition (e.g., 1:1 EtOAc/hexane v/v). All reactions using anhydrous conditions were performed using oven-dried apparatus under an atmosphere of argon. Brine refers to a saturated solution of sodium chloride. Anhydrous sodium sulfate (Na_2_SO_4_) was used as drying agent after reaction work-up, as indicated. All reagents were purchased from Sigma Aldrich, Cymit, Carbosynth, Apollo Scientific, Fluorochem and Manchester Organics chemical companies. General crystallization procedure: a sample of the product was charged in an HRMS vial and was dissolved using a minimal amount of THF. The HRMS vial was fitted inside a bigger vial containing pentane and the latter was capped and left unperturbed overnight. Slow vapor diffusion caused growing of crystals that showed good quality for single-crystal X-ray diffraction analysis. X-ray figures in the article were rendered with CyLview software.

#### Benzyl(1,1,2,2-tetrafluoroethyl)sulfane (**1a**)

A 250 mL round-bottom flask (reaction flask A), equipped with a magnetic stir bar was charged with potassium hydroxide (90%, 1.68 g, 30 mmol). The flask was then evacuated and backfilled with argon three times. Subsequently, anhydrous and deoxygenated MeCN (100 mL) was added followed by benzyl mercaptan (11.7 mL, 100 mmol) and an argon balloon was attached through the rubber septa using a needle. Then, a second flask (reaction flask B) containing NaI (2.25 g, 15 mmol) in anhydrous and deoxygenated THF (100 mL) was attached to a reflux condenser connected to a Teflon (PTFE) tube and the outlet was immersed in the solution of reaction flask A. Then, reaction flask B was heated to 70 °C with an aluminum heating block and TMSCF_3_ was added (4 × 11 mL, 74.4 mmol, every 30 min) while bubbling was observed in reaction flask A. If overpressure was observed by dilation of the balloon connected to flask A, this was detached, emptied, and connected again to liberate excess of pressure. When bubbling stopped after additions of TMSCF_3_, the reaction mixture was stirred at room temperature for further 3 h. Then, the reaction mixture was concentrated in a rotary evaporator without heating, the crude redissolved in Et_2_O and washed with 10% aqueous KOH and brine. The organic phase was dried with Na_2_SO_4_, filtered, and concentrated under gentle vacuum in a rotary evaporator without heating. The product was distilled under reduced pressure to afford **1a** (15.1 g, 67%) as a colorless liquid. *R*_f_ (hexane): 0.26; ^1^H NMR (CDCl_3_, 400 MHz): δ 7.48–7.31 (m, 5H), 5.80 (tt, *J* = 53.9, 3.3 Hz, 1H), 4.19 (s, 2H); ^13^C{^1^H} NMR (CDCl_3_, 100.6 MHz): δ 135.5, 129.2, 129.0, 128.1, 123.8 (tt, *J* = 283.4, 30.1 Hz), 109.9 (tt, *J* = 252.8, 38.2 Hz), 32.4 (t, *J* = 4.0 Hz); ^19^F NMR (CDCl_3_, 376.5 MHz): δ −92.03 (td, *J* = 9.0, 3.2 Hz, 2F), −132.00 (dt, *J* = 54.0, 9.0 Hz, 2F); FTIR–ATR (neat) ν in cm^–1^: 1496, 1455, 1383, 1212, 1105, 990, 808, 768, 697, 663, 636, 551; HRMS (APCI^–^) for (M–H)^−^ C_9_H_7_F_4_S^–^ (*m/z*): calcd 223.0210; found 223.0201.

#### Benzyl(perfluoroethyl)sulfane (**1b**)

A flask containing dry KF (8.3 g, 142.4 mmol) and benzylthiocyanate (32.0 g, 213.6 mmol) was evacuated and backfilled with argon three times followed by sequential addition of anhydrous MeCN (110 mL). Then, the mixture was cooled down to 0 °C and TMSCF_2_CF_3_ (25 mL, 142.4 mmol) was added with a syringe. The mixture was stirred under argon at 40 °C with an aluminum heating block for 48 h. Next, the reaction mixture was cooled down to room temperature and diluted with Et_2_O. The organic phase was washed with brine and dried over Na_2_SO_4_. After filtration, the solvent was removed under reduced pressure and the residue was purified by distillation under reduced pressure to afford **1b** (27.59 g, 80%) as a colorless liquid. *R*_f_ (hexane): 0.44; ^1^H NMR (CDCl_3_, 400 MHz): δ 7.37–7.28 (m, 5H), 4.16 (s, 2H); ^13^C{^1^H} NMR (CDCl_3_, 100.6 MHz): δ 134.81, 129.3, 1291, 128.3, 119.0 (qt, *J* = 284.2, 35.8 Hz), 121.7 (tt, *J* = 288.1, 40.3 Hz), 33.1 (t, *J* = 3.9 Hz); ^19^F NMR (CDCl_3_, 376.5 MHz): δ −83.4 (t, *J* = 3.7 Hz, 3F), −92.4 (q, *J* = 3.7 Hz, 2F); FTIR–ATR (neat) ν in cm^–1^: 1496, 1455, 1099, 1030, 774, 766, 755, 695, 480, 465. After extensive analyses with different spectrometric techniques the molecular peak could not be found; only fragmentation can be described by HRMS (TOF EI) for (Bn)^+^ C_7_H_7_^+^ (*m/z*): calcd 91.0542; found 91.0543.

#### 1,1,2,2-Tetrafluoroethyl hypochlorothioite (**1c**)

To a solution of benzyl thioether **1a** (43.07 g, 192 mmol) in CHCl_3_ (100 mL) was bubbled an excess of chlorine gas (27.2 g, 384 mmol) at 0 °C. The reaction mixture was stirred at room temperature and the conversion monitored by ^19^F NMR. After completion of the reaction, the mixture was distilled to collect the desired 1,1,2,2-tetrafluoroethyl hypochlorothioite **1c** as a yellowish solution in CHCl_3_ (115 mL, 1.59 M, 96%). To determine the concentration of **1c** in CHCl_3_, 0.5 mL of the distilled fraction was transferred to an NMR tube followed by addition of 1,4-difluorobenzene (DFB, 20 μL, internal standard) and the concentration was analyzed by quantitative ^19^F NMR. ^19^F NMR (CHCl_3_, 376.5 MHz): δ −97.40 (m, 2F), −133.90 (dt, *J* = 53.6, 8.3 Hz, 2F). DFB referenced to −119.70 ppm.

#### Perfluoroethyl hypochlorothioite (**1d**)

To a solution of benzyl thioether **1b** (26.85 g, 110.9 mmol) in CH_2_Cl_2_ (110 mL) was bubbled an excess of chlorine gas (4.7 g, 332.6 mmol) at 0 °C. The reaction mixture was stirred at room temperature and the conversion monitored by ^19^F NMR. After completion of the reaction, the mixture was distilled to collect the desired perfluoroethyl hypochlorothioite **1d** as a solution yellowish in CH_2_Cl_2_ (71 mL, 1.13 M, 72%). To determine the concentration of **1d** in CH_2_Cl_2_, 0.5 mL of the distilled fraction was transferred to an NMR tube followed by addition of 1,3-bis(trifluoromethyl)benzene (BTB, 20 μL, internal standard) and the concentration was analyzed by quantitative ^19^F NMR. ^19^F NMR (CH_2_Cl_2_, 376.5 MHz): δ −81.46 (t, *J* = 2.6 Hz, 3F), −97.50 (m, 2F). BTB referenced to −62.90 ppm.

#### 1-((1,1,2,2-Tetrafluoroethyl)thio)pyrrolidine-2,5-dione (**2a**)

A round-bottom flask, equipped with a magnetic stir bar, was charged with potassium succinimide salt (206 mg, 1.5 mmol). Subsequently, CHCl_3_ (5 mL) was added using a syringe. Then, the mixture was cooled down to 0 °C and a 1.59 M solution of 1,1,2,2-tetrafluoroethyl hypochlorothioite **1c** in CHCl_3_ was added (0.63 mL, 1 mmol). The mixture was stirred at room temperature for 1 h. The reaction mixture was filtered through Celite, concentrated under reduced pressure, and purified by flash column chromatography (1:1 EtOAc/hexane) to afford **2a** (203 mg, 88%) as a white solid. *R*_f_ (2:3 EtOAc/hexane): 0.28; m.p.: 61–63 °C; ^1^H NMR (CDCl_3_, 400 MHz): δ 5.96 (tt, *J* = 53.2, 3.8 Hz, 1H), 2.92 (s, 4H); ^13^C{^1^H} NMR (CDCl_3_, 100.6 MHz): δ 175.1, 120.8 (tt, *J* = 291.9, 30.0 Hz), 109.5 (tt, *J* = 253.8, 35.7 Hz), 28.6; ^19^F NMR (CDCl_3_, 376.5 MHz): δ −98.1 (td, *J* = 8.9, 3.8 Hz, 2F), −132.8 (dt, *J* = 53.2, 8.9 Hz, 2F); FTIR–ATR (neat) ν in cm^–1^: 1717, 1299, 1217, 1115, 1001, 812, 656, 621, 547, 462, 438; HRMS (TOF ES^+^) for (M + H)^+^ C_6_H_6_F_4_NO_2_S^+^ (*m*/*z*): calcd 232.0050; found 232.0057.

#### 2-((1,1,2,2-Tetrafluoroethyl)thio)isoindoline-1,3-dione (**3a**)

A round-bottom flask, equipped with a magnetic stir bar, was charged with potassium phthalimide salt (278 mg, 1.5 mmol). Subsequently, CHCl_3_ (1.6 mL) was added using a syringe. Then, the mixture was cooled down to 0 °C and a 1.59 M solution of 1,1,2,2-tetrafluoroethyl hypochlorothioite **1c** in CHCl_3_ was added (0.63 mL, 1 mmol). The mixture was stirred at room temperature for 1 h. The reaction mixture was filtered through Celite, concentrated under reduced pressure, and purified by flash column chromatography (1:9 EtOAc/hexane) to afford **3a** (170 mg, 60%) as a white solid. *R*_f_ (1:9 EtOAc/hexane): 0.14; m.p.: 78–80 °C; ^1^H NMR (CDCl_3_, 400 MHz): δ 8.01–7.93 (m, 2H), 7.89–7.81 (m, 2H), 5.98 (tt, *J* = 53.2, 3.8 Hz, 1H); ^13^C{^1^H} NMR (CDCl_3_, 100.6 MHz): δ 166.4, 135.4, 131.6, 124.7, 120.9 (tt, *J* = 291.5, 30.2 Hz), 109.43 (tt, *J* = 253.8, 35.8 Hz); ^19^F NMR (CDCl_3_, 376.5 MHz): δ −98.96 (td, *J* = 8.8, 3.8 Hz, 2F), −132.98 (dt, *J* = 53.2, 8.8 Hz, 2F); FTIR–ATR (neat) ν in cm^–1^: 1747, 1719, 1281, 1100, 1038, 868, 714, 689, 626, 526, 402; HRMS (TOF ES^+^) for (M + H)^+^ C_10_H_6_F_4_NO_2_S^+^ (*m*/*z*): calcd 280.0050; found 280.0056.

#### *N*-(Phenylsulfonyl)-*N*-((1,1,2,2-tetrafluoroethyl)thio)benzamide (**4a**)

A round-bottom flask, equipped with a magnetic stir bar, was charged with potassium *N*-(phenylsulfonyl)benzamide salt (790 mg, 2.64 mmol). Subsequently, CHCl_3_ (5 mL) was added using a syringe. Then, the mixture was cooled down to 0 °C and a 1.59 M solution of 1,1,2,2-tetrafluoroethyl hypochlorothioite **1c** in CHCl_3_ (1.25 mL, 2 mmol) was added. The mixture was stirred at room temperature for 1 h. The reaction mixture was filtered through a sintered funnel and concentrated under reduced pressure to afford **4a** (605 mg, 77%) as a white solid. *R*_f_ (1:4 EtOAc/hexane): decomposes; m.p.: 47–49 °C; ^1^H NMR (CDCl_3_, 400 MHz): δ 8.13–8.08 (m, 2H), 7.70–7.60 (m, 3H), 7.60–7.52 (m, 3H), 7.46–7.40 (m, 2H), 5.89 (tt, *J* = 53.1, 3.6 Hz, 1H); ^13^C{^1^H} NMR (CDCl_3_, 100.6 MHz): δ 171.7, 137.0, 134.8, 133.3, 131.9, 129.5, 129.4, 129.1, 128.6, 121.2 (tt, *J* = 293.7, 30.4 Hz), 109.0 (tt, *J* = 253.6, 36.0 Hz); ^19^F NMR (CDCl_3_, 376.5 MHz): δ −94.81 (d, *J* = 234.3 Hz, 1F), −98.94 (d, *J* = 234.3 Hz, 1F), −133.65 (m, 2F); FTIR–ATR (neat) ν in cm^–1^: 1716, 1360, 1170, 1101, 1053, 1024, 562, 545; HRMS (TOF ES^+^) for (M + H)^+^ C_15_H_12_F_4_NO_3_S_2_^+^ (*m*/*z*): calcd 394.0189; found 394.0192.

#### 6-Nitro-2-((1,1,2,2-tetrafluoroethyl)thio)benzo[*d*]isothiazol-3(2*H*)-one 1,1-Dioxide (**5a**)

A round-bottom flask, equipped with a magnetic stir bar, was charged with potassium 6-nitrobenzo[*d*]isothiazol-3(2*H*)-one-1,1-dioxide salt (703 mg, 2.64 mmol). Subsequently, CHCl_3_ (5 mL) was added using a syringe. Then, the mixture was cooled down to 0 °C and a 1.59 M solution of 1,1,2,2-tetrafluoroethyl hypochlorothioite **1c** in CHCl_3_ was added (1.25 mL, 2 mmol). The mixture was stirred at room temperature for 1 h. The reaction mixture was filtered through a sintered funnel concentrated under reduced pressure to afford **5a** (186 mg, 26%) as a yellowish solid. *R*_f_ (1:4 EtOAc/hexane): decomposes; m.p.: 70–72 °C (decomposes); ^1^H NMR (CDCl_3_, 400 MHz): δ 8.87 (d, *J* = 1.9 Hz, 1H), 8.76 (dd, *J* = 8.4, 1.9 Hz, 1H), 8.41 (d, *J* = 8.4 Hz, 1H), 6.10 (tt, *J* = 53.0, 3.8 Hz, 1H); ^13^C{^1^H} NMR (CDCl_3_, 100.6 MHz): δ 157.2, 152.4, 139.2, 130.6, 130.0, 128.3, 120.5 (m), 118.2, 109.2 (tt, *J* = 254.1, 35.1 Hz); ^19^F NMR (CDCl_3_, 376.5 MHz): δ −96.60 (m, 2F), −133.18 (d, *J* = 53.0 Hz, 2F); HRMS could not be obtained due to instability of the final product.

#### *N*-(Phenylsulfonyl)-*N*-((1,1,2,2-tetrafluoroethyl)thio)benzenesulfonamide (**6a**)

A round-bottom flask, equipped with a magnetic stir bar, was charged with silver *N*-(phenylsulfonyl)benzenesulfonamide salt (404 mg, 1.5 mmol). Subsequently, CHCl_3_ (1.6 mL) was added using a syringe. Then, the mixture was cooled down to 0 °C and a 1.59 M solution of 1,1,2,2-tetrafluoroethyl hypochlorothioite **1c** in CHCl_3_ was added (0.63 mL, 1 mmol). The mixture was stirred at room temperature for 1 h. The reaction mixture was filtered through a sintered funnel and concentrated under reduced pressure to afford **6a** (386 mg, 90%) as a white solid. *R*_f_ (1:4 EtOAc/hexane): decomposes; m.p.: 123–125 °C; ^1^H NMR (CDCl_3_, 400 MHz): δ 8.02–7.96 (m, 4H), 7.70–7.62 (m, 2H), 7.57–7.48 (m, 4H), 6.18 (tt, *J* = 53.0, 4.7 Hz, 1H); ^13^C{^1^H} NMR (CDCl_3_, 100.6 MHz): δ 137.5, 135.0, 129.2, 129.0, 120.0 (tt, *J* = 296.4, 29.0 Hz), 109.0 (tt, *J* = 253.1, 34.2 Hz); ^19^F NMR (CDCl_3_, 376.5 MHz): δ −99.29 (td, *J* = 10.1, 4.7 Hz, 2F), −135.08 (dt, *J* = 53.0, 10.1 Hz, 2F); FTIR–ATR (neat) ν in cm^–1^: 1362, 1151, 1082, 863, 755, 723, 684, 575, 539, 466; HRMS (APCI^+^) for (M)^+·^ C_14_H_11_F_4_NO_4_S_3_^+·^ (*m*/*z*): calcd 428.9786; found 428.9786.

#### *N*-(Methylsulfonyl)-*N*-((perfluoroethyl)thio)methanesulfonamide (**7a**)

A round-bottom flask, equipped with a magnetic stir bar, was charged with potassium *N*-(methylsulfonyl)methanesulfonamide salt (1.06 g, 5.02 mmol). Subsequently, CHCl_3_ (8 mL) was added using a syringe. Then, the mixture was cooled down to 0 °C and a 1.59 M solution of 1,1,2,2-tetrafluoroethyl hypochlorothioite **1c** in CHCl_3_ was added (2.26 mL, 3.6 mmol). The mixture was stirred at room temperature for 2 h. The reaction mixture was filtered through a sintered funnel and concentrated under reduced pressure to afford **7a** (1.1 g, 87%) as a white solid. *R*_f_ (3:7 EtOAc/hexane): decomposes; m.p.: 41–43 °C; ^1^H NMR (CDCl_3_, 400 MHz): δ 6.12 (tt, *J* = 52.9, 4.1 Hz, 1H), 3.39 (s, 6H); ^13^C{^1^H} NMR (CDCl_3_, 100.6 MHz): δ 120.6 (tt, *J* = 293.6, 29.8 Hz), 109.1 (tt, *J* = 253.3, 34.9 Hz), 43.2; ^19^F NMR (CDCl_3_, 376.5 MHz): δ −98.7 (td, *J* = 9.2, 4.1 Hz, 2F), −134.0 (dt, *J* = 52.9, 9.2 Hz, 2F); FTIR–ATR (neat) ν in cm^–1^: 1353, 1161, 1118, 996, 961, 909, 794, 755, 526, 500, 472, 429; HRMS (TOF EI) for (M)^+·^ C_4_H_7_F_4_NO_4_S_3_^+·^ (*m*/*z*): calcd 304.9468; found 304.9458.

#### 2-((1,1,2,2-Tetrafluoroethyl)thio)benzo[*d*]isothiazol-3(2*H*)-one 1,1-Dioxide (**8a**)

A round-bottom flask, equipped with a magnetic stir bar, was charged with potassium saccharin salt (46.17 g, 209 mmol). Subsequently, CHCl_3_ (100 mL) was added using a syringe. Then, the mixture was cooled down to 0 °C and a 1.59 M solution of 1,1,2,2-tetrafluoroethyl hypochlorothioite **1c** in CHCl_3_ was added (105 mL, 167 mmol). The mixture was stirred at room temperature for 1 h. The reaction mixture was filtered through a sintered funnel and concentrated under reduced pressure to afford **8a** (52 g, 99%) as a white solid. *R*_f_ (1:4 EtOAc/hexane): decomposes; m.p: 75–78 °C; ^1^H NMR (CDCl_3_, 400 MHz): δ 8.17 (d, *J* = 7.7 Hz, 1H), 8.05–7.96 (m, 2H), 7.96–7.89 (m, 1H), 6.12 (tt, *J* = 52.8, 4.3 Hz, 1H); ^13^C{^1^H} NMR (CDCl_3_, 100.6 MHz): δ 158.9, 137.8, 136.5, 135.1, 126.5, 126.2, 120.3 (tt, *J* = 294.3, 30.1), 120.0, 109.2 (tt, *J* = 253.9, 34.6 Hz); ^19^F NMR (CDCl_3_, 376.5 MHz): δ −96.9 (bd, *J* = 225.0 Hz, 1F), −99.8 (bd, *J* = 225.0 Hz, 1F), −133.9 (m, 2F); FTIR–ATR (neat) ν in cm^–1^: 1761, 1338, 1214, 1099, 978, 936, 806, 746, 668, 592, 528, 500; HRMS (TOF ES^+^) for (M + H)^+^ C_9_H_5_F_4_NO_3_S_2_^+^ (*m*/*z*): calcd 315.9720; found 315.9727.

#### 2-((Perfluoroethyl)thio)benzo[*d*]isothiazol-3(2*H*)-one 1,1-Dioxide (**8b**)

*A* round-bottom flask, equipped with a magnetic stir bar, was charged with potassium saccharin salt (20.32 g, 91.83 mmol). Subsequently, the flask was cooled down to 0 °C and a 1.13 M solution of perfluoroethyl hypochlorothioite **1d** in CH_2_Cl_2_ was added (71 mL, 79.85 mmol). The mixture was stirred at room temperature for 1 h. The reaction mixture was filtered through a sintered funnel and concentrated under reduced pressure to afford **8b** (25.55 g, 96%) as a white solid. *R*_f_ (1:4 EtOAc/hexane): decomposes; m.p: 68–70 °C; ^1^H NMR (CDCl_3_, 400 MHz): δ 8.18 (d, *J* = 7.7 Hz, 1H), 8.05–7.98 (m, 2H), 7.96–7.89 (m, 1H); ^13^C{^1^H} NMR (CDCl_3_, 100.6 MHz): δ 158.5, 138.0, 136.5, 135.1, 126.6, 126.1, 122.1, 118.2 (tq, *J* = 41.8, 299.5 Hz), 118.1 (qt, *J* = 286.7, 35.3 Hz); ^19^F NMR (CDCl_3_, 376.5 MHz): δ −82.4 (t, *J* = 3.0 Hz, 3F), −95.36 (m, 2F); FTIR–ATR (neat) ν in cm^–1^: 1764, 1351, 1194, 1096, 930, 749, 671, 590, 576, 529, 499, 414; HRMS (APCI^+^) for (M + H)^+^ C_9_H_5_F_5_NO_3_S_2_^+^ (*m*/*z*): calcd 333.9626; found 333.9619.

#### 3-((1,1,2,2-Tetrafluoroethyl)thio)-1*H*-indole (**9a**)

An 8 mL reaction vial, equipped with a magnetic stir bar, was charged with 1*H*-indole (35 mg, 0.3 mmol). The flask was then evacuated and backfilled with argon three times. Subsequently, anhydrous CH_2_Cl_2_ (1.5 mL) was added using a syringe. Then, reagent **8a** (104 mg, 0.33 mmol) was added to the flask and the mixture was stirred at 40 °C with an aluminum heating block for 1 h. The reaction mixture was diluted with CH_2_Cl_2_, washed with saturated aqueous NaHCO_3_, and dried over MgSO_4_. Upon filtration, the organic layer was concentrated under reduced pressure and purified by flash column chromatography (1:4 EtOAc/hexane) to afford **9a** (71 mg, 95%) as a brownish solid. *R*_f_ (1:4 EtOAc/hexane): 0.39; m.p: 48 °C; ^1^H NMR (CDCl_3_, 400 MHz): δ 8.44 (bs, 1H), 7.88–7.81 (m, 1H), 7.50 (d, *J* = 2.7 Hz, 1H), 7.47–7.39 (m, 1H), 7.36–7.28 (m, 2H), 5.75 (tt, *J* = 53.7, 4.0 Hz, 1H); ^13^C{^1^H} NMR (CDCl_3_, 100.6 MHz): δ 136.1, 133.3, 129.9, 123.6, 122.2 (tt, *J* = 284.4, 28.5 Hz), 121.8, 119.4, 111.9, 109.4 (tt, *J* = 252.8, 36.8 Hz), 94.4 (t, *J* = 3.9 Hz); ^19^F NMR (CDCl_3_, 376.5 MHz): δ −94.4 (td, *J* = 9.8, 4.0 Hz, 2F), −133.7 (dt, *J* = 53.7, 9.8 Hz, 2F); FTIR–ATR (neat) ν in cm^–1^: 3383, 1376, 1095, 1070, 759, 750, 676, 651, 618, 584, 536, 426; HRMS (APCI^+^) for (M + H)^+^ C_10_H_8_F_4_NS^+^ (*m*/*z*): calcd 250.0308; found 250.0302.

### Large-Scale Preparation of **9a**

To a solution of 1*H*-indole (2.34 g, 20 mmol) in CH_2_Cl_2_ (20 mL) was added reagent **8a** (6.93 g, 22 mmol) under vigorous stirring at 0 °C. The reaction mixture was gradually warmed to room temperature and stirred for 16 h. Then, EtOAc (150 mL) was added and the organic phase was washed with saturated aqueous Na_2_CO_3_ (4 × 20 mL). The combined organic fractions were dried with Na_2_SO_4_, filtered, and the solvent evaporated under reduced pressure to afford **9a** (4.95 g, 99%) as a brownish solid.

#### 3-((Perfluoroethyl)thio)-1*H*-indole (**9b**)

To a round-bottom flask containing 1*H*-indole (18 mg, 0.15 mmol) was sequentially added anhydrous CH_2_Cl_2_ (0.6 mL) and reagent **8b** (55 mg, 0.17 mmol) under argon. The mixture was stirred at 40 °C with an aluminum heating block for 24 h. The solvent was then removed under reduced pressure and the residue was purified by column chromatography (1:4 EtOAc/hexane) to afford **9b** (34 mg, 85%) as a yellowish solid. *R*_f_ (1:4 EtOAc/hexane): 0.22; m.p: 62 °C; ^1^H NMR (CDCl_3_, 400 MHz): δ 8.50 (bs, 1H), 7.85–7.78 (m, 1H), 7.53 (d, *J* = 2.8 Hz, 1H), 7.46–7.40 (m, 1H), 7.34–7.27 (m, 2H); ^13^C{^1^H} NMR (CDCl_3_, 100.6 MHz): δ 136.1, 133.5, 130.0, 123.6, 121.8, 124.5–118.0 (m), 119.5, 117.4 (m), 111.8, 94.0 (t, *J* = 3.7 Hz); ^19^F NMR (CDCl_3_, 376.5 MHz): δ −82.47 (t, *J* = 3.4 Hz, 3F), −93.13 (q, *J* = 3.4 Hz, 2F); FTIR–ATR (neat) ν in cm^–1^: 3380, 1316, 1194, 1092, 958, 746, 555, 533, 426; HRMS (APCI^+^) for (M + H)^+^ C_10_H_7_F_5_NS^+^ (*m*/*z*): calcd 268.0214; found 268.0205.

#### 4-((1,1,2,2-Tetrafluoroethyl)thio)phenol (**10a**)

A 5 mL round-bottom flask, equipped with a magnetic stir bar, was charged with phenol (28 mg, 0.3 mmol). The flask was then evacuated and backfilled with argon three times. Subsequently, anhydrous CH_2_Cl_2_ (3 mL) was added using a syringe followed by trifluoromethanesulfonic acid (32 μL, 0.36 mmol). Then, reagent **8a** (114 mg, 0.36 mmol) was quickly added to the flask. The mixture was stirred at room temperature for 16 h. The reaction mixture was diluted with Et_2_O, washed with saturated aqueous NaHCO_3_ and dried over MgSO_4_. Upon filtration, the organic layer was concentrated under reduced pressure and purified by flash column chromatography (1:9 EtOAc/hexane) to afford **10a** (66 mg, 97%) as a yellowish semisolid. *R*_f_ (1:9 EtOAc/hexane): 0.14; ^1^H NMR (CDCl_3_, 400 MHz): δ 7.51 (d, *J* = 8.6 Hz, 2H), 6.85 (d, *J* = 8.6 Hz, 2H), 5.75 (tt, *J* = 53.8, 3.5 Hz, 1H), 5.23 (s, 1H); ^13^C{^1^H} NMR (CDCl_3_, 100.6 MHz): δ 158.0, 139.2, 122.4 (tt, *J* = 283.9, 29.2 Hz), 116.6, 109.6 (tt, *J* = 252.9, 37.6 Hz); ^19^F NMR (CDCl_3_, 376.5 MHz): δ −92.74 (td, *J* = 9.2, 3.4 Hz, 2F), −132.58 (dt, *J* = 53.8, 9.5 Hz, 2F); FTIR–ATR (neat) ν in cm^–1^: 3370, 1586, 1496, 1212, 1113, 996, 833, 524; HRMS (TOF ES^–^) for (M–H)^−^ C_8_H_5_F_4_OS^–^ (*m/z*): calcd 225.0003; found 225.0004.

#### 4-((Perfluoroethyl)thio)phenol (**10b**)^18b^

A 5 mL round-bottom flask, equipped with a magnetic stir bar, was charged with phenol (28 mg, 0.3 mmol). The flask was then evacuated and backfilled with argon three times. Subsequently, anhydrous CH_2_Cl_2_ (3 mL) was added using a syringe followed by trifluoromethanesulfonic acid (32 μL, 0.36 mmol). Then, reagent **8b** (120 mg, 0.36 mmol) was quickly added to the flask. The mixture was stirred at room temperature for 16 h. The reaction mixture was diluted with Et_2_O, washed with saturated aqueous NaHCO_3_, and dried over MgSO_4_. Upon filtration, the organic layer was concentrated under reduced pressure and purified by flash column chromatography (1:9 EtOAc/hexane) to afford **10b** (41 mg, 56%) as a colorless syrup. *R*_f_ (1:4 EtOAc/hexane): 0.29; ^1^H NMR (CDCl_3_, 400 MHz): δ 7.51 (m, 2H), 6.87 (m, 2H), 5.40 (s, 1H); ^13^C{^1^H} NMR (CDCl_3_, 100.6 MHz): δ 158.3, 139.4, 120.4 (m), 119.9 (m), 116.7; ^19^F NMR (CDCl_3_, 376.5 MHz): δ −82.4 (t, *J* = 3.6 Hz, 3F), −92.8 (q, *J* = 3.6 Hz, 2F); FTIR–ATR (neat) ν in cm^–1^: 3286, 1584, 1495, 1433, 1334, 1195, 1088, 960, 831, 749, 523; HRMS (TOF ES^–^) for (M–H)^−^ C_8_H_4_F_5_OS^–^ (*m*/*z*): calcd 242.9909; found 242.9913.

#### *N*-Benzyl-*S*-(1,1,2,2-tetrafluoroethyl)thiohydroxylamine (**11a**)

A 10 mL round-bottom flask, equipped with a magnetic stir bar, was charged with benzylamine (33 μL, 0.3 mmol). The flask was then evacuated and backfilled with argon three times. Subsequently, anhydrous CH_2_Cl_2_ (6 mL) was added using a syringe. Then, reagent **8a** (99 mg, 0.32 mmol) was quickly added to the flask. The mixture was stirred at room temperature for 1 h. The reaction mixture was concentrated under reduced pressure and purified by flash column chromatography (pentane) to afford **11a** (48 mg, 67%) as a yellowish liquid. *R*_f_ (pentane): 0.15; ^1^H NMR (CD_2_Cl_2_, 400 MHz): δ 7.59–7.17 (m, 5H), 5.98 (tt, *J* = 53.7, 4.0 Hz, 1H), 4.19 (d, *J* = 5.6 Hz, 2H), 3.12 (bs, 1H); ^13^C{^1^H} NMR (CD_2_Cl_2_, 100.6 MHz): δ 139.2, 129.2, 128.7, 128.4, 123.8 (tt, *J* = 286.0, 29.1 Hz), 110.1 (tt, *J* = 251.2, 37.0 Hz), 58.5; ^19^F NMR (CD_2_Cl_2_, 376.5 MHz): δ −103.0 (td, *J* = 8.5, 3.8 Hz, 2F), −135.1 (dt, *J* = 53.9, 8.7 Hz, 2F); FTIR–ATR (neat) ν in cm^–1^: 3356, 1214, 1108, 1003, 814, 699; HRMS (APCI^+^) for (M + H)^+^ C_9_H_10_F_4_NS^+^ (*m*/*z*): calcd 240.0465; found 240.0462.

#### *N*-Benzyl-*S*-(perfluoroethyl)thiohydroxylamine (**11b**)

A 10 mL round-bottom flask, equipped with a magnetic stir bar, was charged with benzylamine (22 μL, 0.2 mmol). The flask was then evacuated and backfilled with argon three times. Subsequently, anhydrous CH_2_Cl_2_ (6 mL) was added using a syringe. Then, reagent **8b** (70 mg, 0.21 mmol) was quickly added to the flask. The mixture was stirred at room temperature for 1 h. The reaction mixture was concentrated under reduced pressure and purified by flash column chromatography (pentane) to afford **11b** (39 mg, 76%) as a colorless oil. *R*_f_ (pentane): 0.35; ^1^H NMR (CD_2_Cl_2_, 400 MHz): δ 7.46–7.26 (m, 5H), 4.24 (d, *J* = 5.4 Hz, 2H), 3.19 (bs, 1H); ^13^C{^1^H} NMR (CD_2_Cl_2_, 100.6 MHz): δ 139.1, 129.2, 128.8, 128.5, 121.3 (tq, *J* = 289.7, 39.8 Hz), 119.6 (qt, *J* = 286.2, 37.3 Hz), 58.4; ^19^F NMR (CD_2_Cl_2_, 376.5 MHz): δ −82.77 (t, *J* = 3.1 Hz, 3F), −102.53 (q, *J* = 2.6 Hz, 2F); FTIR–ATR (neat) ν in cm^–1^: 3853, 3744, 2924, 2372, 2320, 1653, 1558, 1541, 1457; HRMS (APCI^–^) for (M–H)^−^ C_9_H_7_F_5_NS^–^ (*m/z*): calcd 256.0225; found 256.0216.

#### 2-((1,1,2,2-Tetrafluoroethyl)disulfaneyl)benzo[*d*]oxazole (**12a**)

A 10 mL round-bottom flask, equipped with a magnetic stir bar, was charged with 2-mercaptobenzoxazole (48 mg, 0.3 mmol). The flask was then evacuated and backfilled with argon three times. Subsequently, anhydrous CH_2_Cl_2_ (2 mL) and anhydrous MeCN (2 mL) were added using a syringe. Then, the mixture was cooled down to 0 °C and reagent **8a** (104 mg, 0.33 mmol) was quickly added to the flask. The mixture was stirred at room temperature for 5 min. The reaction mixture was diluted with EtOAc, washed with saturated aqueous NaHCO_3_, and dried over MgSO_4_. Upon filtration, the organic layer was concentrated under reduced pressure and the organic residue was redissolved in pentane, extracted, and concentrated again under reduced pressure to afford **12a** as a yellow oil (85 mg, 99%). *R*_f_ (1:9 EtOAc/hexane): 0.33; ^1^H NMR (CDCl_3_, 400 MHz): δ 7.74–7.68 (m, 1H), 7.56–7.50 (m, 1H), 7.39–7.33 (m, 2H), 6.11 (tt, *J* = 53.2, 3.5 Hz, 1H); ^13^C{^1^H} NMR (CDCl_3_, 100.6 MHz): δ 159.6, 152.6, 141.8, 125.9, 125.2, 121.3 (t, *J* = 290.5, 30.0 Hz), 120.1, 110.7, 109.2 (t, *J* = 253.7, 36.1 Hz); ^19^F NMR (CDCl_3_, 376.5 MHz): δ −95.02 (td, *J* = 8.6, 3.6 Hz, 2F), −132.96 (dt, *J* = 53.3, 8.6 Hz, 2F); FTIR–ATR (neat) ν in cm^–1^: 1499, 1450, 1237, 1218, 1125, 1096, 1079, 984, 803, 757, 744; HRMS (APCI^–^) for (M–H)^−^ C_9_H_4_F_4_NOS_2_^–^ (*m*/*z*): calcd 281.9676; found 281.9673.

#### 2-((Perfluoroethyl)disulfaneyl)benzo[*d*]oxazole (**12b**)

A 10 mL round-bottom flask, equipped with a magnetic stir bar, was charged with 2-mercaptobenzoxazole (48 mg, 0.3 mmol). The flask was then evacuated and backfilled with argon three times. Subsequently, anhydrous CH_2_Cl_2_ (2 mL) and anhydrous MeCN (2 mL) were added using a syringe. Then, the mixture was cooled down to 0 °C and reagent **8b** (107 mg, 0.32 mmol) was quickly added to the flask. The mixture was stirred at room temperature for 5 min. The reaction mixture was diluted with EtOAc, washed with saturated aqueous NaHCO_3_ and dried over MgSO_4_. Upon filtration, the organic layer was concentrated under reduced pressure and the organic residue was redissolved in pentane, extracted, and concentrated again under reduced pressure to **12b** (81 mg, 89%) as a white-off solid. *R*_f_ decomposes; m.p.: 71–73 °C (decomposes); ^1^H NMR (CDCl_3_, 400 MHz): δ 7.74–7.68 (m, 1H), 7.57–7.51 (m, 1H), 7.40–7.32 (m, 2H); ^13^C{^1^H} NMR (CDCl_3_, 100.6 MHz): δ 158.8, 152.6, 141.9, 125.9, 125.3, 120.28 (tq, 118.0, *J* = 295.8, 41.1 Hz), 118.4 (qt, *J* = 287.0, 36.0 Hz), 110.8; ^19^F NMR (CDCl_3_, 376.5 MHz): δ −82.27 (t, *J* = 3.0 Hz, 3F), −94.91 (q, *J* = 2.9 Hz, 2F); FTIR–ATR (neat) ν in cm^–1^: 1500, 1449, 1232, 1127, 1092, 801, 745; HRMS (APCI^–^) for (M–H)^−^ C_9_H_3_F_5_NOS_2_^–^ (*m/z*): calcd 299.9582; found 299.9575.

#### (Adamantan-1-yloxy)(1,1,2,2-tetrafluoroethyl)sulfane (**13a**)

A 10 mL round-bottom flask, equipped with a magnetic stir bar, was charged with 1-adamantol (46 mg, 0.3 mmol). The flask was then evacuated and backfilled with argon three times. Subsequently, anhydrous CH_2_Cl_2_ (6 mL) was added using a syringe followed by triethylamine (104 μL, 0.75 mmol). Then, reagent **8a** (123 mg, 0.39 mmol) was quickly added to the flask. The mixture was stirred at room temperature for 20 min. The reaction mixture was concentrated under reduced pressure and purified by flash column chromatography (pentane) to afford **13a** (78 mg, 91%) as a colorless oil. *R*_f_ (pentane): 0.26; ^1^H NMR (CDCl_3_, 400 MHz): δ 5.99 (tt, *J* = 53.5, 4.3 Hz, 1H), 2.23 (bs, 3H), 1.80 (m, 6H), 1.61 (m, 6H); ^13^C{^1^H} NMR (CDCl_3_, 100.6 MHz): δ 123.3 (tt, *J* = 286.6, 28.2 Hz), 109.17 (tt, *J* = 252.9, 35.6 Hz), 82.6, 41.6, 35.9, 31.4; ^19^F NMR (CDCl_3_, 376.5 MHz): δ −103.9 (td, *J* = 9.9, 4.3 Hz, 2F), −135.1 (dt, *J* = 53.5, 9.9 Hz, 2F); FTIR–ATR (neat) ν in cm^–1^: 2913, 2855, 1214, 1118, 1000, 892, 819; HRMS (APCI^–^) for (M–H)^−^ C_12_H_15_F_4_OS^–^ (*m*/*z*): calcd 283.0785; found 283.0780.

#### (Adamantan-1-yloxy)(perfluoroethyl)sulfane (**13b**)

A 10 mL round-bottom flask, equipped with a magnetic stir bar, was charged with 1-adamantol (46 mg, 0.3 mmol). The flask was then evacuated and backfilled with argon three times. Subsequently, anhydrous CH_2_Cl_2_ (6 mL) was added using a syringe followed by triethylamine (104 μL, 0.75 mmol). Then, reagent **8b** (130 mg, 0.39 mmol) was quickly added to the flask. The mixture was stirred at room temperature for 10 min. The reaction mixture was concentrated under reduced pressure and purified by flash column chromatography (pentane) to afford **13b** (54 mg, 60%) as a colorless oil. *R*_f_ (pentane): 0.66; ^1^H NMR (CDCl_3_, 400 MHz): δ 2.24 (bs, 3H), 1.81 (m, 6H), 1.69–1.54 (m, 6H); ^13^C{^1^H} NMR (CDCl_3_, 100.6 MHz): δ 121.1 (m), 118.9 (qt, *J* = 286.8, 36.8 Hz), 83.2, 41.6, 35.9, 31.5; ^19^F NMR (CDCl_3_, 376.5 MHz): δ −81.9 (t, *J* = 3.6 Hz, 3F), −102.5 (q, *J* = 3.6 Hz, 2F); FTIR–ATR (neat) ν in cm^–1^: 3853, 3649, 2923, 2852, 1699, 1653, 1558, 1541, 1507, 1457, 1035. After extensive analyses with different spectrometric techniques the molecular peak could not be found; only fragmentation can be described by HRMS (TOF EI) for (Adamantyl)^+^ C_10_H_15_^+^ (*m*/*z*): calcd 135.1168; found 135.1169; (pentafluoroethyl)^+^ C_2_F_5_^+^ (*m*/*z*): calcd 118.9915; found 118.9910; SC_2_F_4_^+^ (*m*/*z*): calcd 131.9651; found 131.9645.

#### 2,2-Dimethyl-5,5-bis((1,1,2,2-tetrafluoroethyl)thio)cyclopentan-1-one (**14a**)

A 0.35 M stock solution of 2,2-dimethylcyclopentan-1-one potassium enolate was prepared using the following procedure: a 5 mL round-bottom flask, equipped with a magnetic stir bar, was charged with 2,2-dimethylcyclopentan-1-one (113 μL, 0.9 mmol). The flask was then evacuated and backfilled with argon three times. Subsequently, anhydrous THF (1.5 mL) was added using a syringe and the mixture was cooled down to −78 °C. Next, a solution of potassium bis(trimethylsilyl)amide (1.0 M in toluene, 1.1 mL, 1.1 mmol) was added dropwise. The mixture was stirred at −78 °C for 30 min. Then, to a 5 mL round-bottom flask, equipped with a magnetic stir bar, was charged with reagent **8a** (237 mg, 0.75 mmol). The flask was then evacuated and backfilled with argon three times. Subsequently, anhydrous THF (2 mL) was added using a syringe. The mixture was cooled down to −78 °C and then, the previously prepared enolate solution (0.86 mL, 0.35 M, 0.3 mmol) was added using a syringe. Then, the mixture was left to stir at room temperature for 3 h. The reaction mixture was diluted with Et_2_O, washed with saturated aqueous NH_4_Cl and dried over MgSO_4_. Upon filtration, the organic layer was concentrated under reduced pressure and purified by flash column chromatography (hexane) to afford **14a** (60 mg, 53%) as a colorless liquid. *R*_f_ (1:9 EtOAc/hexane): 0.31; ^1^H NMR (CD_2_Cl_2_, 400 MHz): δ 5.99 (tdd, *J* = 53.4, 5.1, 2.8 Hz, 2H), 2.64 (t, *J* = 6.8 Hz, 2H), 2.08 (t, *J* = 6.8 Hz, 2H), 1.23 (s, 6H); ^13^C{^1^H} NMR (CD_2_Cl_2_, 100.6 MHz): δ 210.7, 127.1–120.4 (m), 112.4–106.5 (m), 65.3, 44.6, 37.8, 35.1, 26.8; ^19^F NMR (CD_2_Cl_2_, 376.5 MHz): δ −88.02 (dt, *J* = 234.5, 8.3 Hz), −89.89 (ddd, *J* = 234.5, 14.2, 9.2 Hz), −132.4 (m), −134.50 (ddt, *J* = 295.2, 53.5, 9.2 Hz); FTIR–ATR (neat) ν in cm^–1^: 2975, 1743, 1461, 1382, 1208, 1113, 984, 877, 812, 634, 553; HRMS (APCI^+^) for (M + H)^+^ C_11_H_13_F_8_OS_2_^+^ (*m*/*z*): calcd 377.0275; found 377.0268.

#### 2,2-Dimethyl-5,5-bis((perfluoroethyl)thio)cyclopentan-1-one (**14b**)

A 0.35 M stock solution of 2,2-dimethylcyclopentan-1-one potassium enolate was prepared using the following procedure: a 5 mL round-bottom flask, equipped with a magnetic stir bar, was charged with 2,2-dimethylcyclopentan-1-one (113 μL, 0.9 mmol). The flask was then evacuated and backfilled with argon three times. Subsequently, anhydrous THF (1.5 mL) was added using a syringe and the mixture was cooled down to −78 °C. Next, a solution of potassium bis(trimethylsilyl)amide (1.0 M in toluene, 1.1 mL, 1.1 mmol) was added dropwise. The mixture was stirred at −78 °C for 30 min. Then, to a 5 mL round-bottom flask, equipped with a magnetic stir bar, was charged with reagent **8b** (250 mg, 0.75 mmol). The flask was then evacuated and backfilled with argon three times. Subsequently, anhydrous THF (2 mL) was added using a syringe. The mixture was cooled down to −78 °C and then, the previously prepared enolate solution (0.86 mL, 0.35 M, 0.3 mmol) was added using a syringe. Then, the mixture was left to stir at room temperature for 3 h. The reaction mixture was diluted with Et_2_O, washed with saturated aqueous NH_4_Cl and dried over MgSO_4_. Upon filtration, the organic layer was concentrated under reduced pressure and purified by flash column chromatography (hexane) to afford **14b** (47 mg, 38%) as a colorless liquid. *R*_f_ (1:9 EtOAc/hexane): 0.47; ^1^H NMR (CD_2_Cl_2_, 400 MHz): δ 2.71 (t, *J* = 6.8 Hz, 2H), 2.13 (t, *J* = 6.8 Hz, 2H), 1.29 (s, 6H); ^13^C{^1^H} NMR (CD_2_Cl_2_, 100.6 MHz): δ 210.7, 127.1–120.4 (m), 112.4–106.5 (m), 65.3, 44.6, 37.8, 35.1, 26.8; ^19^F NMR (CD_2_Cl_2_, 376.5 MHz): δ −88.02 (dt, *J* = 234.5, 8.3 Hz), −89.89 (ddd, *J* = 234.5, 14.2, 9.2 Hz), −132.4 (m), −134.50 (ddt, *J* = 295.2, 53.5, 9.2 Hz); FTIR–ATR (neat) ν in cm^–1^: 1751, 1328, 1212, 1099, 953, 751; HRMS (APCI^+^) for (M + H)^+^ C_11_H_11_F_10_OS_2_^+^ (*m*/*z*): calcd 413.0086; found 413.0085.

#### Diethyl 2-Benzyl-2-((1,1,2,2-tetrafluoroethyl)thio)malonate (**15a**)

A 5 mL round-bottom flask, equipped with a magnetic stir bar, was charged with NaH (60% in mineral oil, 9 mg, 0.23 mmol). The flask was then evacuated and backfilled with argon three times. Subsequently, anhydrous THF (1.5 mL) was added using a syringe followed by diethyl 2-benzylmalonate (35.5 μL, 0.15 mmol) and the mixture was stirred at room temperature for 15 min. Then, reagent **8a** (118 mg, 0.38 mmol) was quickly added to the flask and the mixture was stirred at room temperature for 15 min. The reaction mixture was diluted with Et_2_O, washed with aqueous NH_4_Cl, and dried over MgSO_4_. Upon filtration, the organic layer was concentrated under reduced pressure and purified by flash column chromatography (1:9 EtOAc/hexane) to afford **15a** (51 mg, 88%) as a colorless oil. *R*_f_ (1:9 EtOAc/hexane): 0.26; ^1^H NMR (CDCl_3_, 400 MHz): δ 7.30–7.18 (m, 5H), 5.80 (tt, *J* = 53.7, 3.5 Hz, 1H), 4.29–4.15 (m, 4H), 3.64 (s, 2H), 1.23 (t, *J* = 7.1 Hz, 6H); ^13^C{^1^H} NMR (CDCl_3_, 100.6 MHz): δ 167.2, 134.1, 130.7, 128.3, 127.8, 124.06 (tt, *J* = 287.4, 29.3 Hz), 109.47 (tt, *J* = 254.4, 36.4 Hz), 64.7, 63.2, 41.3, 13.9; ^19^F NMR (CDCl_3_, 376.5 MHz): δ −88.79 (td, *J* = 8.9, 3.5 Hz, 2F), −132.48 (dt, *J* = 53.7, 8.9 Hz, 2F); FTIR–ATR (neat) ν in cm^–1^: 2985, 1260, 1224, 1114, 990, 860, 810, 701; HRMS (APCI^+^) for (M + H)^+^ C_16_H_19_F_4_O_4_S^+^ (*m*/*z*): calcd 383.0935; found 383.0922.

#### Diethyl 2-Benzyl-2-((perfluoroethyl)thio)malonate (**15b**)

A 5 mL round-bottom flask, equipped with a magnetic stir bar, was charged with NaH (60% in mineral oil, 22 mg, 0.9 mmol). The flask was then evacuated and backfilled with argon three times. Subsequently, anhydrous THF (3 mL) was added using a syringe followed by diethyl 2-benzylmalonate (71 μL, 0.3 mmol) and the mixture was stirred at room temperature for 15 min. Then, reagent **8b** (170 mg, 0.51 mmol) was quickly added to the flask and the mixture was stirred at room temperature for 15 min. The reaction mixture was diluted with Et_2_O, washed with saturated aqueous NH_4_Cl, and dried over MgSO_4_. Upon filtration, the organic layer was concentrated under reduced pressure and purified by flash column chromatography (9:0.5 EtOAc/hexane) to afford **15b** (112 mg, 93%) as a yellowish oil. *R*_f_ (1:9 EtOAc/hexane): 0.35; ^1^H NMR (CDCl_3_, 400 MHz): δ 7.34–7.19 (m, 5H), 4.32–4.18 (m, 4H), 3.68 (s, 2H), 1.26 (t, *J* = 7.2 Hz, 6H); ^13^C{^1^H} NMR (CDCl_3_, 100.6 MHz): δ 166.7, 134.0, 130.7, 128.4, 127.8, 122.1 (tq, *J* = 292.3, 40.9), 118.2 (qt, *J* = 286.8, 34.9 Hz), 65.3, 63.3, 41.2 (d, *J* = 1.9 Hz), 13.8; ^19^F NMR (CDCl_3_, 376.5 MHz): δ −83.44 (t, *J* = 3.5 Hz, 3F), −88.59 (q, *J* = 3.5 Hz, 2F); FTIR–ATR (neat) ν in cm^–1^: 1739, 1311, 1259, 1217, 1095, 1083, 959, 750, 701; HRMS (APCI^+^) for (M + H)^+^ C_16_H_18_F_5_O_4_S^+^ (*m*/*z*): calcd 401.0840; found 401.0828.

#### (*E*/*Z*)-(2-(Naphthalen-2-yl)vinyl)(1,1,2,2-tetrafluoroethyl)sulfane (**16a**)

A 25 mL round-bottom flask, equipped with a magnetic stir bar, was charged with 2-vinylnaphthalene (46 mg, 0.3 mmol). The flask was then evacuated and backfilled with argon three times. Subsequently, anhydrous MeCN (9 mL) was added using a syringe followed by trimethylsilyl chloride (114 μL, 0.9 mmol). Then, reagent **8a** (104 mg, 0.33 mmol) was quickly added to the flask. The mixture was stirred at room temperature for 5 h. Next, 1,8-diazabicyclo[5.4.0]undec-7-ene (DBU, 269 μL, 1.8 mmol) was added and the mixture was left to stir at room temperature for 16 h. Then, the reaction mixture was concentrated under reduced pressure and purified by flash column chromatography (hexane) to afford **16a** (85 mg, 99%) as a white solid as an inseparable 96:4 *E/Z* mixture. *R*_f_ (hexane): 0.24; m.p: 48–50 °C; FTIR–ATR (neat) ν in cm^–1^: 2320, 1699, 1653, 1558, 1541, 1507, 1457, 1105; HRMS (TOF EI) for (M)^+·^ C_14_H_10_F_4_S^+·^ (*m*/*z*): calcd 286.0434; found 286.0435. *E*-isomer: ^1^H NMR (CDCl_3_, 400 MHz): δ 7.87–7.79 (m, 3H), 7.76 (d, *J* = 0.7 Hz, 1H), 7.57 (dt, *J* = 6.7, 3.3 Hz, 1H), 7.53–7.45 (m, 2H), 7.16 (d, *J* = 15.4 Hz, 1H), 6.86 (d, *J* = 15.3 Hz, 1H), 5.92 (tt, *J* = 53.8, 3.2 Hz, 1H); ^13^C{^1^H} NMR (CDCl_3_, 100.6 MHz): δ 141.0, 133.6, 133.5, 132.9, 128.8, 128.4, 127.9, 127.5, 126.8, 126.8, 123.2, 122.7 (tt, *J* = 284.4, 30.0 Hz), 111.5 (t, *J* = 5.0 Hz), 109.8 (tt, *J* = 253.1, 38.3 Hz); ^19^F NMR (CDCl_3_, 376.5 MHz): δ −92.94 (td, *J* = 8.9, 2.8 Hz, 2F), −132.08 (dt, *J* = 54.0, 8.9 Hz, 2F). Selected signals for the *Z-*isomer: ^1^H NMR (CDCl_3_, 400 MHz): δ 6.98 (d, *J* = 10.5 Hz, 1H), 6.53 (d, *J* = 10.6 Hz, 1H); ^19^F NMR (CDCl_3_, 376.5 MHz): δ −93.93 (td, *J* = 8.4, 2.8 Hz, 2F), 131.9–132.1 (m, 2H).

#### (*E*/*Z*)-(2-(Naphthalen-2-yl)vinyl)(perfluoroethyl)sulfane (**16b**)

A 25 mL round-bottom flask, equipped with a magnetic stir bar, was charged with 2-vinylnaphthalene (46 mg, 0.3 mmol). The flask was then evacuated and backfilled with argon three times. Subsequently, anhydrous MeCN (9 mL) was added using a syringe followed by trimethylsilyl chloride (228 μL, 1.8 mmol). Then, reagent **8b** (220 mg, 0.66 mmol) was quickly added to the flask. The mixture was stirred at 65 °C for 16 h. Next, 1,8-diazabicyclo[5.4.0]undec-7-ene (DBU, 269 μL, 1.8 mmol) was added and the mixture was left to stir at room temperature for 16 h. Then, the reaction mixture was concentrated under reduced pressure and purified by flash column chromatography (hexane) to afford **16b** (51 mg, 56%) as a white solid as an inseparable 88:12 *E/Z* mixture. *R*_f_ (hexane): 0.50; m.p: 46–48 °C; FTIR–ATR (neat) ν in cm^–1^: 1507, 1338, 1202, 1092, 947, 862, 819, 795, 748, 624, 479; HRMS (APCI^+^) for (M)^+·^ C_14_H_9_F_5_S^+·^ (*m*/*z*): calcd 304.0340; found 304.0334. *E-*isomer: ^1^H NMR (CDCl_3_, 400 MHz): δ 7.89–7.80 (m, 3H), 7.77 (s, 1H), 7.57 (dd, *J* = 8.6, 1.7 Hz, 1H), 7.54–7.48 (m, 2H), 7.20 (d, *J* = 15.3 Hz, 1H), 6.82 (d, *J* = 15.3 Hz, 1H); ^13^C{^1^H} NMR (CDCl_3_, 100.6 MHz): δ 142.4, 133.8, 133.5, 132.6, 128.8, 128.5, 127.9, 127.8, 127.0, 126.9, 123.2, 120.3 (tq, *J* = 288.8, 40.7 Hz), 118.9 (qt, *J* = 286.3, 37.2 Hz), 110.6 (t, *J* = 5.0 Hz); ^19^F NMR (CDCl_3_, 376.5 MHz): δ −82.98 (t, *J* = 3.3 Hz, 2F), −93.76 (q, *J* = 3.7 Hz, 2F). Selected signals for the *Z-*isomer: ^1^H NMR (CDCl_3_, 400 MHz): δ 7.02 (d, *J* = 10.5 Hz, 1H), 6.48 (d, *J* = 10.5 Hz, 1H); ^19^F NMR (CDCl_3_, 376.5 MHz): δ −83.24 (t, *J* = 3.3 Hz, 2F), −94.69 (q, *J* = 3.6 Hz, 2F).

#### (Phenylethynyl)(1,1,2,2-tetrafluoroethyl)sulfane (**17a**)

A 0.42 M stock solution of lithium phenylacetylide was prepared using the following procedure: a 5 mL round-bottom flask, equipped with a magnetic stir bar, was charged with phenylacetlylene (102 mg, 1.0 mmol). The flask was then evacuated and backfilled with argon three times. Subsequently, anhydrous THF (2 mL) was added using a syringe and the mixture was cooled down to −78 °C. Next, a titrated solution of *n*-BuLi (2.88 M in hexanes, 0.38 mL, 1.1 mmol) was added dropwise. The mixture was stirred at −78 °C for 30 min. To a 5 mL round-bottom flask, equipped with a magnetic stir bar, reagent **8a** (114 mg, 0.36 mmol) was charged. The flask was then evacuated and backfilled with argon three times. Subsequently, anhydrous THF (2.2 mL) was added using a syringe and the mixture was cooled down to −78 °C. Then, the previously prepared solution of lithium phenylacetylide (0.72 mL, 0.3 mmol) was added dropwise to the reaction flask. The reaction mixture was stirred at −78 °C for 15 min and then left to warm up to room temperature. Finally, the crude was cooled down to 0 °C and first quenched with H_2_O (5 mL) and secondly with saturated aqueous NH_4_Cl (5 mL). The mixture is transferred to an extraction funnel and the organic layer is separated and dried over MgSO_4_. Upon filtration, the organic layer was concentrated under reduced pressure and purified by flash column chromatography (pentane) to afford **17a** (59 mg, 84%) as a colorless liquid. *R*_f_ (hexane): 0.40; ^1^H NMR (CDCl_3_, 400 MHz): δ 7.50–7.45 (m, 2H), 7.40–7.30 (m, 3H), 6.05 (tt, *J* = 53.4, 3.8 Hz, 1H); ^13^C{^1^H} NMR (CDCl_3_, 100.6 MHz): δ 132.2, 129.7, 128.5, 121.6, 121.4 (tt, *J* = 290.1, 29.5 Hz), 108.9 (tt, *J* = 254.0, 35.8 Hz), 99.8, 66.9 (s, *J* = 6.6 Hz); ^19^F NMR (CDCl_3_, 376.5 MHz): δ −95.12 (td, *J* = 8.9, 3.8 Hz, 2F), −133.50 (dt, *J* = 53.4, 9.0 Hz, 2F); FTIR–ATR (neat) ν in cm^–1^: 2923, 1653, 1558, 1541, 1457, 465; HRMS (APCI^+^) for (M)^+.^ C_10_H_6_F_4_S^+·^ (*m*/*z*): calcd 234.0126; found 234.0116.

#### (Perfluoroethyl)(phenylethynyl)sulfane (**17b**)^18c^

A 0.41 M stock solution of lithium phenylacetylide was prepared using the following procedure: a 5 mL round-bottom flask, equipped with a magnetic stir bar, was charged with phenylacetlylene (102 mg, 1.0 mmol). The flask was then evacuated and backfilled with argon three times. Subsequently, anhydrous THF (2 mL) was added using a syringe and the mixture was cooled down to −78 °C. Next, a titrated solution of *n-*BuLi (2.58 M in hexanes, 0.43 mL, 1.1 mmol) was added dropwise. The mixture was stirred at −78 °C for 30 min. To a 5 mL round-bottom flask, equipped with a magnetic stir bar, reagent **8b** (120 mg, 0.36 mmol) was charged. The flask was then evacuated and backfilled with argon three times. Subsequently, anhydrous THF (2 mL) was added using a syringe and the mixture was cooled down to −78 °C. Then, the previously prepared solution of lithium phenylacetylide (0.73 mL, 0.3 mmol) was added dropwise to the reaction flask. The reaction mixture was stirred at −78 °C for 15 min and then left to warm up to room temperature. Finally, the crude was cooled down to 0 °C and first quenched with H_2_O (5 mL) and secondly with saturated aqueous NH_4_Cl (5 mL). The mixture is transferred to an extraction funnel and the organic layer is separated and dried over MgSO_4_. Upon filtration, the organic layer was concentrated under reduced pressure and purified by flash column chromatography (pentane) to afford **17b** (40 mg, 53%) as a yellowish semisolid. *R*_f_ (hexane): 0.61; ^1^H NMR (CDCl_3_, 400 MHz): δ 7.57–7.47 (m, 2H), 7.45–7.32 (m, 3H); ^13^C{^1^H} NMR (CDCl_3_, 100.6 MHz): δ 132.4, 129.9, 128.6, 121.7, 119.1 (tq, *J* = 294.8, 40.0), 118.6 (qt, *J* = 287.1, 36.4 Hz), 101.0, 65.9 (t, *J* = 6.4 Hz); ^19^F NMR (CDCl_3_, 376.5 MHz): δ −82.59 (t, *J* = 2.7 Hz, 3F), −94.61 (q, *J* = 2.6 Hz, 2F); FTIR–ATR (neat) ν in cm^–1^: 2922, 2320, 1221, 1109, 958, 753; HRMS (APCI^+^) for (M)^+·^ C_10_H_5_F_5_S^+·^ (*m*/*z*): calcd 252.0032; found 252.0024.

#### [1,1′-Biphenyl]-4-yl(1,1,2,2-tetrafluoroethyl)sulfane (**18a**)

A 0.18 M stock solution of [1,1′-biphenyl]-4-yllithium was prepared using the following procedure: a 10 mL round-bottom flask, equipped with a magnetic stir bar, was charged with 4-bromo-1,1′-biphenyl (233 mg, 1.0 mmol). The flask was then evacuated and backfilled with argon three times. Subsequently, anhydrous THF (5.5 mL) was added using a syringe and the mixture was cooled down to −78 °C. Next, a titrated solution of *n*-BuLi (2.88 M in hexanes, 0.36 mL, 1.0 mmol) was added dropwise. The mixture was stirred at −78 °C for 1.5 h. To a 25 mL round-bottom flask, equipped with a magnetic stir bar, reagent **8a** (114 mg, 0.36 mmol) was charged. The flask was then evacuated and backfilled with argon three times. Subsequently, anhydrous THF (8.4 mL) was added using a syringe and the mixture was cooled down to −78 °C. Then, the previously prepared solution of [1,1′-biphenyl]-4-yllithium (1.8 mL, 0.3 mmol) was added dropwise to the reaction flask. The reaction mixture was stirred at −78 °C for 30 min and then left to warm up to room temperature. Finally, the crude was cooled down to 0 °C and first quenched with H_2_O (3 mL) and secondly with saturated aqueous NH_4_Cl (3 mL). The mixture is transferred to an extraction funnel and the organic layer is separated and dried over MgSO_4_. Upon filtration, the organic layer was concentrated under reduced pressure and purified by flash column chromatography (pentane) to afford **18a** (60 mg, 70%) as a white solid. *R*_f_ (pentane): 0.48; m.p: 42–44 °C; ^1^H NMR (CDCl_3_, 400 MHz): δ 7.75–7.69 (m, 2H), 7.67–7.58 (m, 4H), 7.53–7.45 (m, 2H), 7.44–7.35 (m, 1H), 5.82 (tt, *J* = 53.8, 3.4 Hz, 1H); ^13^C{^1^H} NMR (CDCl_3_, 100.6 MHz): δ 143.68, 139.66, 137.38, 128.96, 128.12, 128.05, 127.20, 122.45 (tt, *J* = 284.9, 29.3 Hz), 122.05, 109.43 (tt, *J* = 253.2, 37.5 Hz); ^19^F NMR (CDCl_3_, 376.5 MHz): δ −91.8 (td, *J* = 9.5, 2.8 Hz, 2F), −133.7 (dt, *J* = 53.8, 9.6 Hz, 2F); FTIR–ATR (neat) ν in cm^–1^: 1477, 1379, 1097, 1005, 836, 760, 688, 673, 626, 473; HRMS (APCI^+^) for (M)^+·^ C_14_H_10_F_4_S^+·^ (*m*/*z*): calcd 286.0439; found 286.0427.

#### [1,1′-Biphenyl]-4-yl(perfluoroethyl)sulfane (**18b**)

A 2.0 M stock solution of [1,1′-biphenyl]-4-yllithium was prepared using the following procedure: a 10 mL round-bottom flask, equipped with a magnetic stir bar, was charged with 4-bromo-1,1′-biphenyl (233 mg, 1.0 mmol). The flask was then evacuated and backfilled with argon three times. Subsequently, anhydrous THF (5.5 mL) was added using a syringe and the mixture was cooled down to −78 °C. Next, a titrated solution of *n*-BuLi (2.58 M in hexanes, 0.39 mL, 1.0 mmol) was added dropwise. The mixture was stirred at −78 °C for 1.5 h. To a 25 mL round-bottom flask, equipped with a magnetic stir bar, reagent **8b** (160 mg, 0.48 mmol) was charged. The flask was then evacuated and backfilled with argon three times. Subsequently, anhydrous THF (8 mL) was added using a syringe and the mixture was cooled down to −78 °C. Then, the previously prepared solution of [1,1′-biphenyl]-4-yllithium (0.2 mL, 0.4 mmol) was added dropwise to the reaction flask. The reaction mixture was stirred at −78 °C for 30 min and then left to warm up to room temperature. Finally, the crude was cooled down to 0 °C and first quenched with H_2_O (3 mL) and secondly with saturated aqueous NH_4_Cl (3 mL). The mixture is transferred to an extraction funnel and the organic layer is separated and dried over MgSO_4_. Upon filtration, the organic layer was concentrated under reduced pressure and purified by flash column chromatography (hexane) to afford **18b** (76 mg, 62%) as a white solid. *R*_f_ (hexane): 0.55; m.p: 54–56 °C; ^1^H NMR (CDCl_3_, 400 MHz): δ 7.72 (m, 2H), 7.67–7.56 (m, 4H), 7.52–7.45 (m, 2H), 7.44–7.36 (m, 1H). ^13^C{^1^H} NMR (CDCl_3_, 100.6 MHz): δ 144.5. 141.2, 139.9, 139.8, 138.0, 129.4, 128.6, 128.5, 127.6, 121.6 (t, *J* = 3.3 Hz), 121.1–114.5 (m); ^19^F NMR (CDCl_3_, 376.5 MHz): δ −82.82 (t, *J* = 3.6 Hz, 3F), −92.15 (q, *J* = 3.6 Hz, 2F); FTIR–ATR (neat) ν in cm^–1^: 2319, 1336, 1204, 1104, 1089, 970, 837, 761, 718, 690; HRMS (APCI^+^) for (M)^+·^ C_14_H_9_F_5_S^+·^ (*m*/*z*): calcd 304.0345; found 304.0333.

#### 1,5-Anhydro-3,4,6-tri-*O*-benzyl-2-deoxy-2-(1,1,2,2-tetrafluoroethyl)thio-d-arabino-hex-1-enitol (**19a**)

A 5 mL round-bottom flask, equipped with a magnetic stir bar, was charged with tri-*O*-benzyl-d-glucal (42 mg, 0.1 mmol) and 3 Å molecular sieves (300 mg, 3 g/mmol glucal). The flask was then evacuated and backfilled with argon three times. Subsequently, anhydrous MeCN (1.5 mL) and reagent **8a** (38 mg, 0.12 mmol) were added. The mixture was stirred at room temperature for 2 h. Then, trimethylsilyl chloride (38 μL, 0.3 mmol) was added and the mixture was stirred until complete consumption of the starting material as monitored by TLC (ca. 3.5 h). Next, 1,8-diazabicyclo(5.4.0)undec-7-ene (45 μL, 0.6 mmol) was added and the reaction mixture was left to stir at room temperature for 16 h. The reaction mixture was diluted with CH_2_Cl_2_, filtered through Celite washed with H_2_O, brine, and dried over MgSO_4_. Upon filtration, the organic layer was concentrated under reduced pressure and purified by flash column chromatography (1:9 EtOAc/hexane) to afford **19a** (44 mg, 80%) as a colorless oil. *R*_f_ (1:9 EtOAc/hexane): 0.19; ^1^H NMR (CDCl_3_, 400 MHz): δ 7.40–7.23 (m, 15H), 6.98 (s, 1H), 5.87 (tdd, *J* = 53.7, 4.9, 2.9 Hz, 1H), 4.74 (d, *J* = 11.1 Hz, 1H), 4.66 (d, *J* = 11.6 Hz, 1H), 4.61 (d, *J* = 11.1 Hz, 1H), 4.58 (d, *J* = 11.6 Hz, 1H), 4.53 (s, 2H), 4.47–4.40 (m, 1H), 4.08 (d, *J* = 4.0 Hz, 1H), 3.91 (dd, *J* = 5.0, 4.3 Hz, 1H), 3.77 (dd, *J* = 10.7, 6.3 Hz, 1H), 3.68 (dd, *J* = 10.7, 4.2 Hz, 1H); ^13^C{^1^H} NMR (CDCl_3_, 100.6 MHz): δ 156.1, 137.8, 137.7, 137.5, 128.7, 128.6, 128.24, 128.2, 128.1, 128.0, 127.9, 127.9, 125.9–119.3 (m), 108.2 (tdd, *J* = 252.7, 38.6, 35.3 Hz), 97.0 (t, *J* = 2.9 Hz), 77.0, 76.3, 73.6, 73.4, 72.9, 67.9; ^19^F NMR (CDCl_3_, 376.5 MHz): δ −92.6 (m, 1F), −95.1 (m, 1F), −133.74 (m, 2F); FTIR–ATR (neat) ν in cm^–1^: 1613, 1454, 1365, 1210, 1184, 1102, 1065, 989, 914, 811, 735, 695; HRMS (TOF ES^+^) for (M + Na)^+^ C_29_H_28_F_4_NaO_4_S^+^ (*m*/*z*): calcd 571.1537; found 571.1540.

#### 1,5-Anhydro-3,4,6-tri-*O*-benzyl-2-deoxy-2-(perfluoroethyl)thio-d-arabino-hex-1-enitol (**19b**)

A 5 mL round-bottom flask, equipped with a magnetic stir bar, was charged with tri-*O*-benzyl-d-glucal (126 mg, 0.3 mmol) and 3 Å molecular sieves (900 mg, 3 g/mmol glucal). The flask was then evacuated and backfilled with argon three times. Subsequently, anhydrous MeCN (9 mL) and reagent **8b** (220 mg, 0.66 mmol) were added. The mixture was stirred at room temperature for 2 h. Then, trimethylsilyl chloride (228 μL, 1.8 mmol) was added and the mixture was stirred until complete consumption of the starting material as monitored by TLC (ca. 4 h). Next, 1,8-diazabicyclo(5.4.0)undec-7-ene (538 μL, 3.6 mmol) was added and the reaction mixture was left to stir at room temperature for 18 h. The reaction mixture was diluted with CH_2_Cl_2_, filtered through Celite washed with H_2_O, brine, and dried over MgSO_4_. Upon filtration, the organic layer was concentrated under reduced pressure and purified by flash column chromatography (hexane to 5:1 EtOAc/hexane) to afford **19b** (130 mg, 78%) as a colorless oil. *R*_f_ (1:4 EtOAc/hexane): 0.43; ^1^H NMR (CDCl_3_, 400 MHz): δ 7.69–7.49 (m, 15H), 7.26 (s, 1H), 5.05 (d, *J* = 11.1 Hz, 1H), 4.97 (d, *J* = 11.5 Hz, 1H), 4.91 (d, *J* = 11.1 Hz, 1H), 4.85 (d, *J* = 11.5 Hz, 1H), 4.80 (s, 2H), 4.67 (dd, *J* = 10.0, 5.4 Hz, 1H), 4.40 (d, *J* = 4.6 Hz, 1H), 4.19 (dd, *J* = 5.8, 4.9 Hz, 1H), 4.07 (dd, *J* = 10.7, 5.9 Hz, 1H), 3.99 (dd, *J* = 10.7, 3.9 Hz, 1H); ^13^C{^1^H} NMR (CDCl_3_, 100.6 MHz): δ 156.6, 137.8, 137.6, 128.6, 128.5, 128.2, 128.1, 128.0, 127.9, 127.9, 127.8, 124–114 (m), 118.9 (qt, *J* = 284.8, 37.2), 97.1 (t, *J* = 2.3 Hz), 77.5, 76.5, 73.8, 73.6, 73.2, 72.2, 67.8; ^19^F NMR (CDCl_3_, 376.5 MHz): δ −82.39 (t, *J* = 3.6 Hz, 3F), −92.79 (q, *J* = 3.4 Hz, 2F); FTIR–ATR (neat) ν in cm^–1^: 1614, 1454, 1329, 1206, 1186, 1091, 1027, 957, 914, 748, 695; HRMS (TOF ES^+^) for (M + Na)^+^ C_29_H_27_F_5_NaO_4_S^+^ (*m*/*z*): calcd 589.1442; found 589.1444.

#### (1*S*,2*R*)-1-Phenyl-2-(((1,1,2,2-tetrafluoroethyl)thio)amino)propan-1-ol (d-(+)-norephedrine-SCF_2_CF_2_H (**20a**)

A 5 mL round-bottom flask, equipped with a magnetic stir bar, was charged with (1*S*,2*R*)-(+)-norephedrine (46 mg, 0.3 mmol). The flask was then evacuated and backfilled with argon three times. Subsequently, anhydrous CH_2_Cl_2_ (3 mL) was added using a syringe. Next, reagent **8a** (284 mg, 0.9 mmol) was quickly added to the flask and the mixture was stirred at room temperature for 3 h. Then, the reaction mixture was concentrated under reduced pressure and purified by flash column chromatography (1:10 MeOH/CH_2_Cl_2_) to afford **20a** (50 mg, 59%) as a colorless syrup. *R*_f_ (1:19 CH_3_OH/CH_2_Cl_2_): 0.67; ^1^H NMR (CDCl_3_, 400 MHz): δ 7.41–7.27 (m, 5H), 5.90 (tt, *J* = 53.8, 3.9 Hz, 1H), 4.87 (d, *J* = 3.9 Hz, 1H), 3.33–3.21 (m, 1H), 2.88 (bd, *J* = 4.9 Hz, 1H), 2.17 (bs, *J* = 69.3 Hz, 1H), 1.04 (d, *J* = 6.7 Hz, 3H); ^13^C{^1^H} NMR (CDCl_3_, 100.6 MHz): 140.7, 128.6, 128.0, 126.3, 122.9 (tt, *J* = 285.5, 28.0 Hz), 109.5 (tt, *J* = 250.8, 37.6 Hz), 75.4, 63.3, 14.9; ^19^F NMR (CDCl_3_, 376.5 MHz): δ −102.45 (dtd, *J* = 244.7, 8.4, 4.0 Hz, 1F), −103.18 (dtd, *J* = 20.8, 8.8, 4.0 Hz, 1F), −134.74 (dt, *J* = 53.7, 8.5 Hz, 2F); FTIR–ATR (neat) ν in cm^–1^: 3350, 2310, 1699, 1684, 1653, 1558, 1541, 1507, 1457, 1111, 703; HRMS (APCI^–^) for (M–H)^−^ C_11_H_12_F_4_NOS^–^ (*m*/*z*): calcd 282.0581; found 282.0574.

#### (1*S*,2*R*)-2-(((Perfluoroethyl)thio)amino)-1-phenylpropan-1-ol (d-(+)-norephedrine-SCF_2_CF_3_ (**20b**)

A 5 mL round-bottom flask, equipped with a magnetic stir bar, was charged with (1*S*,2*R*)-(+)-norephedrine (46 mg, 0.3 mmol). The flask was then evacuated and backfilled with argon three times. Subsequently, anhydrous CH_2_Cl_2_ (3 mL) was added using a syringe. Next, reagent **8b** (300 mg, 0.9 mmol) was quickly added to the flask and the mixture was stirred at room temperature for 3 h. Then, the reaction mixture was concentrated under reduced pressure and purified by flash column chromatography (1:10 MeOH/CH_2_Cl_2_) to afford **20b** (81 mg, 90%) as a colorless syrup. *R*_f_ (1:19 CH_3_OH/CH_2_Cl_2_): 0.71; ^1^H NMR (CDCl_3_, 400 MHz): δ 7.42–7.28 (m, 5H), 4.84 (d, *J* = 3.9 Hz, 1H), 3.37–3.24 (m, 1H), 2.97 (d, *J* = 4.4 Hz, 1H), 2.26 (s, *J* = 37.0 Hz, 1H), 1.05 (d, *J* = 6.7 Hz, 3H); ^13^C{^1^H} NMR (CDCl_3_, 100.6 MHz): 140.5, 128.6, 128.0, 126.4, 120.3 (tq, *J* = 290.5, 38.9 Hz), 119.0 (qt, *J* = 287.4, 37.2 Hz), 75.7, 63.2, 14.7; ^19^F NMR (CDCl_3_, 376.5 MHz): δ −82.56 (t, *J* = 3.1 Hz), −102.79 (dq, *J* = 245.6, 2.8 Hz), −103.52 (dq, *J* = 245.8, 2.8 Hz); FTIR–ATR (neat) ν in cm^–1^: 3370, 2310, 1699, 1684, 1653, 1558, 1541, 1507, 1457, 1208; HRMS (TOF ES^–^) for (M–H)^−^ C_11_H_11_F_5_NOS^–^ (*m*/*z*): calcd 300.0487; found 300.0479.

#### N-Methyl-*N*-(3-phenyl-3-(4-(trifluoromethyl)phenoxy)propyl)-*S*-(1,1,2,2-tetrafluoroethyl)thiohydroxylamine (Fluoxetine-SCF_2_CF_2_H, **21a**)

A 10 mL round-bottom flask, equipped with a magnetic stir bar, was charged with fluoxetine (155 mg, 0.5 mmol). The flask was then evacuated and backfilled with argon three times. Subsequently, anhydrous CH_2_Cl_2_ (5 mL) was added using a syringe. Next, reagent **8a** (237 mg, 0.75 mmol) was quickly added to the flask followed by triethylamine (77 μL, 0.55 mmol). The mixture was stirred at room temperature for 3 h. Lastly, the reaction mixture was concentrated under reduced pressure and purified by flash column chromatography (1:9 EtOAc/hexane) to afford **21a** (170 mg, 77%) as a colorless syrup. *R*_f_ (1:4 EtOAc/hexane): 0.45; ^1^H NMR (CDCl_3_, 400 MHz): δ 7.46 (d, *J* = 9.0 Hz, 2H), 7.41–7.34 (m, 4H), 7.34–7.26 (m, 1H), 6.92 (d, *J* = 8.6 Hz, 2H), 5.82 (tt, *J* = 54.1, 3.5 Hz, 1H), 5.23 (dd, *J* = 8.6, 4.6 Hz, 1H), 3.32–3.16 (m, 2H), 2.98 (s, 3H), 2.35–2.12 (m, 2H); ^13^C{^1^H} NMR (CDCl_3_, 100.6 MHz): 160.6 (bq, *J* = 1.1 Hz), 140.9, 129.0, 128.1, 126.9 (q, *J* = 3.8 Hz), 125.9, 125.7 (tt, *J* = 289.4, 30.4 Hz), 124.8 (q, *J* = 271.5 Hz), 123.1 (q, *J* = 32.7 Hz), 115.9, 109.6 (tt, *J* = 251.1, 38.0 Hz), 77.8, 56.9, 48.4, 37.2; ^19^F NMR (CDCl_3_, 376.5 MHz): δ −61.59 (s, 3F), −97.63 (bs, 2F), −133.77 (bd, *J* = 54.3 Hz, 2F); FTIR–ATR (neat) ν in cm^–1^: 1615, 1517, 1324, 1248, 1105, 1066, 835, 812, 701; HRMS (APCI^+^) for (M + H)^+^ C_19_H_19_F_7_NOS^+^ (*m*/*z*): calcd 442.1070; found 442.1063.

#### *N*-Methyl-*S*-(perfluoroethyl)-*N*-(3-phenyl-3-(4-(trifluoromethyl)phenoxy)propyl)thiohydroxylamine (Fluoxetine-SCF_2_CF_3_, **21b**)

A 10 mL round-bottom flask, equipped with a magnetic stir bar, was charged with fluoxetine (155 mg, 0.5 mmol). The flask was then evacuated and backfilled with argon three times. Subsequently, anhydrous CH_2_Cl_2_ (5 mL) was added using a syringe. Next, reagent **8b** (250 mg, 0.75 mmol) was quickly added to the flask followed by triethylamine (77 μL, 0.55 mmol). The mixture was stirred at room temperature for 3 h. Lastly, the reaction mixture was concentrated under reduced pressure and purified by flash column chromatography (1:9 EtOAc/hexane) to afford **21b** as a colorless syrup (200 mg, 87%). *R*_f_ (1:4 EtOAc/hexane): 0.51; ^1^H NMR (CDCl_3_, 400 MHz): δ 7.47 (d, *J* = 8.5 Hz, 2H), 7.42–7.35 (m, 4H), 7.35–7.27 (m, 1H), 6.94 (d, *J* = 8.5 Hz, 2H), 5.23 (dd, *J* = 8.6, 4.5 Hz, 1H), 3.39–3.21 (m, 2H), 3.02 (s, 3H), 2.36–2.25 (m, 1H), 2.25–2.13 (m, 1H); ^13^C{^1^H} NMR (CDCl_3_, 100.6 MHz): 160.6 (bq, *J* = 1.0 Hz), 140.8, 129.1, 128.2, 126.9 (q, *J* = 3.8 Hz), 125.9, 123.2 (q, *J* = 32.7 Hz), 123.0 (tq, *J* = 294.2, 40.0 Hz) 125.0 (q, *J* = 270.5 Hz), 115.9, 118.7 (qt, *J* = 285.4, 36.7 Hz), 77.7, 56.9, 48.2, 37.2; ^19^F NMR (CDCl_3_, 376.5 MHz): δ −61.63 (s, 3F), −83.43 (s, 2F), −98.99 (bs, 2F); FTIR–ATR (neat) ν in cm^–1^: 1615, 1517, 1325, 1249, 1202, 1108, 1067, 948, 835, 700; HRMS (APCI^+^) for (M + H)^+^ C_19_H_18_F_8_NOS^+^ (*m*/*z*): calcd 460.0976; found 460.0968.

#### 2-((1-Benzylpiperidin-4-yl)methyl)-5,6-dimethoxy-2-((1,1,2,2-tetrafluoro-ethyl)thio)-2,3-dihydro-1*H*-inden-1-one (Donepezil-SCF_2_CF_2_H, **22a**)

To a solution of donepezil (114 mg, 0.3 mmol) in anhydrous THF (1.5 mL) at −78 °C was added KHMDS (1.0 M in toluene, 0.4 mL, 0.38 mmol) and stirred at the same temperature for 1 h. Then, a solution in anhydrous THF (2 mL) of the reagent **8a** (142 mg, 0.45 mmol) was added dropwise under argon to the donepezil potassium enolate solution and the reaction mixture was stirred at −78 °C for 1 h. Next, water was added to the reaction mixture (5 mL) and the product was extracted successively with CH_2_Cl_2_ (3 × 20 mL). The combined organic fractions were dried with Na_2_SO_4_, filtered, and evaporated under reduced pressure. The organic crude was purified by flash column chromatography (from CH_2_Cl_2_ to 1:19 CH_3_OH/CH_2_Cl_2_) to afford **22a** (152 mg, 99%) as a brown syrup. *R*_f_ (1:19 CH_3_OH/CH_2_Cl_2_): 0.35; ^1^H NMR (CDCl_3_, 400 MHz): δ 7.34–7.20 (m, 5H), 7.20 (s, 1H), 6.83 (s, 1H), 5.76 (tt, *J* = 53.8, 3.8 Hz, 1H), 3.98 (s, 3H), 3.92 (s, 3H), 3.57 (d, *J* = 17.8 Hz, 1H), 3.47 (s, 2H), 3.42 (d, *J* = 17.9 Hz, 1H), 2.81 (bd, *J* = 8.9 Hz, 2H), 2.07–1.86 (m, 3H), 1.82 (dd, *J* = 14.4, 7.1 Hz, 1H), 1.68–1.46 (m, 3H), 1.42–1.21 (m, 2H); ^13^C{^1^H} NMR (CDCl_3_, 100.6 MHz): 201.1, 156.6, 150.1, 145.8, 138.3, 129.3, 128.2, 127.0, 126.8, 124.3 (tt, *J* = 287.1, 29.4 Hz), 109.4 (tt, *J* = 253.8, 36.2 Hz), 107.1, 105.2, 63.4, 60.0, 56.4, 56.2, 53.6, 53.5, 44.7, 41.8, 33.8, 33.3, 33.0; ^19^F NMR (CDCl_3_, 376.5 MHz): δ −87.07 (m, 2F), −132.39 (ddt, *J* = 294.9, 54.0, 10.0 Hz, 1F), −133.29 (ddt, *J* = 294.9, 54.2, 10.0 Hz, 1F); FTIR–ATR (neat) ν in cm^–1^: 1701, 1592, 1504, 1457, 1311, 1271, 1115, 742; HRMS (TOF ES^+^) for (M + H)^+^ C_26_H_30_F_4_NO_3_S^+^ (*m*/*z*): calcd 512.1877; found 512.1878.

#### (2*E*,4*E*)-5-(Benzo[*d*][1,3]dioxol-5-yl)-1-(piperidin-1-yl)-2-((1,1,2,2-tetrafluoro-ethyl)thio)penta-2,4-dien-1-one (**23aE**) and (2*Z*,4*E*)-5-(Benzo[*d*][1,3]dioxol-5-yl)-1-(piperidin-1-yl)-5-((1,1,2,2-tetrafluoroethyl)thio)penta-2,4-dien-1-one (**23aZ**)

A 10 mL round-bottom flask, equipped with a magnetic stir bar, was charged with piperine (86 mg, 0.3 mmol). The flask was then evacuated and backfilled with argon three times. Subsequently, anhydrous CH_2_Cl_2_ (3 mL) was added using a syringe followed by trimethylsilyl chloride (46 μL, 0.36 mmol). Then, reagent **8a** (208 mg, 0.66 mmol) was quickly added to the flask. The mixture was stirred at room temperature for 18 h. Then, the reaction mixture was concentrated under reduced pressure and the crude purified by flash column chromatography (from CH_2_Cl_2_ to 1:19 CH_3_OH/CH_2_Cl_2_) to afford **23a*E*** (82 mg, 66%) as a yellow solid and **23a*Z*** (19 mg, 15%) as a yellow solid. *E*-isomer: *R*_f_ (1:19 CH_3_OH/CH_2_Cl_2_): 0.70; ^1^H NMR (CDCl_3_, 400 MHz): δ 7.19 (dd, *J* = 15.4, 10.7 Hz, 1H), 7.03 (s, 1H), 7.02 (d, *J* = 10.7 Hz, 1H), 6.93 (dd, *J* = 8.1, 1.6 Hz, 1H), 6.80 (d, *J* = 15.4 Hz, 1H), 6.79 (d, *J* = 8.1 Hz, 1H), 6.06 (tt, *J* = 53.5, 4.2 Hz, 1H), 5.98 (s, 2H), 3.72–3.30 (m, 4H), 1.73–1.52 (m, 6H); ^13^C{^1^H} NMR (CDCl_3_, 100.6 MHz): δ 167.8, 149.0, 148.5, 145.9, 141.3, 130.5, 123.4, 122.8 (tt, *J* = 286.8, 28.8 Hz), 121.9, 117.3 (t, *J* = 2.8 Hz), 109.3 (tt, *J* = 253.4, 35.6 Hz), 108.7, 106.2, 101.6, 48.6 (bs), 43.7 (bs), 25.9 (bs), 24.6; ^19^F NMR (CDCl_3_, 376.5 MHz): δ −91.83 (td, *J* = 9.8, 4.1 Hz, 2H), −133.97 (dt, *J* = 53.3, 9.9 Hz, 2F); FTIR–ATR (neat) ν in cm^–1^: 2940, 2320, 1624, 1490, 1447, 1255, 1108, 1038, 977, 810; HRMS (TOF ES^+^) for (M + H)^+^ C_19_H_20_F_4_NO_3_S^+^ (*m*/*z*): calcd 418.1095; found 418.1093. *Z*-isomer: *R*_f_ (1:19 CH_3_OH/CH_2_Cl_2_): 0.76; ^1^H NMR (CDCl_3_, 400 MHz): δ 6.93 (d, *J* = 1.6 Hz, 1H), 6.93 (d, *J* = 11.1 Hz, 1H), 6.88 (dd, *J* = 8.1, 1.6 Hz, 1H), 6.78 (d, *J* = 8.0 Hz, 1H), 6.73 (d, *J* = 15.5 Hz, 1H), 6.59 (dd, *J* = 15.5, 11.1 Hz, 1H), 6.15 (tt, *J* = 53.4, 4.6 Hz, 1H), 5.99 (s, 2H), 3.69 (d, *J* = 5.5 Hz, 2H), 3.43–3.34 (m, 2H), 1.78–1.53 (m, 6H); ^13^C{^1^H} NMR (CDCl_3_, 100.6 MHz): δ 165.7, 149.0, 148.5, 145.8, 140.2, 130.3, 123.4, 122.8 (tt, *J* = 286.4, 29.2 Hz), 121.7, 117.4 (t, *J* = 3.0 Hz), 109.3 (tt, *J* = 253.3, 34.8 Hz), 108.8, 106.0, 101.6, 48.2, 43.1, 26.4, 25.7, 24.6; ^19^F NMR (CDCl_3_, 376.5 MHz): δ −93.01 (bs, 2F), −134.53 (bd, *J* = 53.2 Hz, 2F); FTIR–ATR (neat) ν in cm^–1^: 2940, 2859, 1620, 1504, 1490, 1445, 1254, 1209, 1107, 1038, 993, 972, 810; HRMS (TOF ES^+^) for (M + H)^+^ C_19_H_20_F_4_NO_3_S^+^ (*m*/*z*): calcd 418.1095; found 418.1091.

#### 5-Benzoyl-7-((1,1,2,2-tetrafluoroethyl)thio)-2,3-dihydro-1*H*-pyrrolizine-1-carboxylic acid (Ketorolac-SCF_2_CF_2_H, **24a**)

A 10 mL round-bottom flask, equipped with a magnetic stir bar, was charged with ketorolac (77 mg, 0.3 mmol). The flask was then evacuated and backfilled with argon three times. Subsequently, anhydrous CH_2_Cl_2_ (3 mL) was added using a syringe followed by trimethylsilyl chloride (76 μL, 0.6 mmol). Then, reagent **8a** (189 mg, 0.6 mmol) was quickly added to the flask. The mixture was stirred at room temperature for 18 h. Then, the reaction mixture was concentrated under reduced pressure and the crude purified by flash column chromatography (10:1:0.1 CH_2_Cl_2_/MeCN/AcOH) to afford **24a** (101 mg, 87%) as a purple syrup. *R*_f_ (1:19 CH_3_OH/CH_2_Cl_2_): 0.29; ^1^H NMR (CDCl_3_, 400 MHz): δ 10.17 (bs, 1H), 7.90–7.71 (m, 2H), 7.64–7.55 (m, 1H), 7.48 (t, *J* = 7.5 Hz, 2H), 6.99 (s, 1H), 5.79 (tt, *J* = 53.7, 3.5 Hz, 1H), 4.67–4.50 (m, 2H), 4.16 (dd, *J* = 9.0, 4.2 Hz, 1H), 3.03–2.80 (m, 2H); ^13^C{^1^H} NMR (CDCl_3_, 100.6 MHz): δ 185.3, 176.67, 147.3, 138.2, 132.3, 131.4, 129.1, 128.6, 128.2, 122.1 (tt, *J* = 284.2, 29.2 Hz), 109.5 (tt, *J* = 252.9, 37.3 Hz), 95.3 (t, *J* = 4.0 Hz), 48.9, 42.2, 32.3; ^19^F NMR (CDCl_3_, 376.5 MHz): δ −92.83 (dtd, *J* = 231.8, 9.4, 3.4 Hz, 1F), −93.5 (dtd, *J* = 231.4, 9.4, 3.7 Hz, 1F), −132.9 (m, 2F); FTIR–ATR (neat) ν in cm^–1^: 2934, 2375, 2320, 1716, 1633, 1457, 1393, 1258, 1213, 1110, 998, 725; HRMS (TOF ES^+^) for (2 M + Na)^+^ C_34_H_26_F_8_N_2_NaO_6_S_2_^+^ (*m*/*z*): calcd 797.0997; found 797.1001.

#### 2-(6-Methoxy-7-((1,1,2,2-tetrafluoroethyl)thio)naphthalen-2-yl)propanoic acid (**25a**)

A 5 mL round-bottom flask, equipped with a magnetic stir bar, was charged with *rac*-naproxen (69.1 mg, 0.3 mmol). The flask was then evacuated and backfilled with argon three times. Subsequently, anhydrous CHCl_3_ (3 mL) was added using a syringe followed by trifluoromethanesulfonic acid (32 μL, 0.36 mmol). Then, reagent **8a** (141.8 mg, 0.45 mmol) was quickly added to the flask. The mixture was stirred at 40 °C with an aluminum heating block for 16 h. The reaction mixture was diluted with Et_2_O, washed with saturated aqueous NaHCO_3_ and dried over MgSO_4_. Upon filtration, the organic layer was concentrated under reduced pressure and purified by flash column chromatography (10:1:0.1 CH_2_Cl_2_/MeCN/AcOH) to afford **25a** (102 mg, 93%) as a purple syrup as an inseparable 81:19 C10/C17 mixture. C10-isomer: *R*_f_ (1:19 CH_3_OH/CH_2_Cl_2_): 0.40; ^1^H NMR (CDCl_3_, 400 MHz): δ 11.0 (bs, 1H), 8.4 (d, *J* = 8.9 Hz, 1H), 7.8 (d, *J* = 9.1 Hz, 1H), 7.6 (d, *J* = 2.0 Hz, 1H), 7.5 (dd, *J* = 8.9, 2.0 Hz, 1H), 7.2 (d, *J* = 9.1 Hz, 1H), 5.7 (tt, *J* = 53.7, 4.1 Hz, 1H), 3.9 (s, 2H), 3.8 (q, *J* = 7.1 Hz, 1H), 1.5 (d, *J* = 7.1 Hz, 3H); ^13^C{^1^H} NMR (CDCl_3_, 100.6 MHz): δ 180.9, 161.0, 136.9, 135.8, 134.0, 129.5, 128.2, 126.8, 126.0, 125.1, 122.6 (tt, *J* = 287.2, 28.53 Hz), 113.4, 109.7 (tt, *J* = 253.3, 36.3 Hz), 56.9, 45.2, 18.1; ^19^F NMR (CDCl_3_, 376.5 MHz): δ −92.6 (td, *J* = 10.2, 4.1 Hz, 1F), −133.1 (dt, *J* = 53.9, 10.2 Hz, 1F); FTIR–ATR (neat) ν in cm^–1^: 2978, 1706, 1595, 1498, 1274, 1207, 1106, 1065, 979, 806; HRMS (TOF ES^+^) for (M + Na)^+^ C_16_H_14_F_4_NaO_3_S^+^ (*m*/*z*): calcd 385.0492; found 385.0474. Selected signals for the C17-isomer: ^1^H NMR (CDCl_3_, 400 MHz): δ 8.2 (d, *J* = 8.8 Hz, 1H), 7.8 (d, *J* = 9.1 Hz, 1H), 7.1–7.1 (m, 1H), 6.1 (tt, *J* = 53.6, 4.4 Hz, 1H), 3.9 (s, 3H). ^19^F NMR (CDCl_3_, 376.5 MHz): δ −95.7 (td, *J* = 9.9, 4.4 Hz, 1F), −135.0 (dt, *J* = 53.6, 10.0 Hz, 1F).

#### 2-(6-Methoxy-7-((1,1,2,2-tetrafluoroethyl)thio)naphthalen-2-yl)propanoic acid (**25b**)

*A* 5 mL round-bottom flask, equipped with a magnetic stir bar, was charged with *rac*-naproxen (69.1 mg, 0.3 mmol). The flask was then evacuated and backfilled with argon three times. Subsequently, anhydrous CHCl_3_ (3 mL) was added using a syringe followed by trifluoromethanesulfonic acid (32 μL, 0.36 mmol). Then, reagent **8b** (150.0 mg, 0.45 mmol) was quickly added to the flask. The mixture was stirred at 70 °C with an aluminum heating block for 16 h. The reaction mixture was diluted with Et_2_O, washed with saturated aqueous NaHCO_3_ and dried over MgSO_4_. Upon filtration, the organic layer was concentrated under reduced pressure and purified by flash column chromatography (10:1:0.1 CH_2_Cl_2_/MeCN/AcOH) to afford **25b** (105 mg, 92%) as a purple syrup. *R*_f_ (1:19 CH_3_OH/CH_2_Cl_2_): 0.58; ^1^H NMR (CDCl_3_, 400 MHz): δ 10.0 (bs, 1H), 8.46 (d, *J* = 8.8 Hz, 1H), 7.98 (d, *J* = 9.0 Hz, 1H), 7.73 (d, *J* = 1.9 Hz, 1H), 7.59 (dd, *J* = 8.8, 1.9 Hz, 1H), 7.33 (d, *J* = 9.0 Hz, 1H), 4.03 (s, 3H), 3.91 (q, *J* = 7.1 Hz, 1H), 1.61 (d, *J* = 7.1 Hz, 3H); ^13^C{^1^H} NMR (CDCl_3_, 100.6 MHz): δ 180.5, 161.2, 136.7, 135.6, 134.2, 129.3, 128.2, 126.7, 125.7, 120.1 (m), 117.3 (m), 113.3, 103.8, 56.8, 45.0, 18.0; ^19^F NMR (CDCl_3_, 376.5 MHz): δ −83.1 (t, *J* = 3.5 Hz, 3F), −91.1 (q, *J* = 3.5 Hz, 2F); FTIR–ATR (neat) ν in cm^–1^: 2917, 1704, 1596, 1458, 1328, 1274, 1196, 1096, 951, 806, 748; HRMS (TOF ES^+^) for (M + Na)^+^ C_16_H_13_F_5_NaO_3_S^+^ (*m*/*z*): calcd 403.0398; found 403.0378.

#### 5-Bromo-3-((1,1,2,2-tetrafluoroethyl)thio)-1*H*-indole (**26a**)

To a solution of 5-bromoindole (3.92 g, 20 mmol) in CH_2_Cl_2_ (20 mL) was added reagent **8a** (6.93 g, 22 mmol) under vigorous stirring at 0 °C. The reaction mixture was gradually warmed to room temperature and stirred for 16 h. Then, EtOAc (150 mL) was added and the organic phase was washed with saturated aqueous Na_2_CO_3_ (4 × 20 mL). The combined organic fractions were dried with Na_2_SO_4_, filtered, and the solvent evaporated under reduced pressure to afford **26a** (6.33 g, 96%) as a brownish solid. *R*_f_ (3:7 EtOAc/hexane): 0.31; m.p: 50 °C; ^1^H NMR (CDCl_3_, 400 MHz): δ 8.48 (s, 1H), 7.95 (bs, 1H), 7.45 (d, *J* = 2.8 Hz, 1H), 7.35 (dd, *J* = 8.6, 1.9 Hz, 1H), 7.22 (d, *J* = 8.7 Hz, 1H), 5.82 (tt, *J* = 53.7, 3.7 Hz, 1H); ^13^C{^1^H} NMR (CDCl_3_, 100.6 MHz): δ 134.8, 134.3, 131.7, 126.6, 122.1, 122.1 (tt, *J* = 285.5, 30.0 Hz), 115.3, 113.3, 109.4 (tt, *J* = 252.8, 37.2 Hz), 94.1 (t, *J* = 4.0 Hz); ^19^F NMR (CDCl_3_, 376.5 MHz): δ −93.47 (td, *J* = 9.4, 3.7 Hz, 2F), −133.05 (dt, *J* = 53.7, 9.4 Hz, 2F); FTIR–ATR (neat) ν in cm^–1^: 3853, 3470, 2321, 1457, 1208, 1103, 994, 799, 585, 513; HRMS (APCI^+^) for (M + H)^+^ C_10_H_7_BrF_4_NS^+^ (*m*/*z*): calcd 327.9413; found 327.9403.

#### 1-Methyl-3-((1,1,2,2-tetrafluoroethyl)thio)-1*H*-indole (**27a**)

To a solution of 1-methyl-1*H*-indole (2.62 g, 20 mmol) in CH_2_Cl_2_ (20 mL) was added reagent **8a** (6.93 g, 22 mmol) under vigorous stirring at 0 °C. The reaction mixture was gradually warmed to room temperature and stirred for 16 h. Then, EtOAc (150 mL) was added and the organic phase was washed with saturated aqueous Na_2_CO_3_ (4 × 20 mL). The combined organic fractions were dried with Na_2_SO_4_, filtered and the solvent evaporated under reduced pressure to afford **27a** (5.20 g, 98%) as a brownish solid. *R*_f_ (1:9 EtOAc/hexane): 0.31; ^1^H NMR (CDCl_3_, 400 MHz): δ 7.84 (bd, *J* = 7.4 Hz, 1H), 7.42–7.29 (m, 4H), 5.76 (tt, *J* = 53.6, 4.1 Hz, 1H), 3.82 (s, 3H); ^13^C{^1^H} NMR (CDCl_3_, 100.6 MHz): δ 137.4, 137.3, 130.7, 123.0, 122.1 (tt, *J* = 283.9, 28.1 Hz), 121.4, 119.4, 110.1, 109.33 (tt, *J* = 252.6, 36.5 Hz), 91.8 (t, *J* = 3.9 Hz); ^19^F NMR (CDCl_3_, 376.5 MHz): δ −94.99 (td, *J* = 10.0, 4.1 Hz, 2F), −133.96 (dt, *J* = 53.6, 10.0 Hz, 2F); FTIR–ATR (neat) ν in cm^–1^: 1512, 1210, 1103, 1080, 996, 972, 812, 741, 543, 426; HRMS (APCI^+^) for (M + H)^+^ C_11_H_10_F_4_NS^+^ (*m*/*z*): calcd 264.0465; found 264.0459.

#### 5-Chloro-3-((1,1,2,2-tetrafluoroethyl)thio)-1*H*-indole (**28a**)

To a solution of 5-chloroindole (45.5 mg, 0.3 mmol) in CH_2_Cl_2_ (1.5 mL) was added reagent **8a** (104 mg, 0.33 mmol) under vigorous stirring at 0 °C. The reaction mixture was gradually warmed to room temperature and stirred for 16 h. Then, EtOAc (50 mL) was added and the organic phase was washed with saturated aqueous Na_2_CO_3_ (4 × 10 mL). The combined organic fractions were dried with Na_2_SO_4_, filtered, and the solvent evaporated under reduced pressure to afford **28a** (77 mg, 90%) as a brownish solid. *R*_f_ (1:4 EtOAc/hexane): 0.25; m.p: 66–67 °C; ^1^H NMR (CDCl_3_, 400 MHz): δ 8.59 (bs, 1H), 7.77 (bd, *J* = 1.8 Hz, 1H), 7.53 (d, *J* = 2.8 Hz, 1H), 7.33 (dd, *J* = 8.6, 0.4 Hz, 1H), 7.24 (d, *J* = 8.7 Hz, 2 Hz, 1H), 5.76 (tt, *J* = 53.7, 3.7 Hz, 1H); ^13^C{^1^H} NMR (CDCl_3_, 100.6 MHz): δ 134.5, 134.5, 131.2, 127.8, 124.1, 122.1 (tt, *J* = 284.3, 29.0 Hz), 119.1, 113.0, 109.4 (tt, *J* = 252.8, 37.1 Hz), 94.3 (t, *J* = 3.8 Hz); ^19^F NMR (CDCl_3_, 376.5 MHz): δ −93.53 (td, *J* = 9.2, 3.5 Hz, 2F), −133.08 (dt, *J* = 53.5, 9.2 Hz, 2F); FTIR–ATR (neat) ν in cm^–1^: 3471, 3438, 1461, 1407, 1381, 1210, 1105, 1010, 994, 892, 801, 590, 493; HRMS (APCI^+^) for (M + H)^+^ C_10_H_7_ClF_4_NS^+^ (*m*/*z*): calcd 283.9918; found 283.9896.

#### 3-((1,1,2,2-Tetrafluoroethyl)thio)-1*H*-indole-5-carbonitrile (**29a**)

To a solution of 1*H*-indole-5-carbonitrile (43 mg, 0.3 mmol) in CH_2_Cl_2_ (1.5 mL) was added reagent **8a** (104 mg, 0.33 mmol) under vigorous stirring at 0 °C. The reaction mixture was gradually warmed to room temperature and stirred for 16 h. Then, EtOAc (50 mL) was added and the organic phase was washed with saturated aqueous Na_2_CO_3_ (4 × 10 mL). The combined organic fractions were dried with Na_2_SO_4_, filtered, and the solvent evaporated under reduced pressure to afford **29a** (76 mg, 92%) as a white solid. *R*_f_ (1:4 EtOAc/hexane): 0.10; m.p: 130–131 °C; ^1^H NMR (CDCl_3_, 400 MHz): δ 9.44 (bs, 1H), 8.15 (s, 1H), 7.70 (bs, 1H), 7.57 (d, *J* = 8.2, 1H), 7.52 (d, *J* = 8.2 Hz, 1H), 5.79 (bt, *J* = 53.7, 1H); ^13^C{^1^H} NMR (CDCl_3_, 100.6 MHz): δ 138.2, 135.7, 130.1, 126.2, 125.3, 122.1 (tt, *J* = 284.6, 29.6 Hz), 120.4, 113.2, 109.5 (tt, *J* = 253.0, 37.8 Hz), 104.6, 95.5 (t, *J* = 4.0 Hz); ^19^F NMR (CDCl_3_, 376.5 MHz): δ −92.56 (bt, *J* = 8.3 Hz, 2F), −132.55 (dt, *J* = 53.8, 8.3 Hz, 2F); FTIR–ATR (neat) ν in cm^–1^: 3280, 2227, 1618, 1471, 1418, 1381, 1341, 1242, 1213, 1107, 1011, 995, 811, 675, 637; HRMS (APCI^+^) for (M + H)^+^ C_11_H_6_F_4_N_2_S^+^ (*m*/*z*): calcd 275.0261; found 275.0281.

#### 5-Nitro-3-((1,1,2,2-tetrafluoroethyl)thio)-1*H*-indole (**30a**)

To a solution of 5-nitro-1*H*-indole (49 mg, 0.3 mmol) in CH_2_Cl_2_ (1.5 mL) was added reagent **8a** (104 mg, 0.33 mmol) under vigorous stirring at 0 °C. The reaction mixture was gradually warmed to room temperature and stirred for 24 h. Then, EtOAc (50 mL) was added and the organic phase was washed with saturated aqueous Na_2_CO_3_ (4 × 10 mL). The combined organic fractions were dried with Na_2_SO_4_, filtered, and the solvent evaporated under reduced pressure to afford **30a** (75 mg, 85%) as a yellow solid. *R*_f_ (4:6 EtOAc/hexane): 0.29; m.p: 147–149 °C; ^1^H NMR (CD_3_CN, 400 MHz): δ 10.37 (bs, 1H), 8.50 (d, *J* = 2.1, 1H), 8.07 (bs, *J* = 9.0, 2.3 Hz, 1H), 7.80 (s, 1H), 7.59 (dd, *J* = 9.0, 0.5 Hz, 1H), 6.09 (tt, *J* = 53.0, 3.9, 1H); ^13^C{^1^H} NMR (CDCl_3_, 100.6 MHz): δ 143.9, 140.5, 139.0, 130.4, 123.2 (tt, *J* = 283.1, 28.7 Hz), 119.1, 116.4, 113.9, 110.6 (tt, *J* = 250.7, 36.3 Hz), 95.8 (t, *J* = 3.9 Hz); ^19^F NMR (CDCl_3_, 376.5 MHz): δ −94.50 (td, *J* = 9.5, 3.9, Hz, 2F), −134.80 (dt, *J* = 53.6, 9.3 Hz, 2F); FTIR–ATR (neat) ν in cm^–1^: 3316, 1518, 1325, 1304, 1213, 1078, 992, 835, 816, 738; HRMS (APCI^+^) for (M + H)^+^ C_11_H_6_F_4_N_2_S^+^ (*m*/*z*): calcd 295.0159; found 295.0186.

#### 1,1,2,2-Tetrafluoro-*N*-methyl-*N*-(3-phenyl-3-(4-(trifluoromethyl)phenoxy)pro-pyl)ethane-1-sulfonamide (Fluoxetine-SO_2_CF_2_CF_2_H, **31a**)

A 5 mL round-bottom flask, equipped with a magnetic stir bar, was charged with **21a** (132 mg, 0.3 mmol) and ammonium molybdate tetrahydrate (37 mg, 0.03 mmol). Next, CH_3_OH (2 mL) was added using a syringe followed by H_2_O_2_ (30% w/w in H_2_O, 340 μL, 3 mmol). The mixture was stirred at room temperature for 16 h. Lastly, the reaction mixture was concentrated under reduced pressure and purified by flash column chromatography (1:4 EtOAc/hexane) to afford **31a** as a colorless syrup (134 mg, 56%). *R*_f_ (1:4 EtOAc/hexane): 0.33; ^1^H NMR (CDCl_3_, 400 MHz): δ 7.44 (d, *J* = 9.0 Hz, 2H), 7.39–7.27 (m, 4H), 6.89 (d, *J* = 8.6 Hz, 2H), 6.12 (tt, *J* = 52.4, 5.7 Hz, 1H), 5.24 (dd, *J* = 9.0, 3.7 Hz, 1H), 4.09–3-30 (bs, 2H), 3.08 (s, 3H), 2.43–2.08 (m, 2H); ^13^C{^1^H} NMR (CDCl_3_, 100.6 MHz): 160.1, 140.1, 129.2, 128.4, 127.0 (q, *J* = 3.7 Hz), 125.8, 124.4 (q, *J* = 271.5 Hz), 123.3 (q, *J* = 32.8 Hz), 115.9, 115.9 (tt, *J* = 291.6, 26.5 Hz), 107.9 (tt, *J* = 255.7, 30.3 Hz), 77.4, 48.3, 37.3, 35.9; ^19^F NMR (CDCl_3_, 376.5 MHz): δ −61.66 (s, 1F), −119.33 (m, 2F), −135.62 (dtd, *J* = 52.4, 7.9, 2.1 Hz, 2F); FTIR–ATR (neat) ν in cm^–1^: 1615, 1517, 1373, 1326, 1247, 1177, 1109, 1067, 837, 702, 584; HRMS (APCI^–^) for (M)^−·^ C_19_H_18_F_7_NO_3_S^–·^ (*m*/*z*): calcd 473.0896; found 473.0886.

#### 1,1,2,2,2-Pentafluoro-*N*-methyl-*N*-(3-phenyl-3-(4-(trifluoromethyl)phenoxy)pro-pyl)ethane-1-sulfonamide (Fluoxetine-SO_2_CF_2_CF_3_, **31b**)

A 5 mL round-bottom flask, equipped with a magnetic stir bar, was charged with **21b** (138 mg, 0.3 mmol) and ammonium molybdate tetrahydrate (37 mg, 0.03 mmol). Next, CH_3_OH (2 mL) was added using a syringe followed by H_2_O_2_ (30% w/w in H_2_O, 340 μL, 3 mmol). The mixture was stirred at 65 °C with an aluminum heating block for 16 h. As the mixture contained still starting material (observed by TLC), H_2_O_2_ (30% w/w in H_2_O, 340 μL, 3 mmol) and ammonium molybdate tetrahydrate (37 mg, 0.03 mmol) were added again, letting the mixture stirred at 65 °C with an aluminum heating block for 16 h more. Lastly, the reaction mixture was concentrated under reduced pressure and purified by flash column chromatography (1:9 EtOAc/hexane) to afford **31b** as a colorless syrup (185 mg, 75%). *R*_f_ (1:4 EtOAc/hexane): 0.43; ^1^H NMR (CDCl_3_, 400 MHz): δ 7.44 (d, *J* = 8.6 Hz, 2H), 7.39–7.27 (m, 4H), 6.89 (d, *J* = 8.6 Hz, 2H), 5.24 (dd, *J* = 9.0, 3.7 Hz, 1H), 3.87 (bs, 1H), 3.42 (bs, 1H), 3.09 (s, 3H), 2.43–2.06 (m, 2H); ^13^C{^1^H} NMR (CDCl_3_, 100.6 MHz): 160.1, 140.0, 129.2, 128.5, 127.0 (q, *J* = 3.7 Hz), 125.8, 123.4 (q, *J* = 32.7 Hz), 124.5 (q, *J* = 270.2 Hz), 117.5 (tt, *J* = 287.6, 31.8 Hz), 115.9, 113.6 (tq, *J* = 295.0, 40.2 Hz), 77.4, 48.5, 37.3, 36.0; ^19^F NMR (CDCl_3_, 376.5 MHz): δ −61.67 (s, 3F), −79.65 (bs, 3F), −115.92 (bs, 2F); FTIR–ATR (neat) ν in cm^–1^: 1615, 1518, 1389, 1325, 1222, 1162, 1111, 1068, 836, 702, 593; HRMS (APCI^–^) for (M–H)^−^ C_19_H_16_F_8_NO_3_S^–^ (*m*/*z*): calcd 490.0729; found 490.0723.

#### 3-((1,1,2,2-Tetrafluoroethyl)sulfonyl)-1*H*-indole (**32a**)

To a solution of indole **9a** (249 mg, 1 mmol) in CH_3_OH (5 mL) was added ammonium molybdate tetrahydrate (61 mg, 0.05 mmol). H_2_O_2_ (30% w/w in H_2_O, 306 μL, 3 mmol) was added and the reaction mixture stirred at room temperature for 16 h. A second batch of H_2_O_2_ (30% w/w in H_2_O, 500 μL, 4.9 mmol) was added and the reaction mixture was stirred at room temperature for 24 h. Water was added to the reaction mixture and the product was extracted successively with CH_2_Cl_2_ (3 × 20 mL). The combined organic fractions were dried with Na_2_SO_4_, filtered, and evaporated under reduced pressure to afford **32a** (267 mg, 95%) as an orange solid. *R*_f_ (1:4 EtOAc/hexane): 0.17; m.p: 92–93 °C; ^1^H NMR (CDCl_3_, 400 MHz): δ 9.56 (bs, 1H), 8.00–7.89 (m, 2H), 7.57–7.44 (m, 1H), 7.41–7.28 (m, 2H), 6.30 (tt, *J* = 52.3, 5.6 Hz, 1H); ^13^C{^1^H} NMR (CDCl_3_, 100.6 MHz): δ 136.4, 135.6, 125.0, 124.5, 124.0, 119.6, 114.6 (tt, *J* = 293.6, 26.6 Hz), 113.0, 108.1 (tt, *J* = 254.7, 28.8 Hz), 105.7; ^19^F NMR (CDCl_3_, 376.5 MHz): δ −120.91 (td, *J* = 8.2, 5.6 Hz, 2F), −134.56 (dt, *J* = 52.2, 8.2 Hz, 2F); FTIR–ATR (neat) ν in cm^–1^: 3358, 1148, 1111, 741, 670, 608, 583, 550, 532, 487, 419; HRMS (APCI^+^) for (M + H)^+^ C_10_H_8_F_4_NO_2_S^+^ (*m*/*z*): calcd 282.0206; found 282.0202.

#### *tert*-Butyl 3-((1,1,2,2-Ttetrafluoroethyl)thio)-1*H*-indole-1-carboxylate (**33a**)

To a solution of indole **9a** (498 mg, 2 mmol) in CH_2_Cl_2_ (10 mL) was added Et_3_N (557 μL, 4 mmol) and 4–(dimethylamino)pyridine (12 mg, 0.1 mmol). At room temperature, di-*tert*-butyl dicarbonate (523 mg, 2.4 mmol) was added and the reaction mixture was stirred at the same temperature for 16 h. Water was then added and the product was extracted with CH_2_Cl_2_ (3 × 20 mL). The combined organic fractions were dried with Na_2_SO_4_, filtered, and evaporated under reduced pressure. The residue was purified by flash column chromatography (1:9 EtOAc/hexane) to afford **33a** (680 mg, 97%) as a white solid. *R*_f_ (1:4 EtOAc/hexane): 0.51; m.p: 71 °C; ^1^H NMR (CDCl_3_, 400 MHz): δ 8.19 (d, *J* = 8.0 Hz, 1H), 7.94 (s, 1H), 7.75 (d, *J* = 7.5 Hz, 1H), 7.44–7.34 (m, 2H), 5.78 (tt, *J* = 53.7, 3.6 Hz, 1H), 1.70 (s, 9H); ^13^C{^1^H} NMR (CDCl_3_, 100.6 MHz): δ 148.8, 135.5, 134.5, 131.6, 125.5, 123.8, 122.2 (tt, *J* = 286.0, 29.1 Hz), 119.7, 115.5, 109.4 (tt, *J* = 252.9, 37.1 Hz), 100.5 (t, *J* = 3.7 Hz), 85.2, 28.1; ^19^F NMR (CDCl_3_, 376.5 MHz): δ −92.35 (td, *J* = 9.0, 3.3 Hz, 2F), −132.91 (dt, *J* = 18.5, 8.8 Hz, 2F); FTIR–ATR (neat) ν in cm^–1^: 1742, 1449, 1371, 1356, 1252, 1224, 1153, 1115, 1064, 747; HRMS (APCI^+^) for (M + H)^+^ C_15_H_16_F_4_NO_2_S^+^ (*m*/*z*): calcd 350.0832; found 350.0823.

#### 3-((1*H*-Indole-3-yl)thio)-2,2,3,3-tetrafluoro-1,1-diphenylpropan-1-ol (**34a**)

To a Schlenk tube charged with indole **33a** (175 mg, 0.5 mmol) and benzophenone (182 mg, 1 mmol), was added anhydrous DMF (5 mL) under argon. The reaction mixture was cooled to −40 °C and KHMDS (1 M, 1 mL) was added and the mixture stirred at the same temperature for 15 min. Et_2_O (30 mL) and water (2 mL) were added at −40 °C under stirring and the organic phase was washed with saturated aqueous NH_4_Cl (3 × 10 mL), dried with Na_2_SO_4_, filtered and the solvent evaporated. The residue was diluted with trifluoroacetic acid (5 mL) and stirred at room temperature for 3 h. The reaction crude was then concentrated under reduced pressure and the residue was diluted with Et_2_O (30 mL) and washed with saturated aqueous NaHCO_3_ (3 × 10 mL), dried with Na_2_SO_4_, filtered and the solvent evaporated. The residue was purified by flash column chromatography (1:9 EtOAc/hexane) to afford **34a** (151 mg, 76%) as a brown solid. *R*_f_ (1:4 EtOAc/hexane): 0.18; m.p: 112 °C; ^1^H NMR (CDCl_3_, 400 MHz): δ 8.36 (s, 1H), 7.78–7.70 (m, 1H), 7.70–7.63 (m, 4H), 7.42–7.31 (m, 8H), 7.30–7.22 (m, 2H), 3.07 (s, 1H); ^13^C{^1^H} NMR (CDCl_3_, 100.6 MHz): δ 140.6, 136.1, 133.2, 130.3, 128.3, 128.1, 127.6, 125.3 (tt, *J* = 291.2, 36.0), 123.2, 121.4, 119.7, 117.8 (tt, *J* = 267.3, 30.7), 111.6, 95.5, 79.9 (t, *J* = 24.1 Hz); ^19^F NMR (CDCl_3_, 376.5 MHz): δ −81.01 (t, *J* = 4.9 Hz, 2F), −109.64 (t, *J* = 4.7 Hz, 2F); FTIR–ATR (neat) ν in cm^–1^: 3534, 3419, 1448, 1092, 741, 698; HRMS (TOF ES^+^) for (M + Na)^+^ C_23_H_17_F_4_NNaOS^+^ (*m*/*z*): calcd 454.0859; found 454.0847.

#### 5-(4-Phenoxyphenyl)-3-((1,1,2,2-tetrafluoroethyl)thio)-1*H*-indole (**35a**)

To a reaction vial equipped with a magnetic stir bar was added **26a** (98 mg, 0.3 mmol), (4–phenoxyphenyl)boronic acid (71 mg, 0.33 mmol), Pd(PPh_3_)_4_ (35 mg, 0.03 mmol) and toluene (2 mL). Then, Na_2_CO_3_ (79 mg, 0.75 mmol), water (0.3 mL) and EtOH (0.6 mL) were successively added and the reaction mixture was sparged with argon for 5 min. The vial was then capped with a rubber septum and the reaction mixture stirred at 90 °C with an aluminum heating block for 24 h. After reaction completion, the reaction crude was concentrated under reduced pressure and the residue was purified by flash column chromatography (hexane to 1:4 EtOAc/hexane) to afford **35a** (113 mg, 90%) as a yellowish syrup. *R*_f_ (1:4 EtOAc/hexane): 0.27; ^1^H NMR (CDCl_3_, 400 MHz): δ 8.54 (s, 1H), 8.02 (s, 1H), 7.69–7.62 (m, 2H), 7.56–7.51 (m, 2H), 7.47 (d, *J* = 8.5 Hz, 1H), 7.42–7.36 (m, 2H), 7.18–7.08 (m, 5H), 5.79 (tt, *J* = 53.7, 3.9 Hz, 1H); ^13^C{^1^H} NMR (CDCl_3_, 100.6 MHz): δ 157.3, 156.5, 137.0, 135.4, 134.7, 133.8, 130.4, 129.8, 128.8, 123.3, 123.1, 122.2 (tt, *J* = 284.2, 28.6 Hz), 119.2, 118.9, 117.4, 112.0, 109.53 (tt, *J* = 252.8, 36.9 Hz), 94.7 (t, *J* = 3.9 Hz); ^19^F NMR (CDCl_3_, 376.5 MHz): δ −94.00 (td, *J* = 9.7, 4.0 Hz, 2F), −133.43 (dt, *J* = 53.6, 9.5 Hz, 2F); FTIR–ATR (neat) ν in cm^–1^: 3409, 1489, 1470, 1235, 1107, 993, 809; HRMS (APCI^+^) for (M + H)^+^ C_22_H_16_F_4_NOS^+^ (*m*/*z*): calcd 418.0883; found 418.0873.

## Supporting Information Available

Optimization, stability, and solvent compatibility studies, DSC and TGA data, copies of ^1^H, ^13^C, and ^19^F NMR spectra for all new compounds, single-crystal X-ray crystallographic data, electrostatic potential surface calculations, and conformational analysis (PDF)
