# Supplementary material for: Electrophilic Reagents for the Direct Incorporation of Uncommon SCF2CF2H and SCF2CF3 Motifs
Source: J Org Chem. 2022 Aug 9;87(16):10791–806. doi: 10.1021/acs.joc.2c01038 (PMC9400389; doi:10.1021/acs.joc.2c01038)
Supplement: Supplementary file 1 — jo2c01038_si_001.pdf [file jo2c01038_si_001.pdf]

Electrophilic reagents for the direct incorporation of uncommon SCF_2CF_2H and SCF_2CF_3 motifs

Jordi Mestre,‡ Miguel Bernús,‡ Sergio Castillón, and Omar Boutureira*

Departament de Química Analítica i Química Orgànica
Universitat Rovira i Virgili, C/ Marcel·lí Domingo 1, 43007 Tarragona, Spain

‡Equal contribution

**E-mail: omar.boutureira@urv.cat*

Table of Contents

1. Reagent Optimization S2
2. Stability of selected reagents **8a** and **8b** S3
    2.1. In solution S3
    2.2. Differential scanning calorimetry (DSC) S4
    2.3. Thermogravimetric analysis (TGA) S5
3. Solvent compatibility S6
4. Electrostatic potential surface calculation S7
5. Conformational analysis of **19a** and **19b** S10
6. NMR Spectra S11
7. X-ray crystallographic data S172
8. References S181

1. Reagent optimization

Aiming to identify the optimal reagent, we prepared different structures with the tetrafluoroethylsulfenyl motif. The performance of the reagents described previously was assayed by reaction with *N*-H indole. Different features of the electrophilic reagents **2a**–**8a** were compared including yield, purity, performance, stability, and the requirement of further purification (Table S1). Moreover, the commercial availability and cost of the scaffolds or methods for their synthesis were also considered. Succinimide **2a** and phthalimide **3a** were discarded since, among other disadvantages, were inefficient to transfer the fluorinated chain to *N*-H indole at room temperature and under catalyst-free conditions (entries 1 and 2). The presence of a sulfonamide motif in reagents **4a**–**8a** increased the reactivity and allowed the preparation of **9a** at room temperature under catalyst-free conditions (entries 3–7). Reagents **4a** and **5a** were obtained in low to moderate yields along with impurities, which could not be purified by other techniques due to decomposition issues. Finally, bis-sulfonimide reagents **6a** and **7a** were also tested and they showed outstanding reactivity, but **6a** also suffered from decomposition upon storage in the freezer (–20 ºC). Additionally, preparation of **6a** failed using the potassium amide and only worked employing the corresponding silver salt. Finally, it was concluded after several optimization experiments that **7a** was incompatible with different reaction conditions (strong-bases, Lewis acids, and certain catalysts) preventing general application of such reagent. Thus, **8a** resulted the reagent of choice for transferring the target polyfluorinated ethyl sulfides.

Table S1. GAR (green-amber-red) analysis of electrophilic reagents **2a**–**8a**

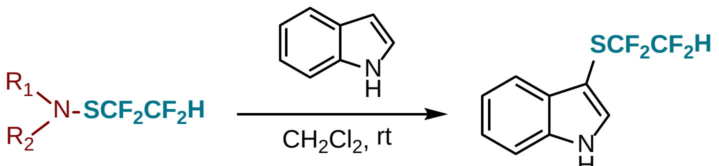

Electrophilic reagents (**2a** **8a**)

9a

entry	reagent	stability	reactivity	solid	ease of purification[a]	yield	commercial availability/cost	reaction compatibility
1	**2a**	green	red	green	red	green	green	ND
2	**3a**	green	red	green	red	amber	green	ND
3	**4a**	green	green	green	red	amber	green	ND
4	**5a**	red	green	green	red	red	red	ND
5	**6a**	amber	green	green	green	green	amber	ND
6	**7a**	green	green	green	green	green	amber	red
7	**8a**	green	green	green	green	green	green	green

[a]Purification by chromatography caused decomposition for **2a**–**5a**. ND = Not determined

2. Stability of selected reagents 8a and 8b

2.1. In solution

In view of future applications, the stability *in solution* of selected reagent **8a** was tested by ^{19}F NMR. During the course of our studies no decomposition was observed upon storage in the freezer (–20 ºC). However, we decided to determine the stability in different solvents at room temperature and at 50 ºC (Figure S1). Reagent **8a** showed high stability in dichloroethane and toluene and no decomposition was observed after 1 week both at room temperature and after heating up to 50 ºC. While only a slightly decomposition was observed in MeCN (named as ACN in Figure S1), stability of **8a** in THF was more compromised and the degradation was further accelerated upon heating, reaching complete decomposition after 91 h. The presence of DMF lowered the stability under both room temperature and heating conditions and the presence of water in a 4:1 MeCN/H_2O mixture caused a fast decomposition rate.

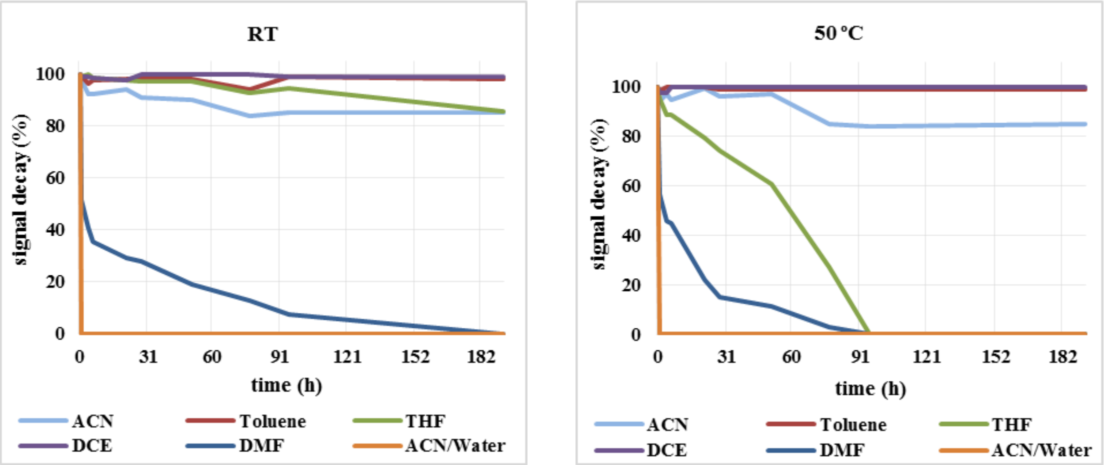

Figure S1. Stability of **8a** in various solvents at room temperature (left panel) and at 50 ºC (right panel)

2.2. Differential scanning calorimetry (DSC)

Calorimetric studies (DSC) were carried out on a Mettler DSC3+ Star system thermal analyzer under N_2 as a purge gas (50 ml/min) using heating rates of 2 °C/min and cooling rates of 30 °C/min in a 25 ºC to 350 °C temperature range. Calibration was made using an indium standard (heat flow calibration) and an indium-lead-zinc standard (temperature calibration). Samples of 10 mg were sealed in aluminum perforated pans.

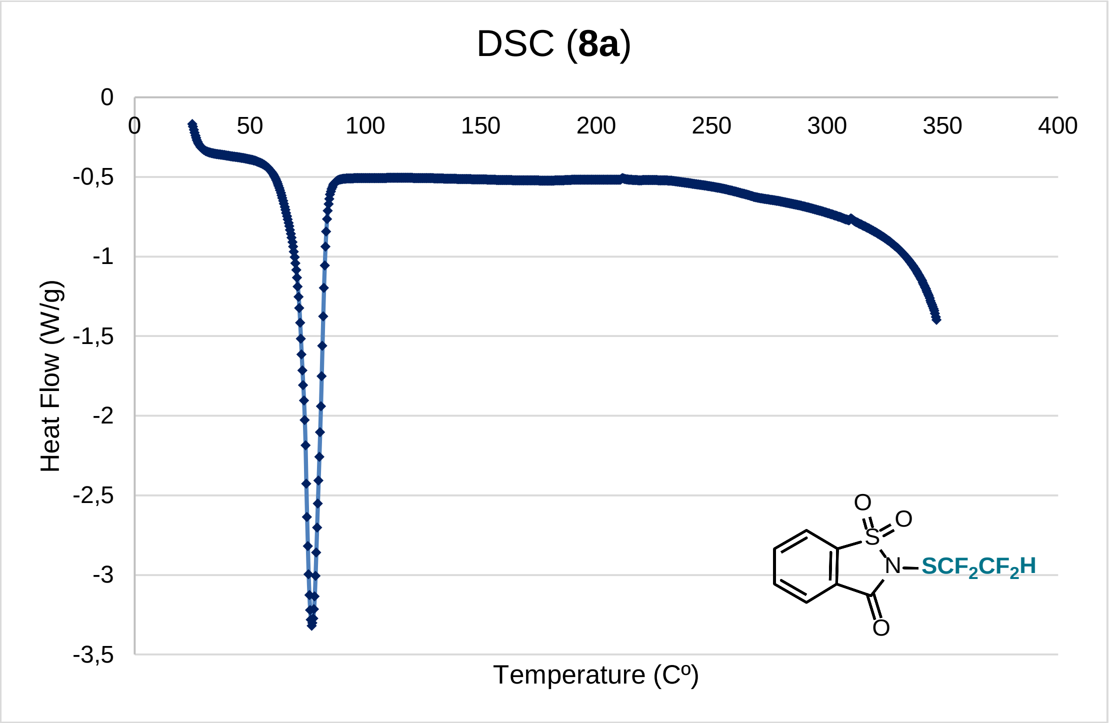

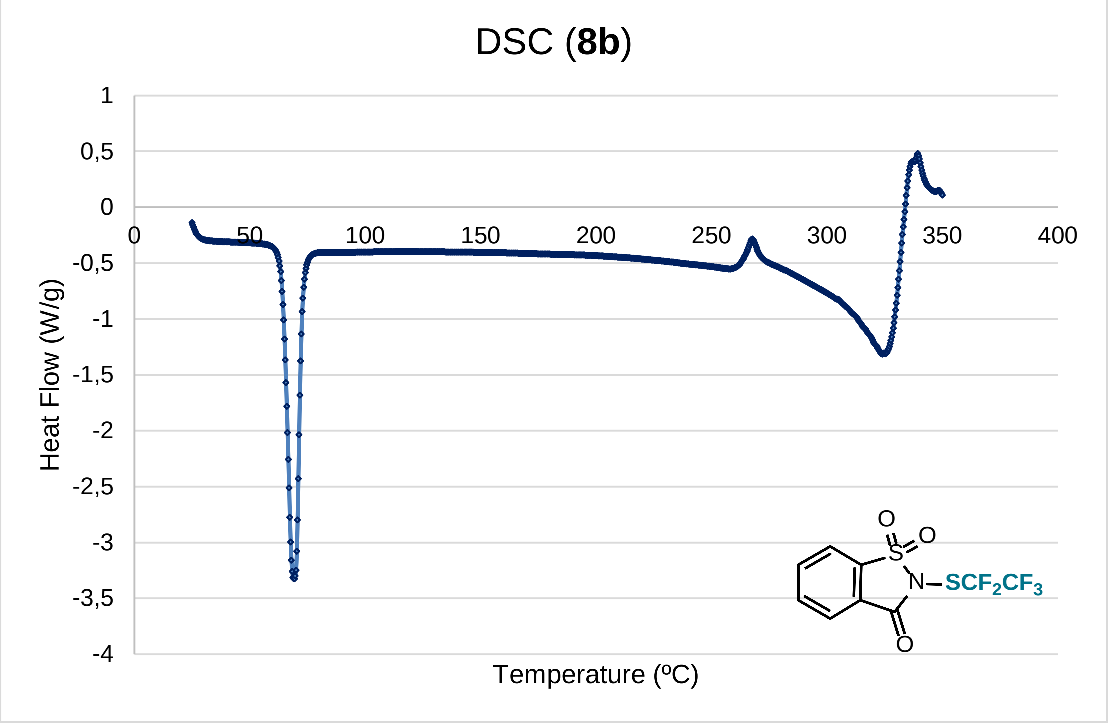

Figure S2. DSC analysis of reagents **8a** and **8b**

2.3. Thermogravimetric analysis (TGA)

Thermogravimetric analysis (TGA) was carried out on a Mettler TGA/SDTA851e/LF/1100 with N_2 as the purge gas (50 ml/min) at a scanning rate of 10 °C/min in a 30 ºC to 400 °C temperature range. Samples of 10–20 mg were analyzed in ceramic crucibles.

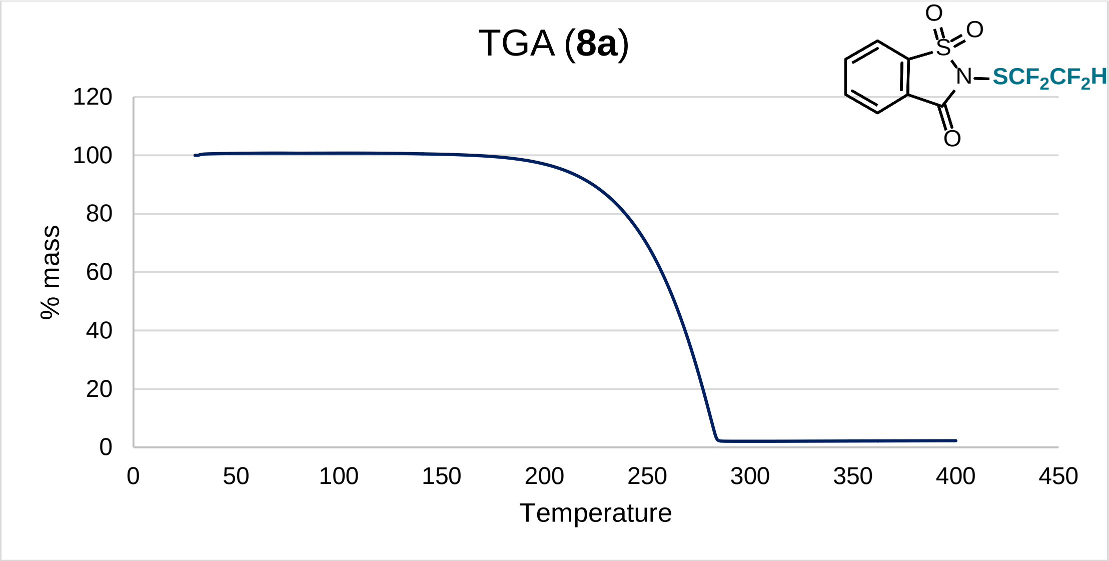

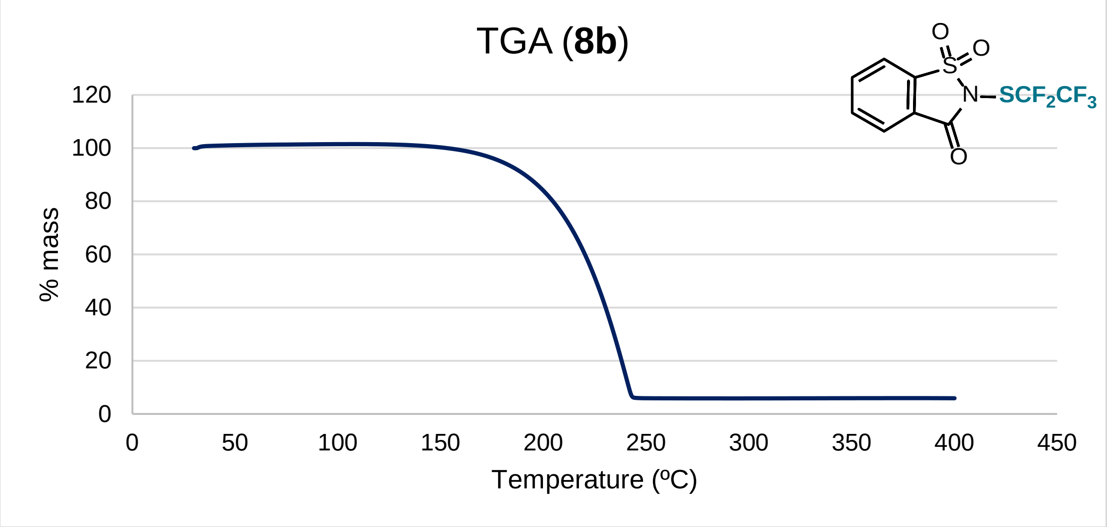

Figure S3. TGA analysis of reagents **8a** and **8b**. 5% weight decomposition (**8a**) = 209 ºC; 5% weight decomposition (**8b**) = 179 ºC

3. Solvent compatibility

The utility of electrophilic reagent **8a** was tested in different solvents to study the tolerance of this system. The results summarized in Figure S4 demonstrate the robustness of the reaction, where solvents of different nature (chlorinated, protic, aprotic polar, and aprotic non-polar solvents) do not substantially affect the performance of the reaction. In the case of CH_3OH and DMSO, unidentified products were detected by ^{19}F NMR, suggesting that these solvents promote the formation of by-products.

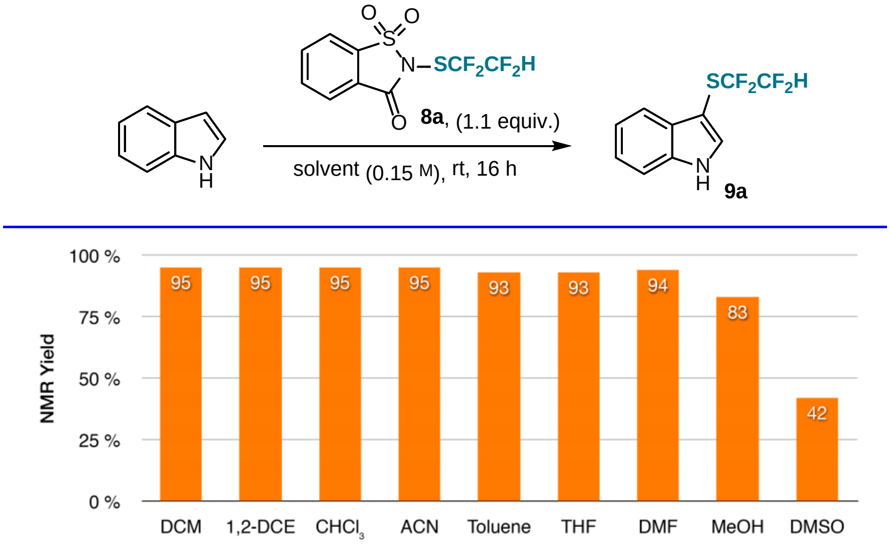

Figure S4. Reaction of electrophilic reagent **8a** with *N*-H-indole in various solvents

4. Electrostatic potential surface calculation

DFT calculations were performed using Gaussian 09.[1] Geometry optimization was conducted at the CPCM (water) B3LYP/6-311+G(d,p) level of theory. Frequencies were calculated at the same level of theory and used to verify the nature of all stationary points as minima.

Phenyl(1,1,2,2-tetrafluoroethyl)sulfane (S3)

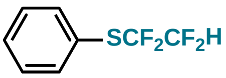

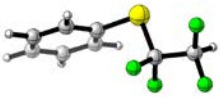

Center Number	Atomic Number	Atomic Type	Coordinates (Angstroms) X	Y	Z
1	16	0	0.270529	-0.451616	1.186595
2	9	0	0.939909	1.468673	-0.527868
3	9	0	1.166667	-0.550747	-1.317327
4	9	0	3.199850	-1.020563	0.558765
5	9	0	3.521647	0.647353	-0.840184
6	6	0	1.297652	0.195303	-0.178764
7	6	0	2.784923	0.235881	0.229956
8	1	0	2.962694	0.908986	1.067787
9	6	0	-1.368939	-0.203224	0.485409
10	6	0	-2.058292	0.986061	0.738407
11	6	0	-1.966732	-1.229001	-0.252166
12	6	0	-3.347667	1.151577	0.235387
13	1	0	-1.592279	1.770143	1.321728
14	6	0	-3.256527	-1.053913	-0.751005
15	1	0	-1.430148	-2.152282	-0.430768
16	6	0	-3.945517	0.134652	-0.509280
17	1	0	-3.883796	2.073461	0.428215
18	1	0	-3.721620	-1.847167	-1.324450
19	1	0	-4.949378	0.266268	-0.896177

Zero-point correction= 0.125632 (Hartree/Particle)

Thermal correction to Energy= 0.137542

Thermal correction to Enthalpy= 0.138486

Thermal correction to Gibbs Free Energy= 0.085010

Sum of electronic and zero-point Energies= -1106.126606

Sum of electronic and thermal Energies= -1106.114696

Sum of electronic and thermal Enthalpies= -1106.113752

Sum of electronic and thermal Free Energies= -1106.167228

Electrostatic potential surface:

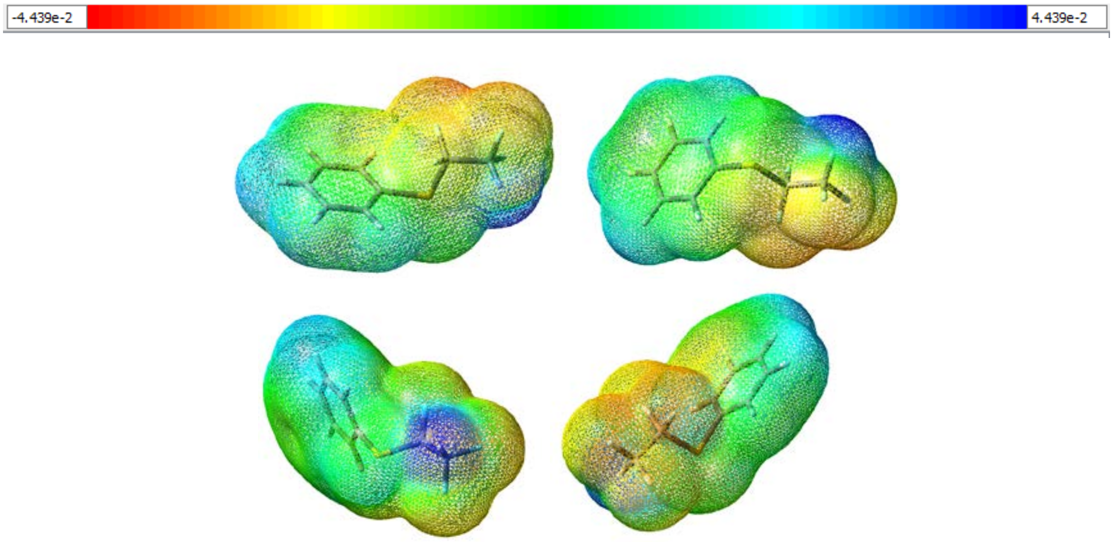

Figure S5. Different views of the electrostatic potential surface of phenyl(1,1,2,2-tetrafluoroethyl)sulfane **S3**

(Perfluoroethyl)(phenyl)sulfane (S4)

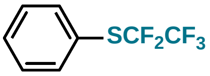

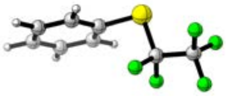

Center Number	Atomic Number	Atomic Type	Coordinates (Angstroms) X	Y	Z
1	9	0	0.763942	0.915436	1.210856
2	9	0	0.852206	-1.246365	0.961278
3	16	0	0.082234	0.071077	-1.220375
4	9	0	2.969846	-0.928297	-0.835888
5	9	0	3.285591	-0.114823	1.154416
6	9	0	2.896828	1.222041	-0.512784
7	6	0	1.063781	-0.064715	0.313030
8	6	0	2.589326	0.031453	0.018896

9	6	0	-1.574005	0.033846	-0.515435
10	6	0	-2.222853	-1.190617	-0.332601
11	6	0	-2.222881	1.236248	-0.221322
12	6	0	-3.524569	-1.207668	0.165173
13	1	0	-1.717644	-2.115986	-0.578777
14	6	0	-3.525578	1.208064	0.273273
15	1	0	-1.716000	2.179799	-0.379291
16	6	0	-4.174561	-0.011191	0.468549
17	1	0	-4.029952	-2.155119	0.310914
18	1	0	-4.031520	2.138347	0.503309
19	1	0	-5.188297	-0.028709	0.851556

Zero-point correction= 0.117020 (Hartree/Particle)

Thermal correction to Energy= 0.129589

Thermal correction to Enthalpy= 0.130533

Thermal correction to Gibbs Free Energy= 0.075401

Sum of electronic and zero-point Energies= -1205.409126

Sum of electronic and thermal Energies= -1205.396557

Sum of electronic and thermal Enthalpies= -1205.395613

Sum of electronic and thermal Free Energies= -1205.450746

Electrostatic potential surface:

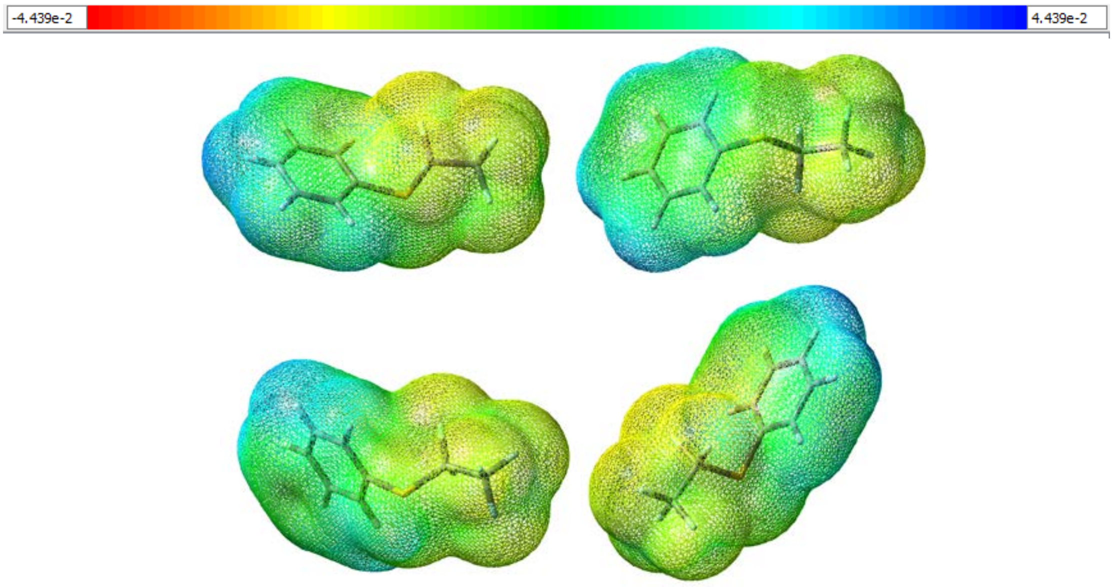

Figure S6. Different views of the electrostatic potential surface of (perfluoroethyl)(phenyl)sulfane **S4**

5. Conformational analysis of 19a and 19b

The incorporation of SC_2F_2H and SC_2F_5 motifs had less impact than their alkyl derivatives C_2F_5 and CF_3 on the conformation of 2-substituted-D-glucals despite their larger Van der Waals volume (81.7 Å^3 for SCF_2CF_2H and 87.7 Å^3 for SCF_2CF_3) as evidenced by the medium $J_{3,4}$ and $J_{4,5}$ values (4–6 Hz). Both coupling constant values decrease while steric hindrance at C-2 increases, probably indicating a progressive ring flattering from the $^4H_5$ conformation (for H) to intermediate/transition $^4H_5 \rightarrow {}^5H_4$ conformations deformed to $^5H_4$ (for I < SC_2F_5 < SC_2F_2H) to a complete ring inversion to the $^5H_4$ conformation (for CF_3 < CF_2CF_3) due to 1,2-allylic ($A^{1,2}$) strain. The presence of a grafted S atom between the sugar ring and the bulky R_F groups relieves such destabilizing interactions, as these groups point away from the ring (Figure S7).[1]

X	Conformation	$J_{3,4}$ (Hz)	$J_{4,5}$ (Hz)	[Ref]
**H**	$^4H_5$	6.2	8.2	3
**I**	intermediate	5.0	6.6	5
**SC_2F_5**	intermediate	4.6	5.8	this work
**SC_2F_4H**	intermediate	4.0	5.0	this work
**CF_3**	$^5H_4$	3.2	3.2	4
**CF_2CF_3**	$^5H_4$	<2	<2	6

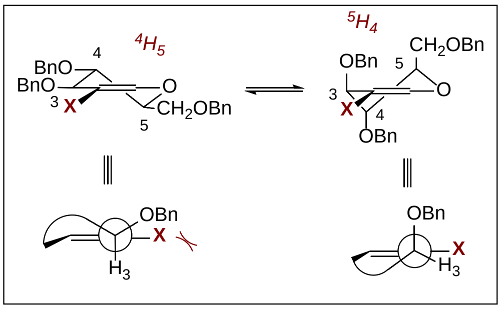

Figure S7. Conformational analysis of 2-substituted-3,4,6-tri-*O*-benzyl-D-glucals[1–4]

6. NMR Spectra

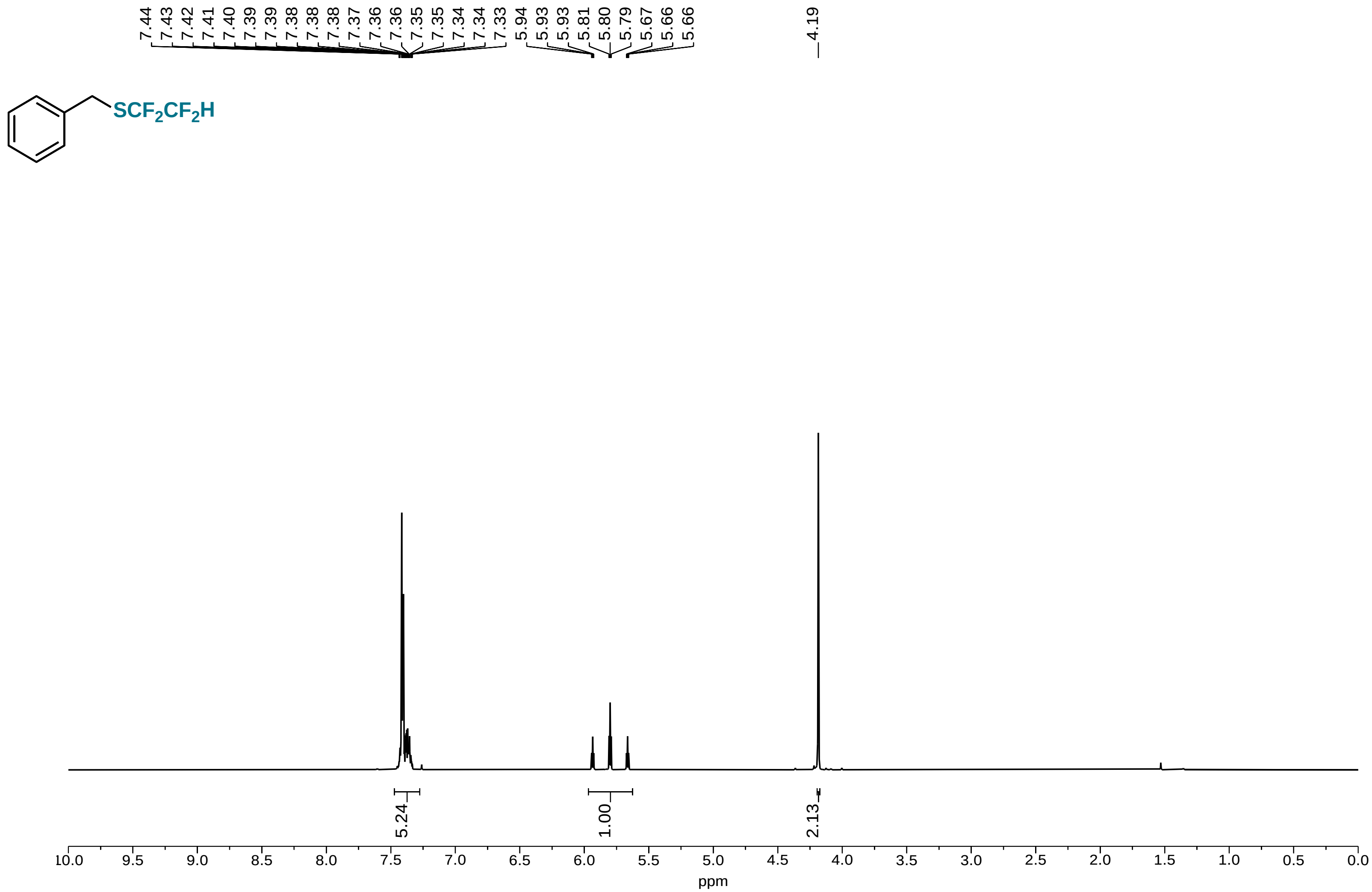

Figure S8. ^{1}H NMR ($CDCl_3$, 400 MHz) of **1a**

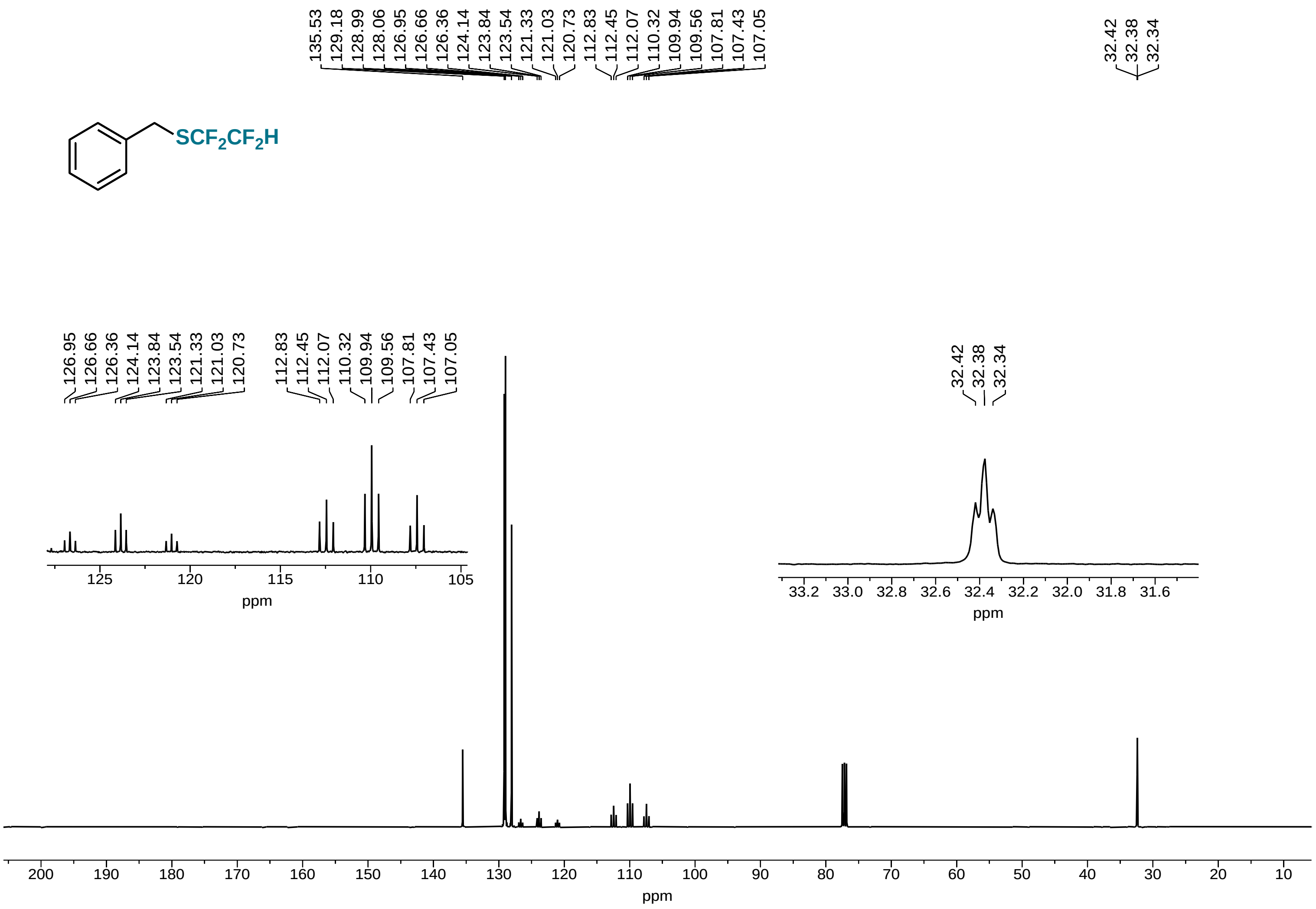

Figure S9. $^{13}C\{^{1}H\}$ NMR ($CDCl_3$, 100.6 MHz) of **1a**

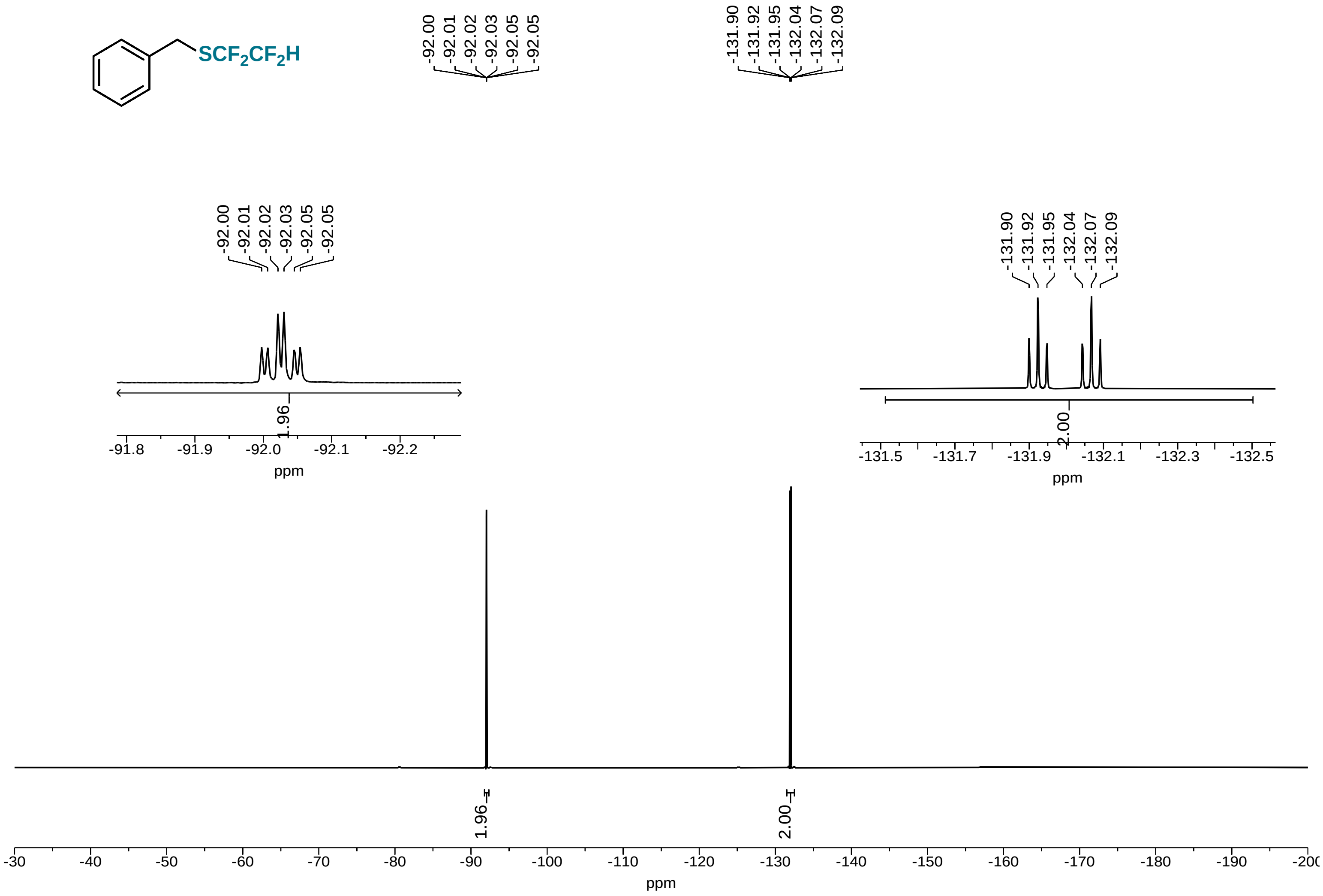

Figure S10. ^{19}F NMR ($CDCl_3$, 376.5 MHz) of **1a**

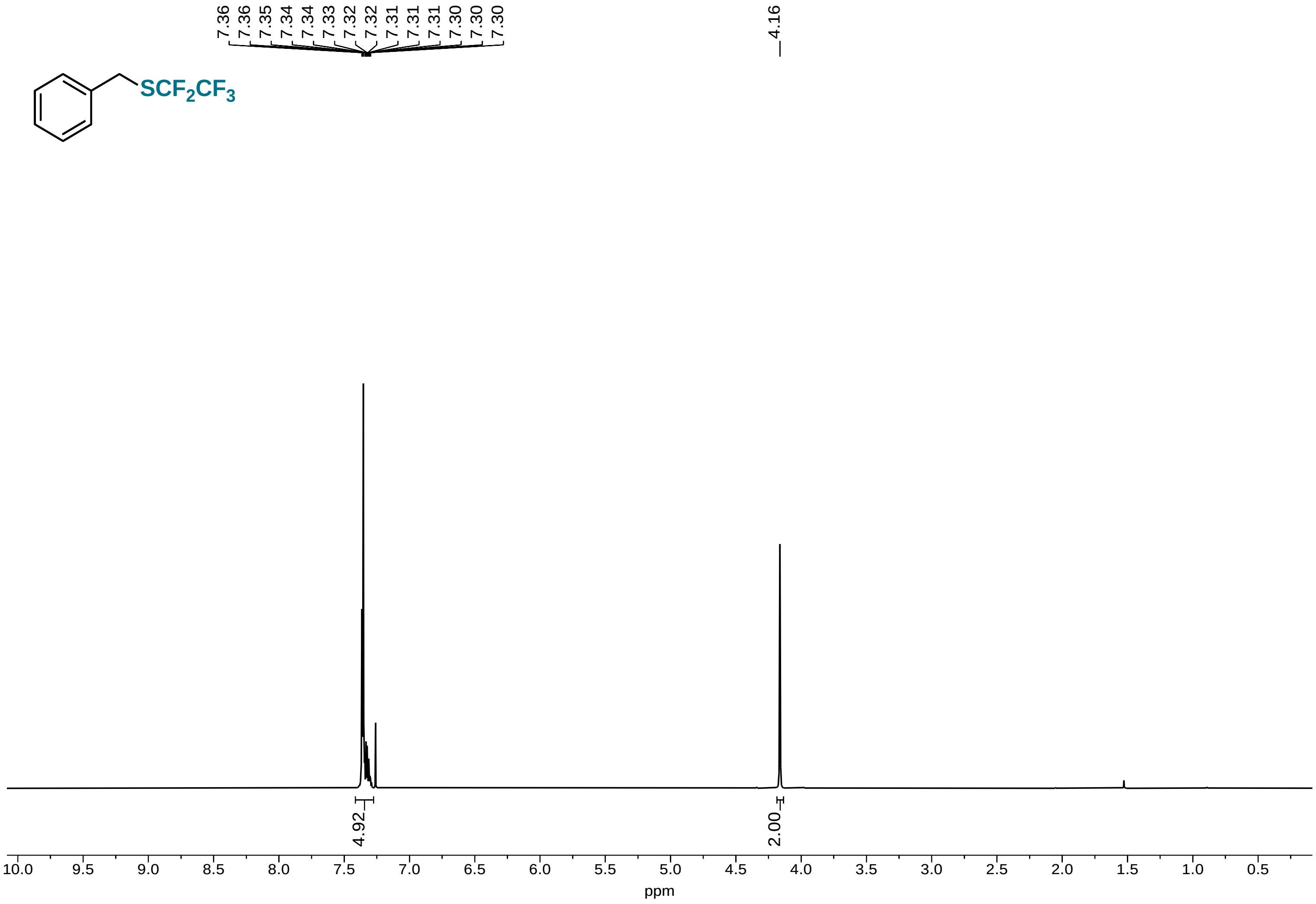

Figure S11. ^{1}H NMR ($CDCl_3$, 400 MHz) of **1b**

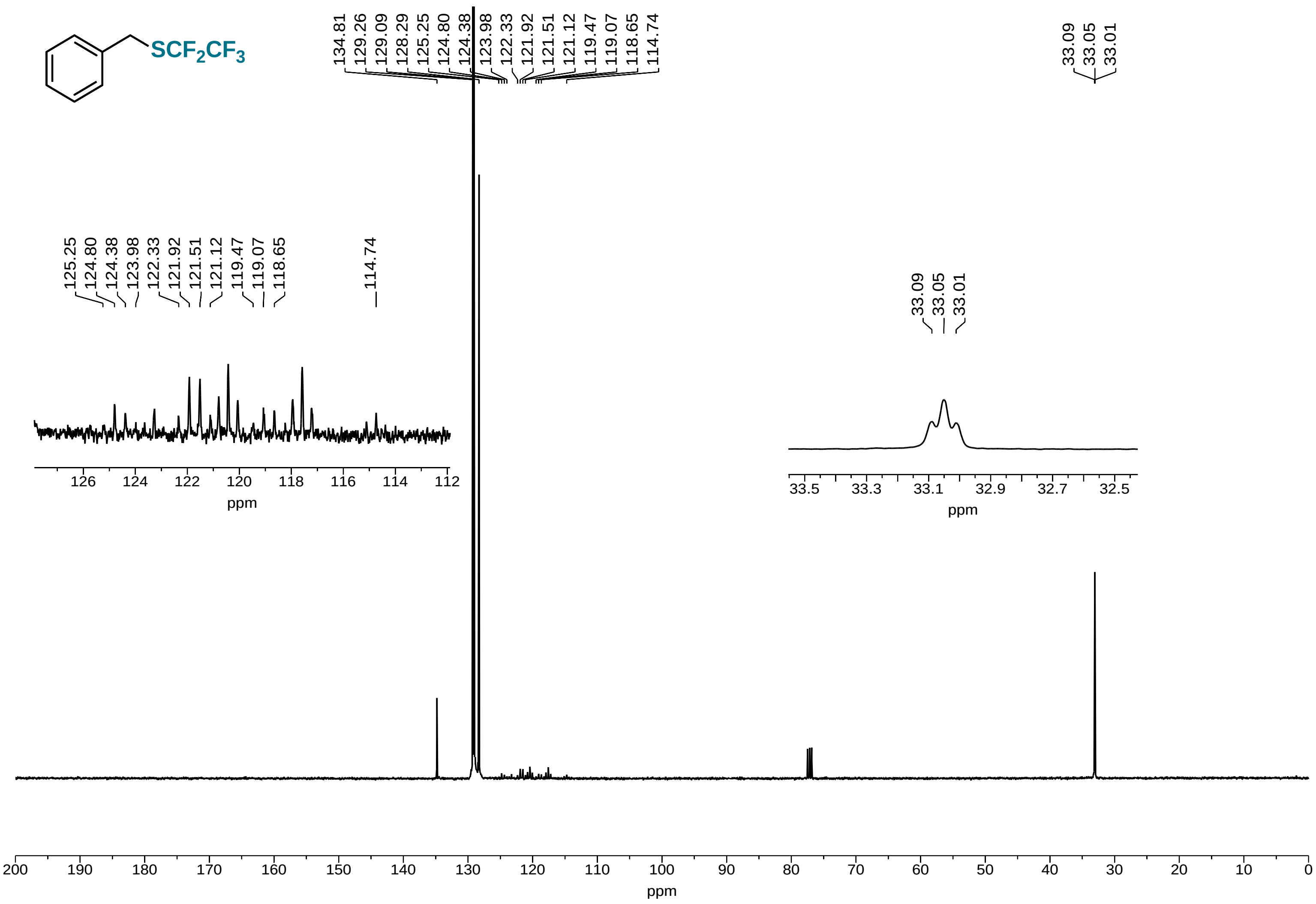

Figure S12. $^{13}C\{^1H\}$ NMR ($CDCl_3$, 100.6 MHz) of **1b**

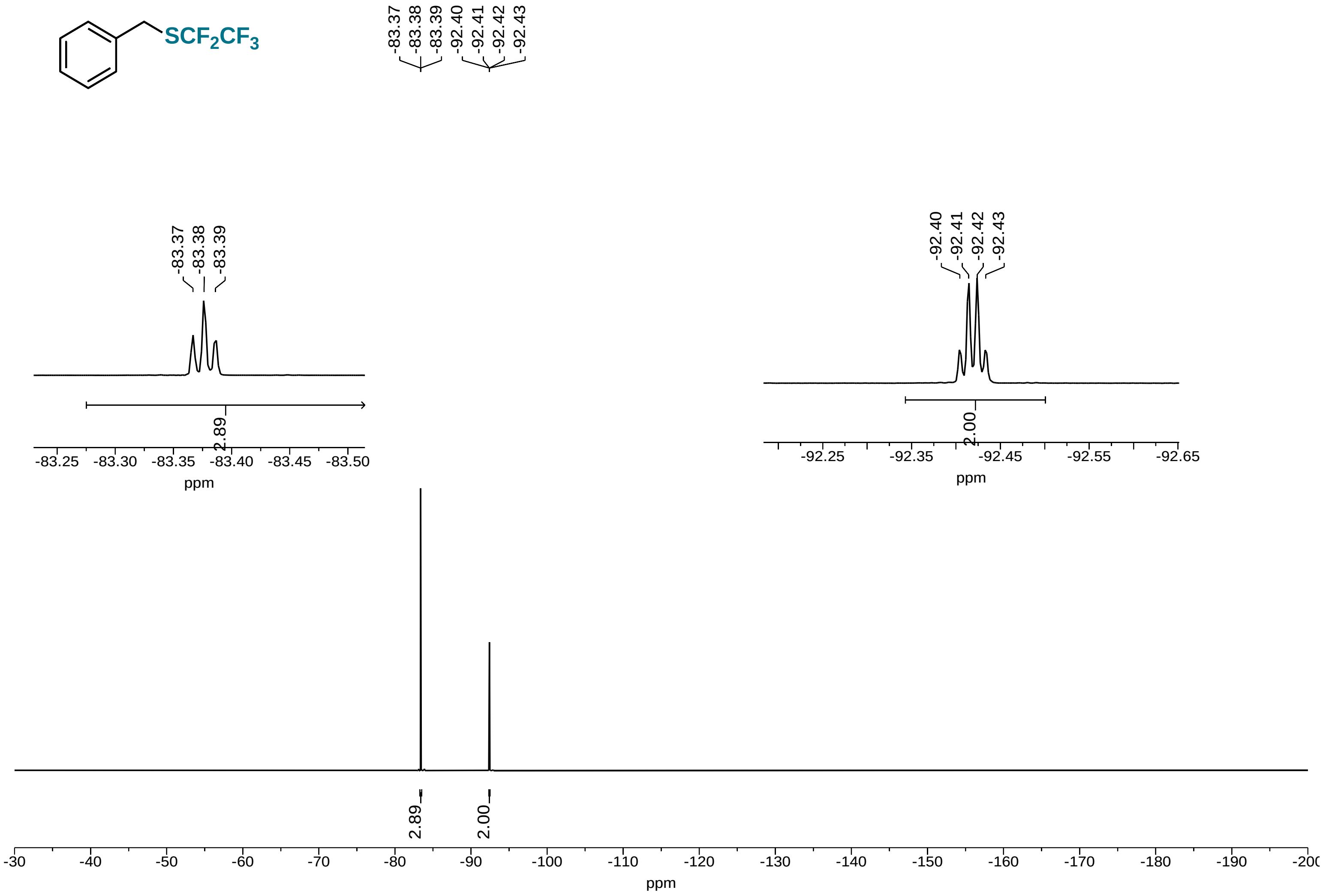

Figure S13. ^{19}F NMR ($CDCl_3$, 376.5 MHz) of **1b**

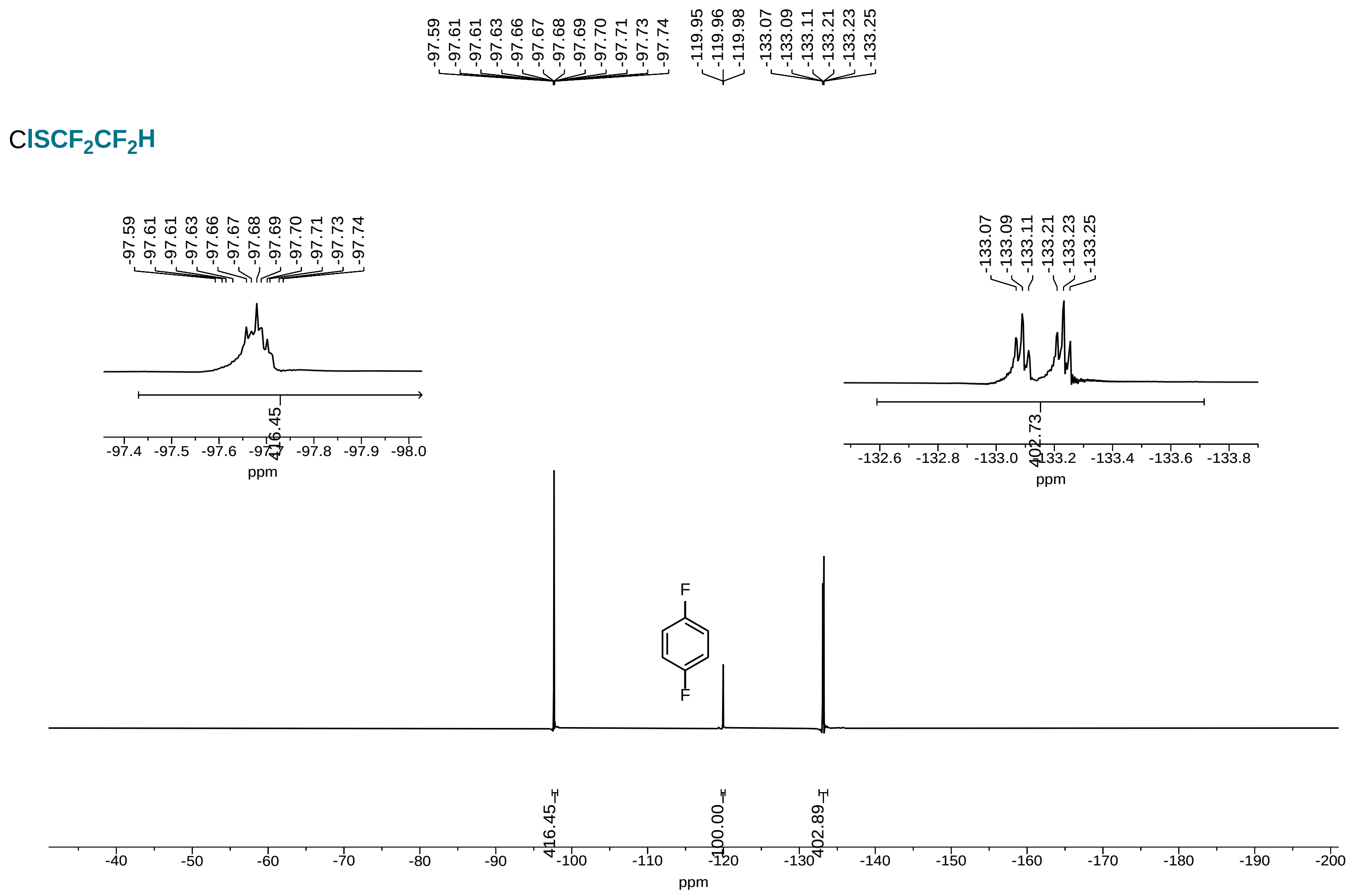

Figure S14. ^{19}F NMR ($CHCl_3$, 376.5 MHz) of 1,1,2,2-tetrafluoroethyl hypochlorothioite **S1**

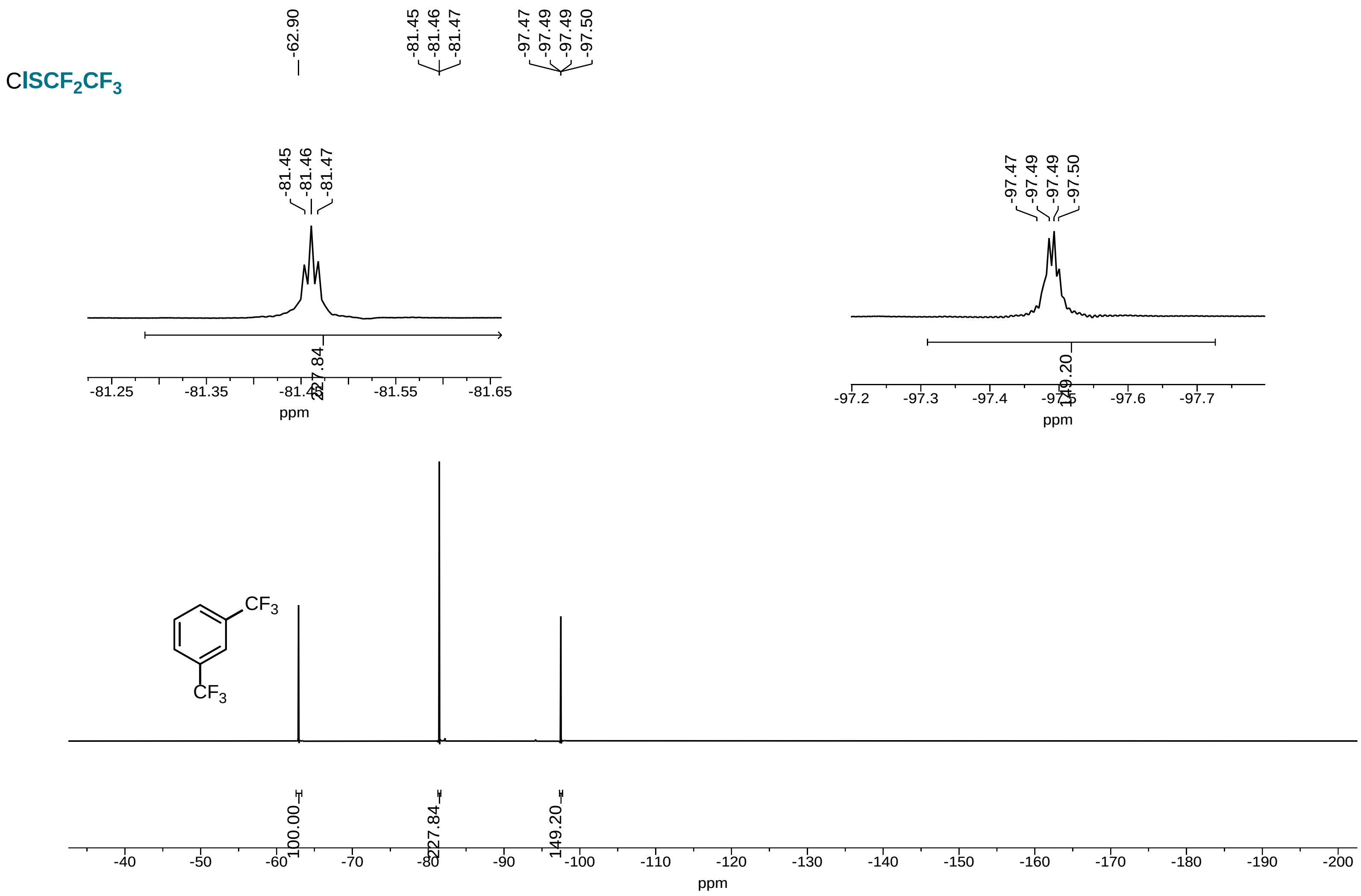

Figure S15. ^{19}F NMR (CH_2Cl_2, 376.5 MHz) of perfluoroethyl hypochlorothioite **S2**

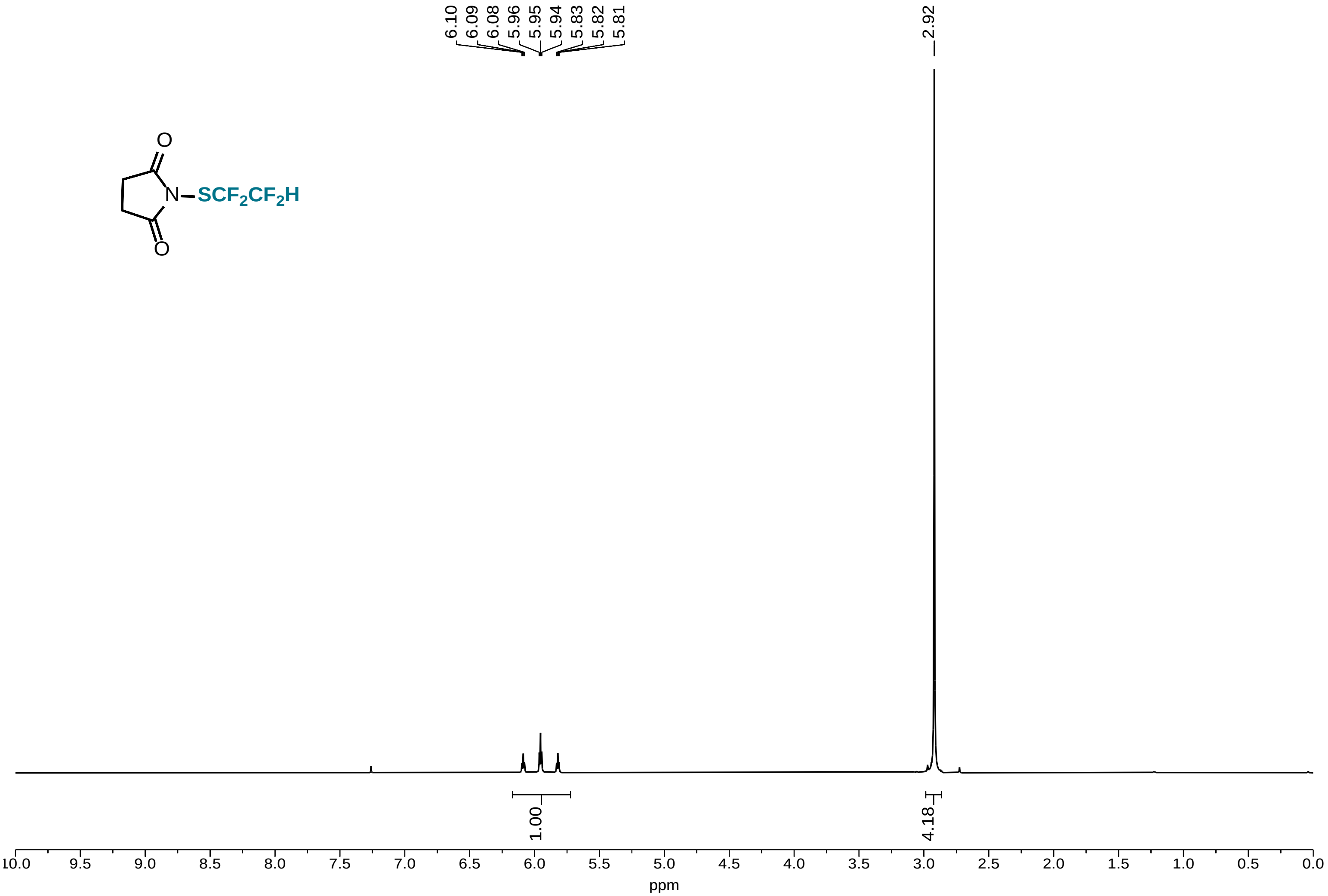

Figure S16. ^{1}H NMR ($CDCl_3$, 400 MHz) of **2a**

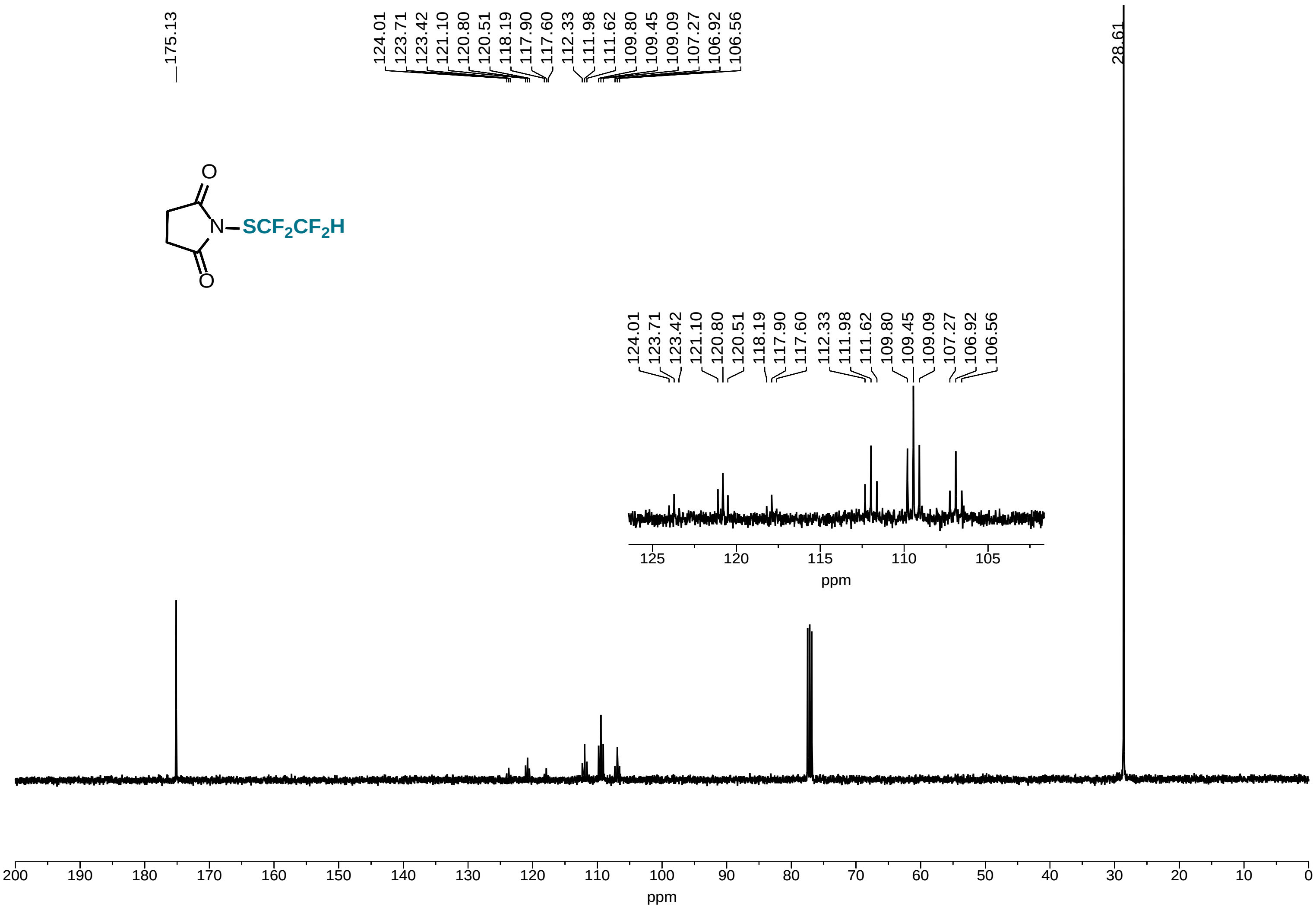

Figure S17. $^{13}C\{^1H\}$ NMR ($CDCl_3$, 100.6 MHz) of **2a**

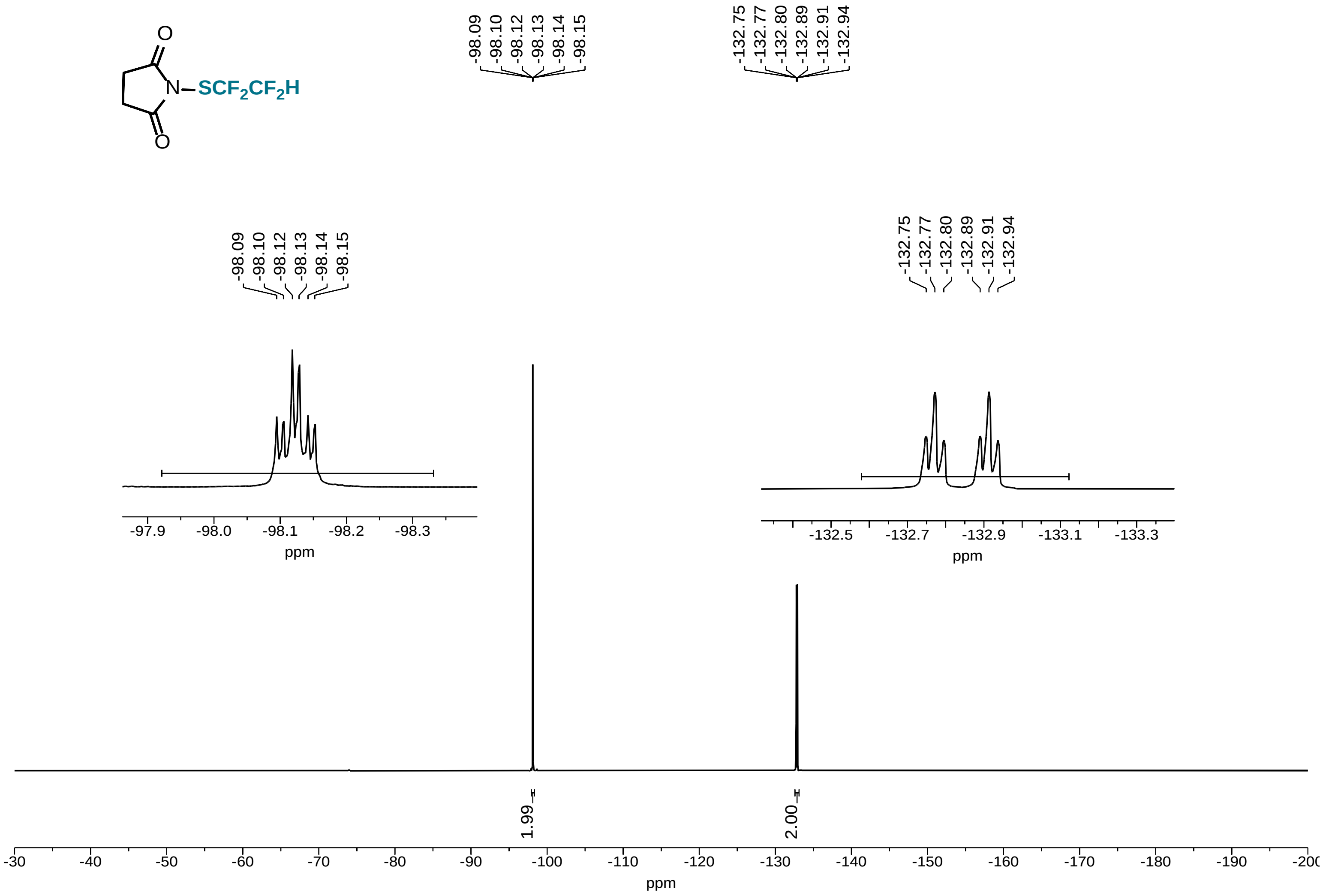

Figure S18. ^{19}F NMR ($CDCl_3$, 376.5 MHz) of **2a**

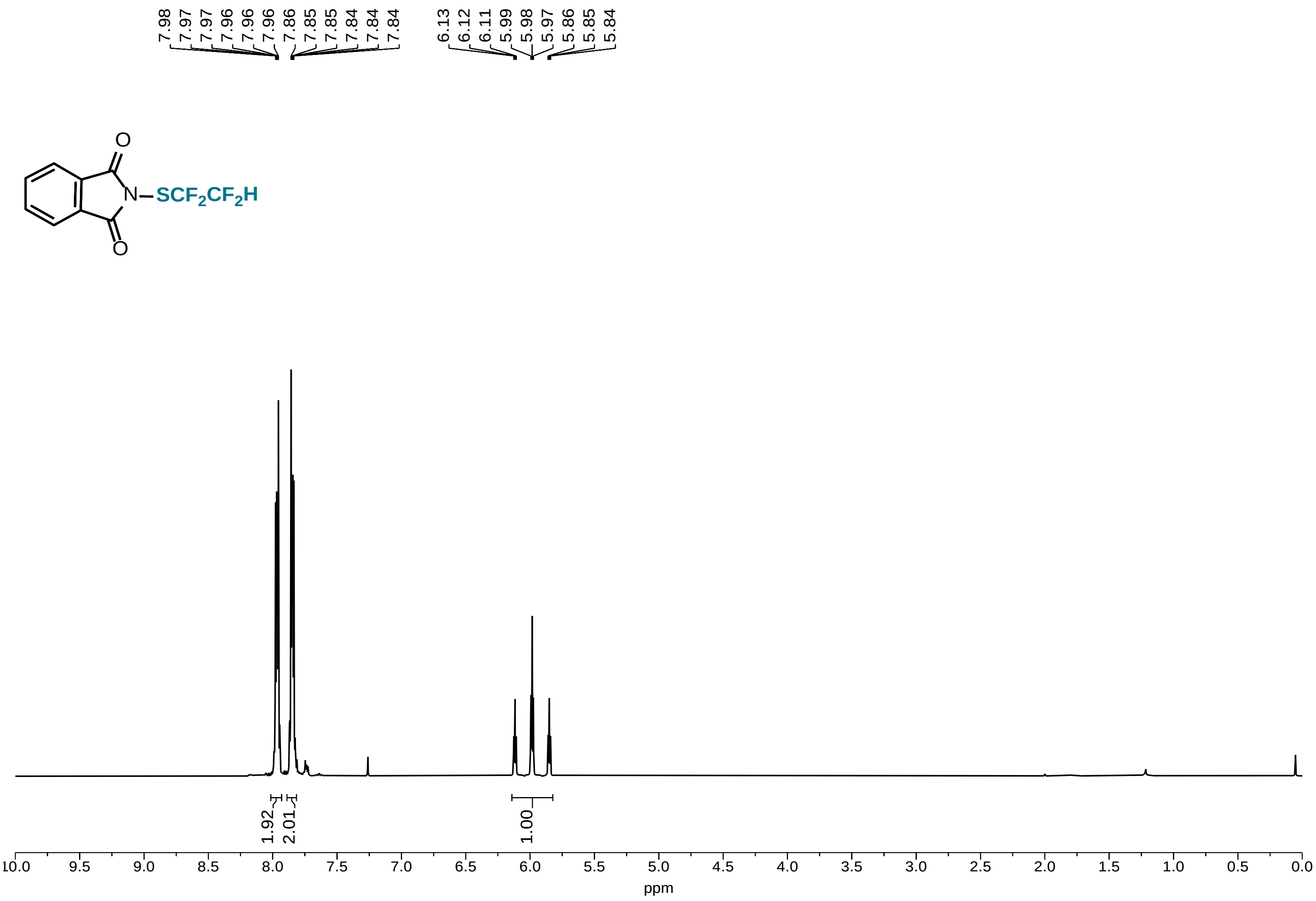

Figure S19. ^{1}H NMR ($CDCl_3$, 400 MHz) of **3a**

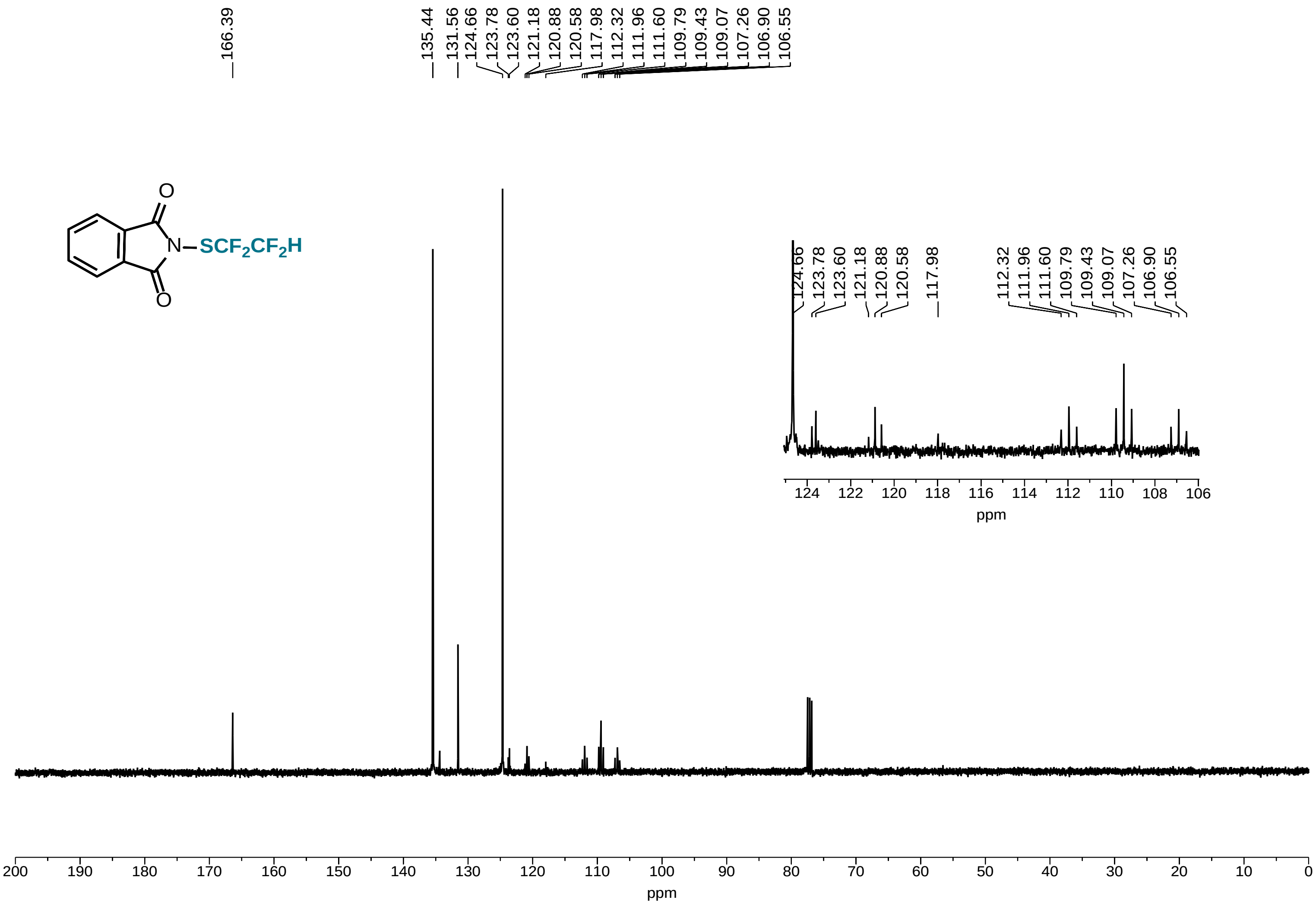

Figure S20. $^{13}C\{^{1}H\}$ NMR ($CDCl_3$, 100.6 MHz) of **3a**

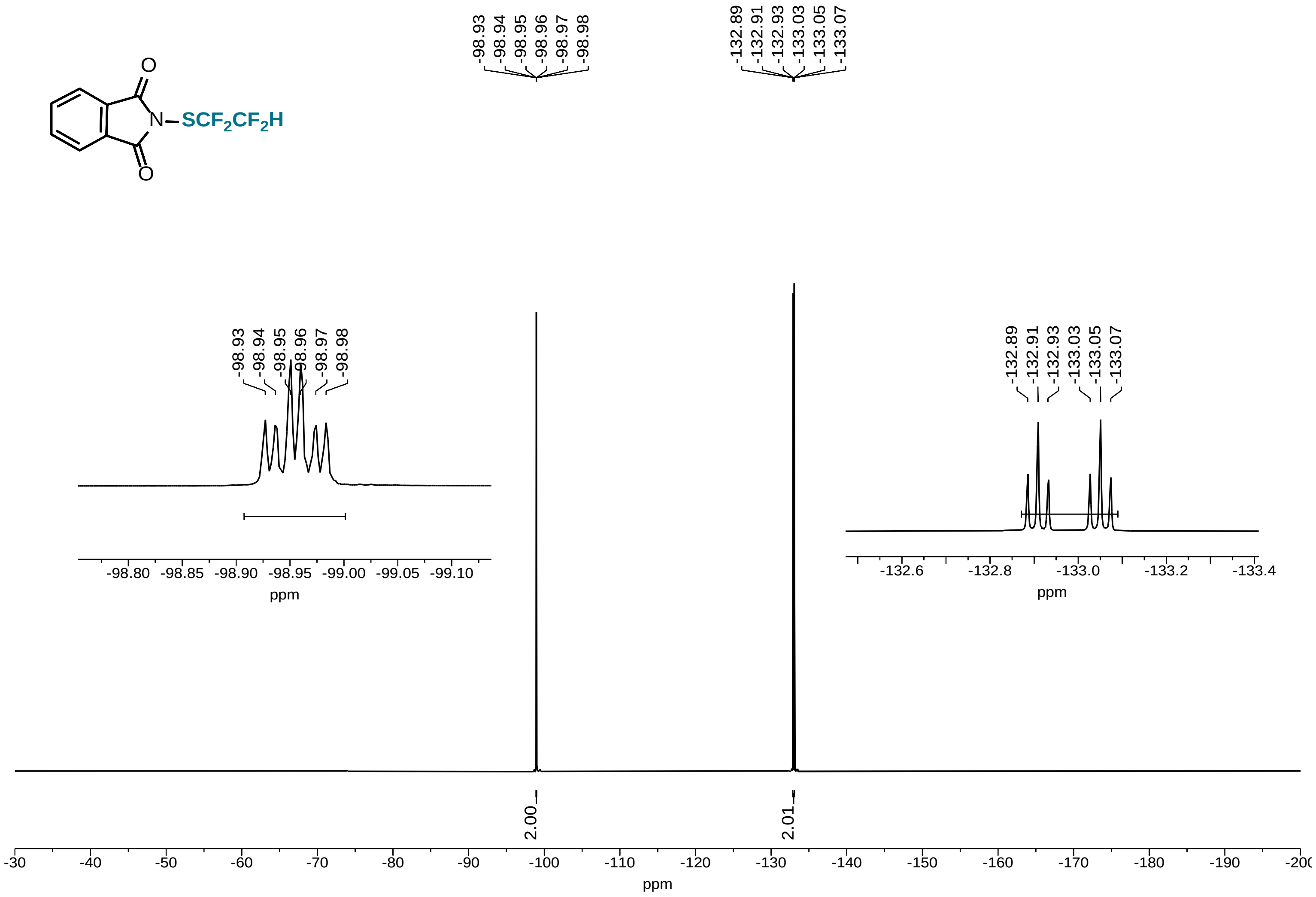

Figure S21. ^{19}F NMR ($CDCl_3$, 376.5 MHz) of **3a**

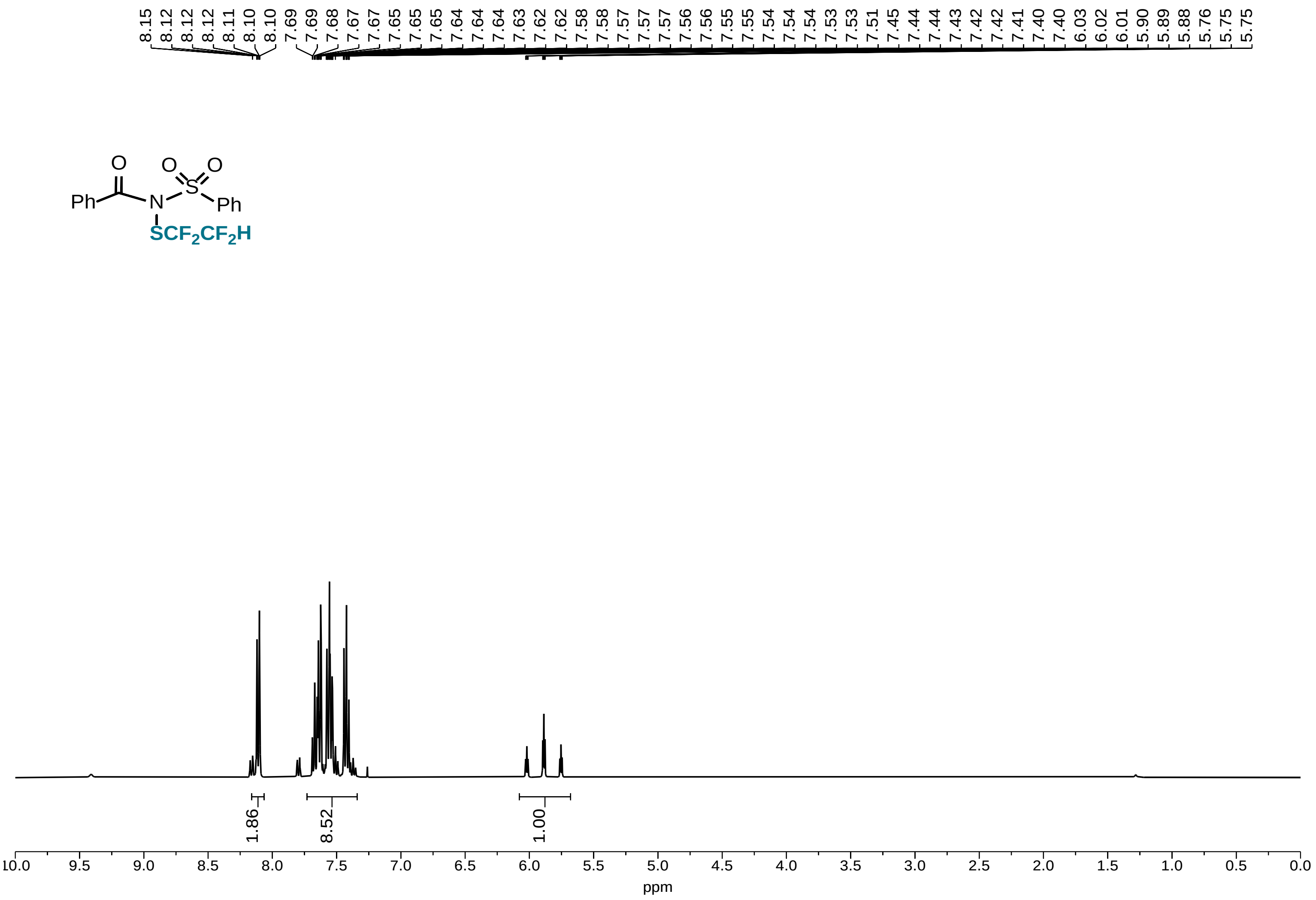

Figure S22. ^{1}H NMR ($CDCl_3$, 400 MHz) of **4a**

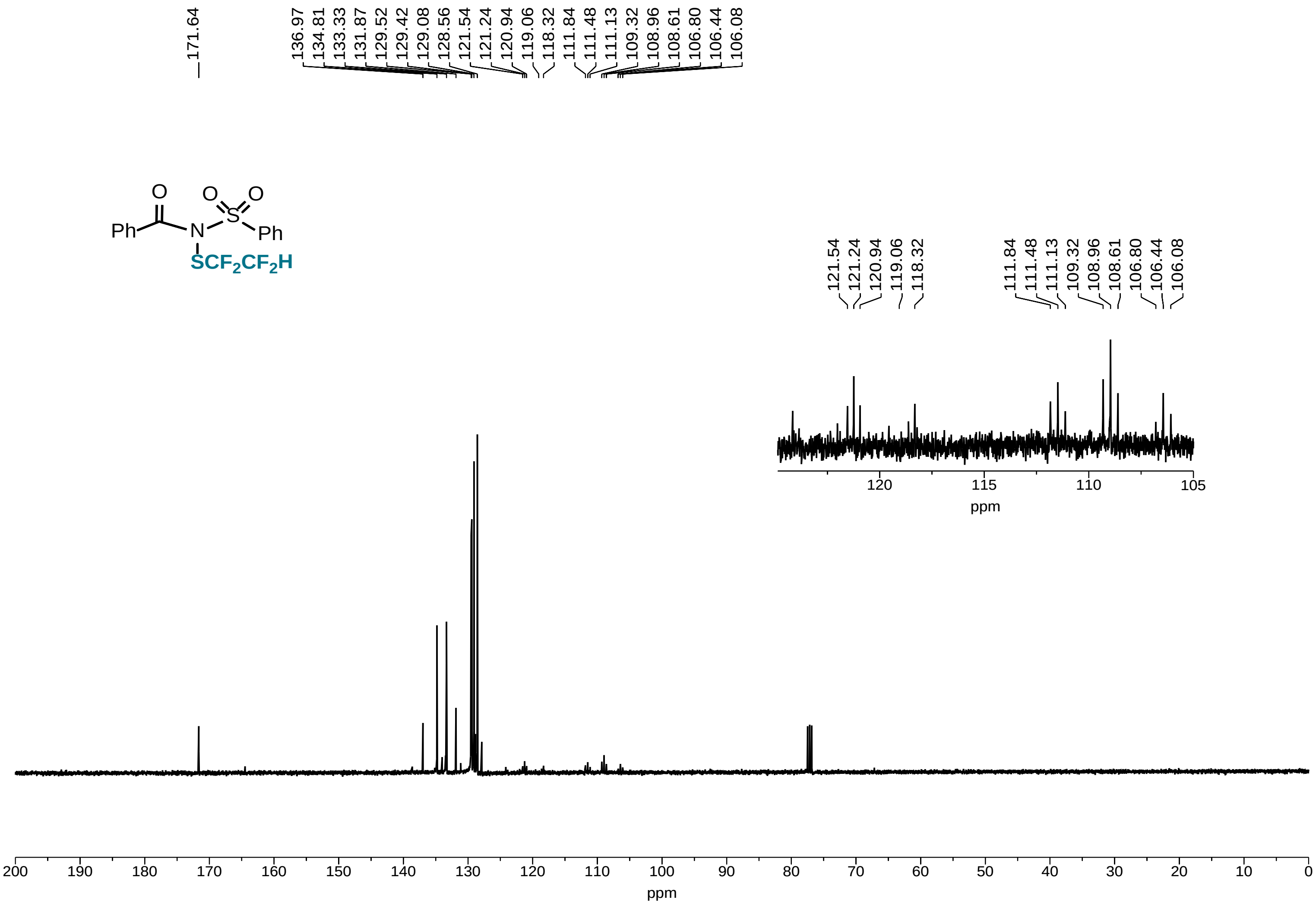

Figure S23. $^{13}C\{^{1}H\}$ NMR ($CDCl_3$, 100.6 MHz) of **4a**

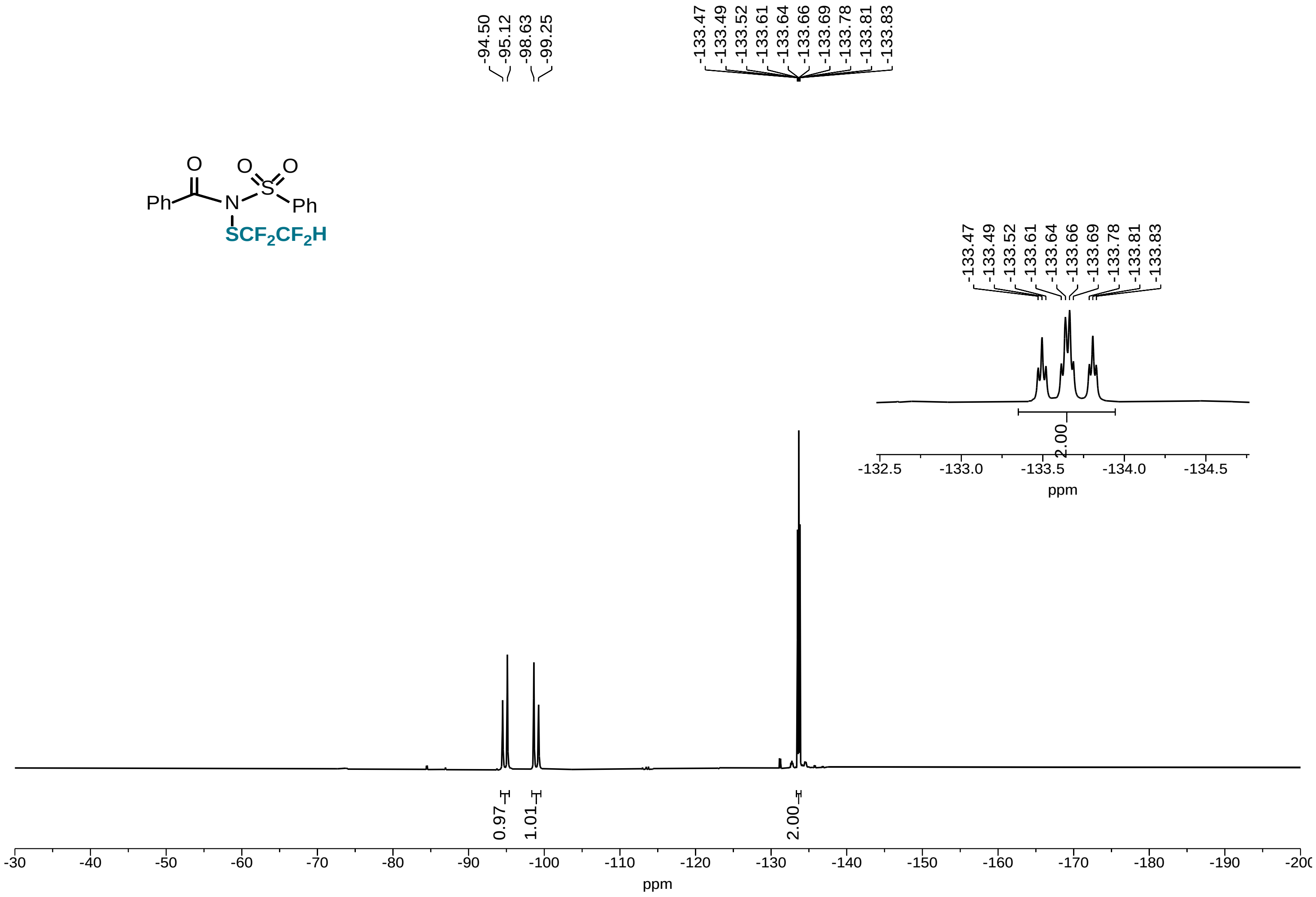

Figure S24. ^{19}F NMR ($CDCl_3$, 376.5 MHz) of **4a**

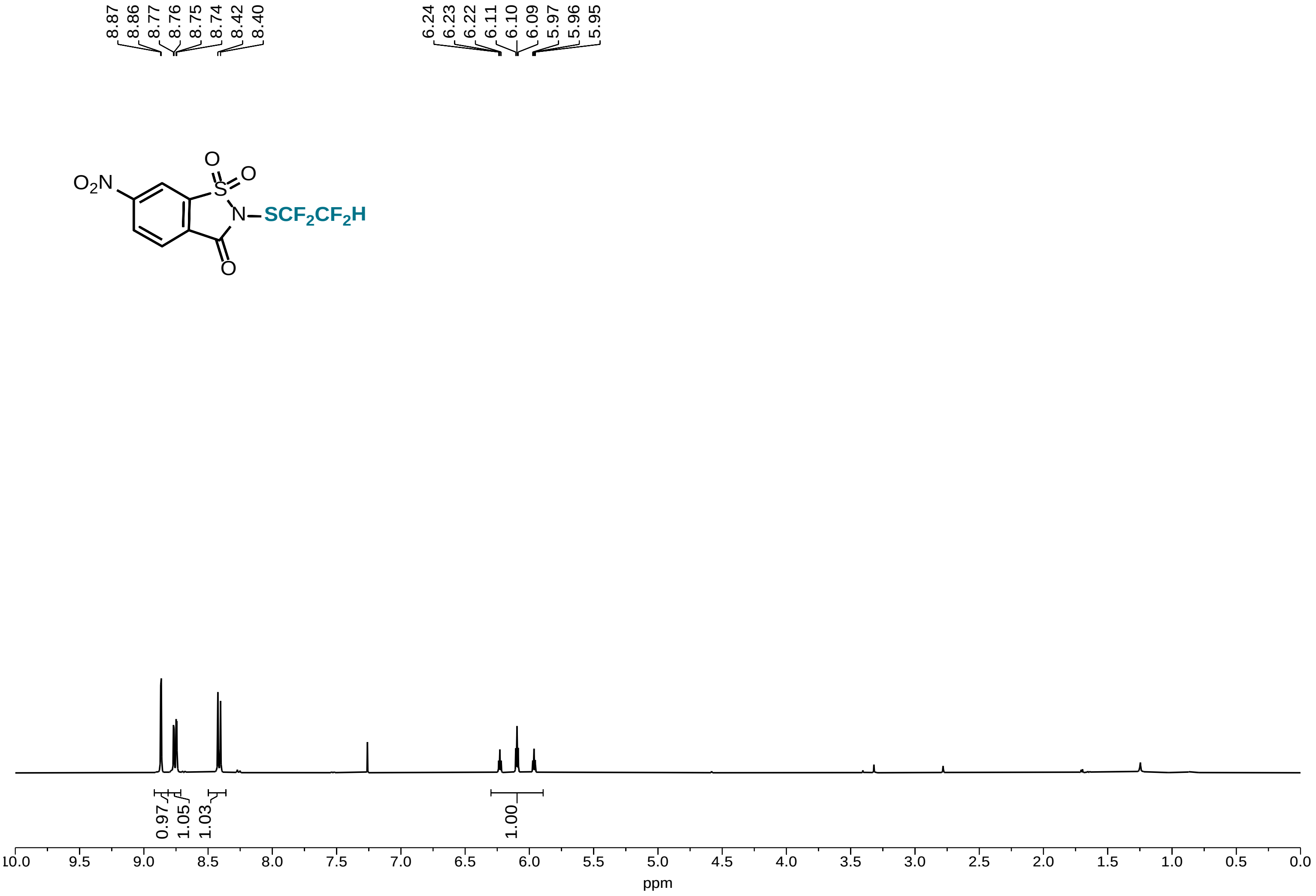

Figure S25. ^{1}H NMR (CD_2Cl_3, 400 MHz) of **5a**

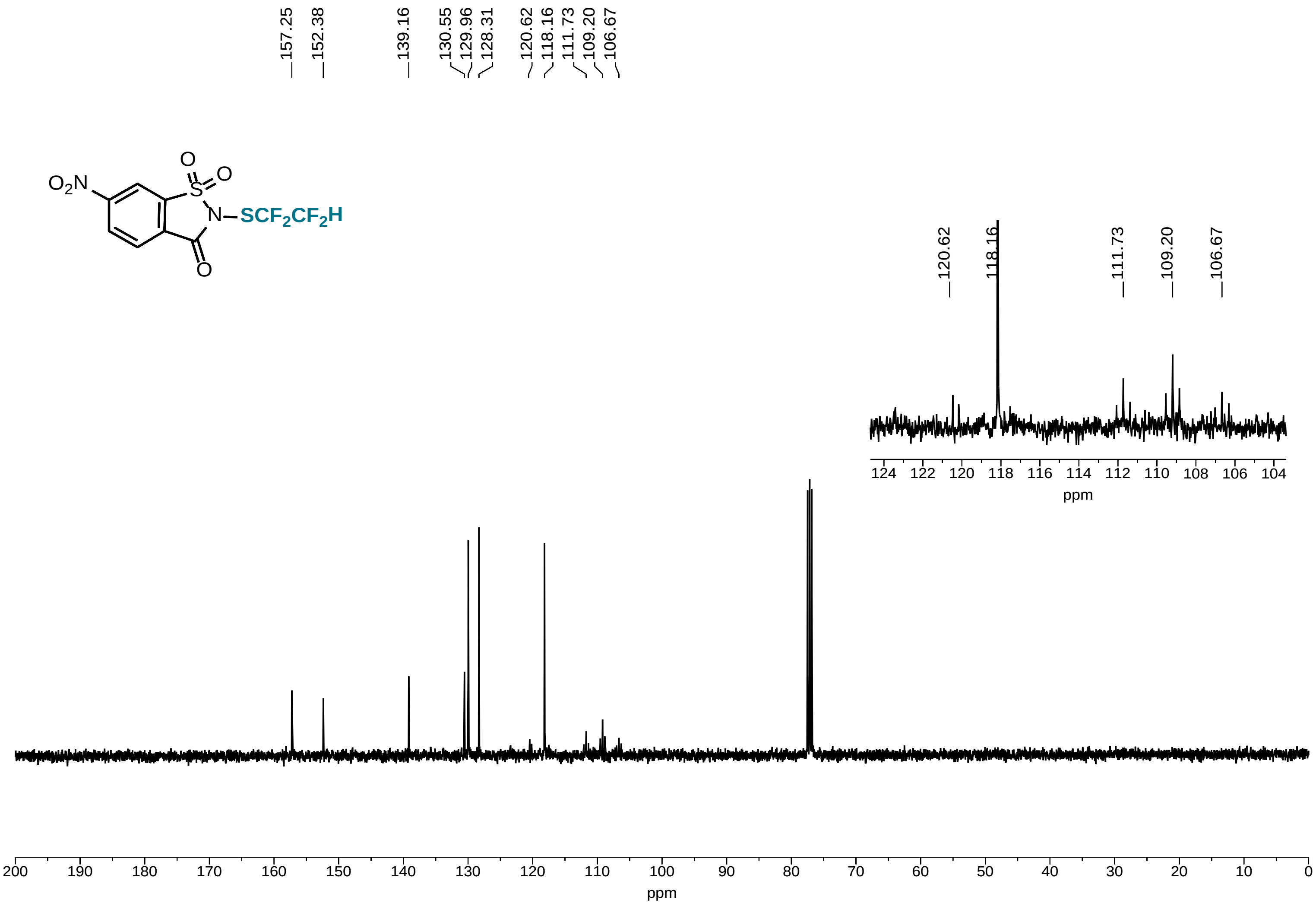

Figure S26. $^{13}C\{^{1}H\}$ NMR ($CDCl_3$, 100.6 MHz) of **5a**

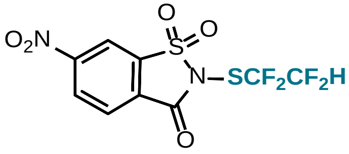

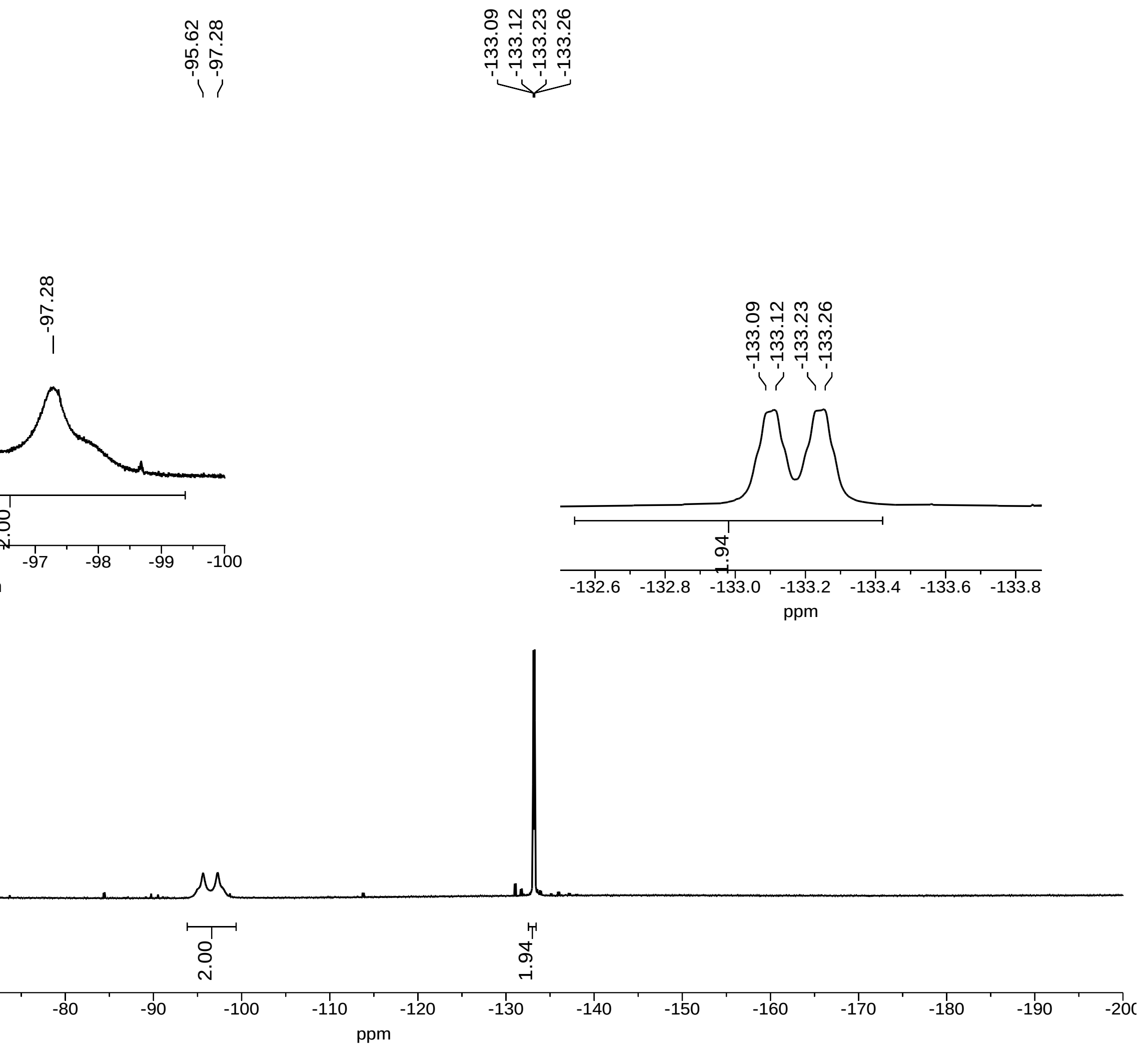

Figure S27. ${}^{19}F$ NMR ($CDCl_3$, 376.5 MHz) of **5a**

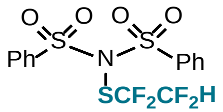

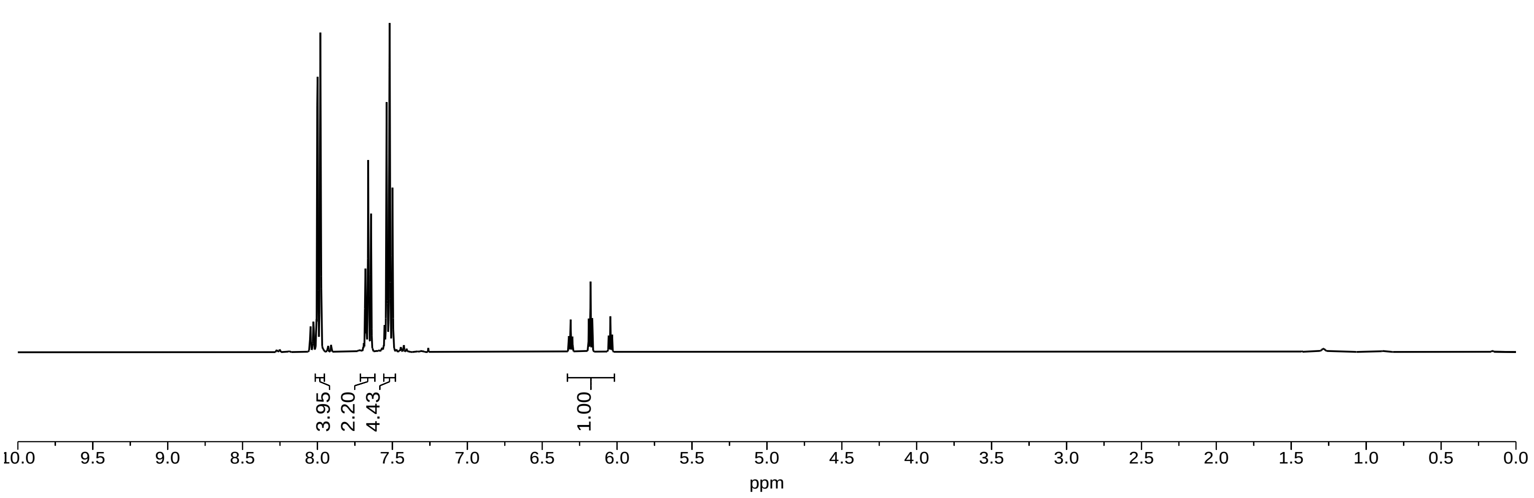

Figure S28. ^{1}H NMR ($CDCl_3$, 400 MHz) of **6a**

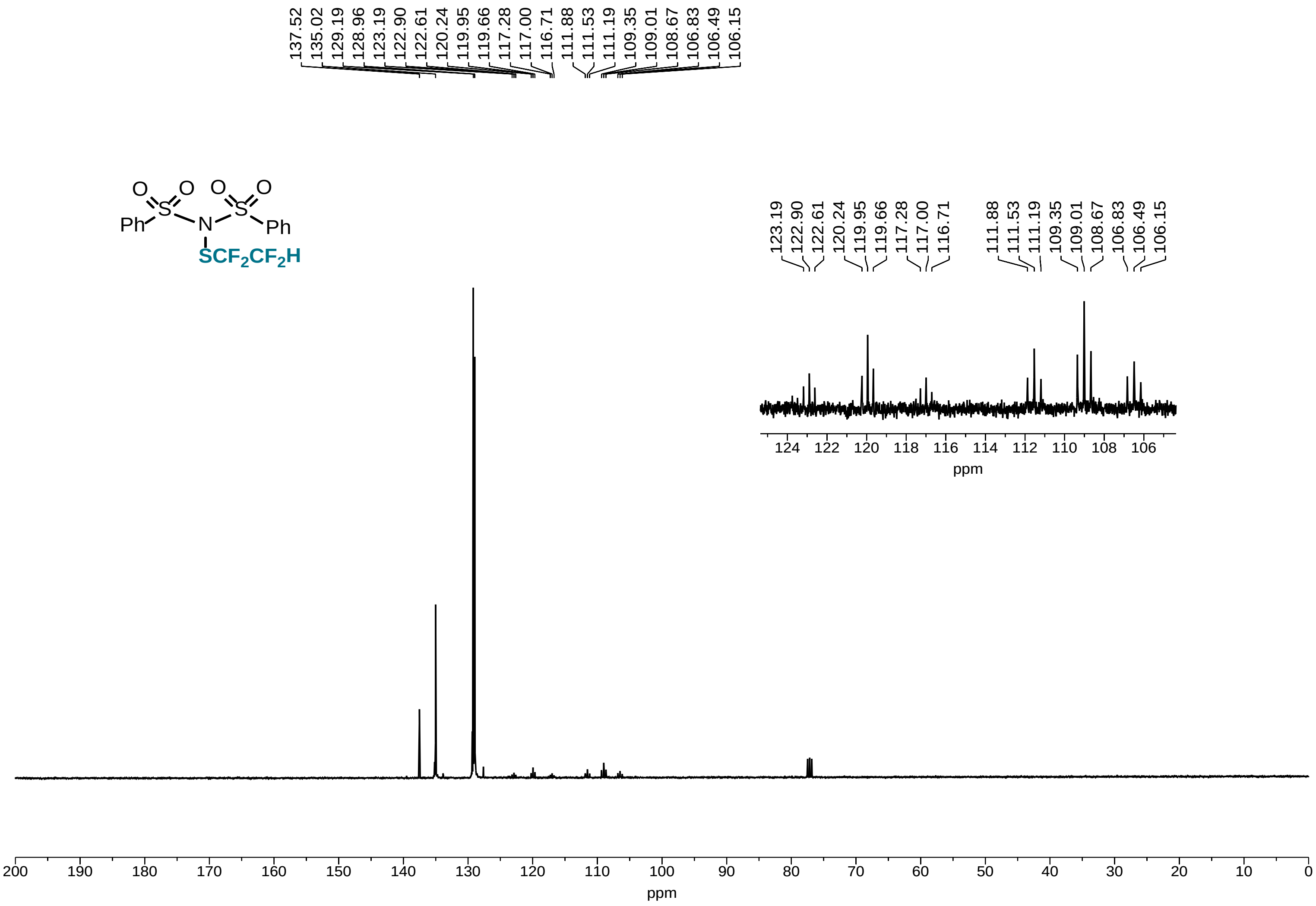

Figure S29. $^{13}C\{^{1}H\}$ NMR ($CDCl_3$, 100.6 MHz) of **6a**

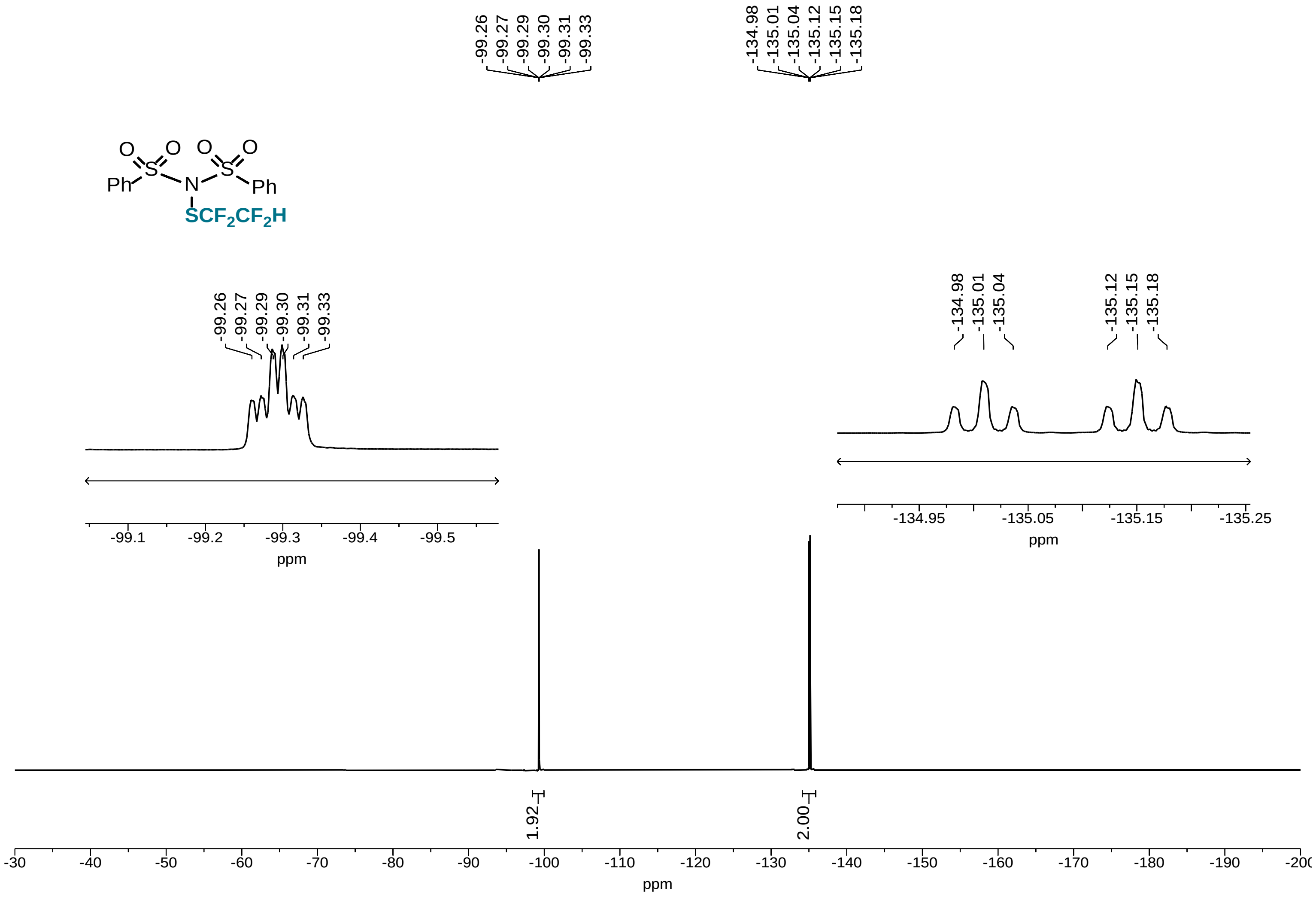

Figure S30. ^{19}F NMR ($CDCl_3$, 376.5 MHz) of **6a**

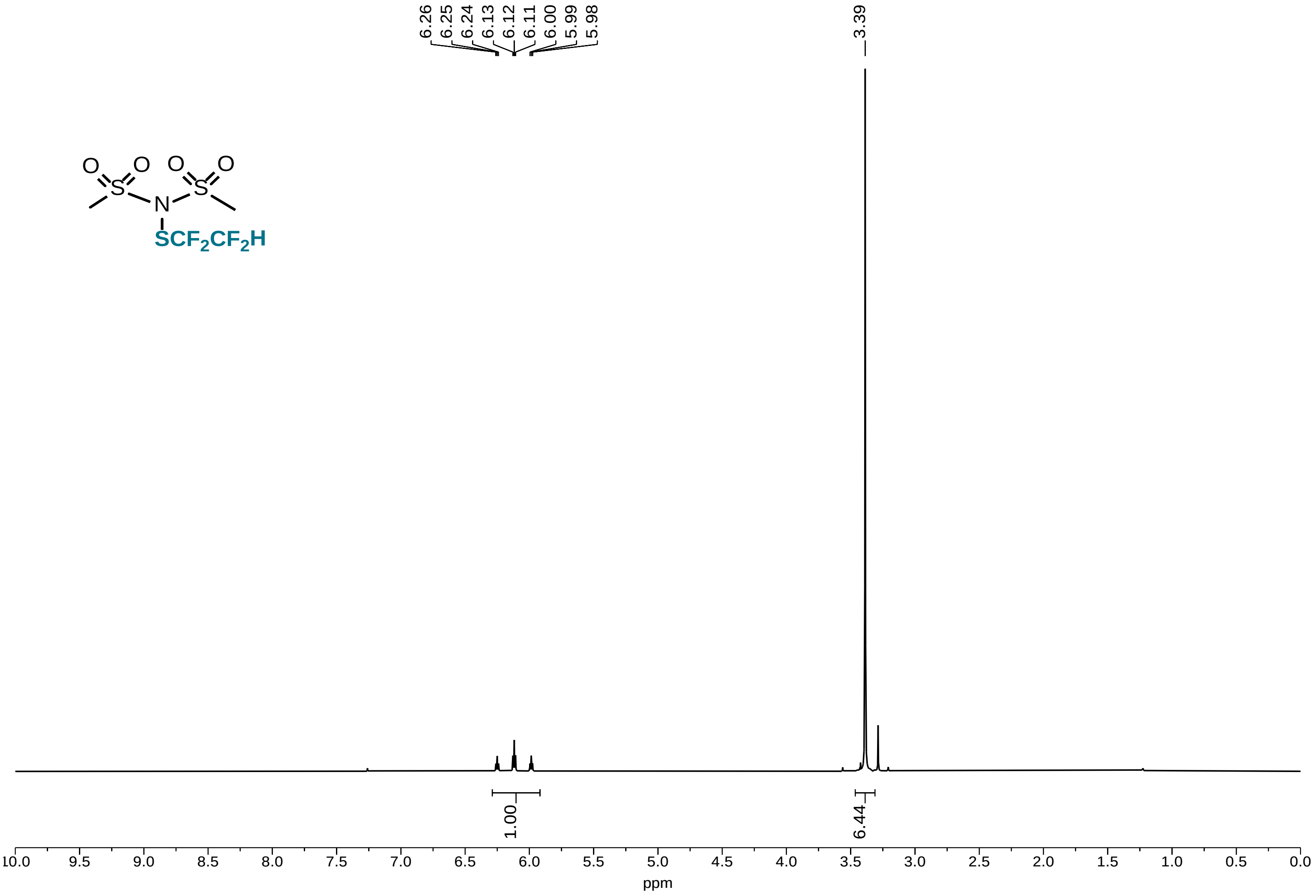

Figure S31. ^{1}H NMR ($CDCl_3$, 400 MHz) of **7a**

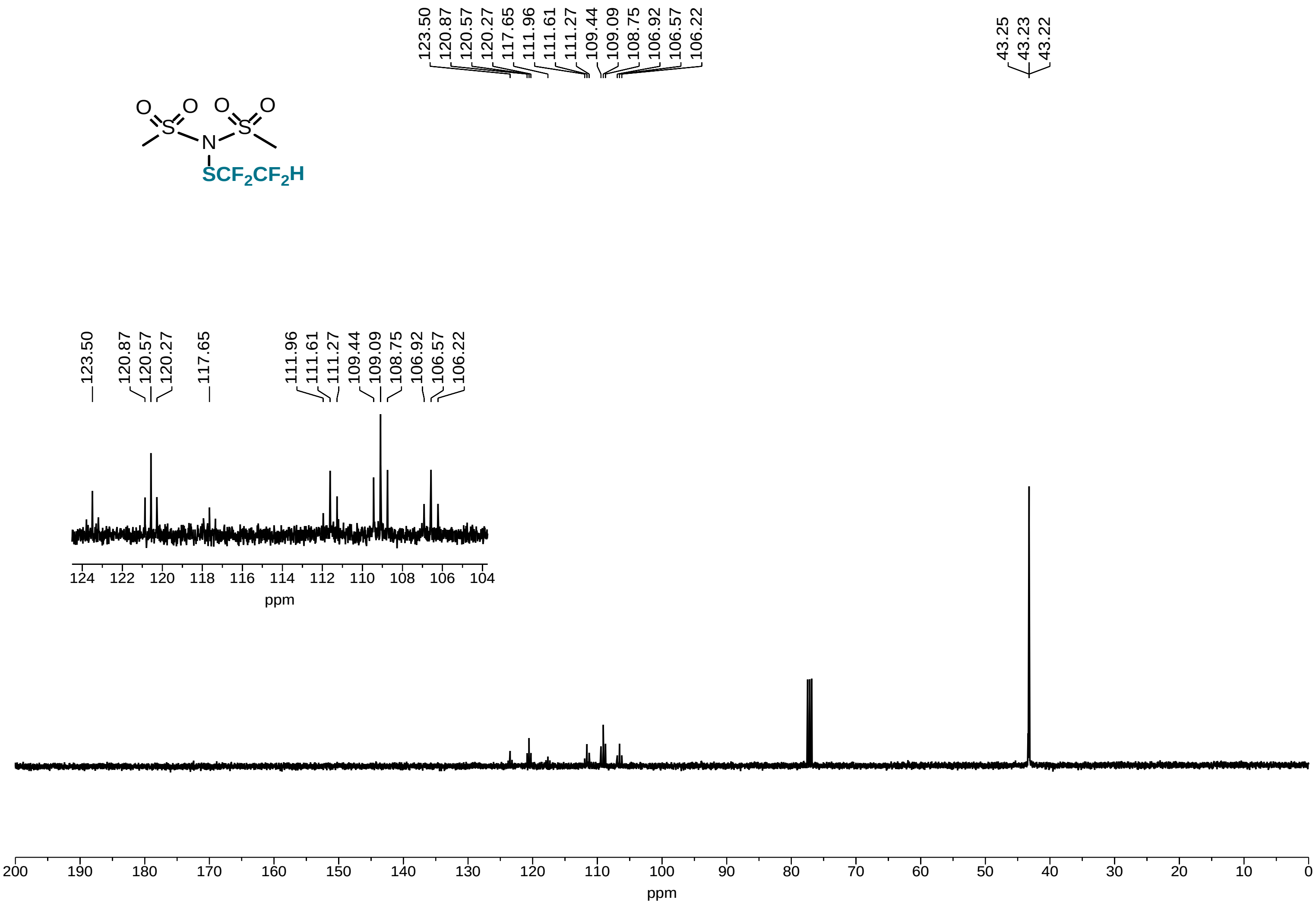

Figure S32. $^{13}C\{^{1}H\}$ NMR ($CDCl_3$, 100.6 MHz) of **7a**

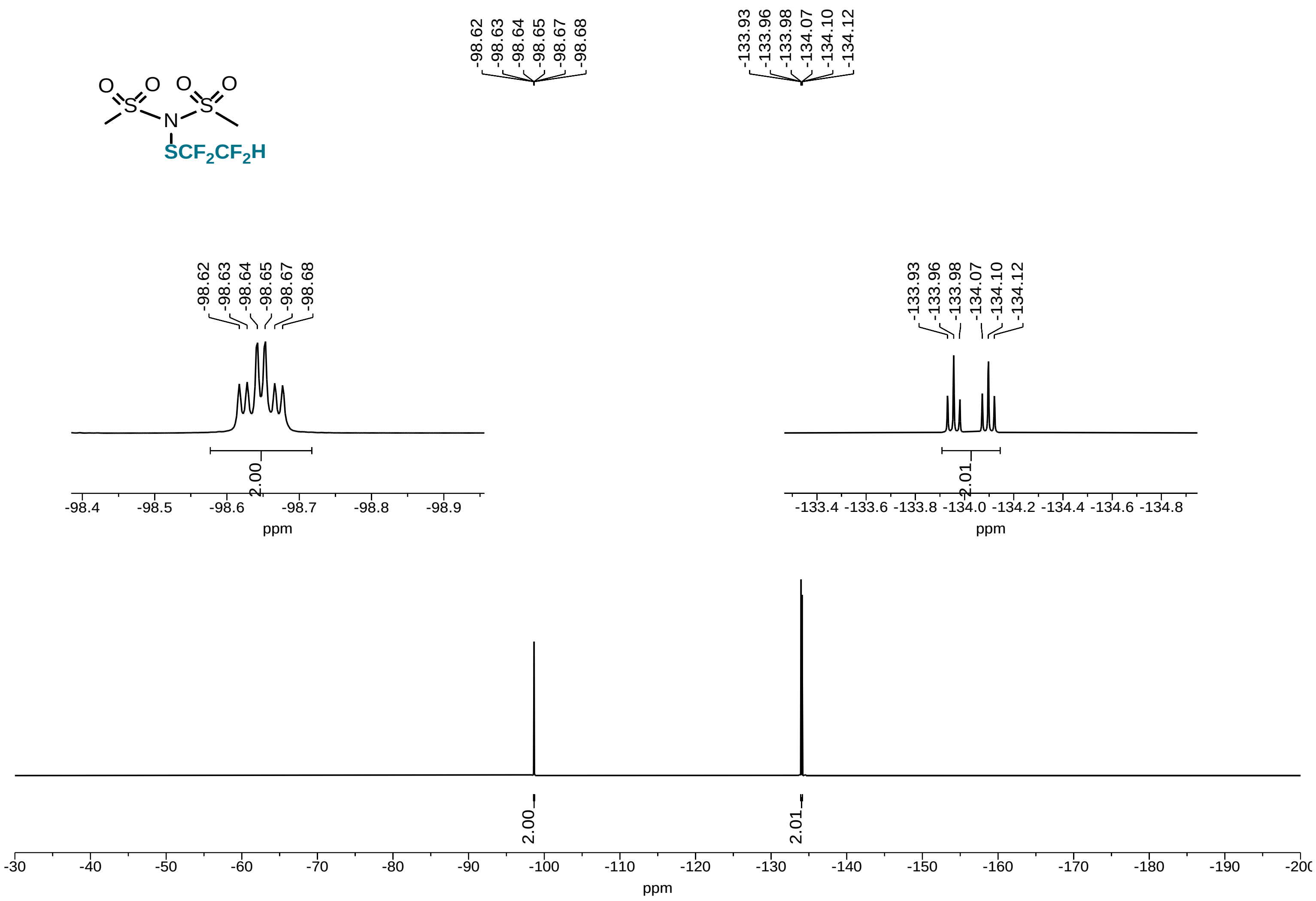

Figure S33. ^{19}F NMR ($CDCl_3$, 376.5 MHz) of **7a**

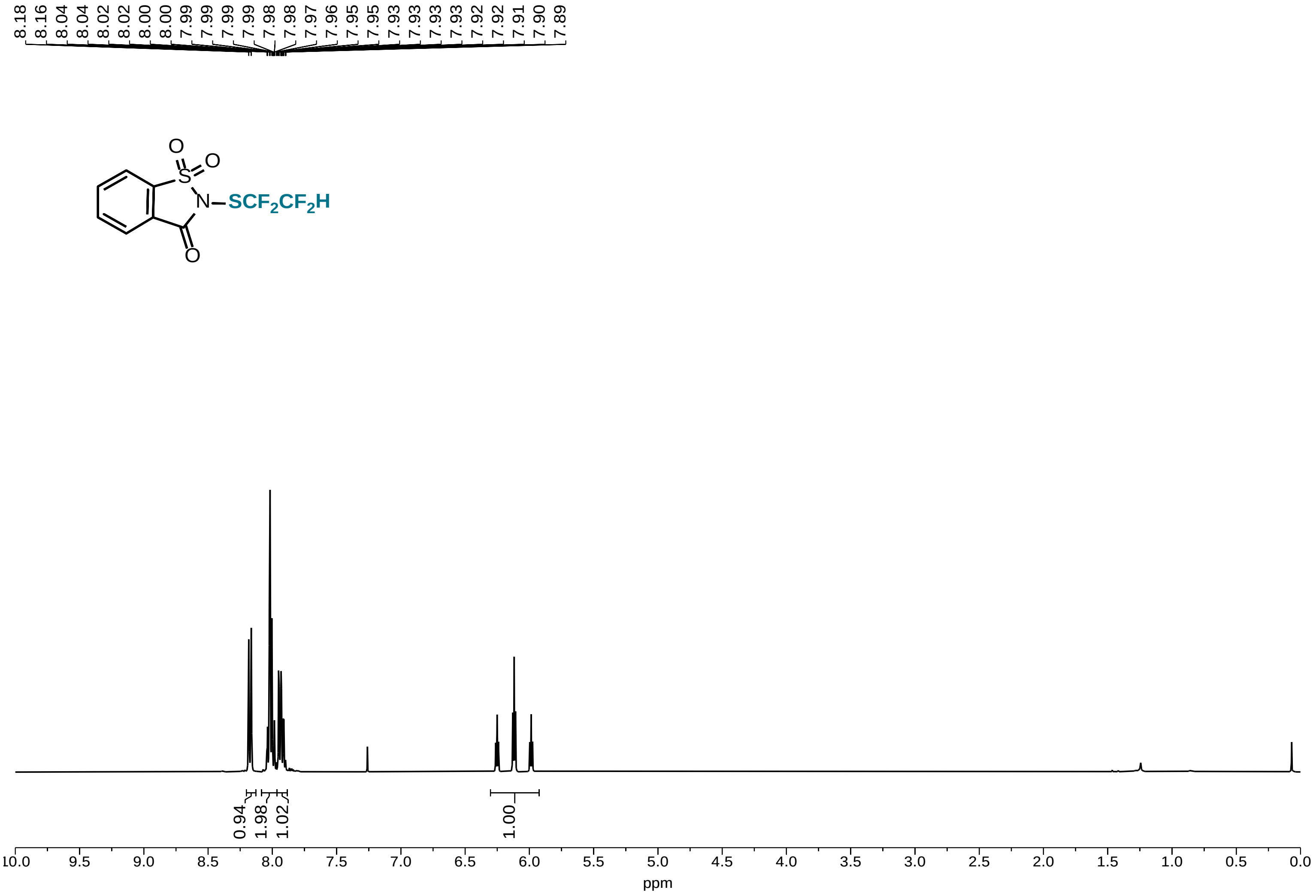

Figure S34. ^{1}H NMR ($CDCl_3$, 400 MHz) of **8a**

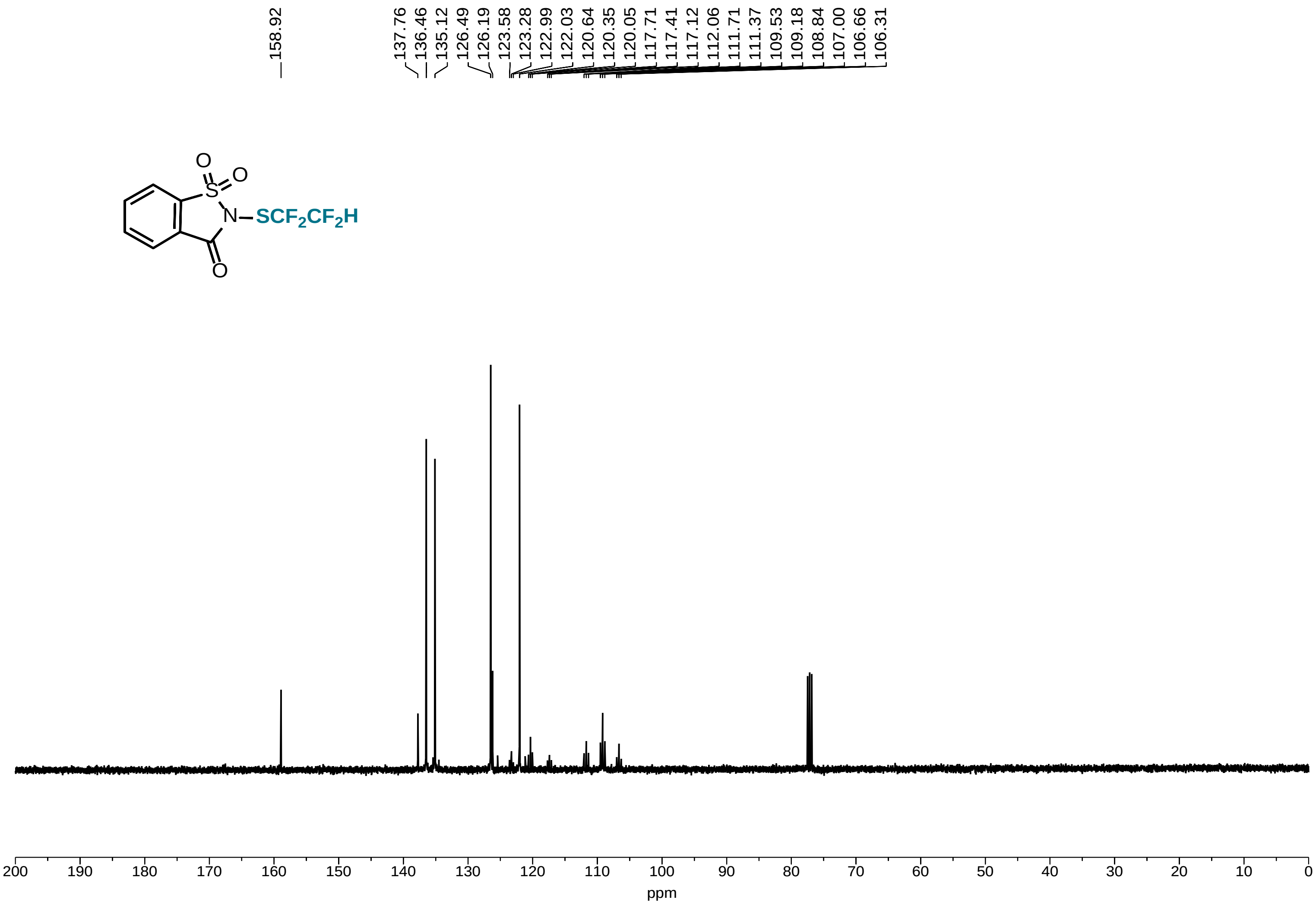

Figure S35. $^{13}C\{^{1}H\}$ NMR ($CDCl_3$, 100.6 MHz) of **8a**

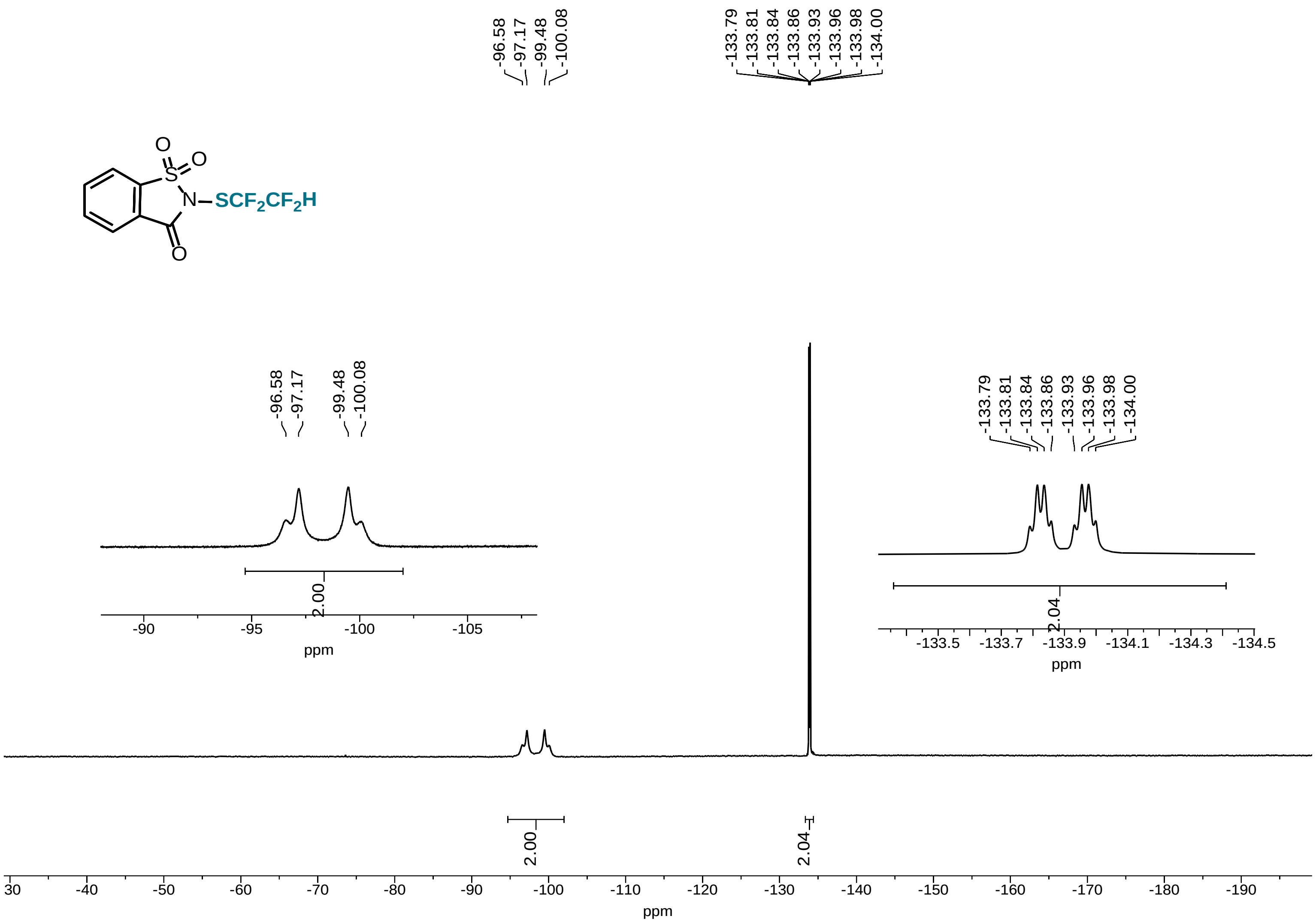

Figure S36. ^{19}F NMR ($CDCl_3$, 376.5 MHz) of **8a**

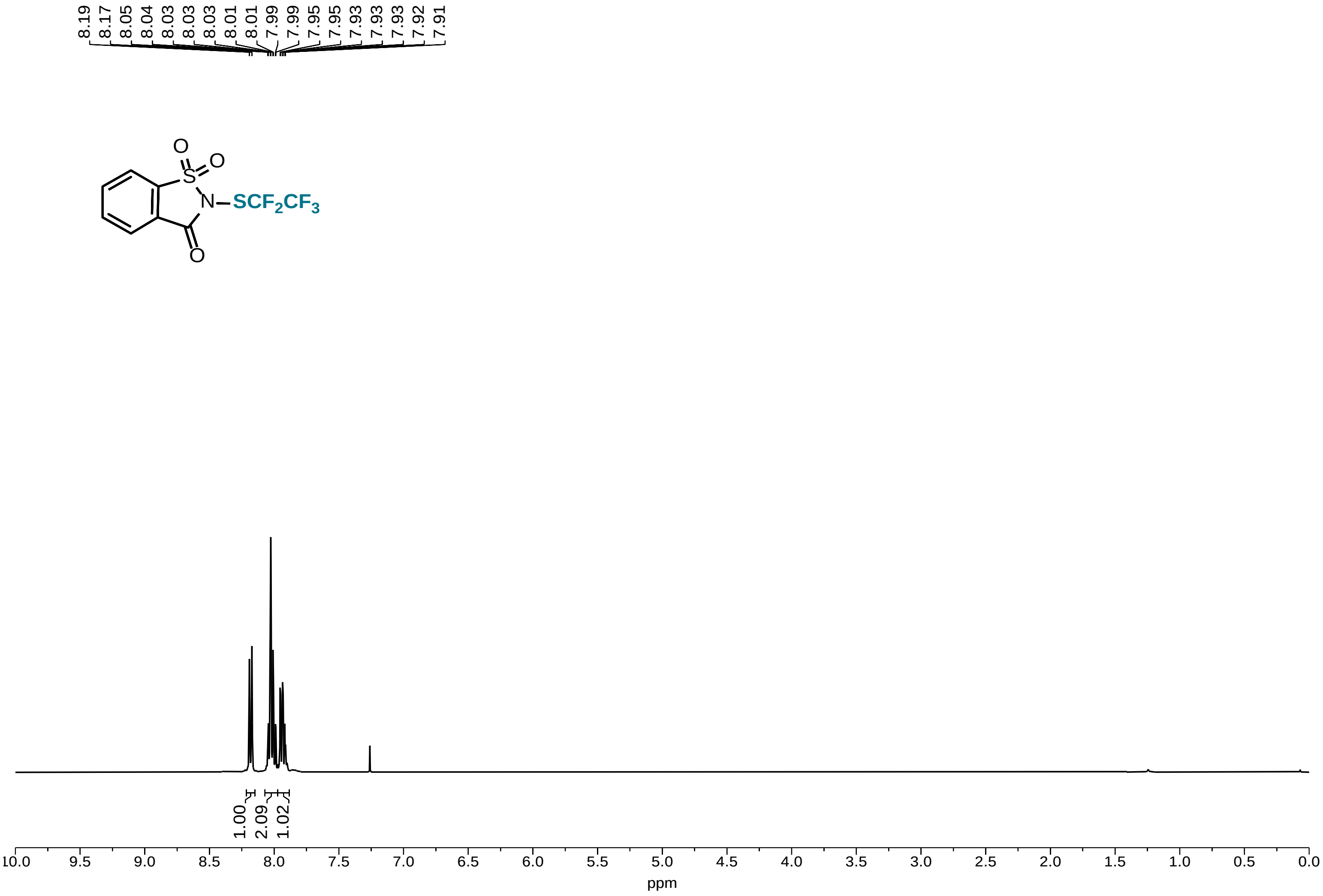

Figure S37. ^{1}H NMR ($CDCl_3$, 400 MHz) of **8b**

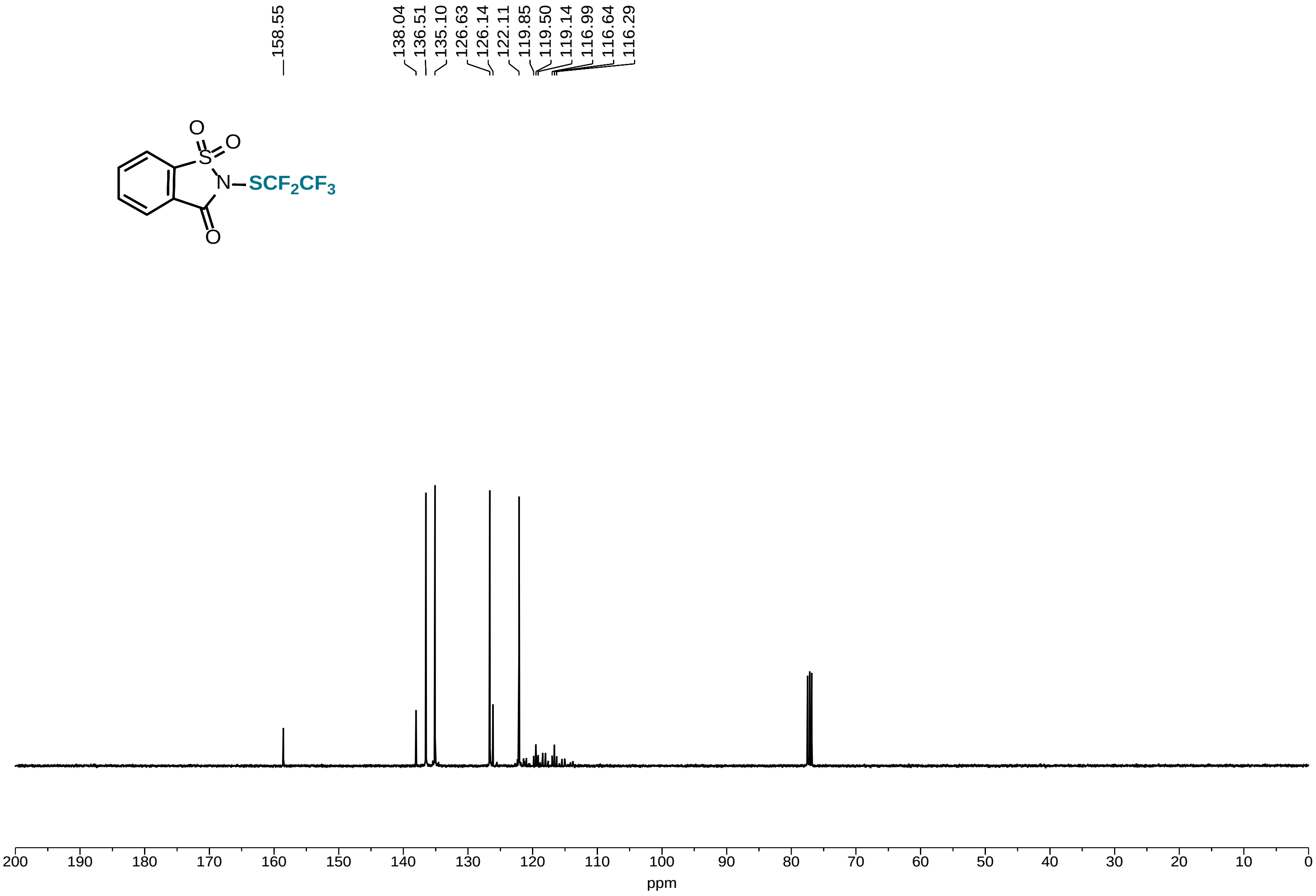

Figure S38. $^{13}C\{^{1}H\}$ NMR ($CDCl_3$, 100.6 MHz) of **8b**

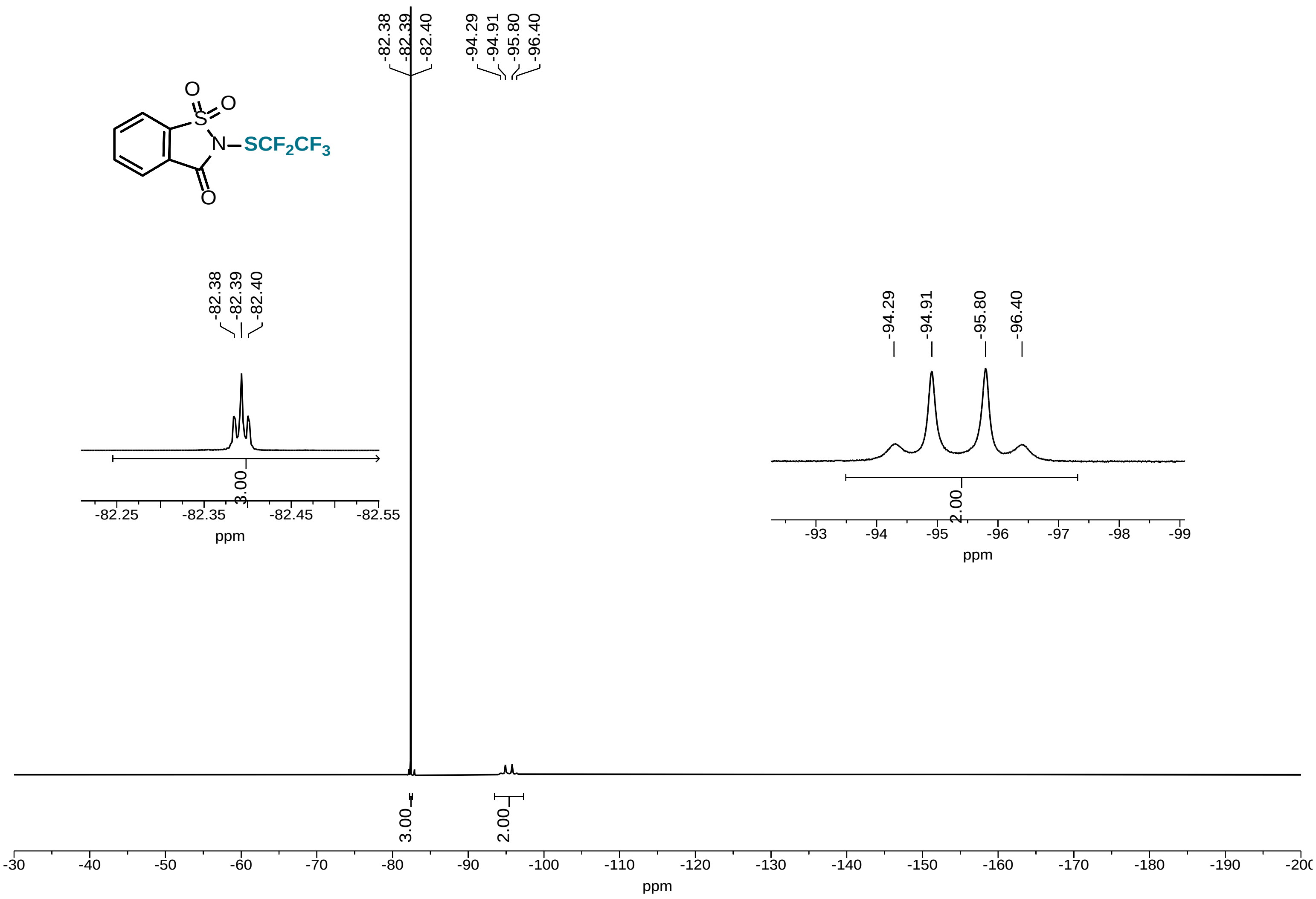

Figure S39. ^{19}F NMR ($CDCl_3$, 376.5 MHz) of **8b**

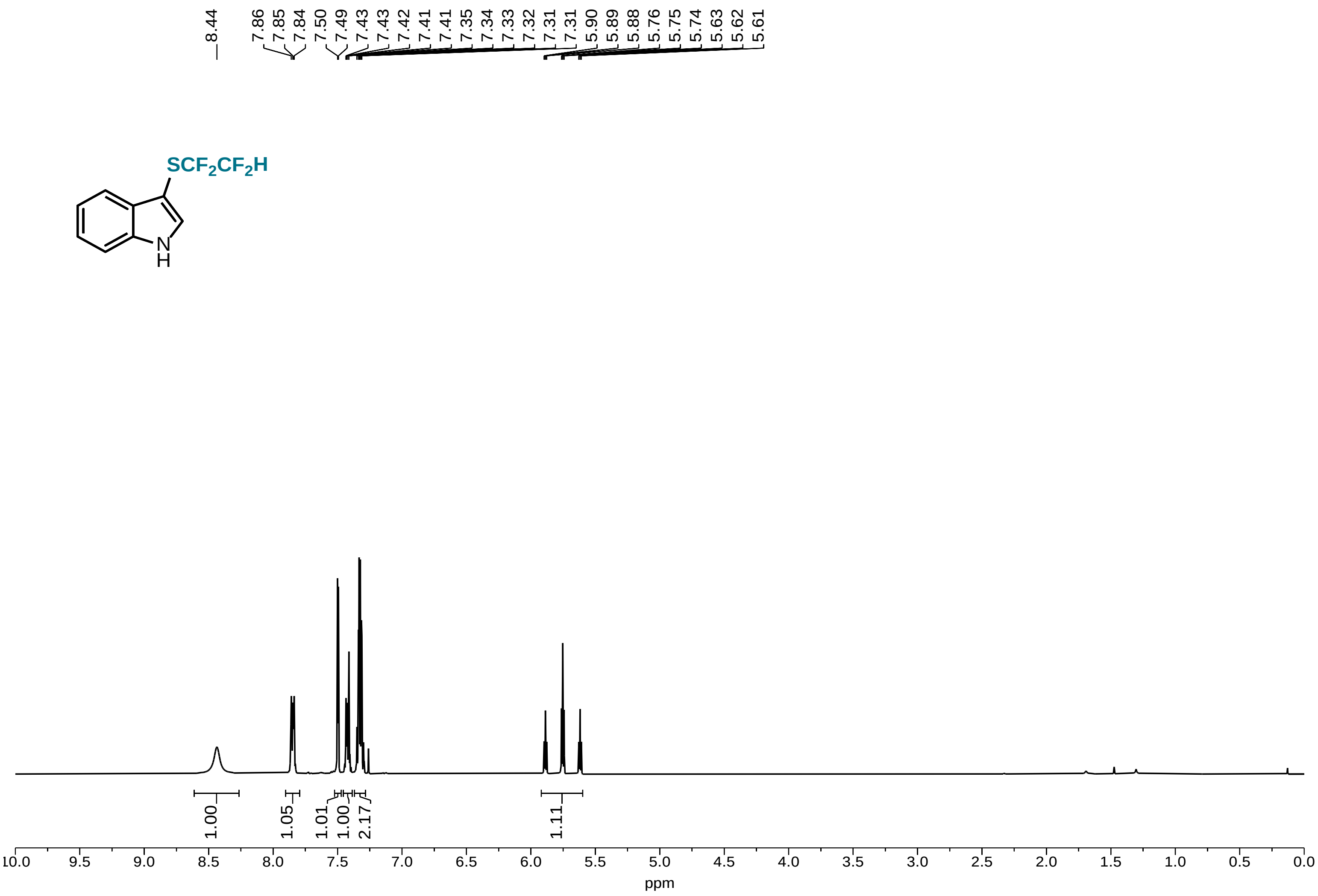

Figure S40. ^{1}H NMR ($CDCl_3$, 400 MHz) of **9a**

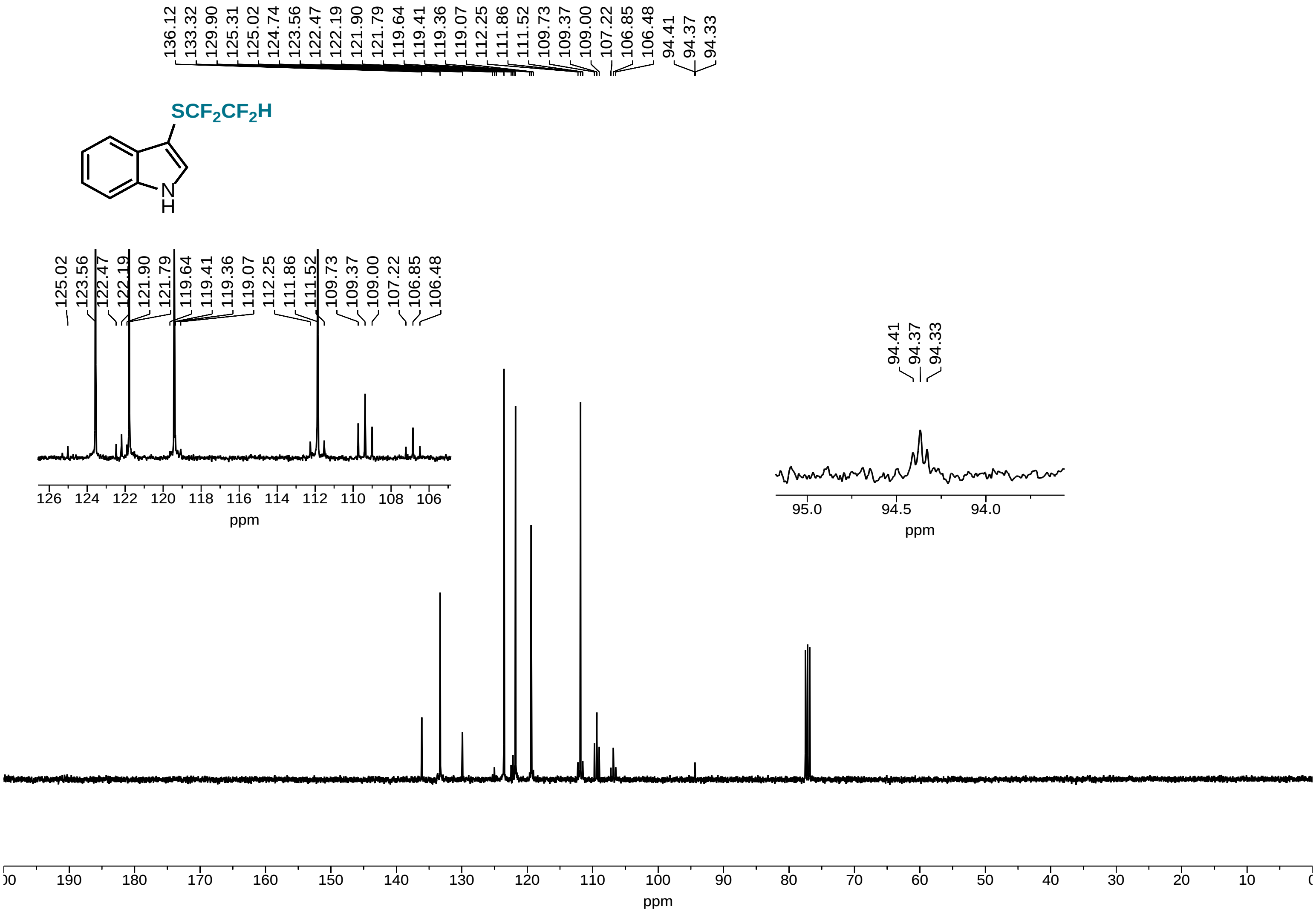

Figure S41. $^{13}C\{^{1}H\}$ NMR ($CDCl_3$, 100.6 MHz) of **9a**

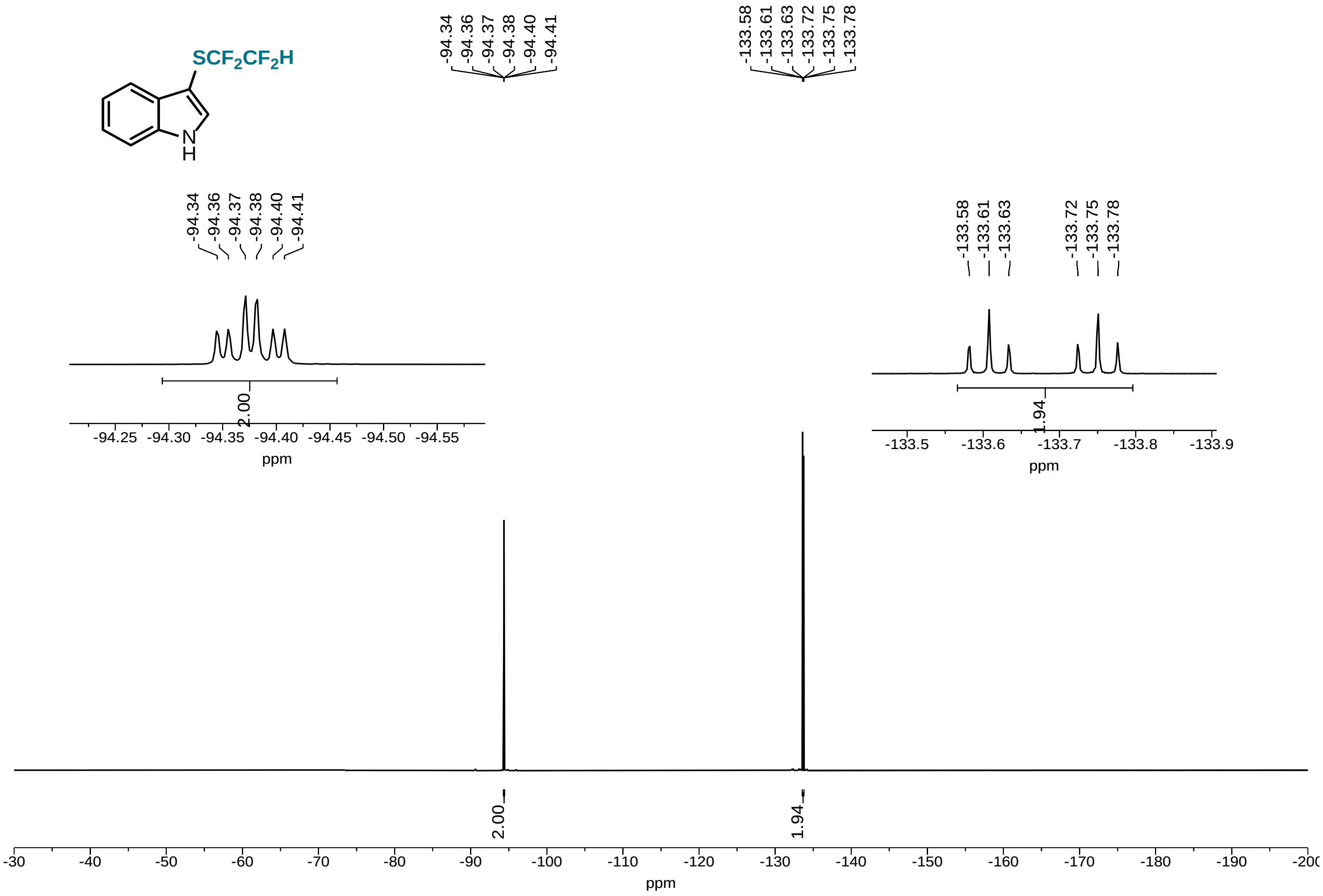

Figure S42. ^{19}F NMR ($CDCl_3$, 376.5 MHz) of **9a**

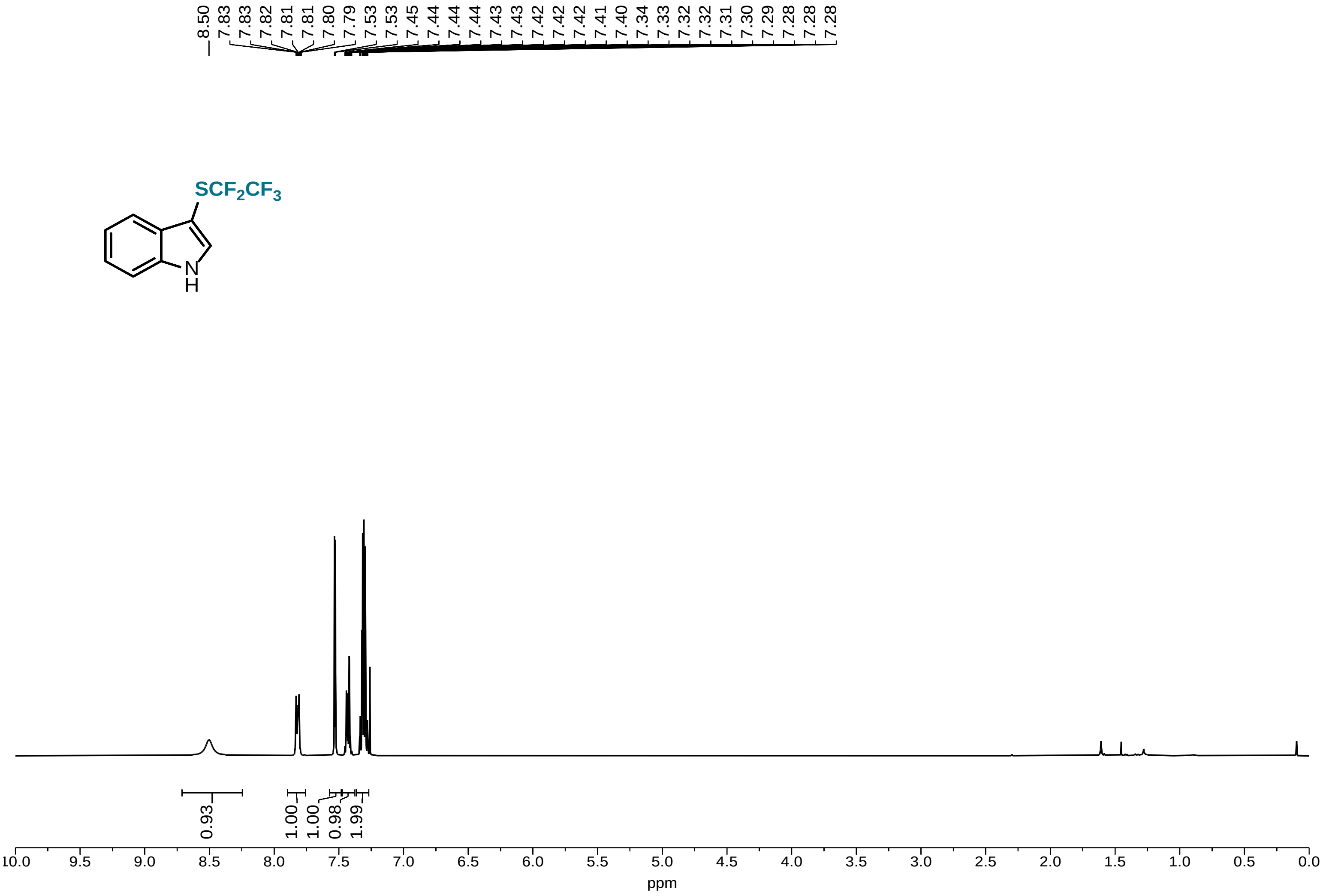

Figure S43. ^{1}H NMR ($CDCl_3$, 400 MHz) of **9b**

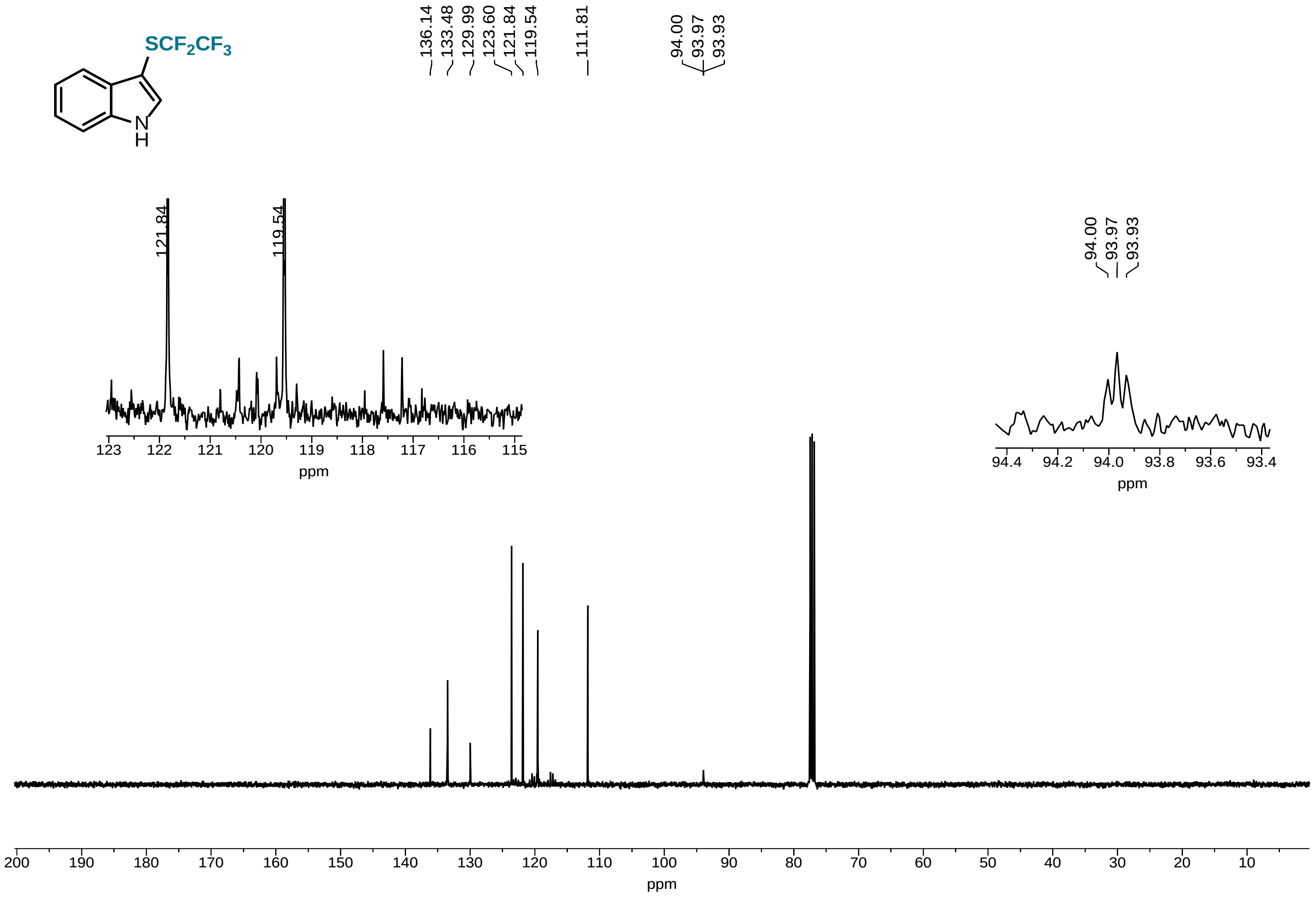

Figure S44. $^{13}C\{^1H\}$ NMR ($CDCl_3$, 100.6 MHz) of **9b**

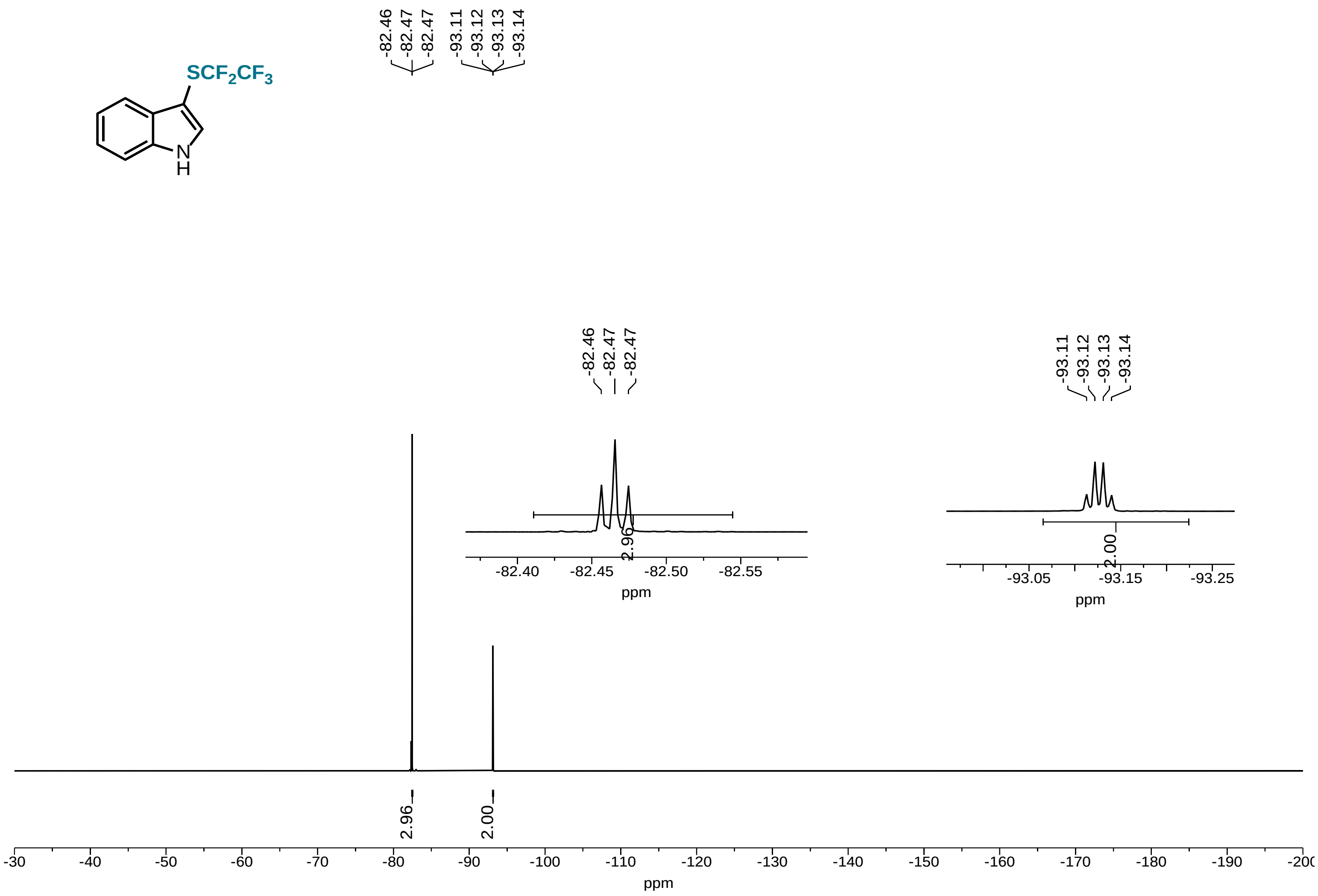

Figure S45. ^{19}F NMR ($CDCl_3$, 376.5 MHz) of **9b**

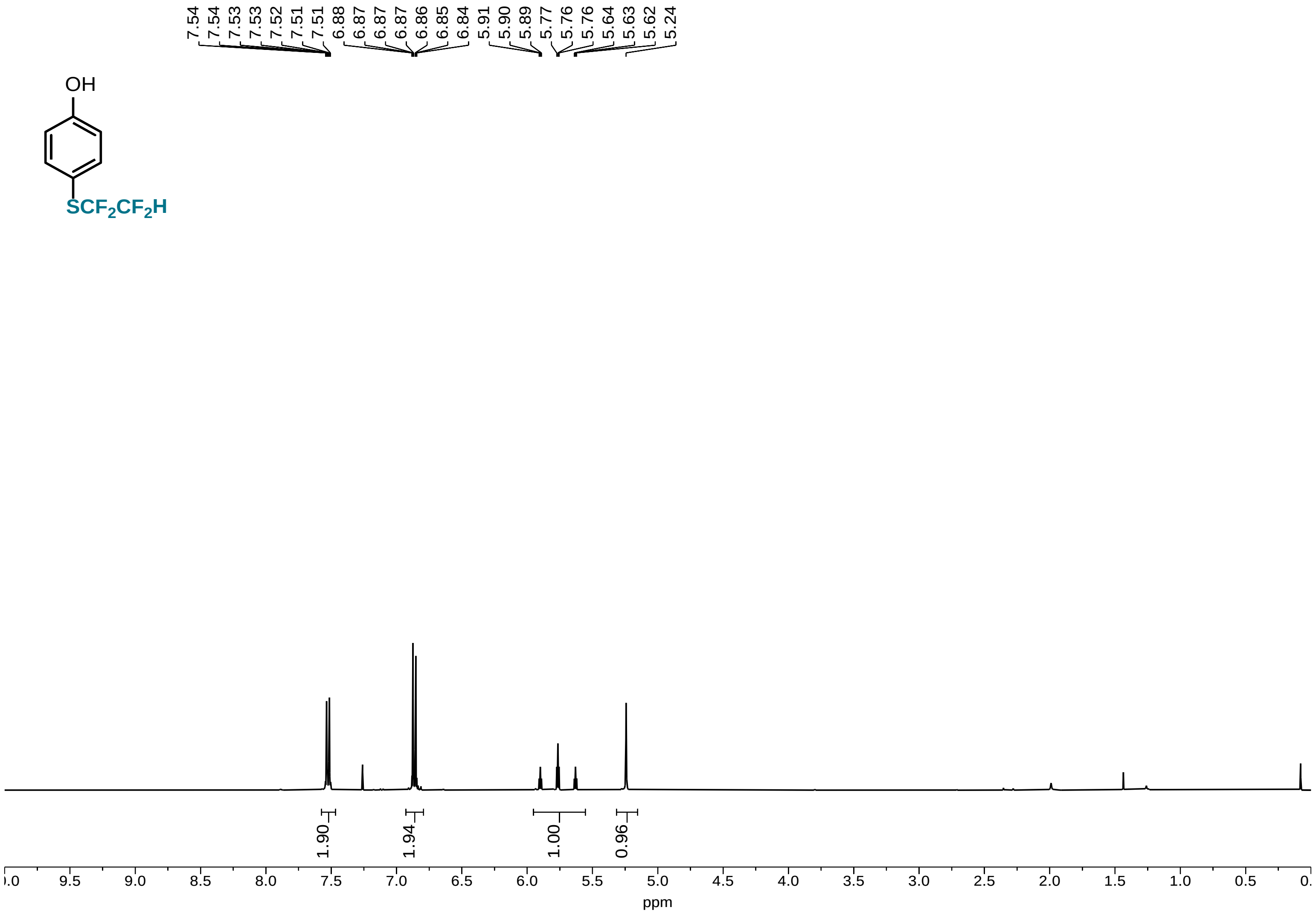

Figure S46. ^{1}H NMR ($CDCl_3$, 400 MHz) of **10a**

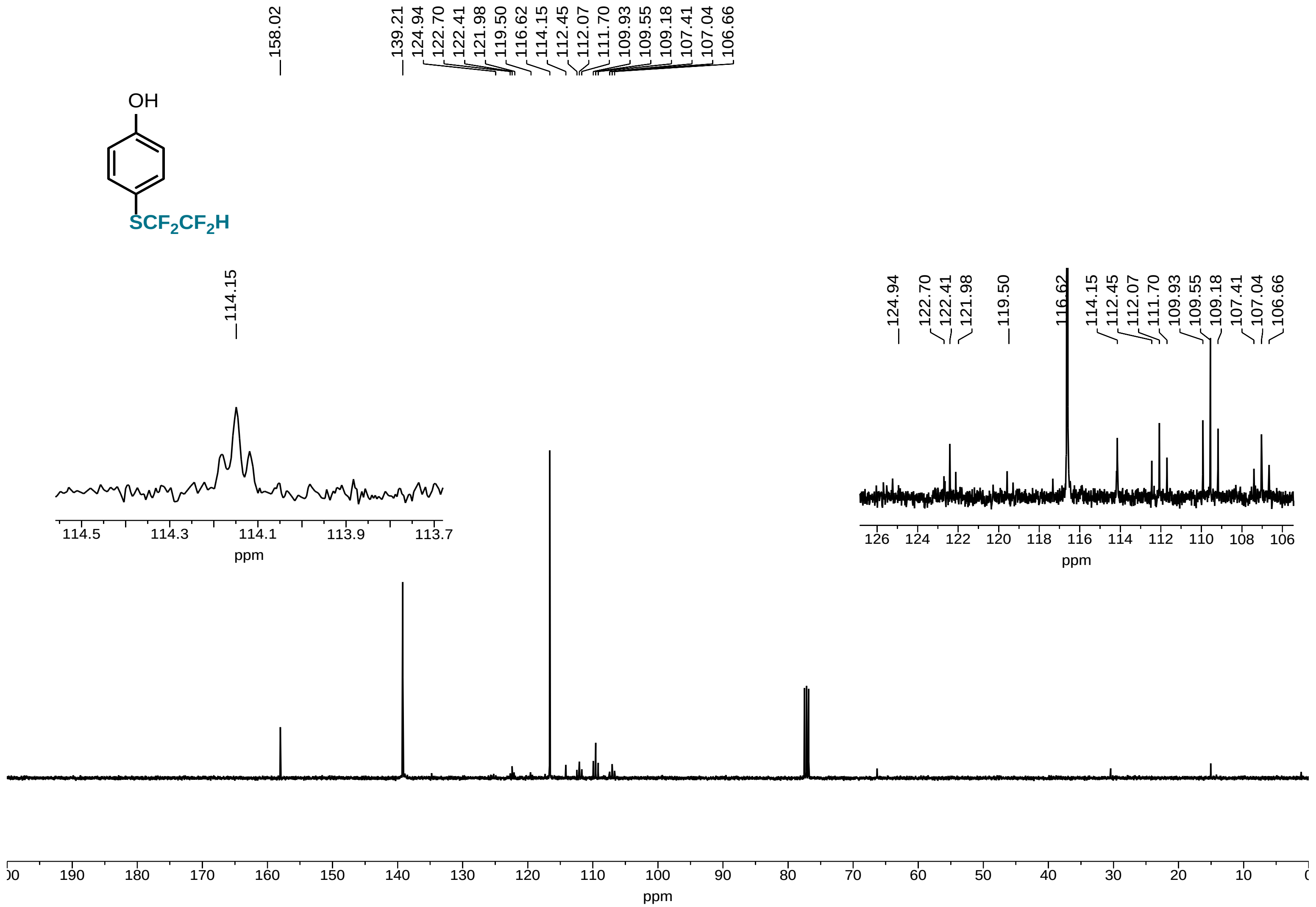

Figure S47. $^{13}C\{^{1}H\}$ NMR ($CDCl_3$, 100.6 MHz) of **10a**

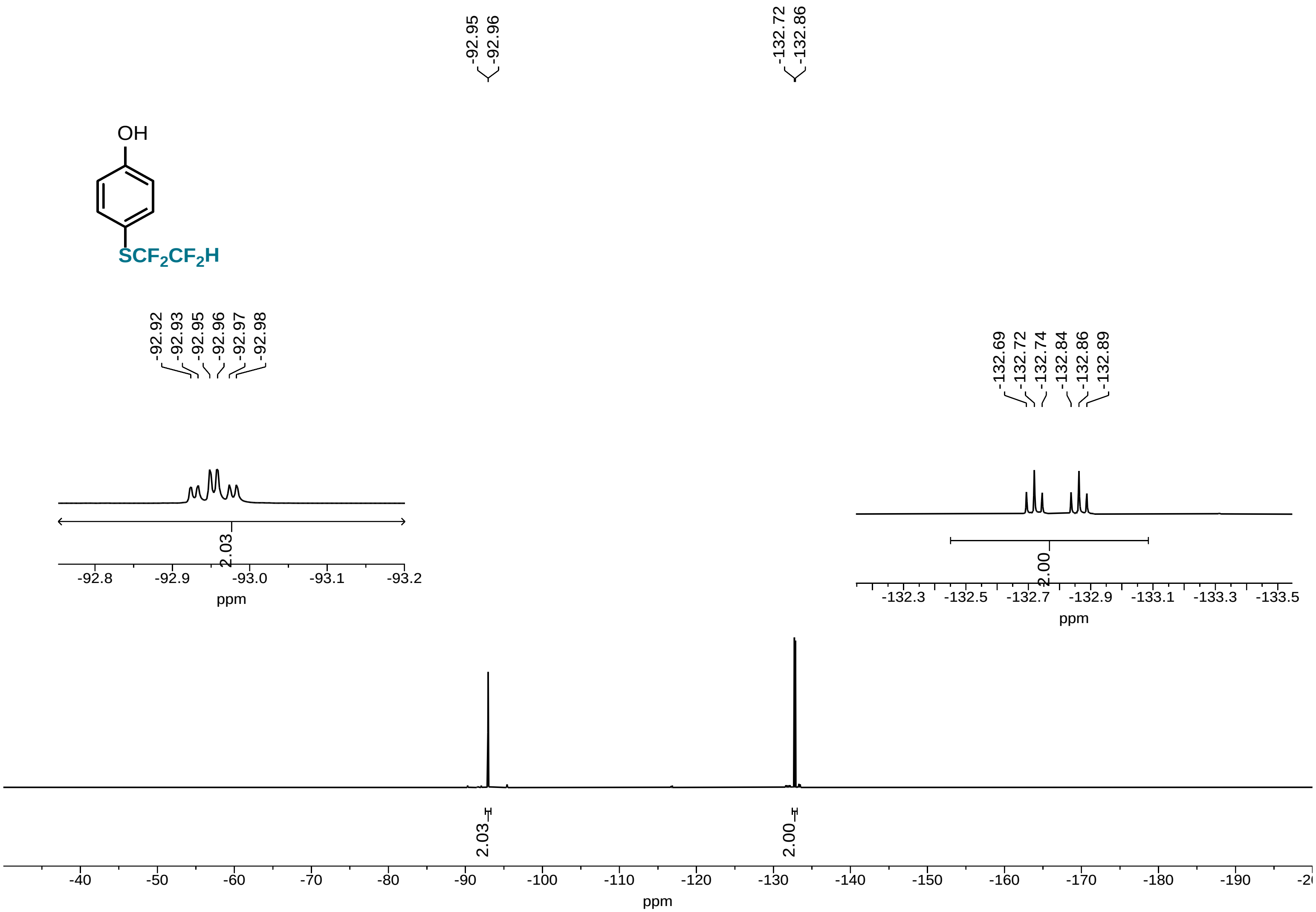

Figure S48. ^{19}F NMR ($CDCl_3$, 376.5 MHz) of **10a**

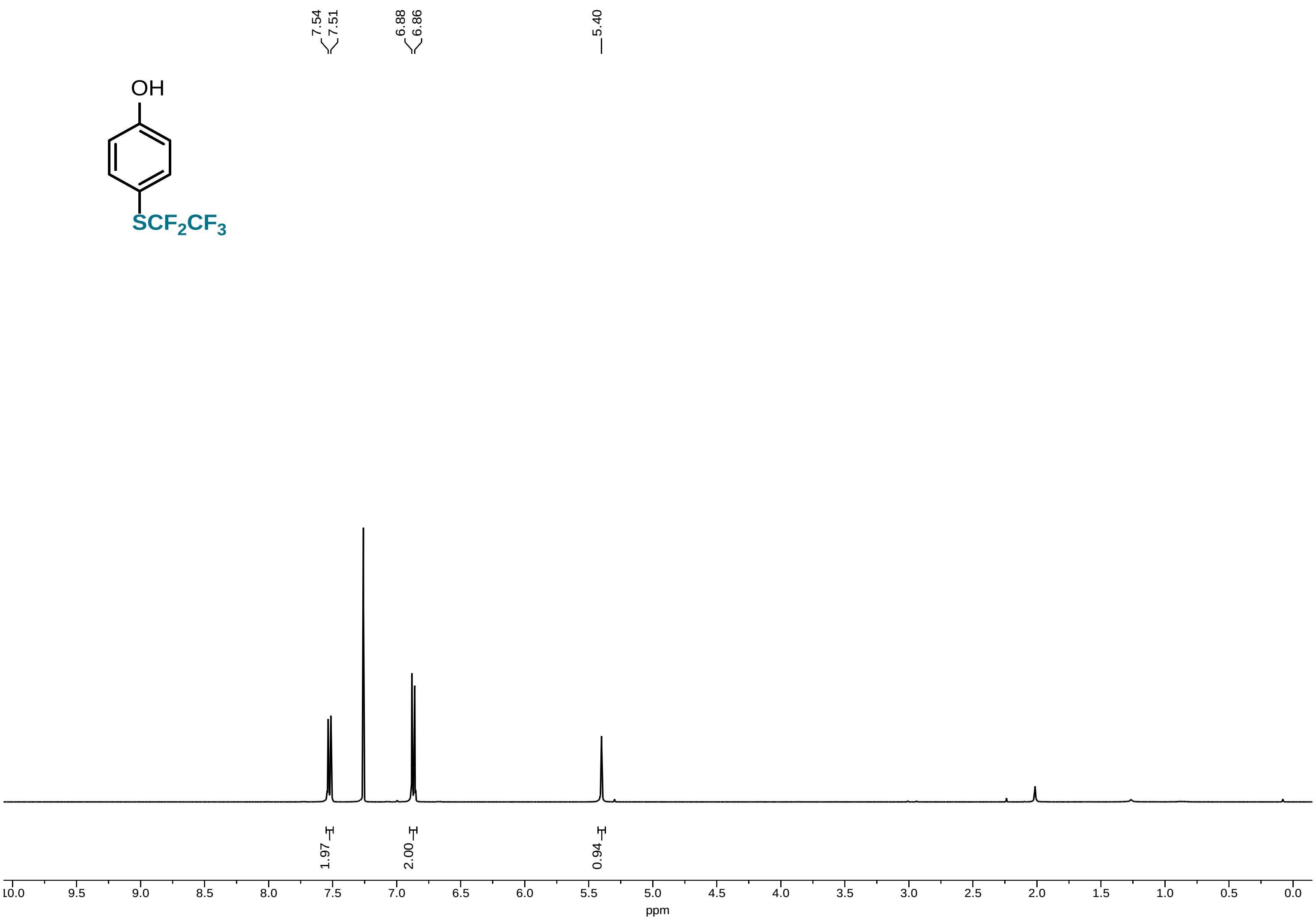

Figure S49. ^{1}H NMR ($CDCl_3$, 400 MHz) of **10b**

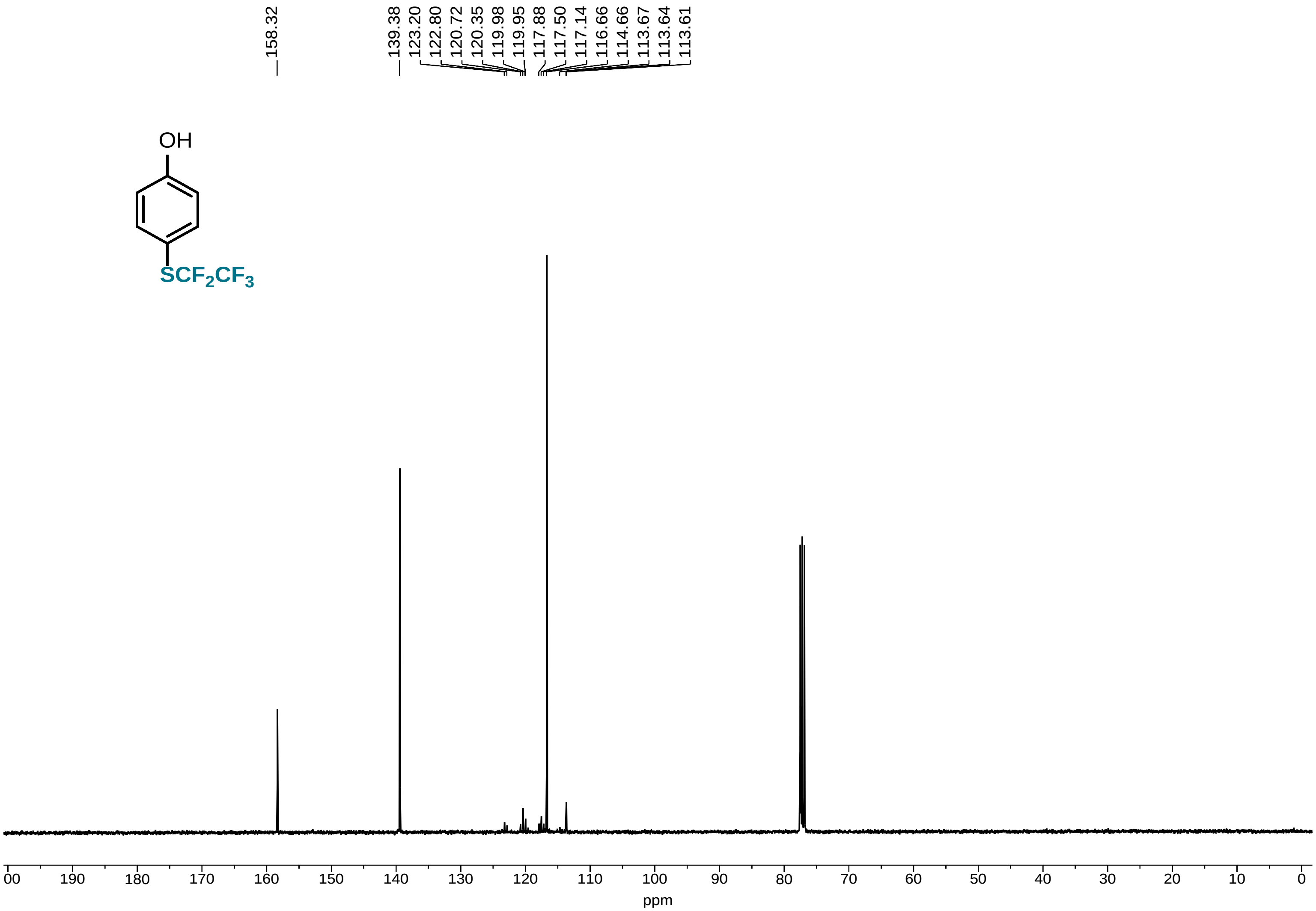

Figure S50. $^{13}C\{^{1}H\}$ NMR ($CDCl_3$, 100.6 MHz) of **10b**

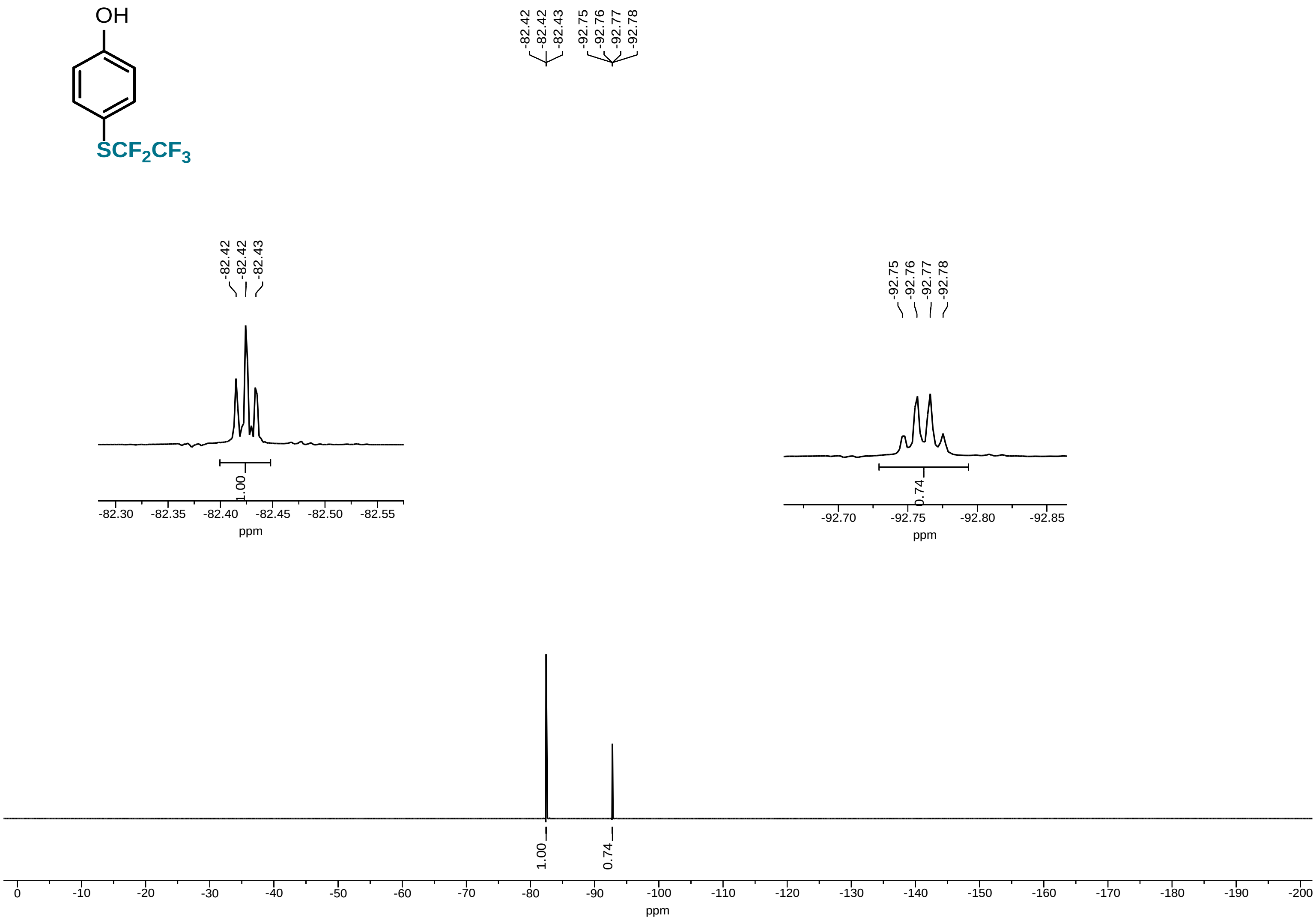

Figure S51. ^{19}F NMR ($CDCl_3$, 376.5 MHz) of **10b**

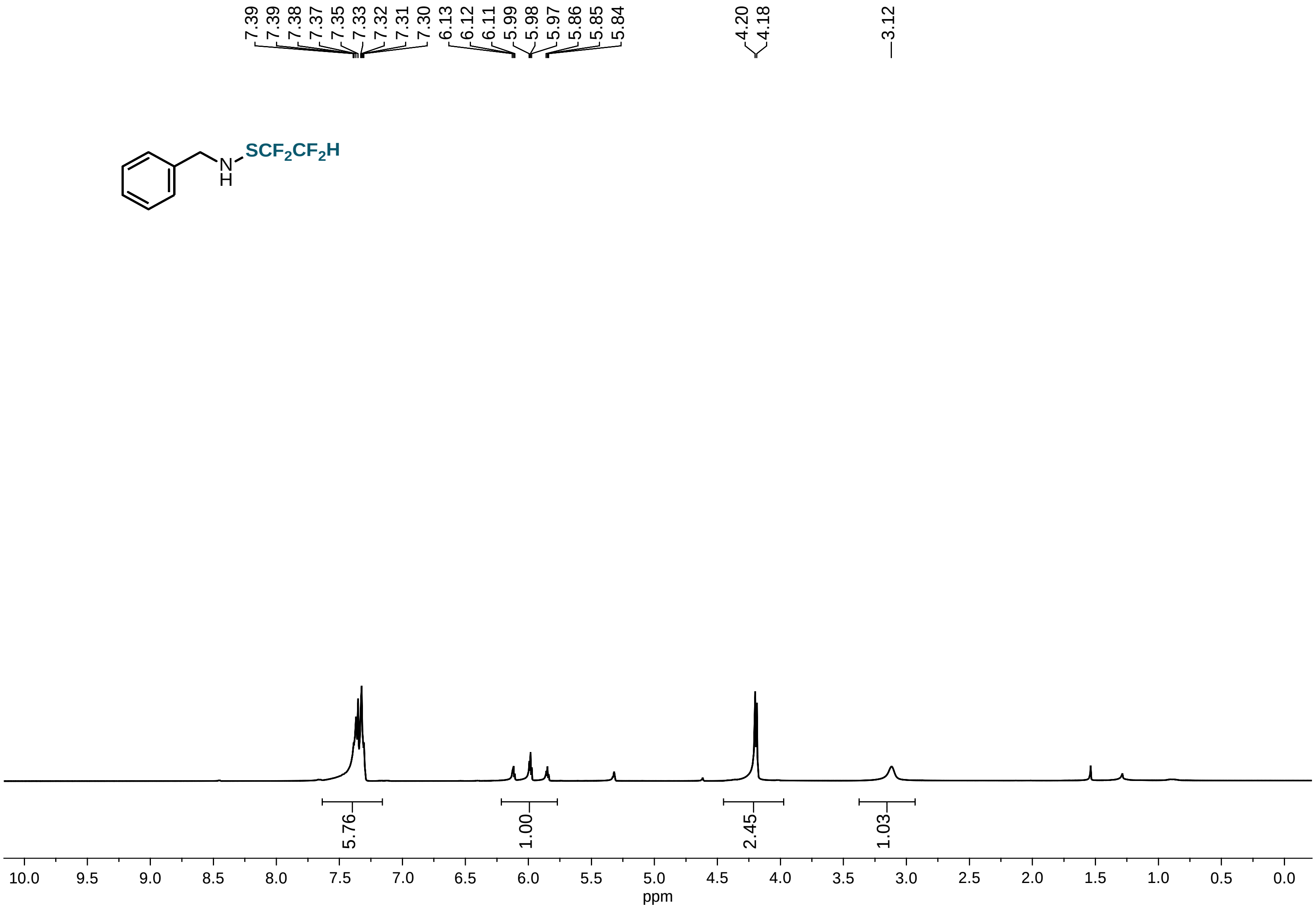

Figure S52. ^{1}H NMR (CD_2Cl_2, 400 MHz) of **11a**

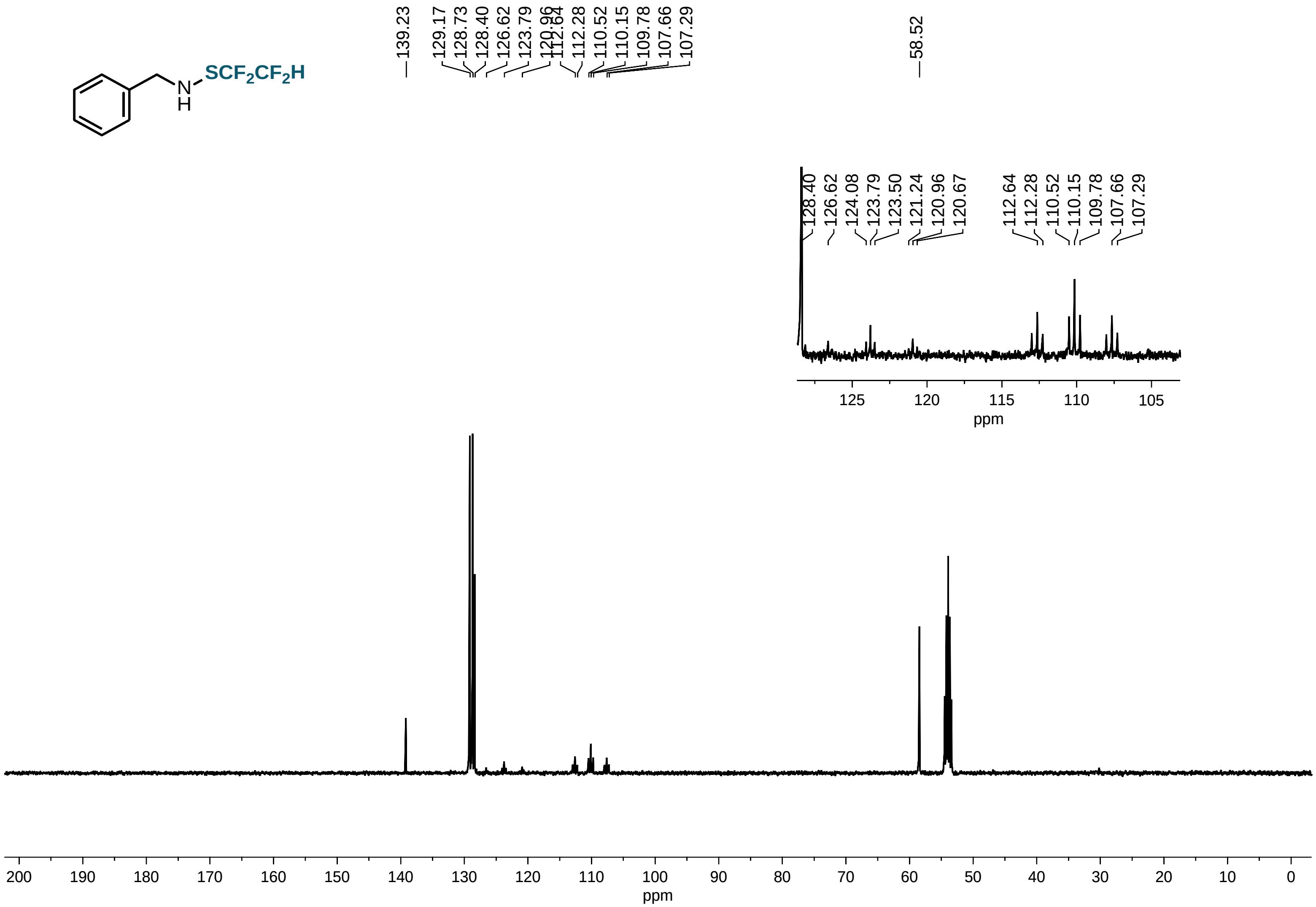

Figure S53. $^{13}C\{^{1}H\}$ NMR (CD_2Cl_2, 100.6 MHz) of **11a**

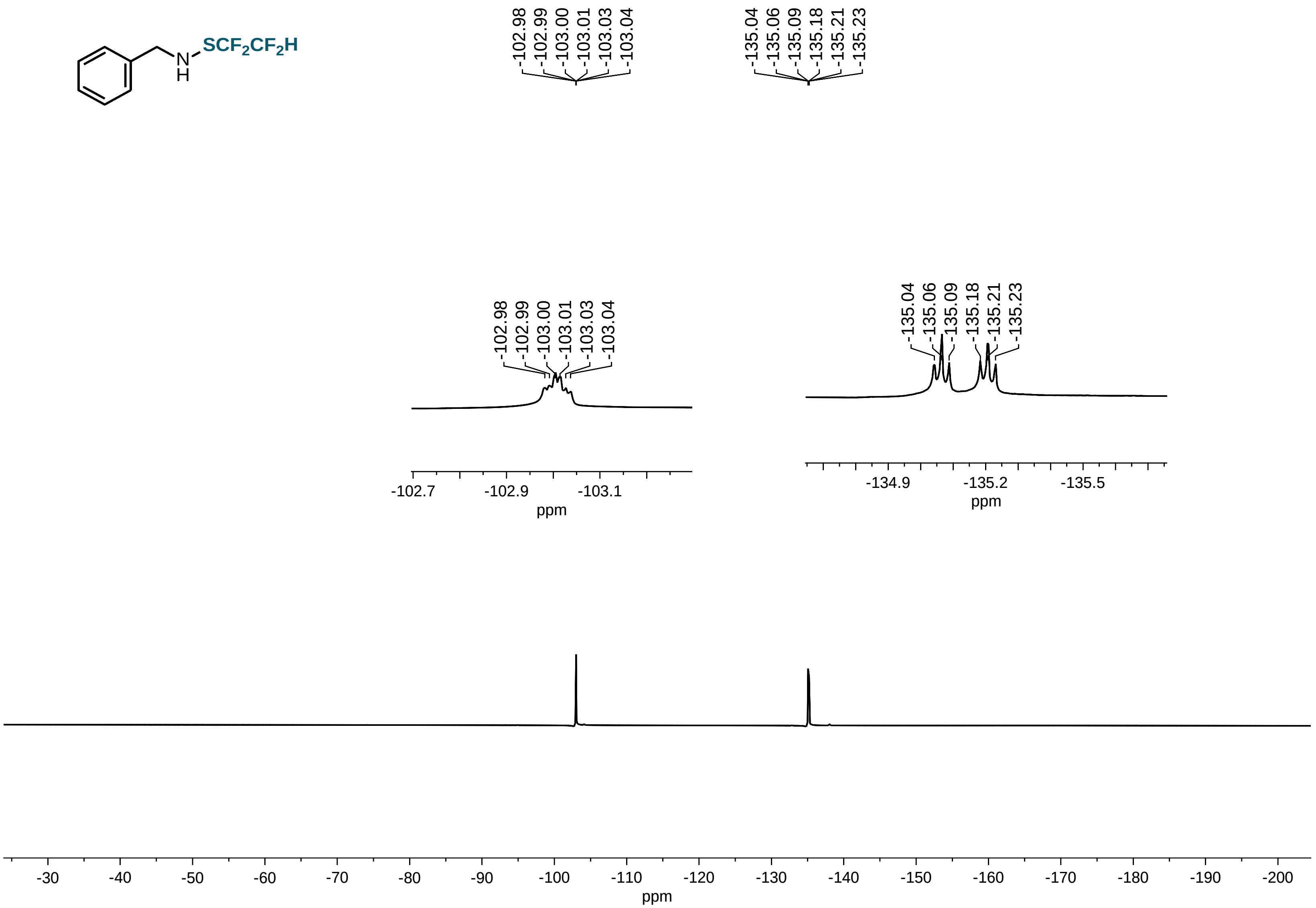

Figure S54. ^{19}F NMR (CD_2Cl_2, 376.5 MHz) of **11a**

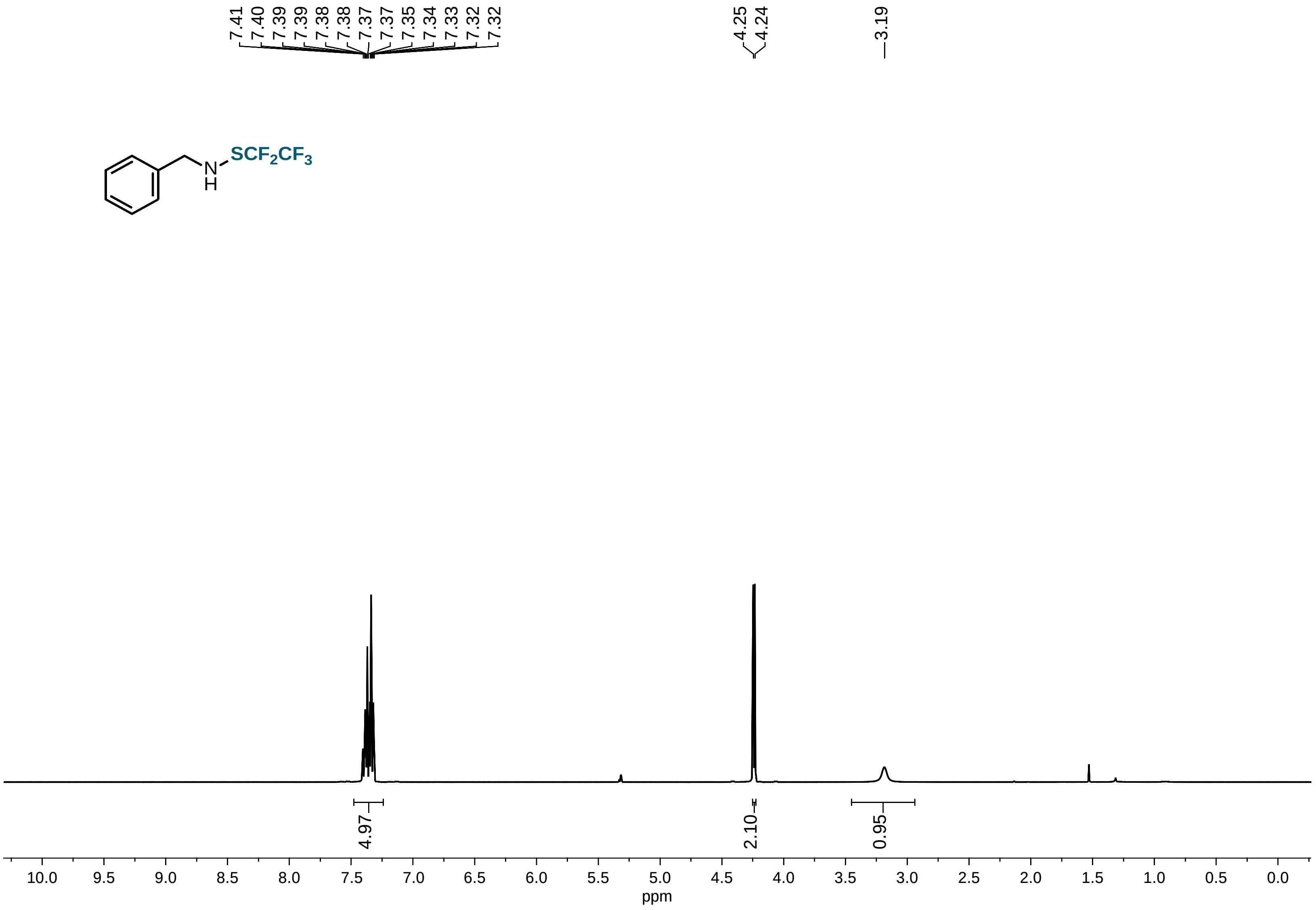

Figure S55. ^{1}H NMR (CD_2Cl_2, 400 MHz) of **11b**

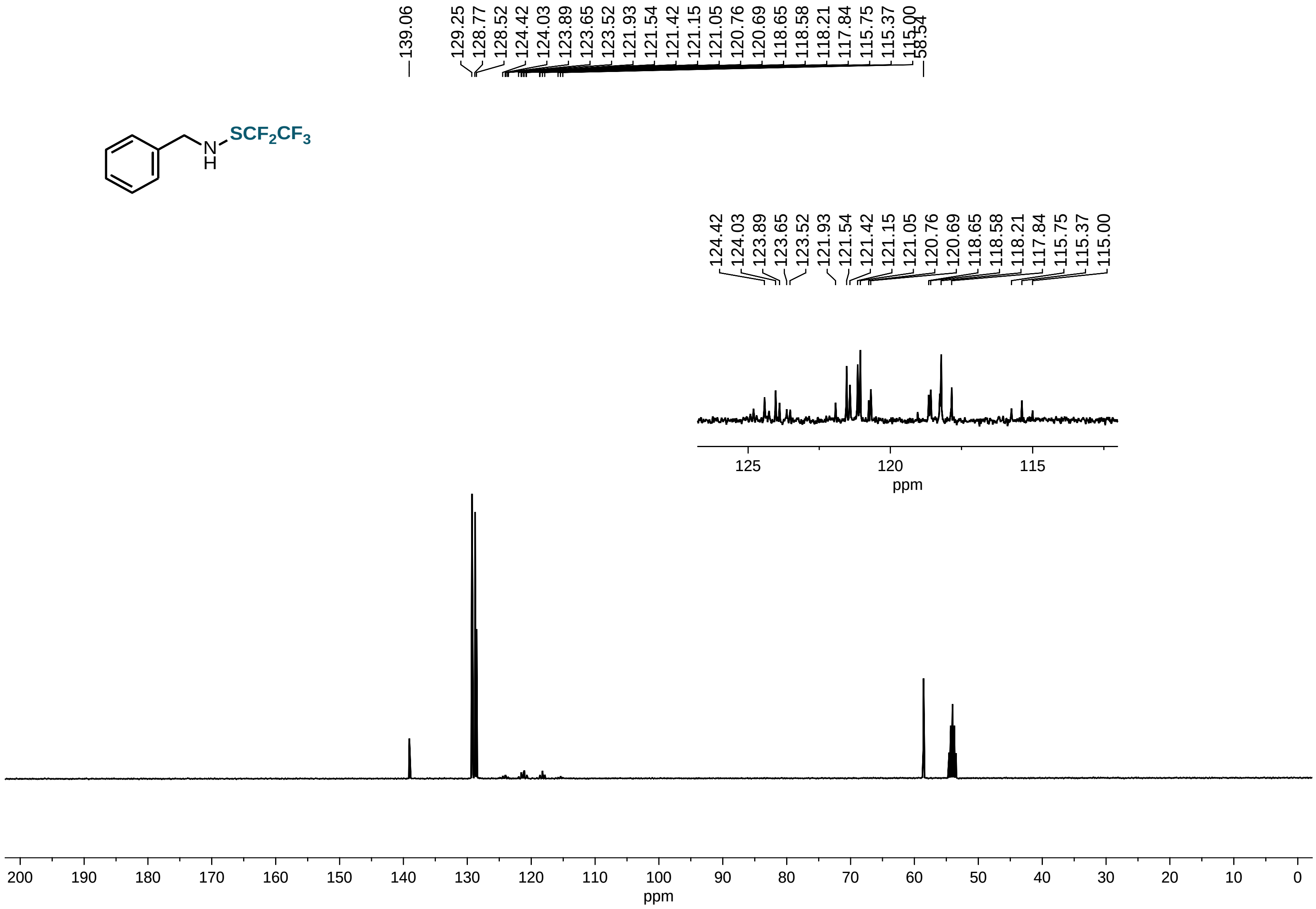

Figure S56. $^{13}C\{^1H\}$ NMR (CD_2Cl_2, 100.6 MHz) of **11b**

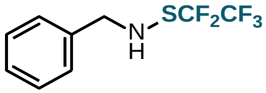

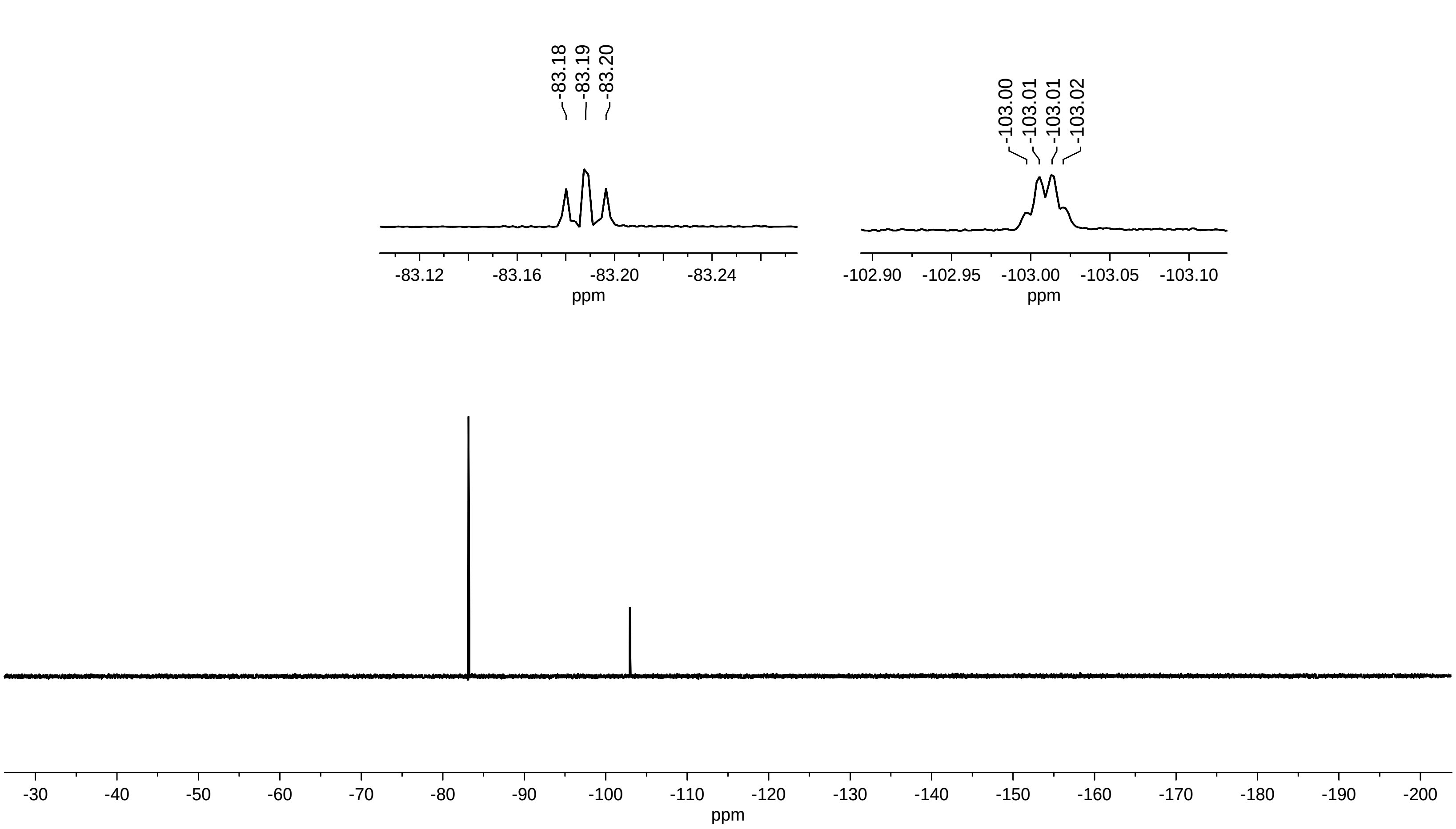

Figure S57. ^{19}F NMR (CD_2Cl_2, 376.5 MHz) of **11b**

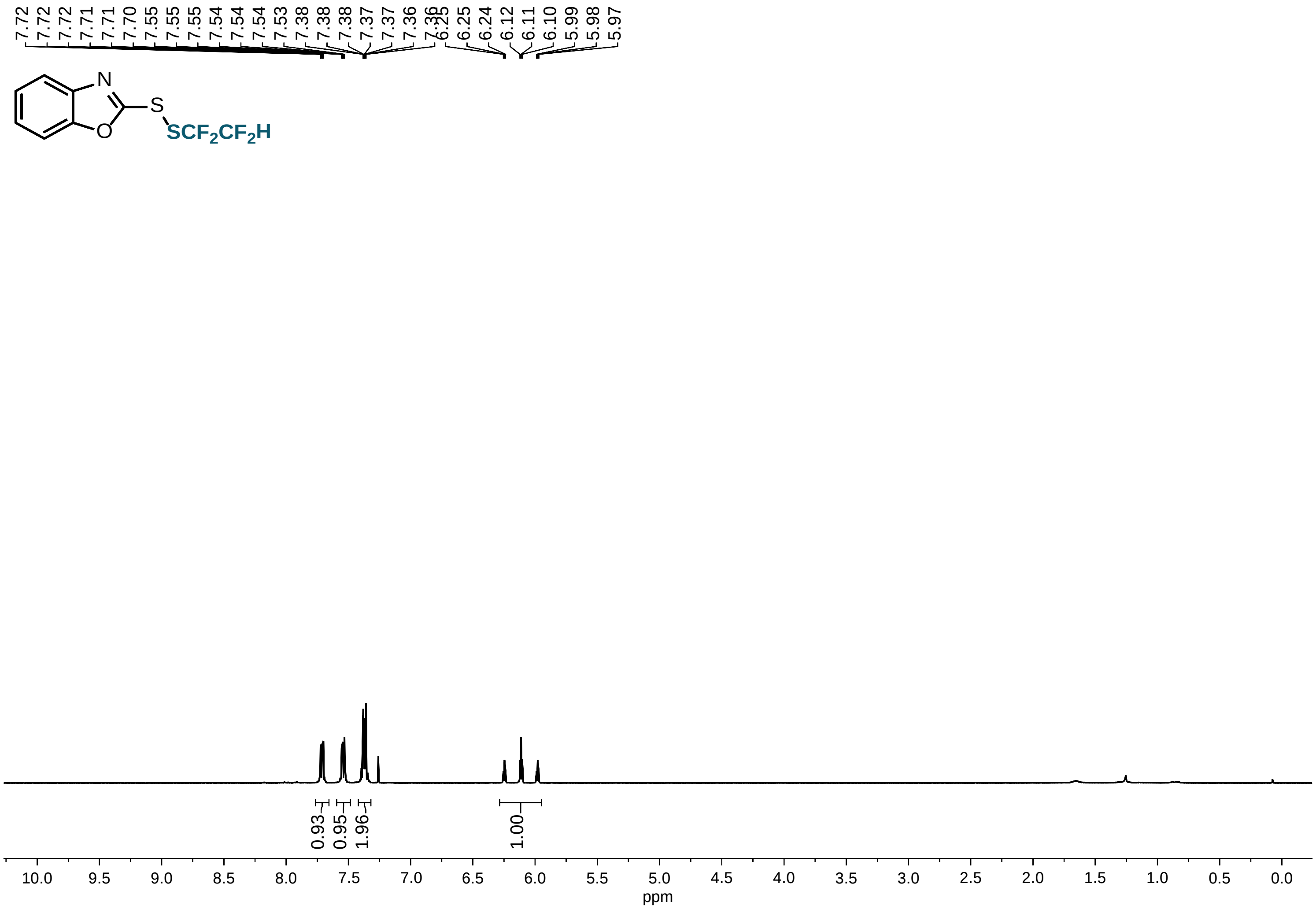

Figure S58. ^{1}H NMR ($CDCl_3$, 400 MHz) of **12a**

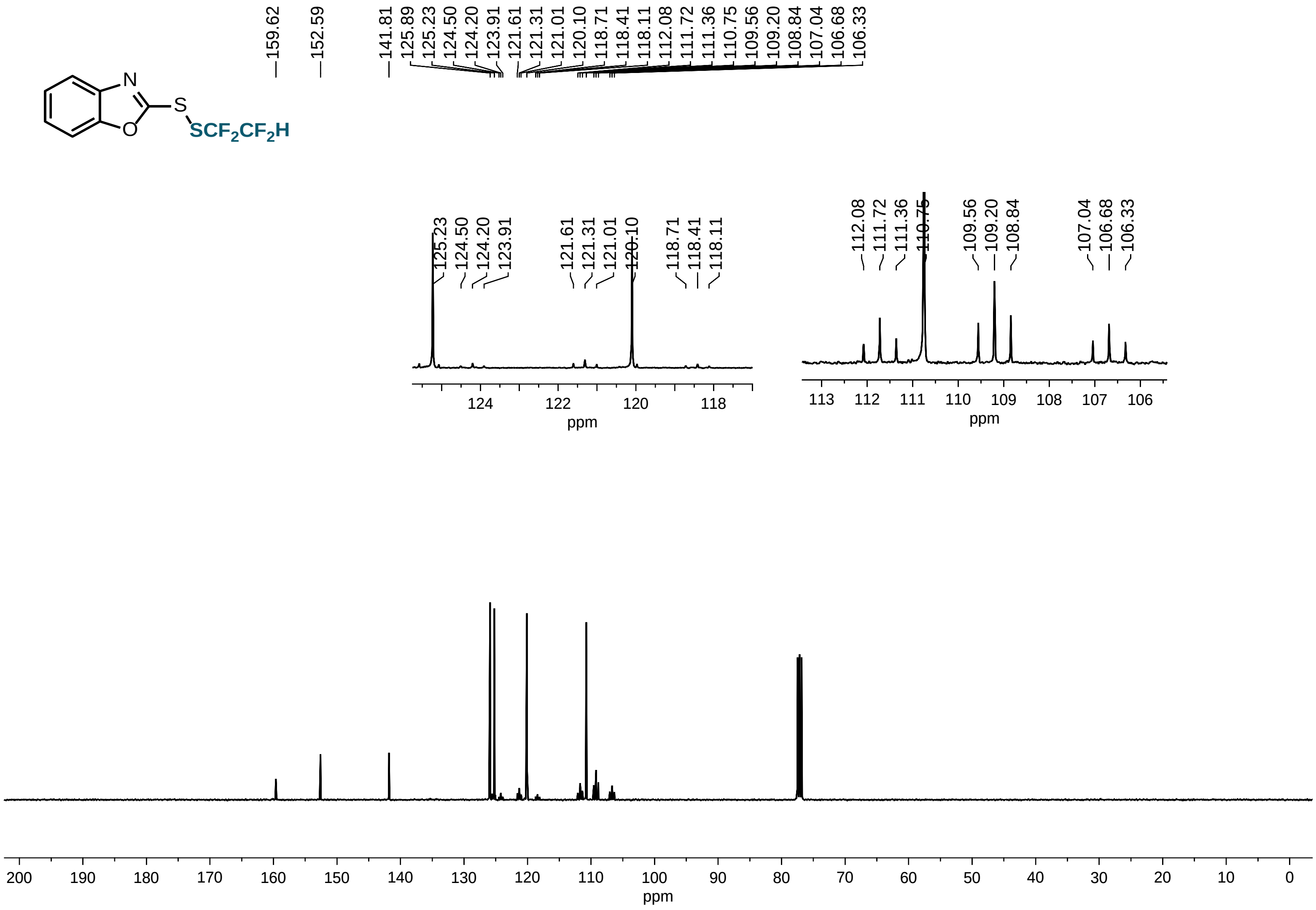

Figure S59. $^{13}C\{^{1}H\}$ NMR ($CDCl_3$, 100.6 MHz) of **12a**

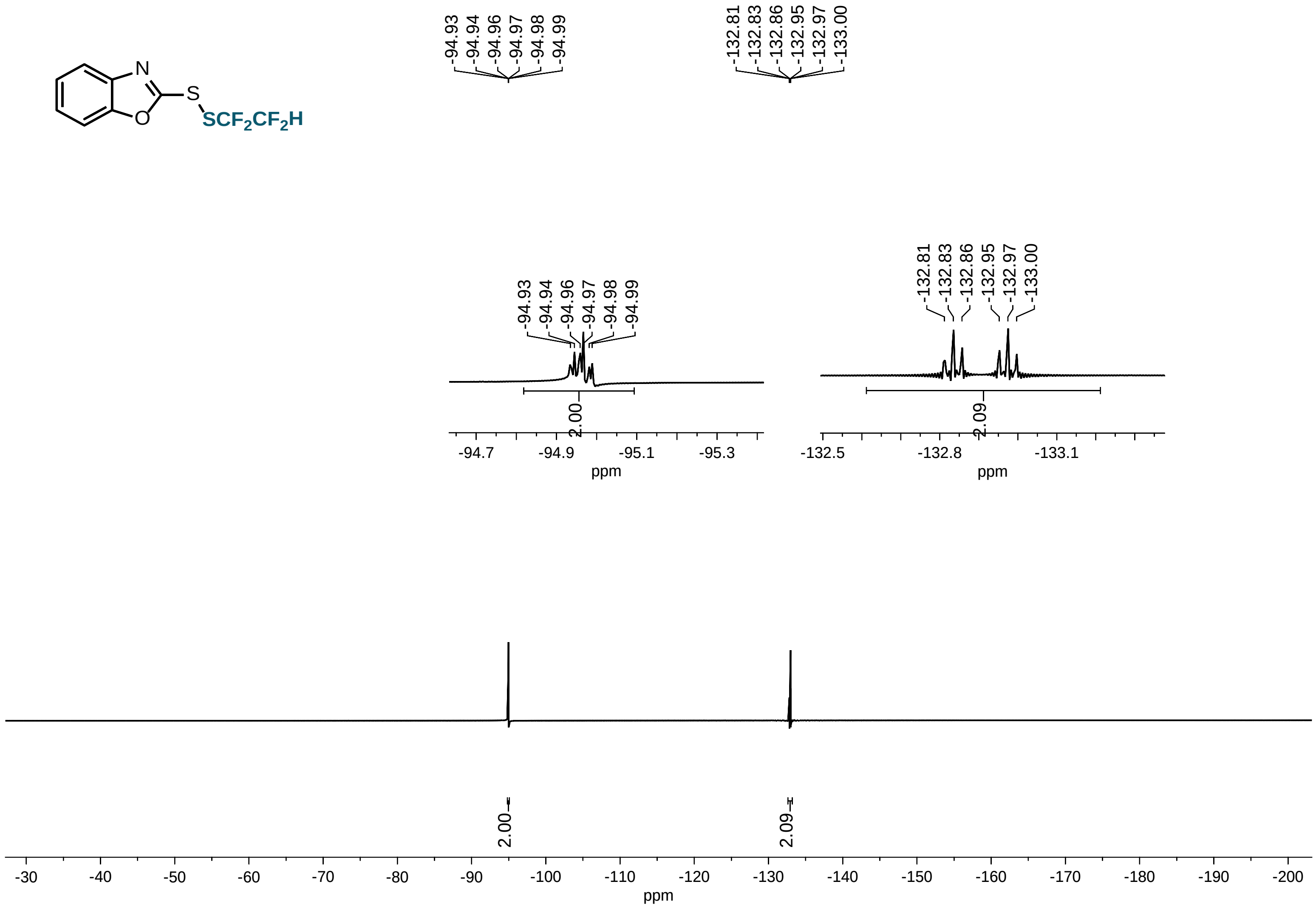

Figure S60. ^{19}F NMR ($CDCl_3$, 376.5 MHz) of **12a**

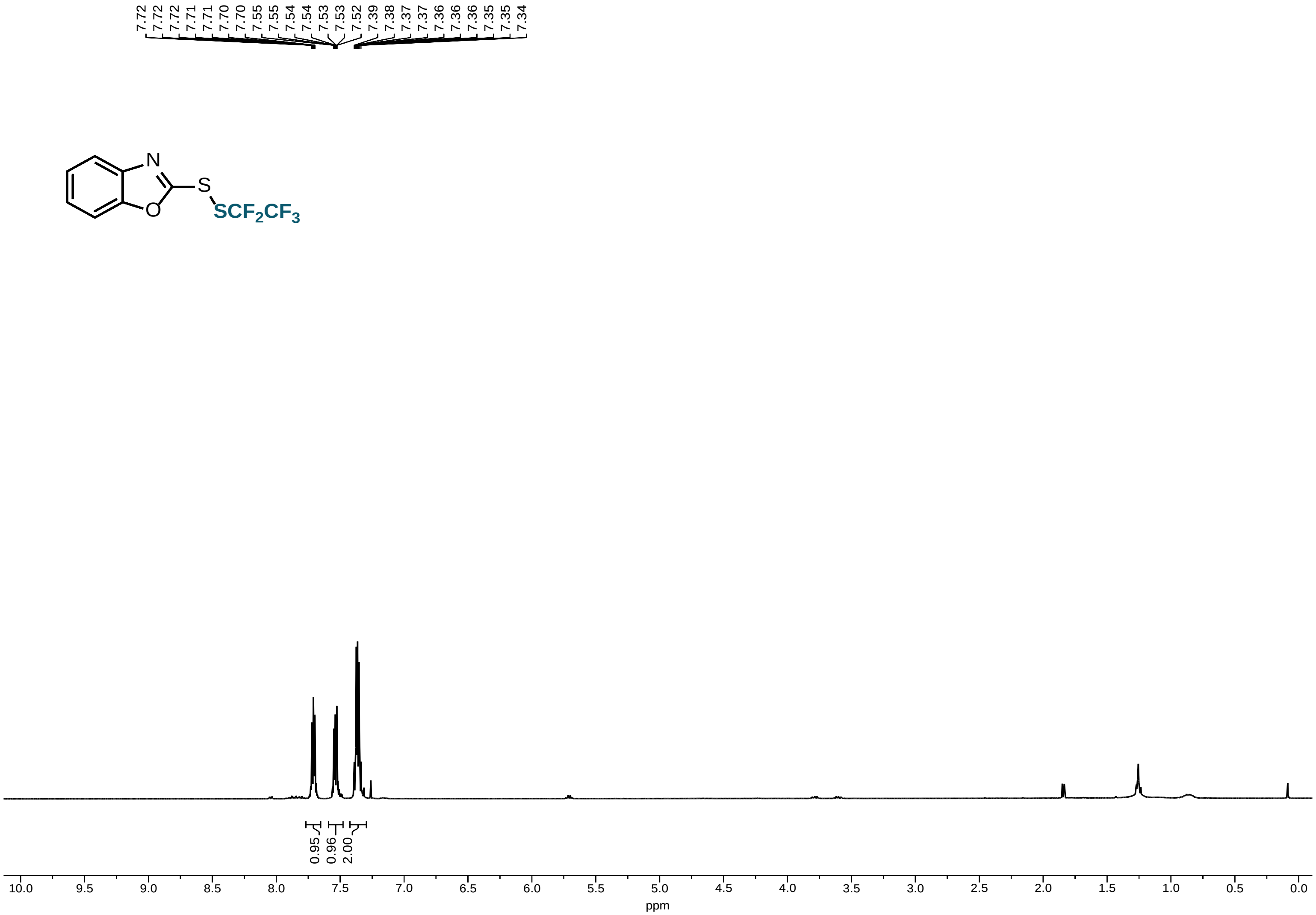

Figure S61. ^{1}H NMR ($CDCl_3$, 400 MHz) of **12b**

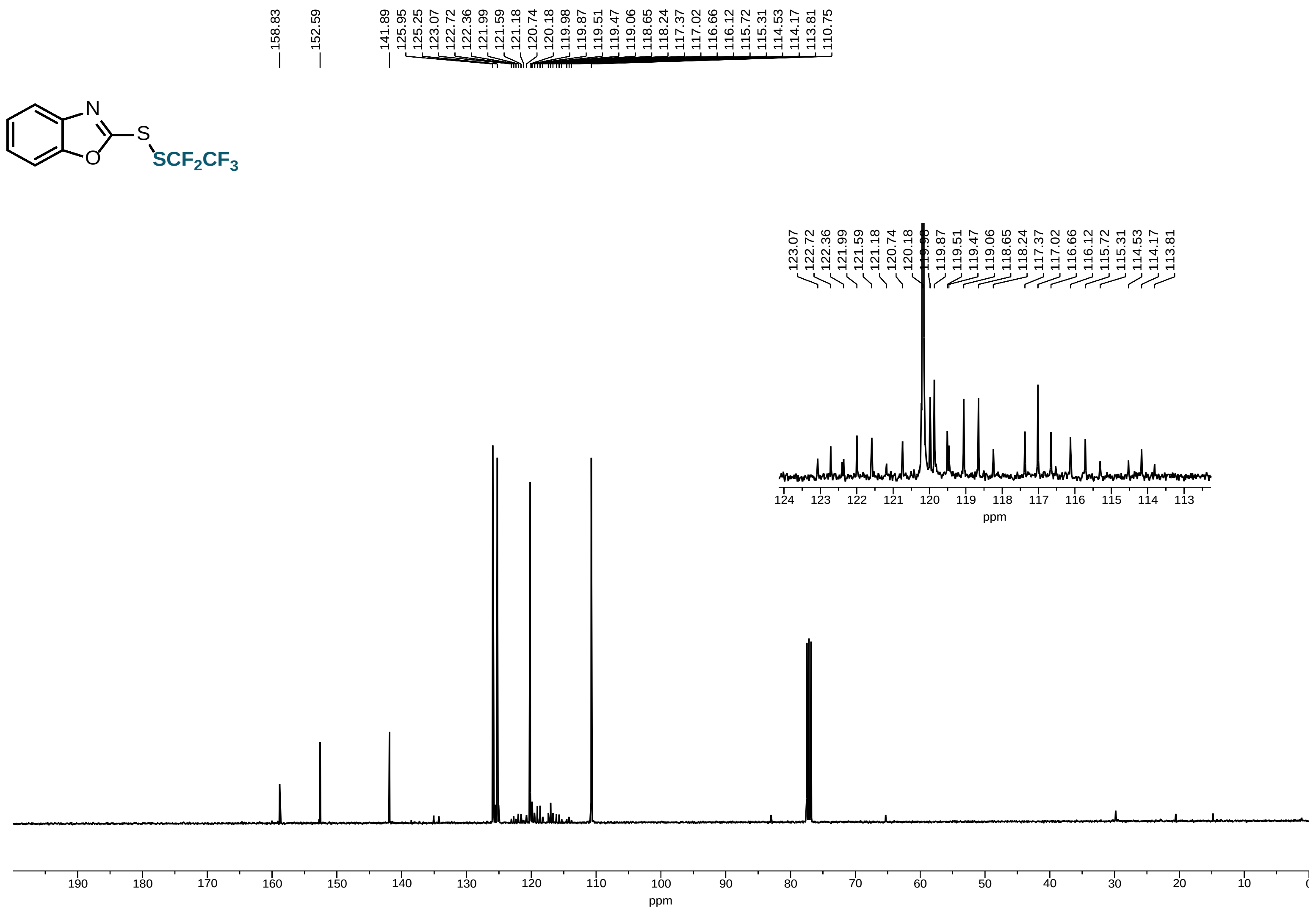

Figure S62. $^{13}C\{^{1}H\}$ NMR ($CDCl_3$, 100.6 MHz) of **12b**

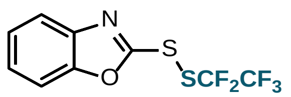

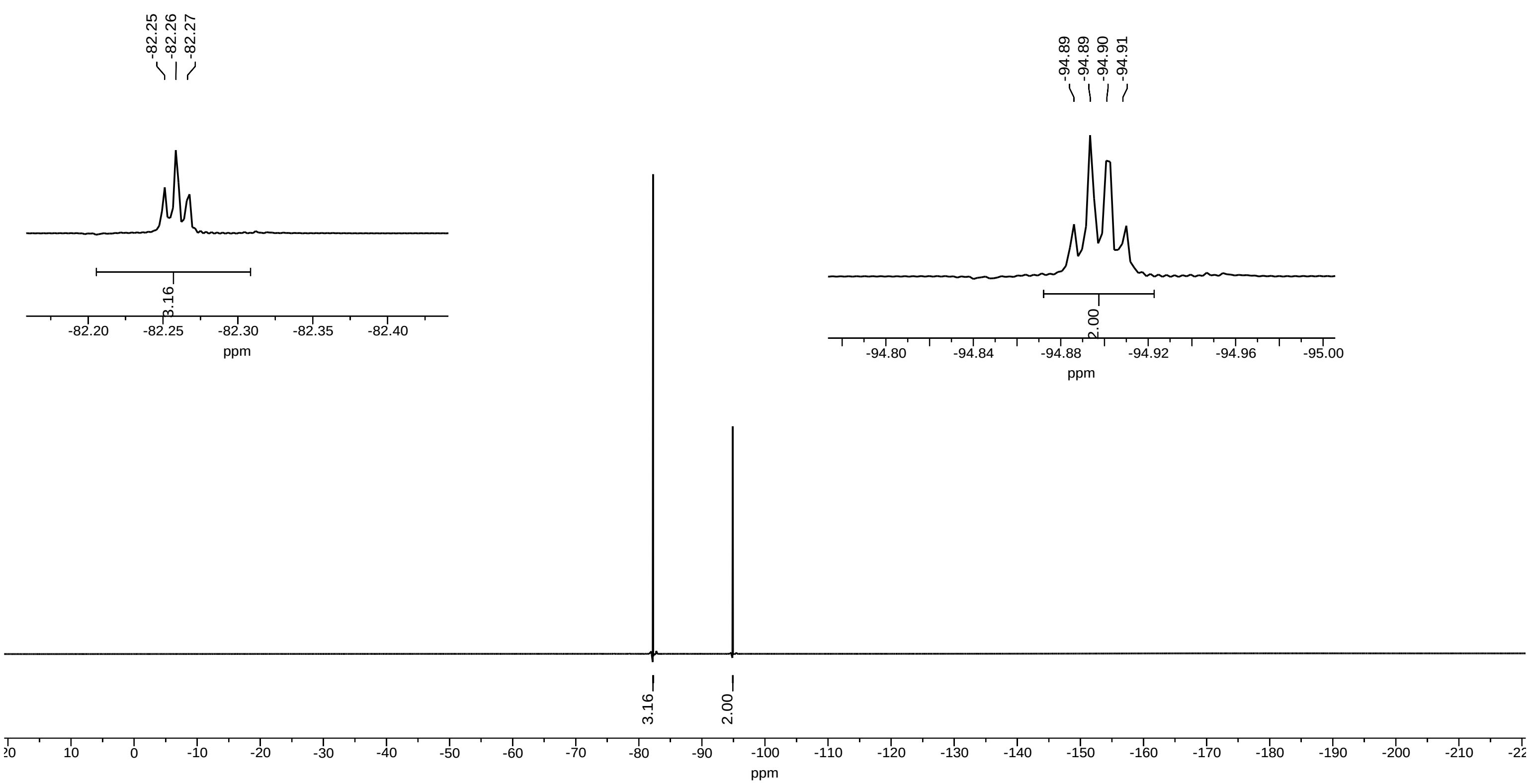

Figure S63. ^{19}F NMR ($CDCl_3$, 376.5 MHz) of **12b**

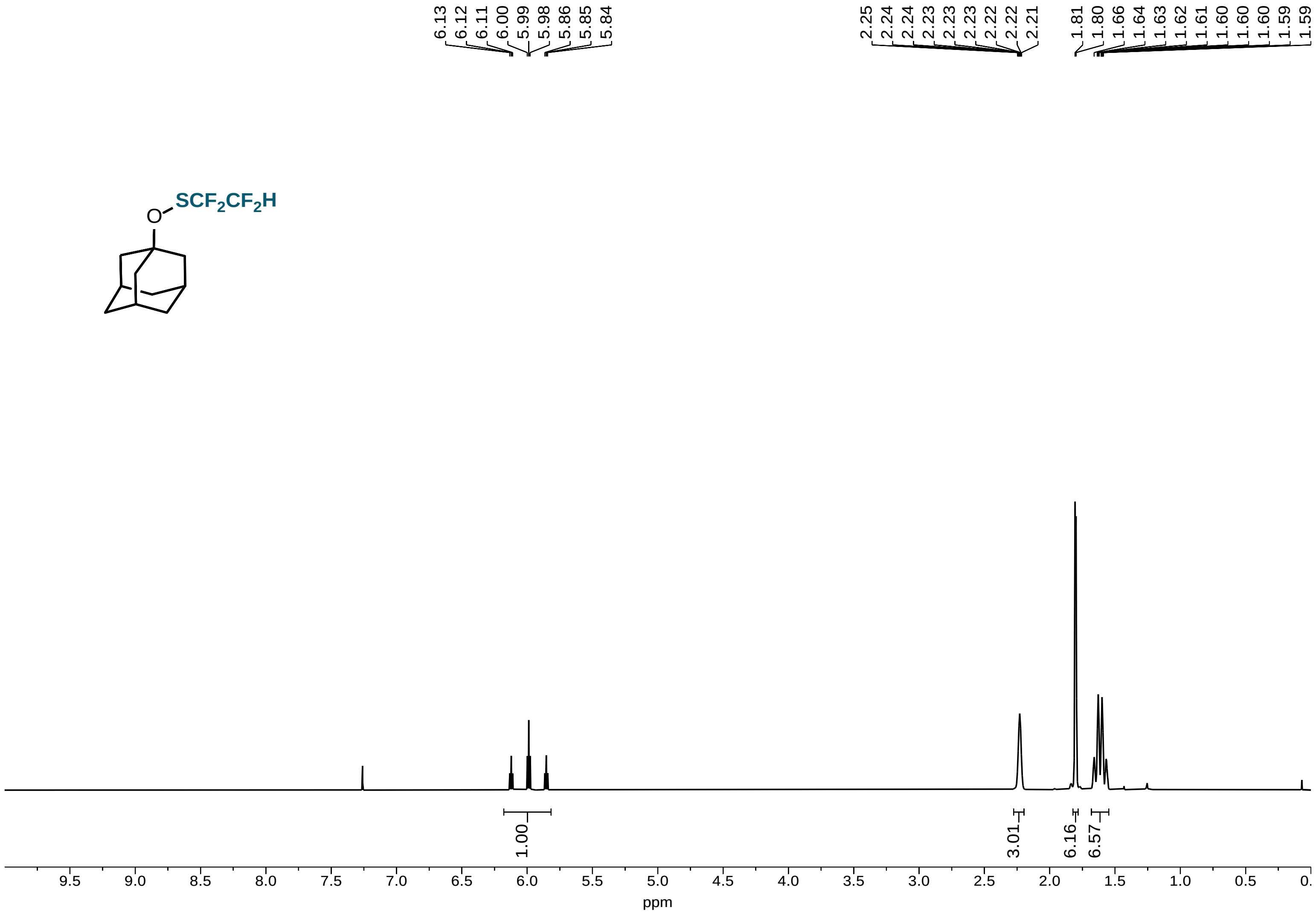

Figure S64. ^{1}H NMR ($CDCl_3$, 400 MHz) of **13a**

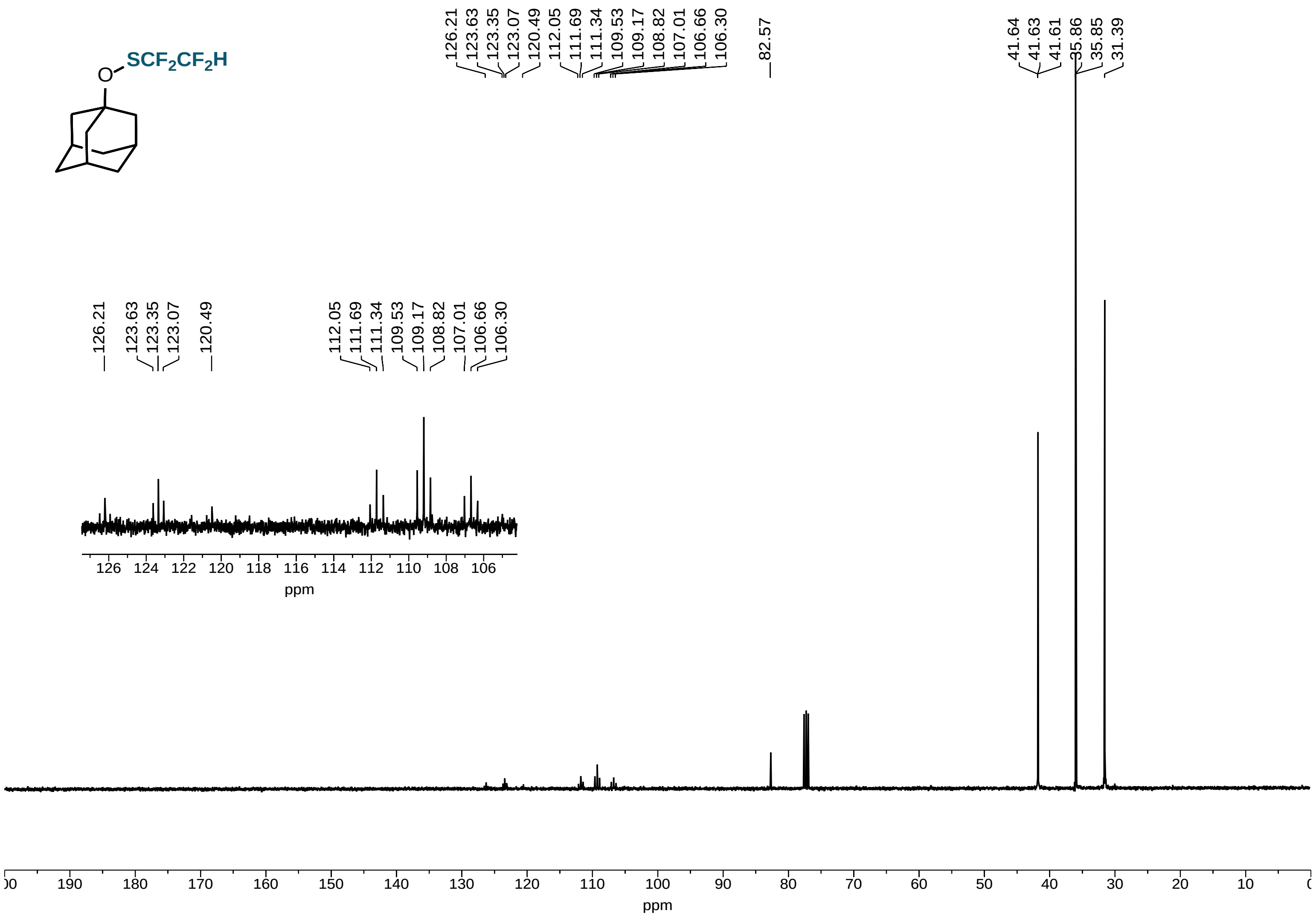

Figure S65. $^{13}C\{^1H\}$ NMR ($CDCl_3$, 100.6 MHz) of **13a**

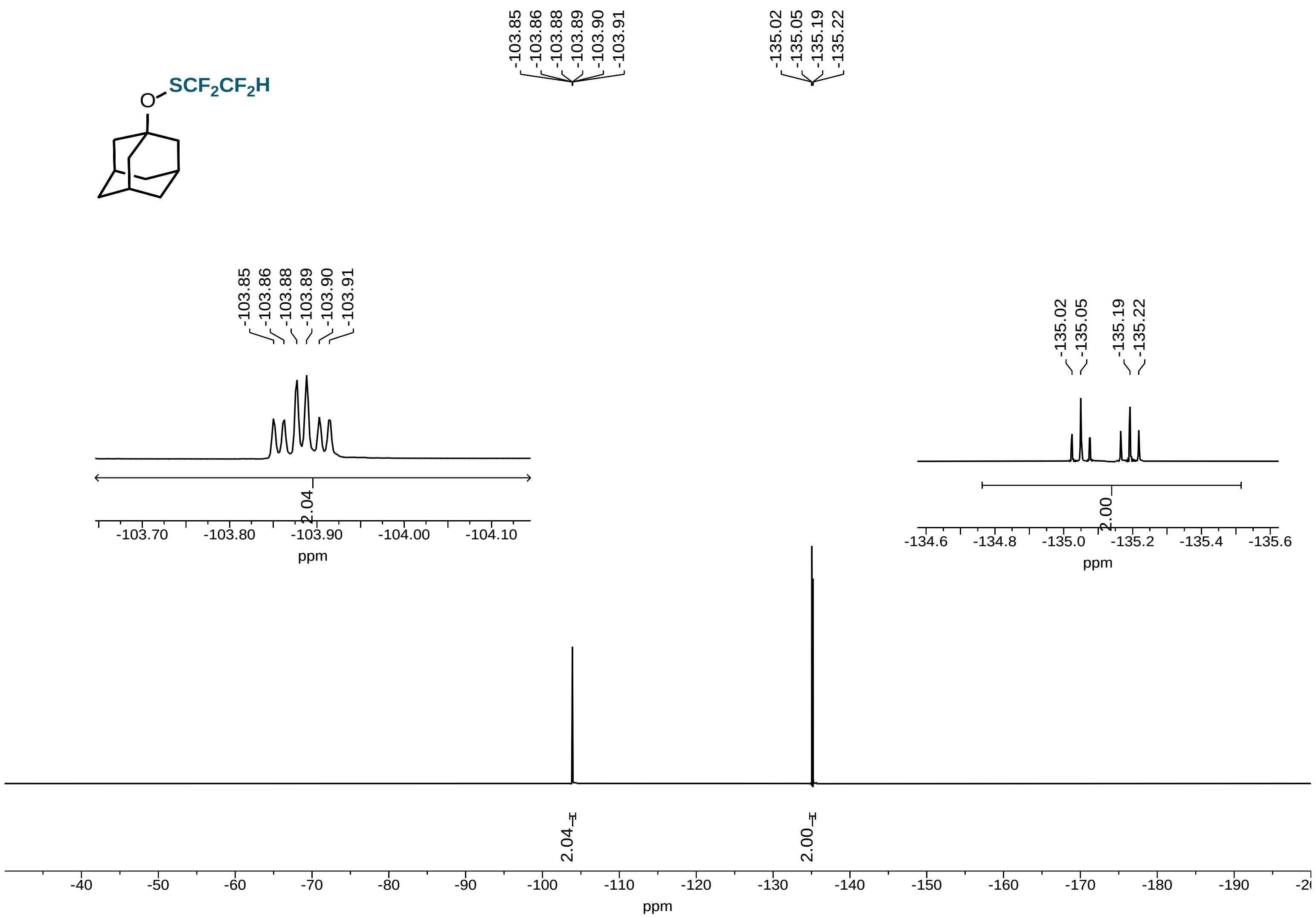

Figure S66. ^{19}F NMR ($CDCl_3$, 376.5 MHz) of **13a**

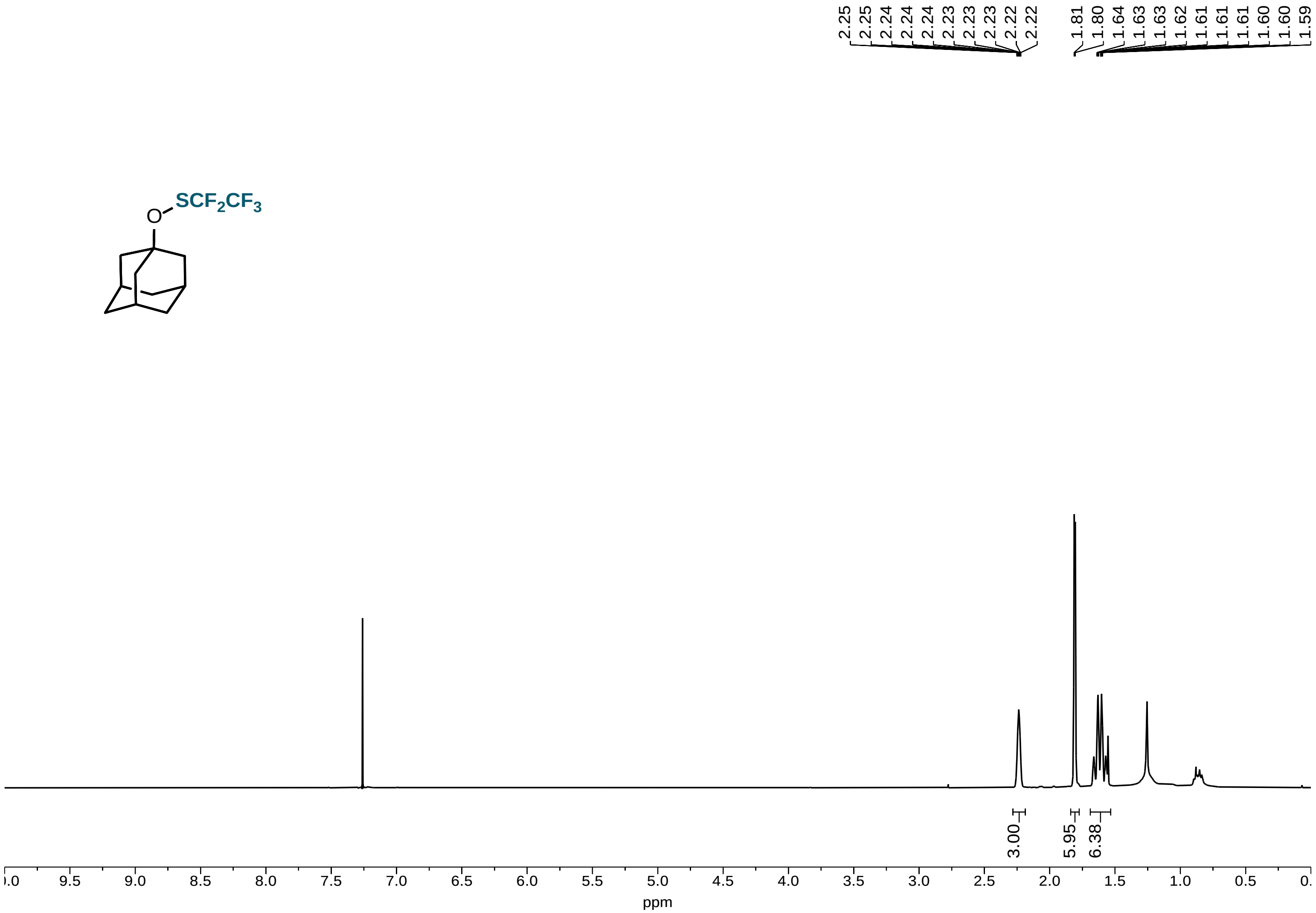

Figure S67. ^{1}H NMR ($CDCl_3$, 400 MHz) of **13b**

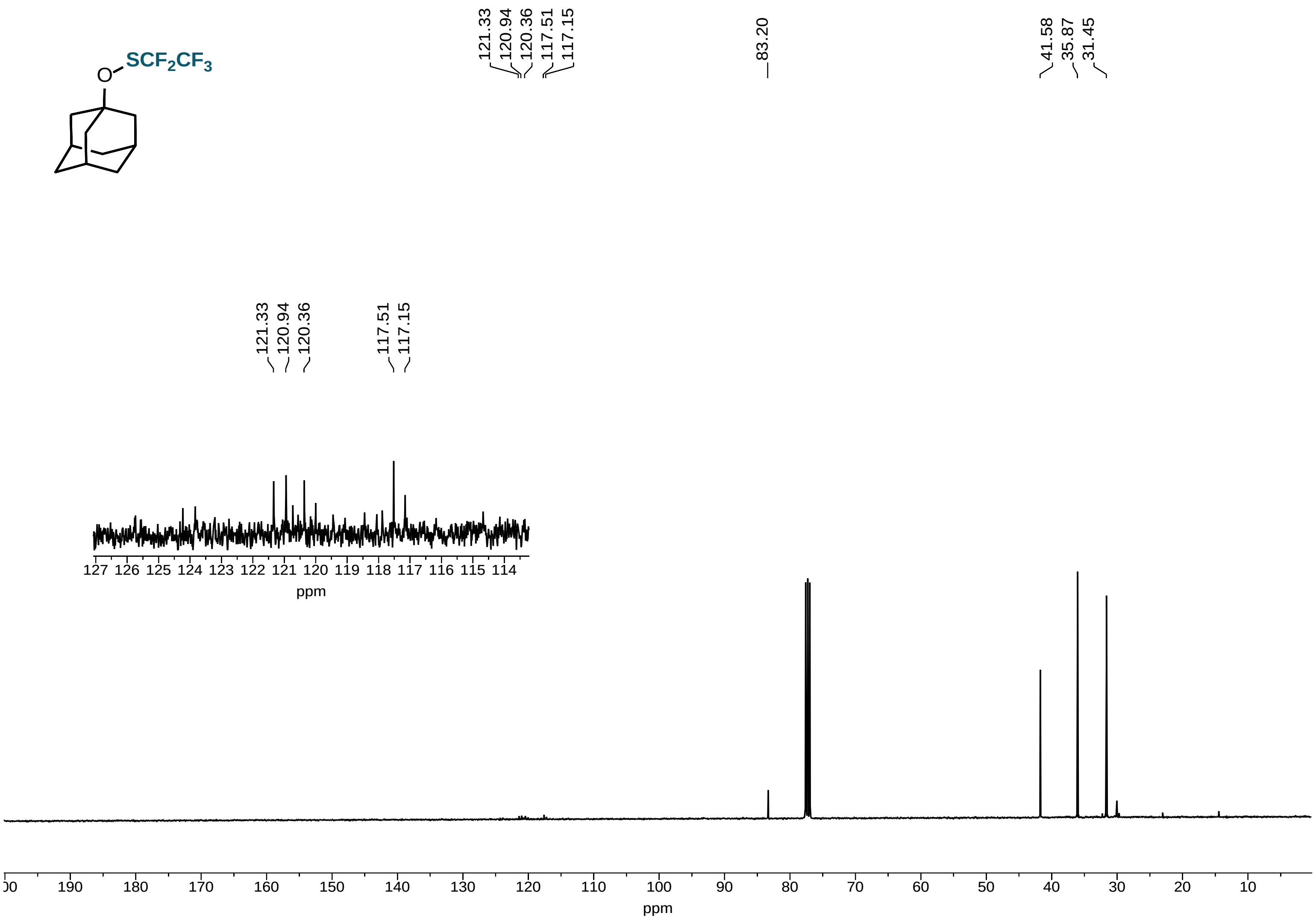

Figure S68. $^{13}C\{^1H\}$ NMR ($CDCl_3$, 100.6 MHz) of **13b**

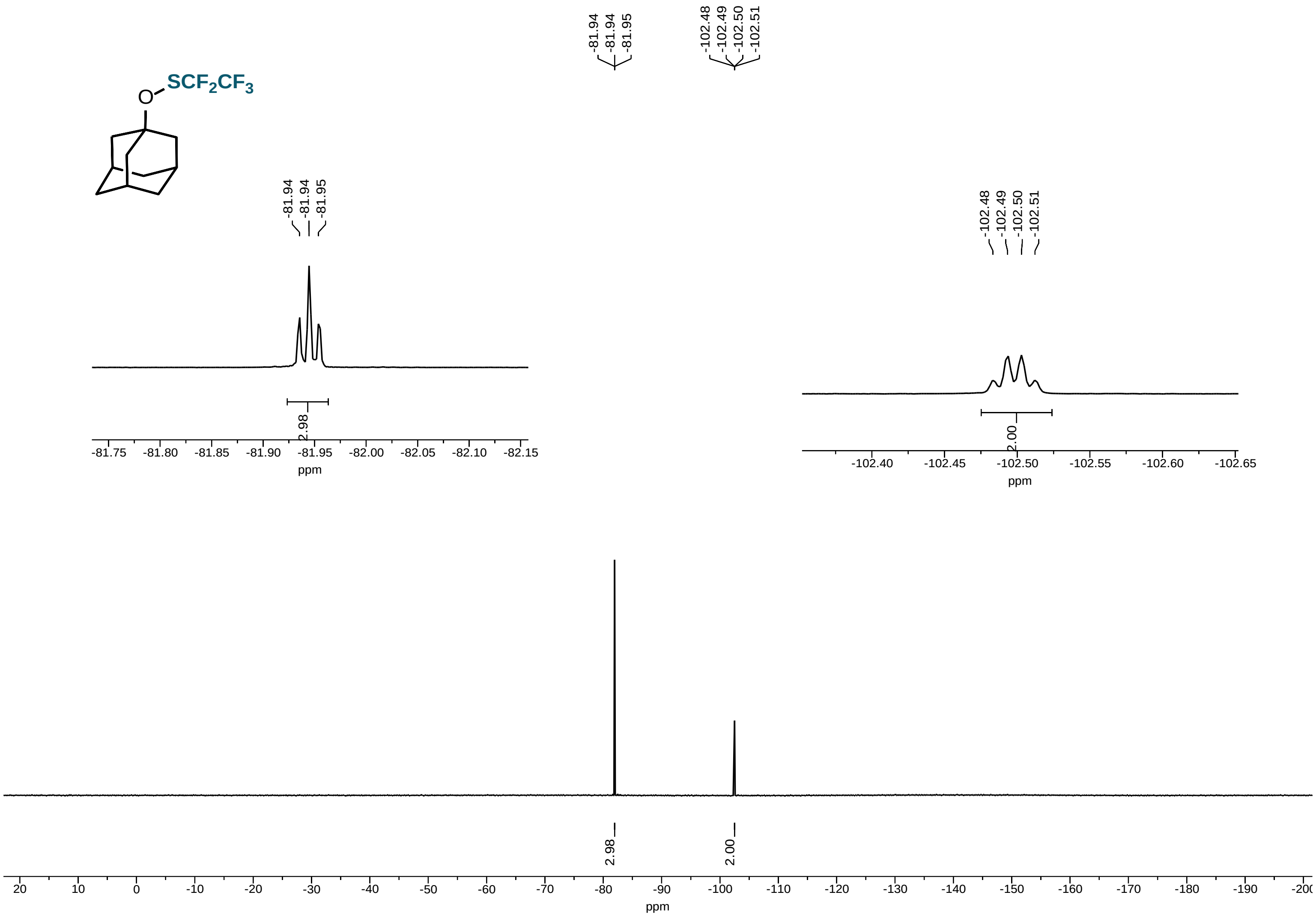

Figure S69. ^{19}F NMR ($CDCl_3$, 376.5 MHz) of **13b**

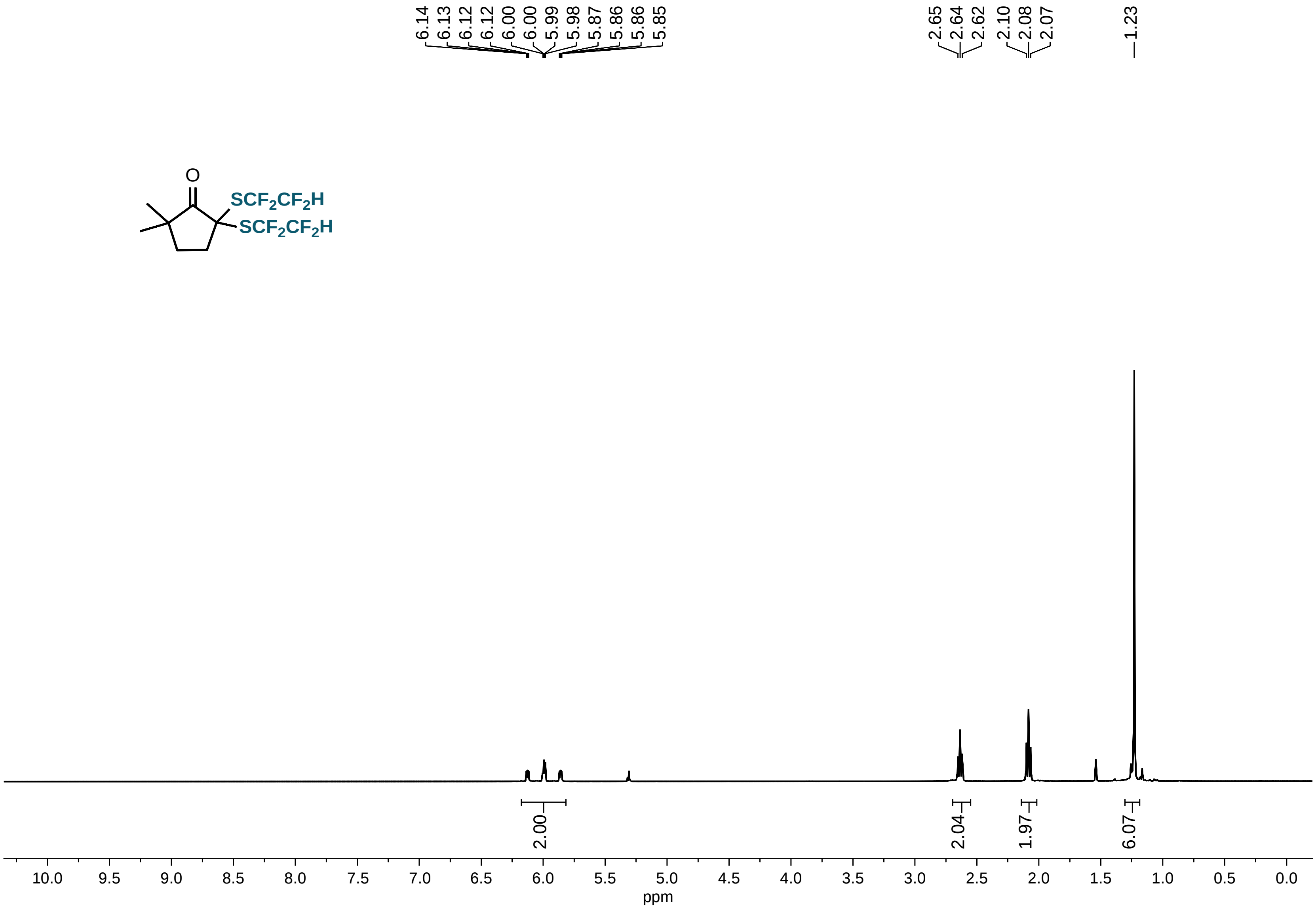

Figure S70. ^{1}H NMR (CD_2Cl_2, 400 MHz) of **14a**

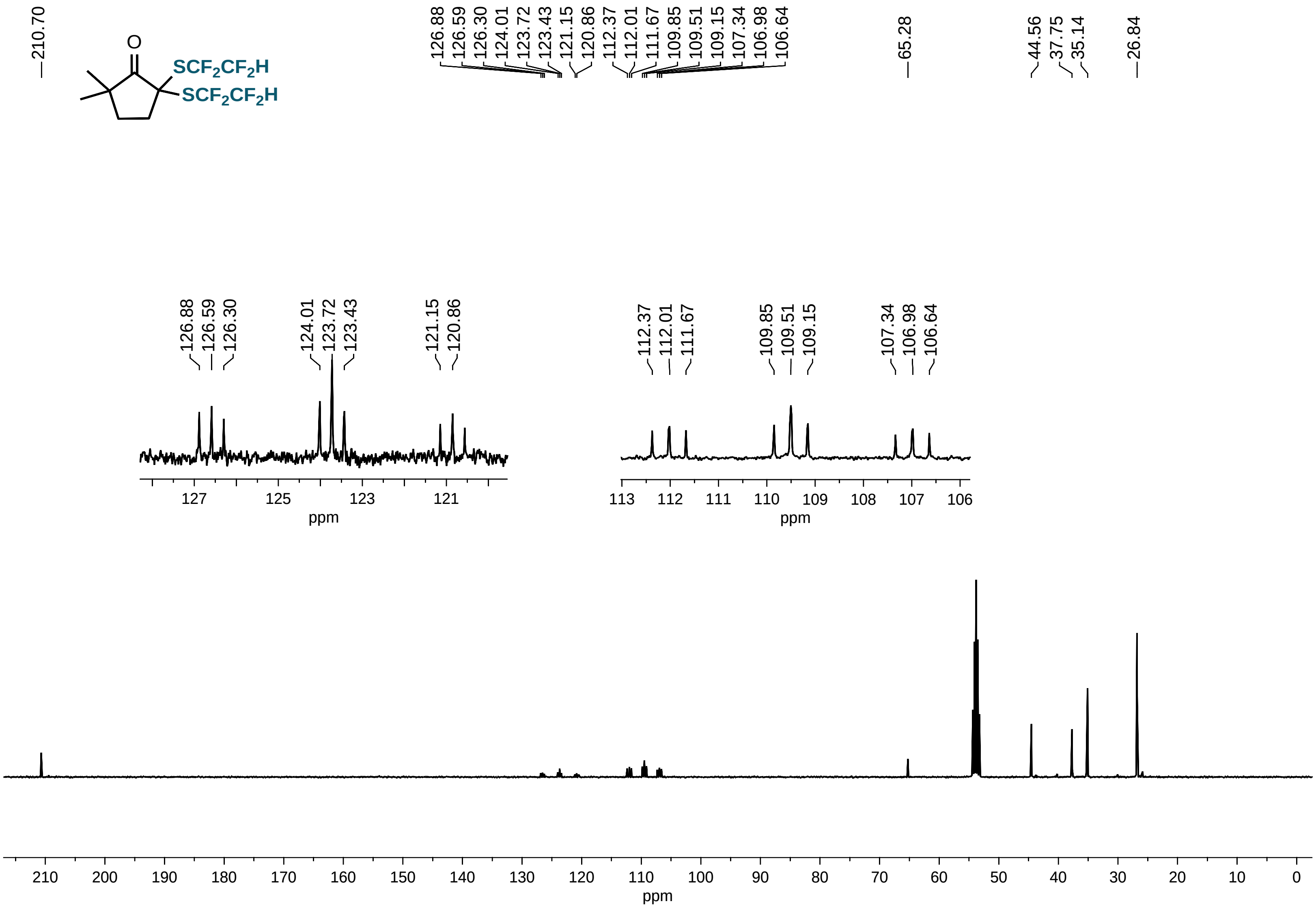

Figure S71. $^{13}C\{^{1}H\}$ NMR (CD_2Cl_2, 100.6 MHz) of **14a**

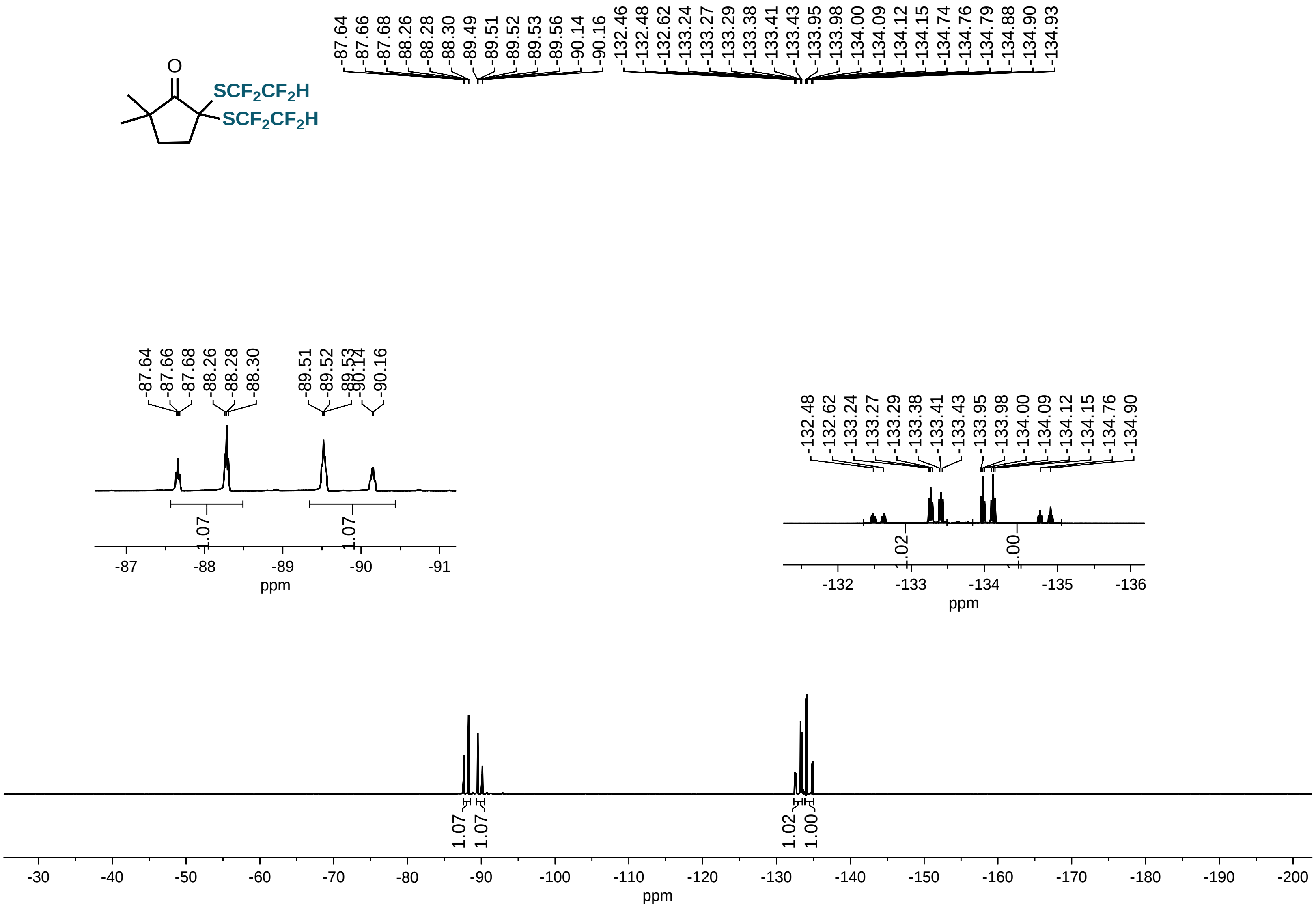

Figure S72. ^{19}F NMR (CD_2Cl_2, 376.5 MHz) of **14a**

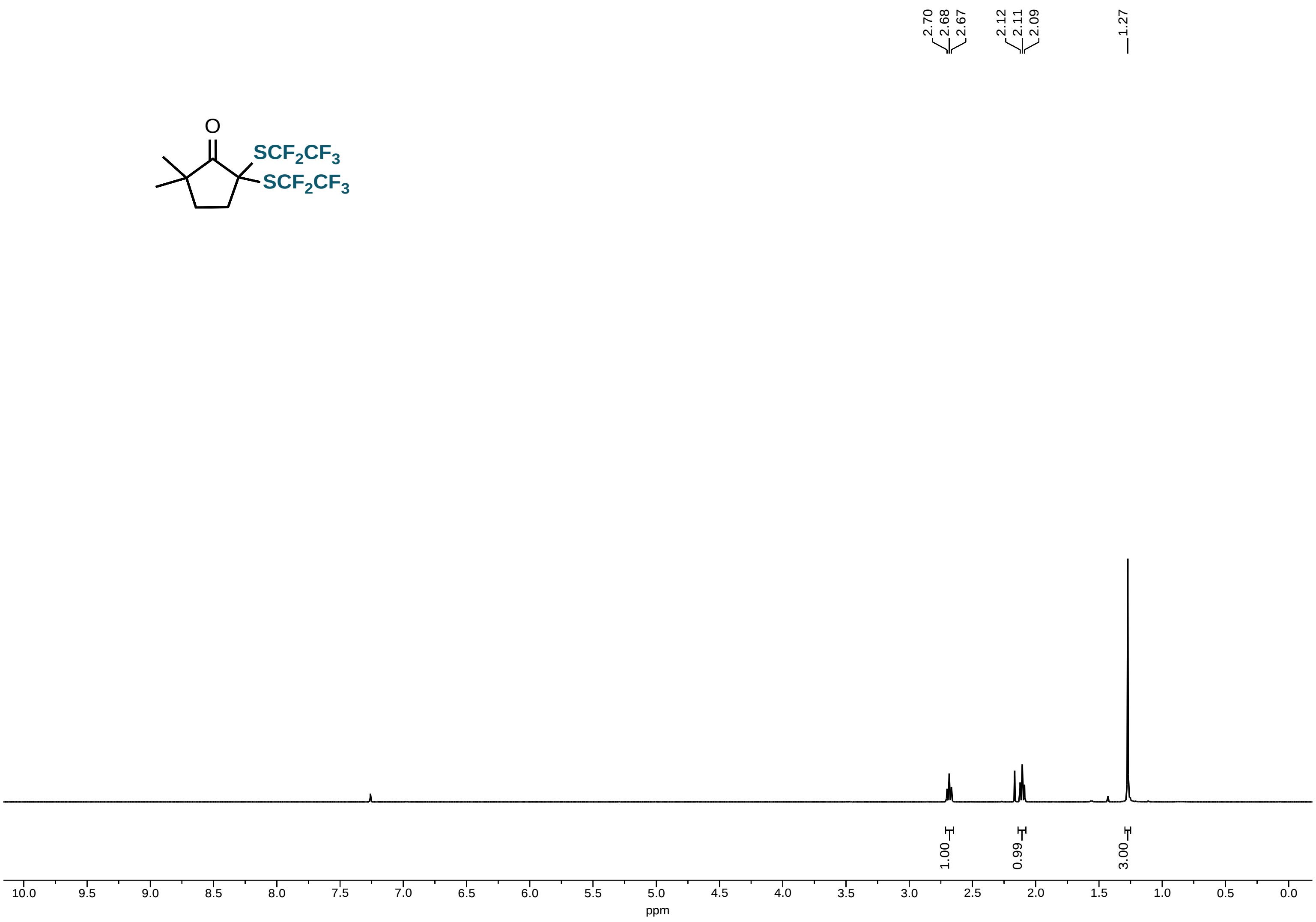

Figure S73. ^{1}H NMR ($CDCl_3$, 400 MHz) of **14b**

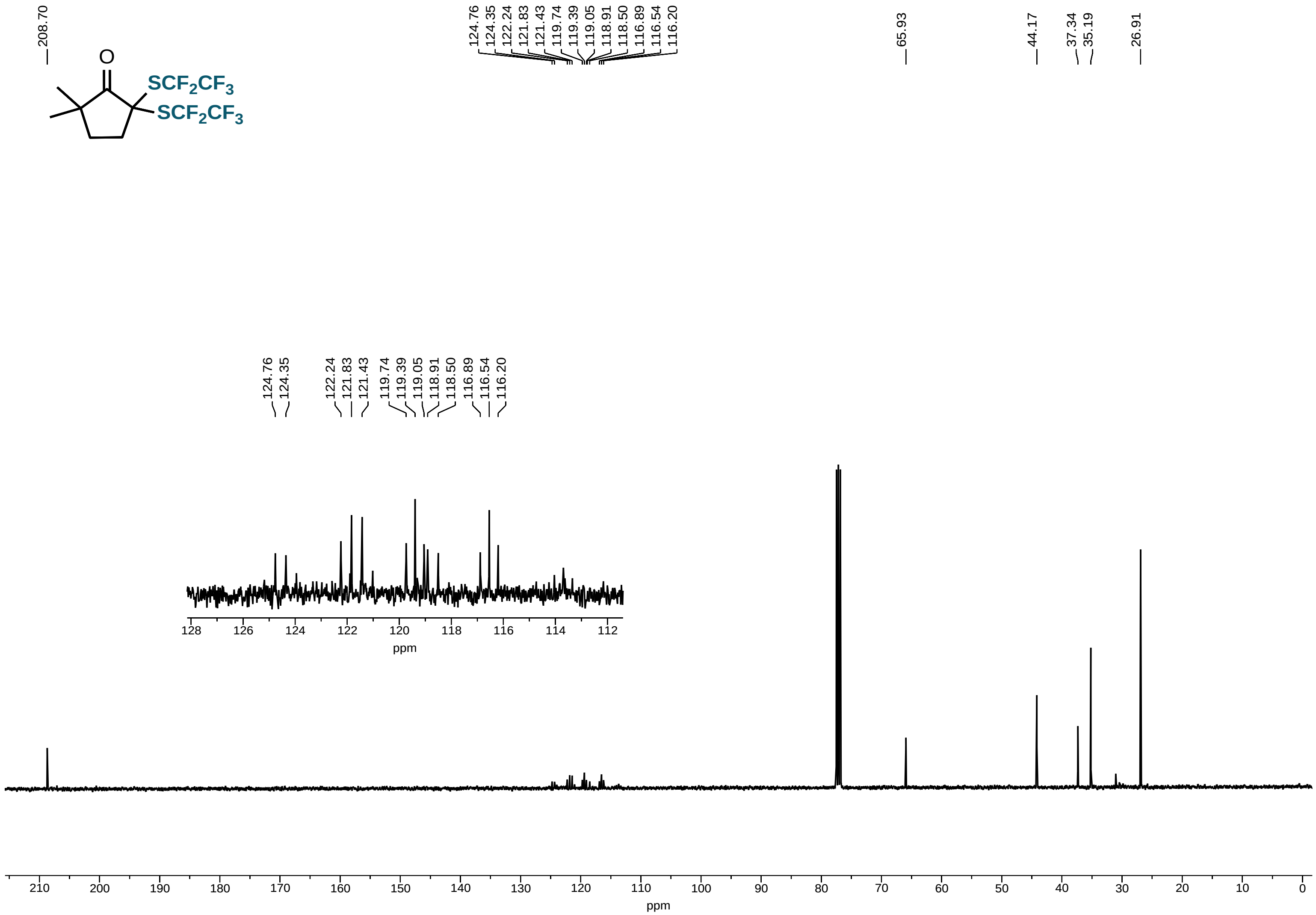

Figure S74. $^{13}C\{^{1}H\}$ NMR ($CDCl_3$, 100.6 MHz) of **14b**

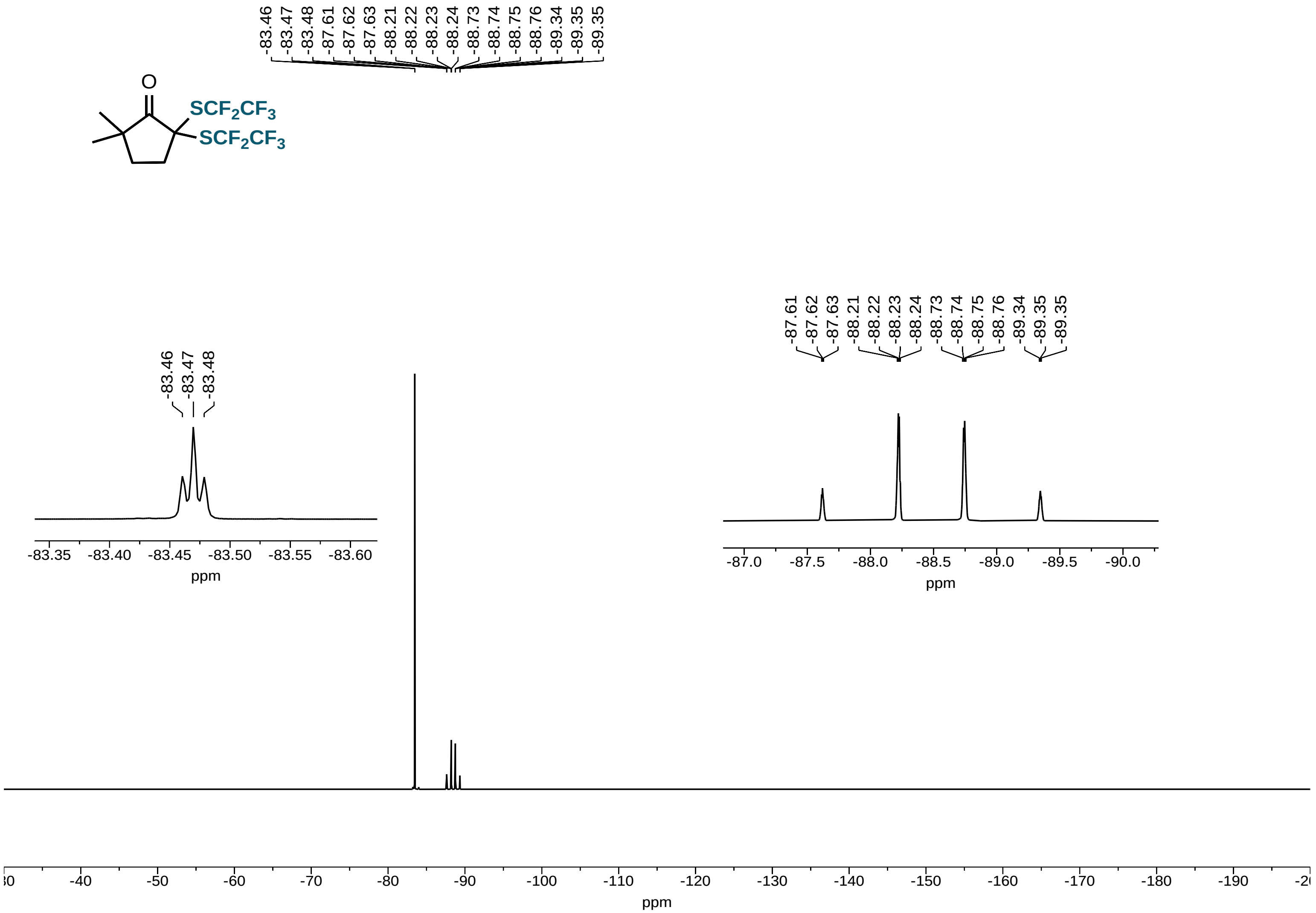

Figure S75. ^{19}F NMR ($CDCl_3$, 376.5 MHz) of **14b**

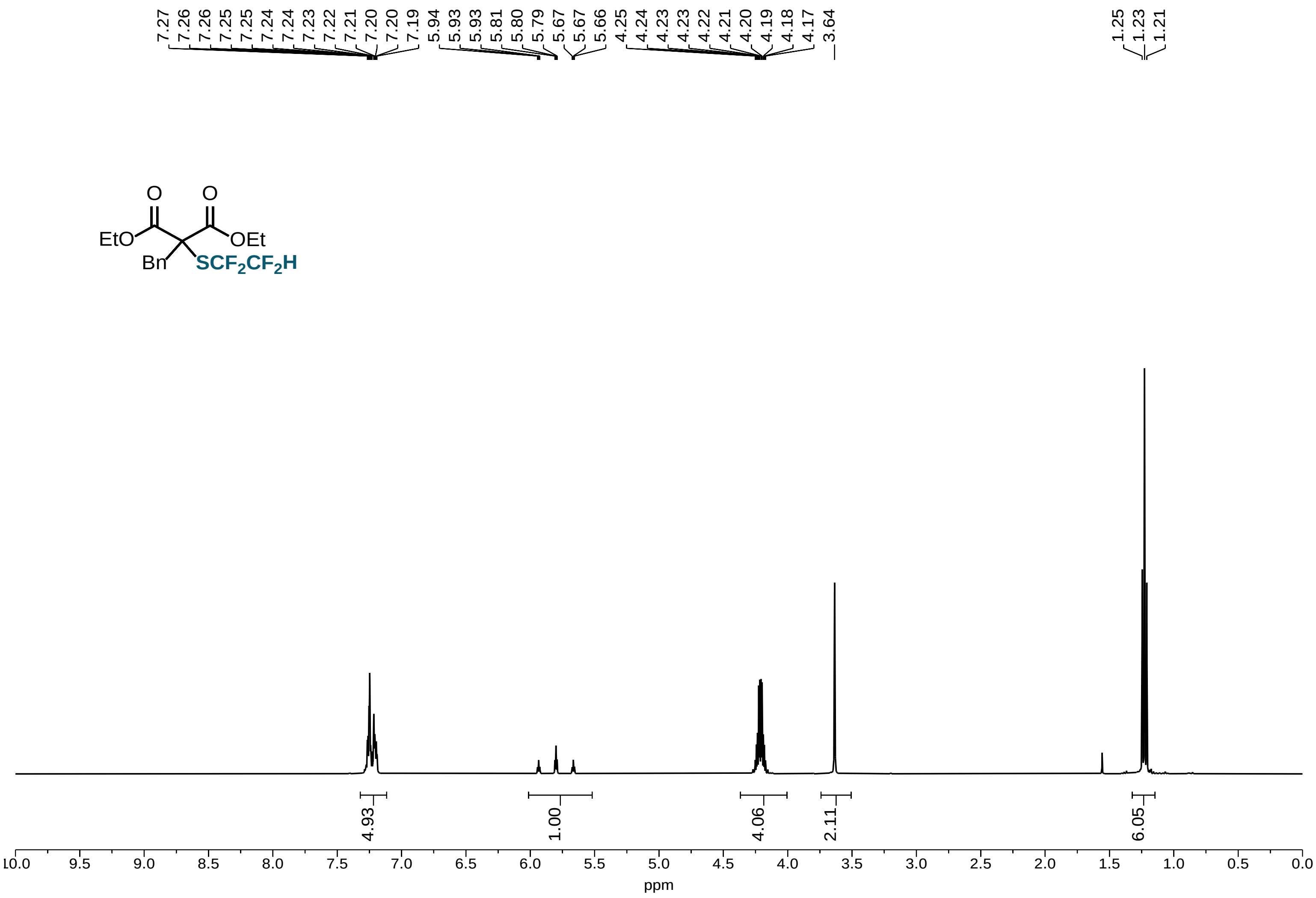

Figure S76. ^{1}H NMR ($CDCl_3$, 400 MHz) of **15a**

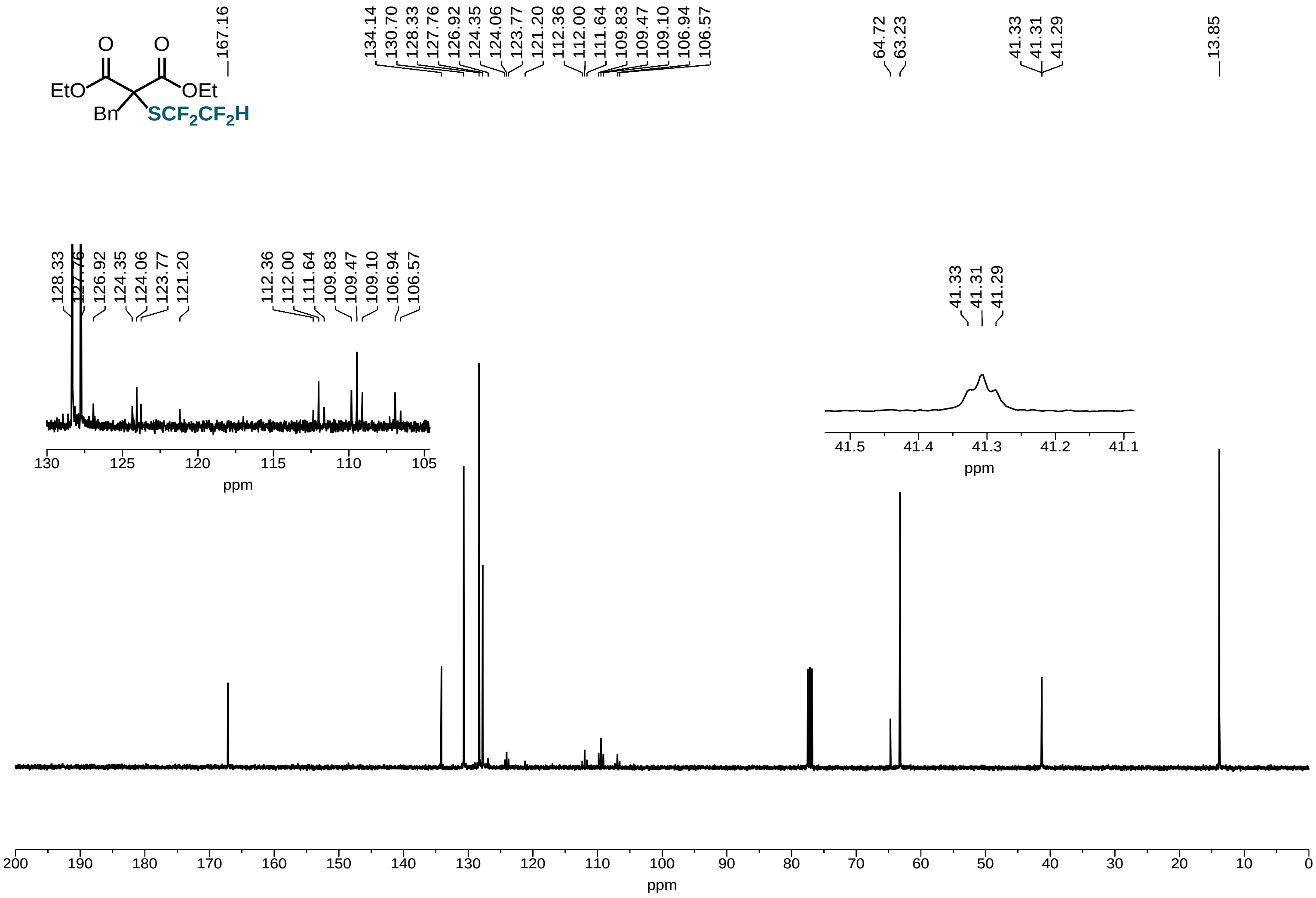

Figure S77. $^{13}C\{^1H\}$ NMR ($CDCl_3$, 100.6 MHz) of **15a**

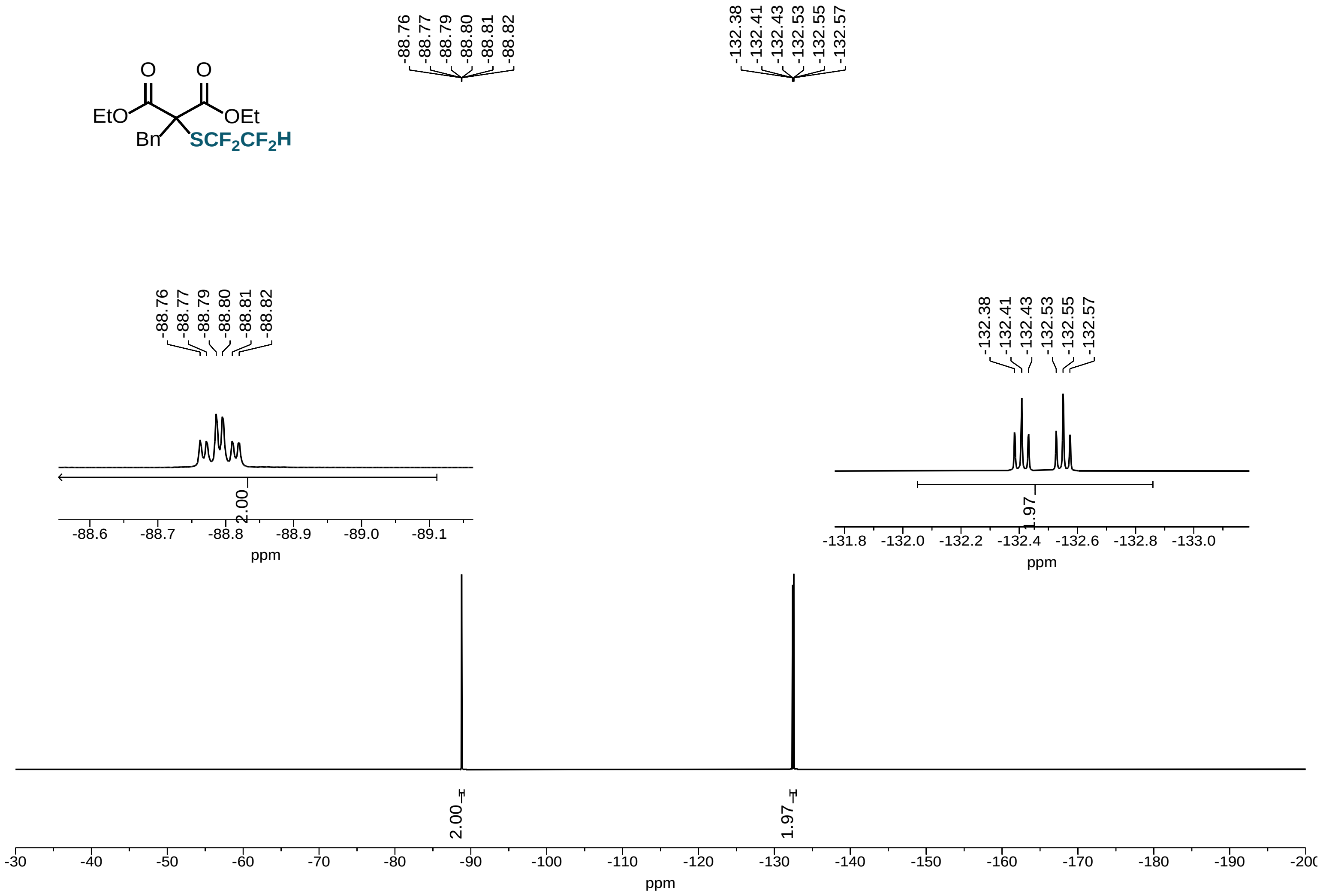

Figure S78. ^{19}F NMR ($CDCl_3$, 376.5 MHz) of **15a**

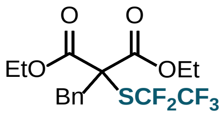

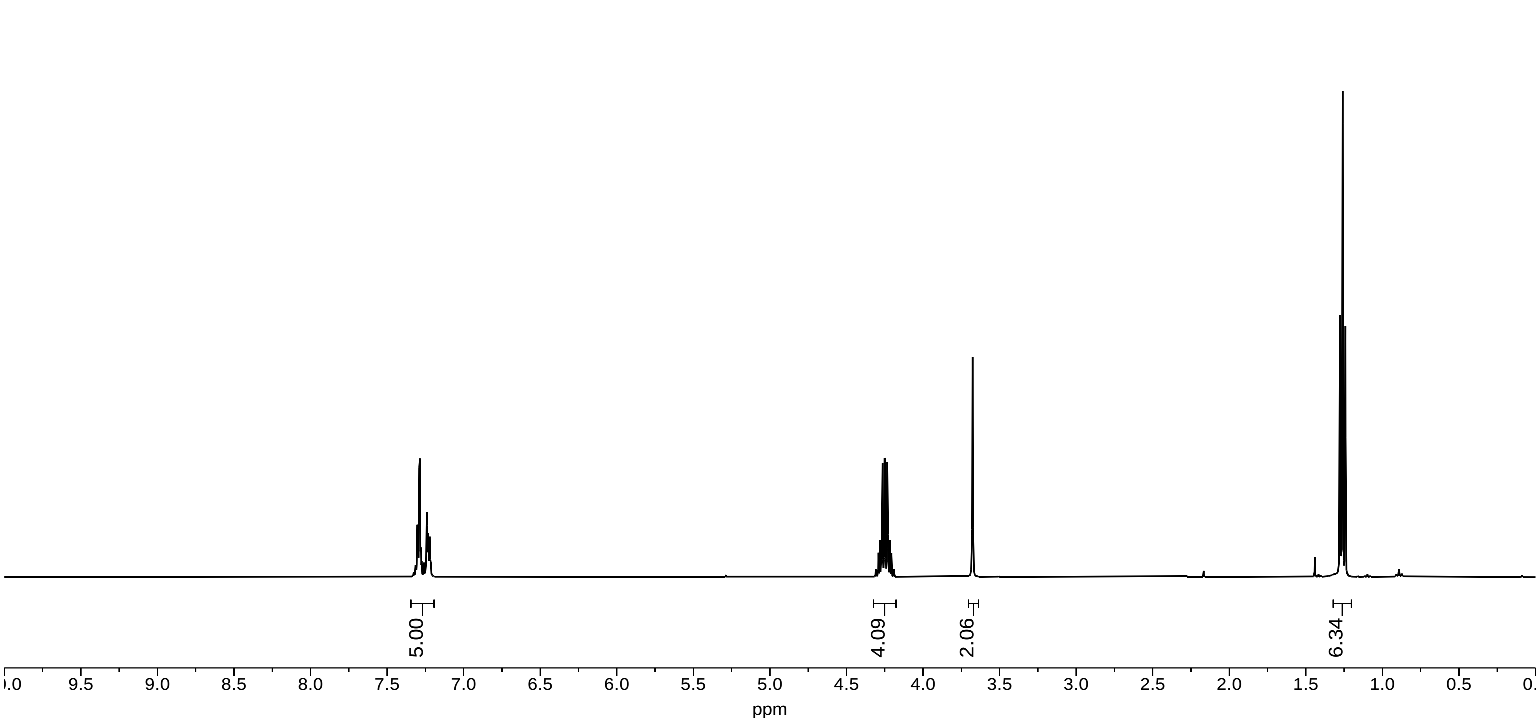

Figure S79. ^{1}H NMR ($CDCl_3$, 400 MHz) of **15b**

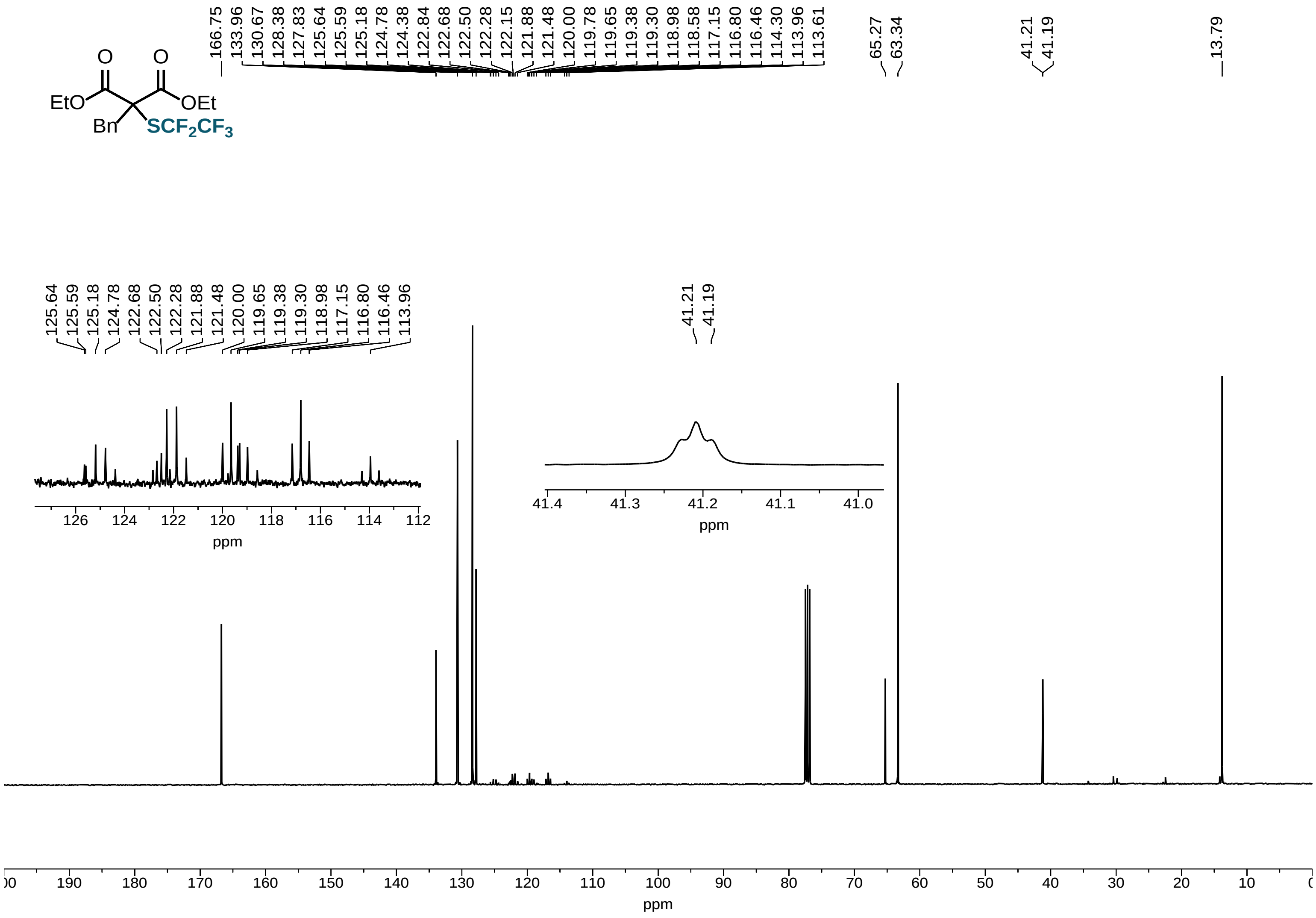

Figure S80. $^{13}C\{^1H\}$ NMR ($CDCl_3$, 100.6 MHz) of **15b**

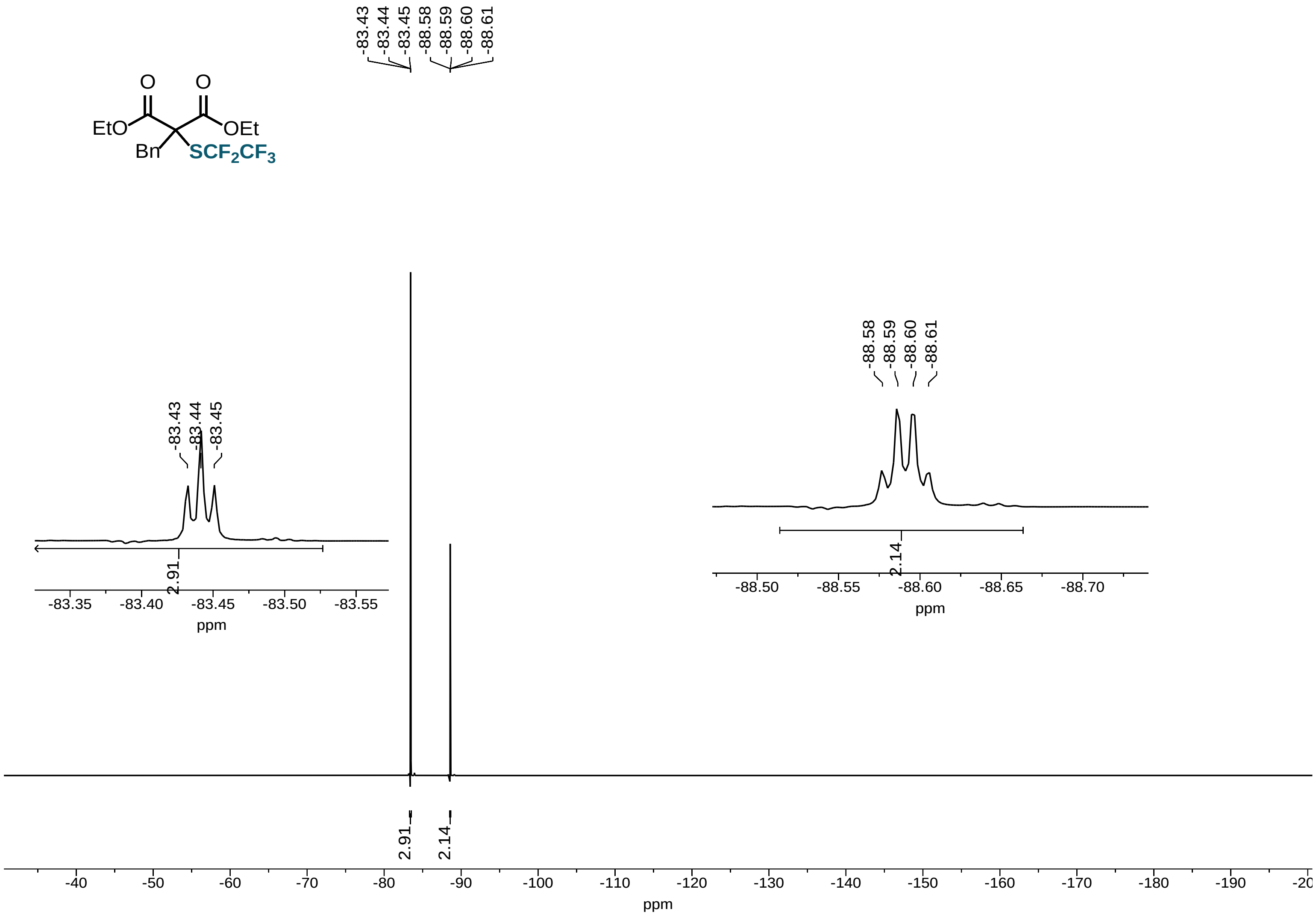

Figure S81. ^{19}F NMR ($CDCl_3$, 376.5 MHz) of **15b**

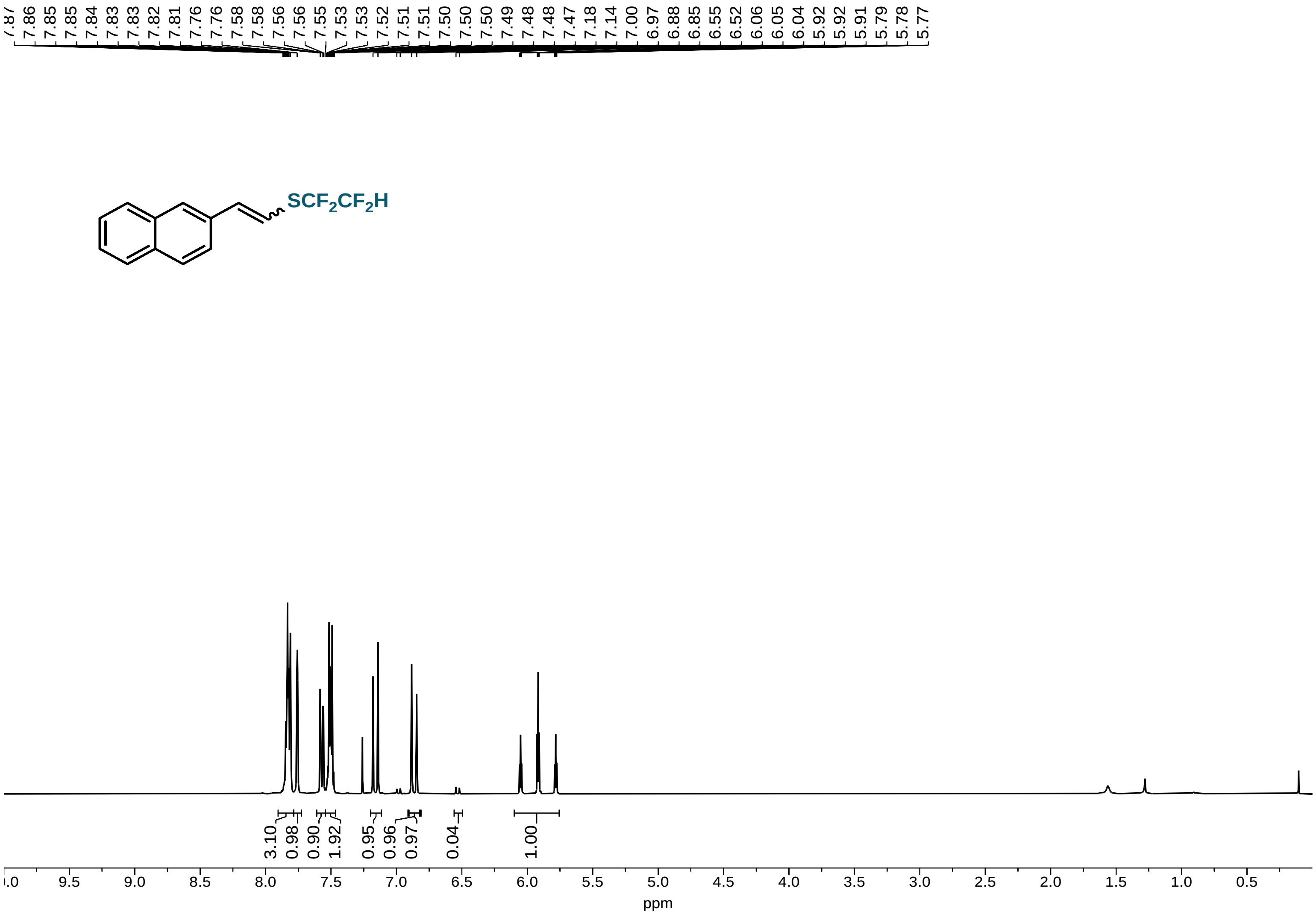

Figure S82. ^{1}H NMR ($CDCl_3$, 400 MHz) of **16a**

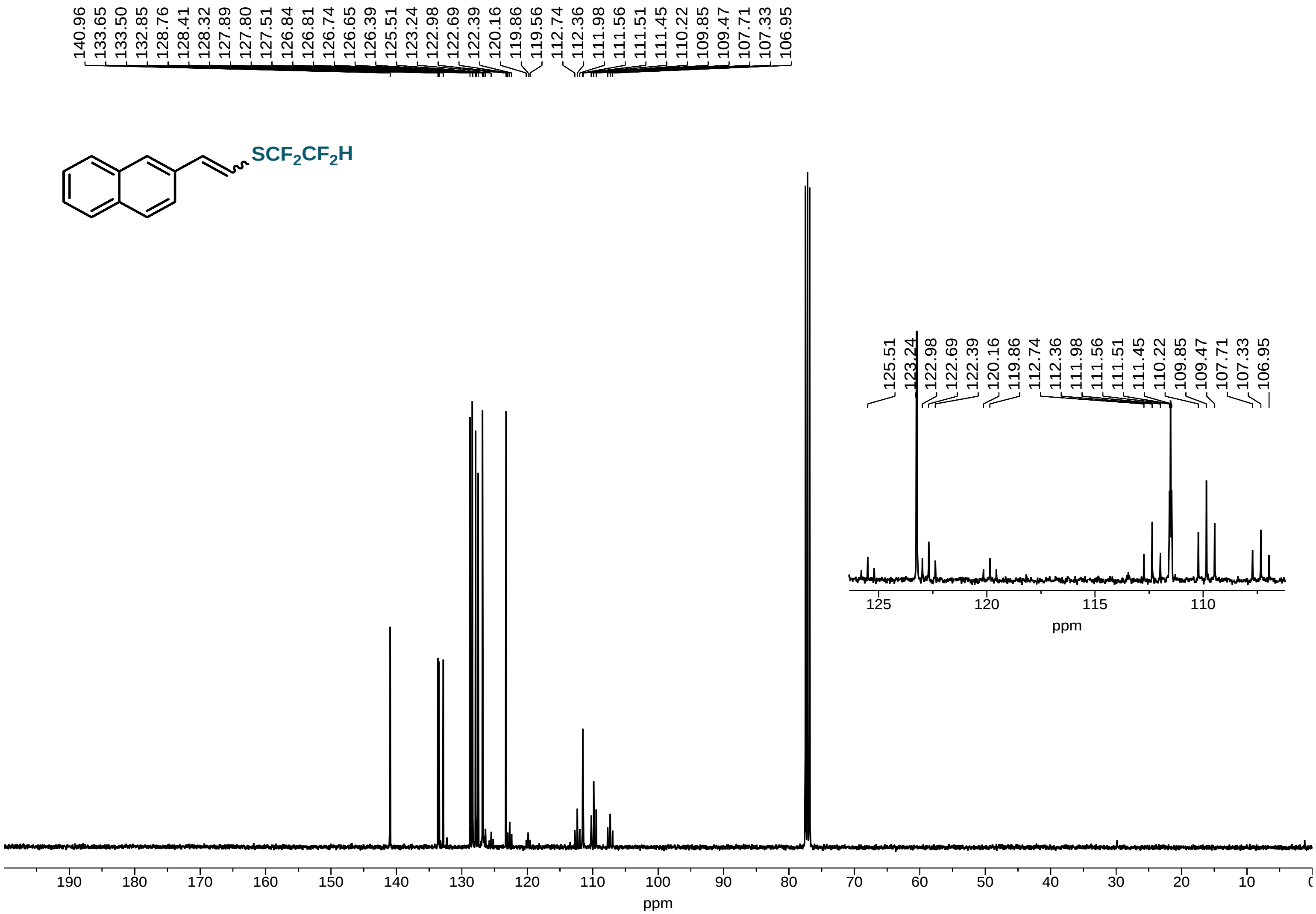

Figure S83. $^{13}C\{^1H\}$ NMR ($CDCl_3$, 100.6 MHz) of **16a**

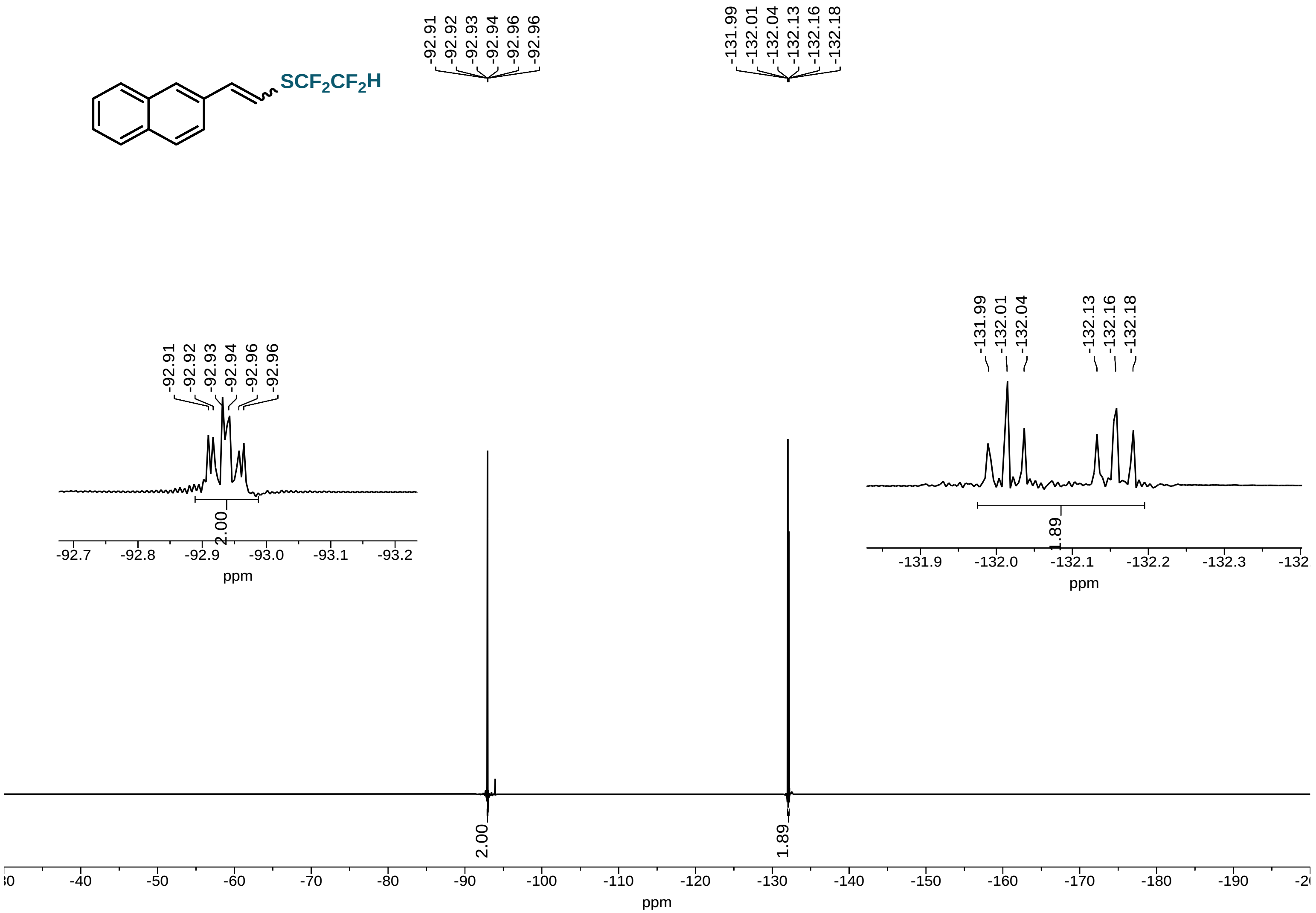

Figure S84. ^{19}F NMR ($CDCl_3$, 376.5 MHz) of **16a**

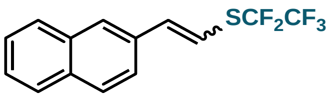

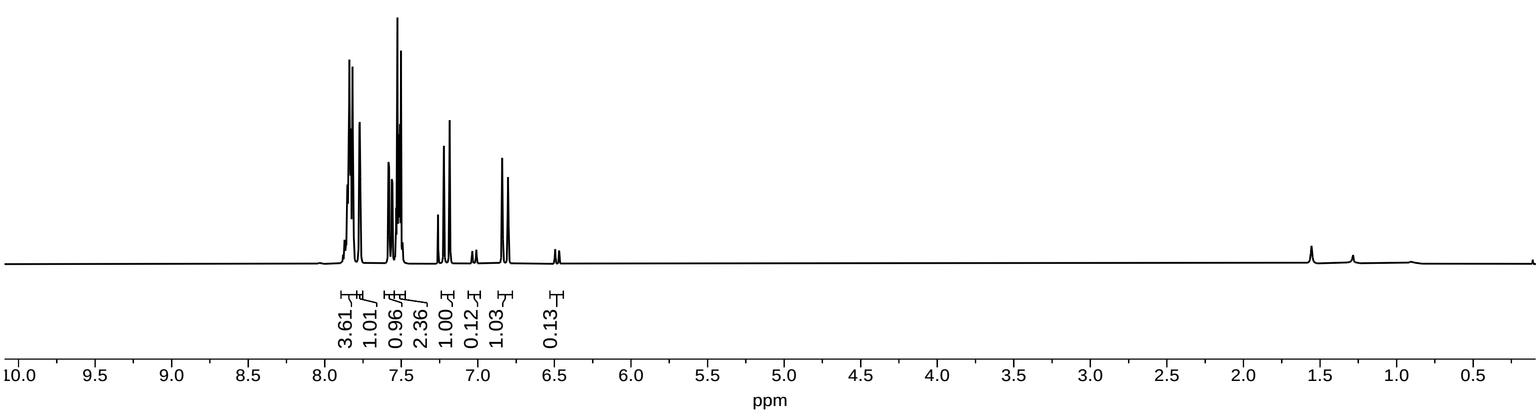

Figure S85. ^{1}H NMR ($CDCl_3$, 400 MHz) of **16b**

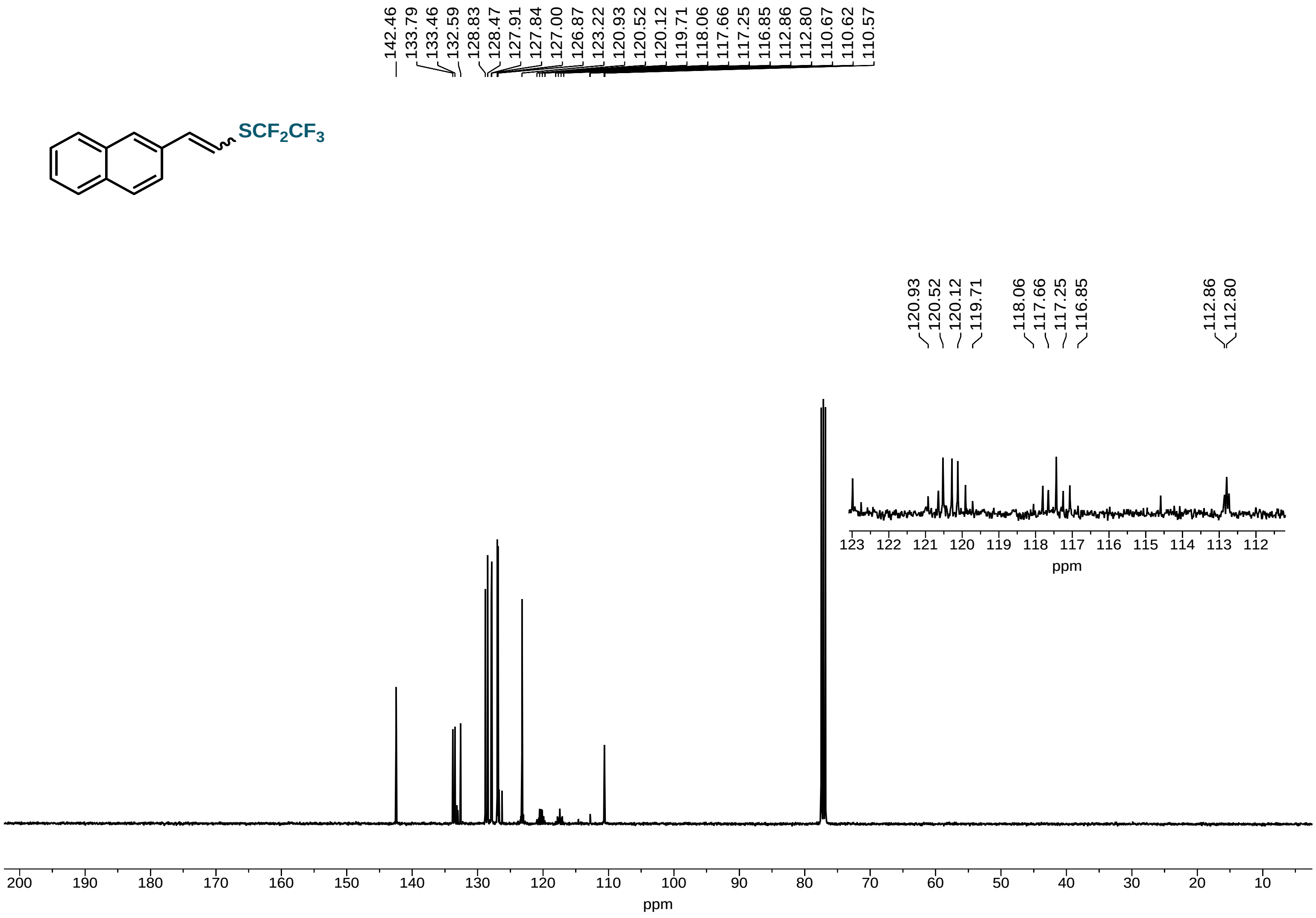

Figure S86. $^{13}C\{^{1}H\}$ NMR ($CDCl_3$, 100.6 MHz) of **16b**

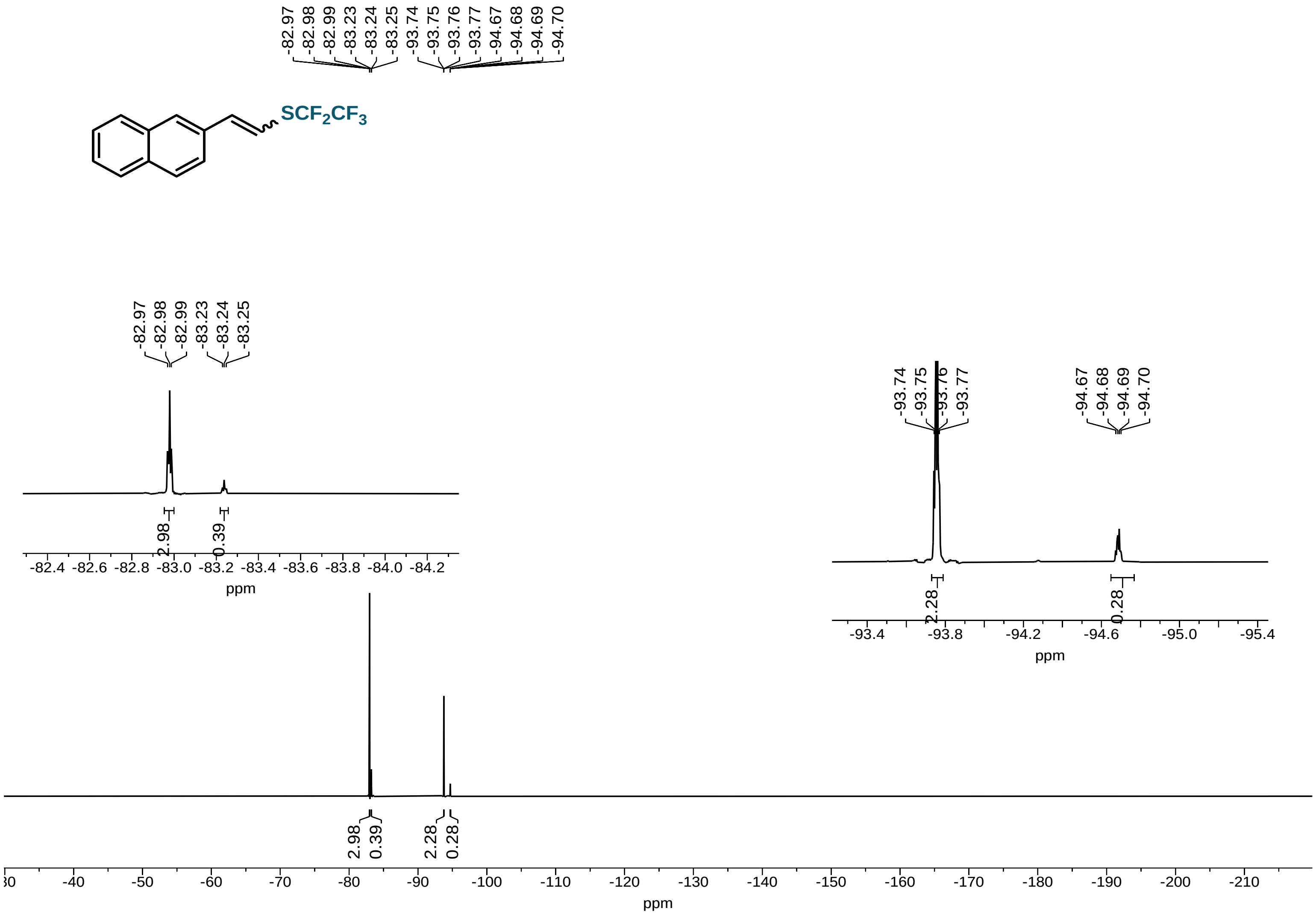

Figure S87. ^{19}F NMR ($CDCl_3$, 376.5 MHz) of **16b**

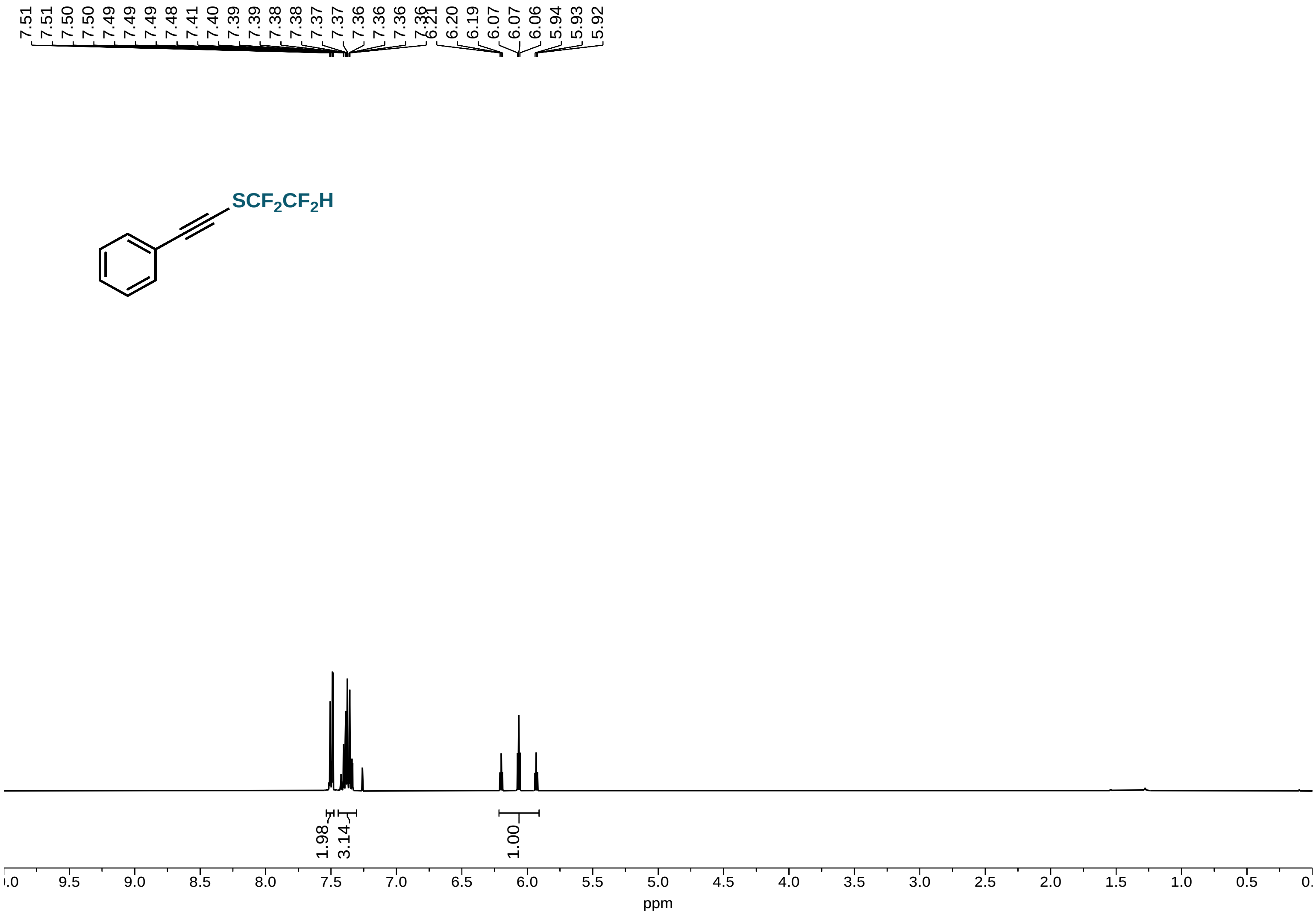

Figure S88. ^{1}H NMR ($CDCl_3$, 400 MHz) of **17a**

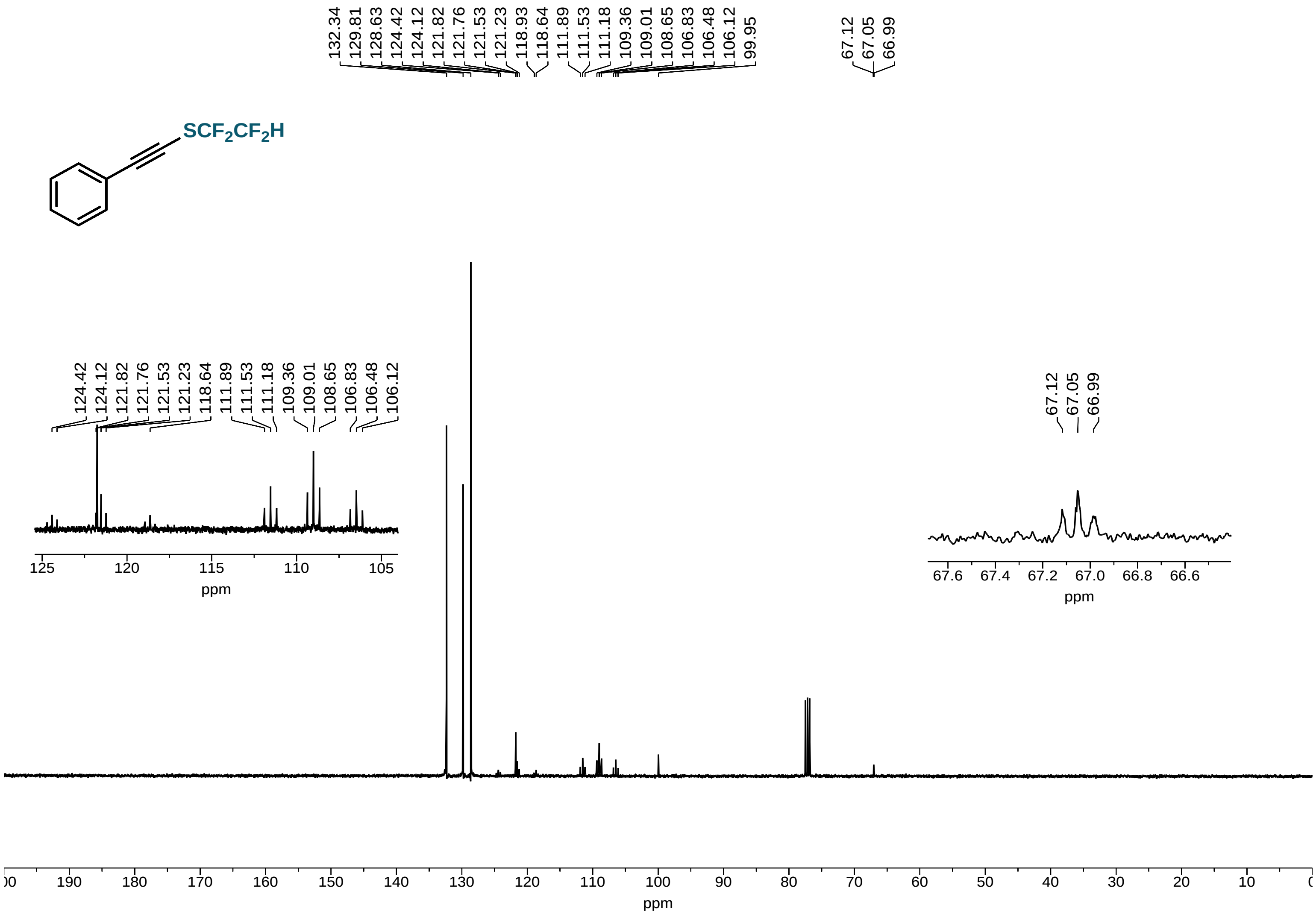

Figure S89. $^{13}C\{^1H\}$ NMR ($CDCl_3$, 100.6 MHz) of **17a**

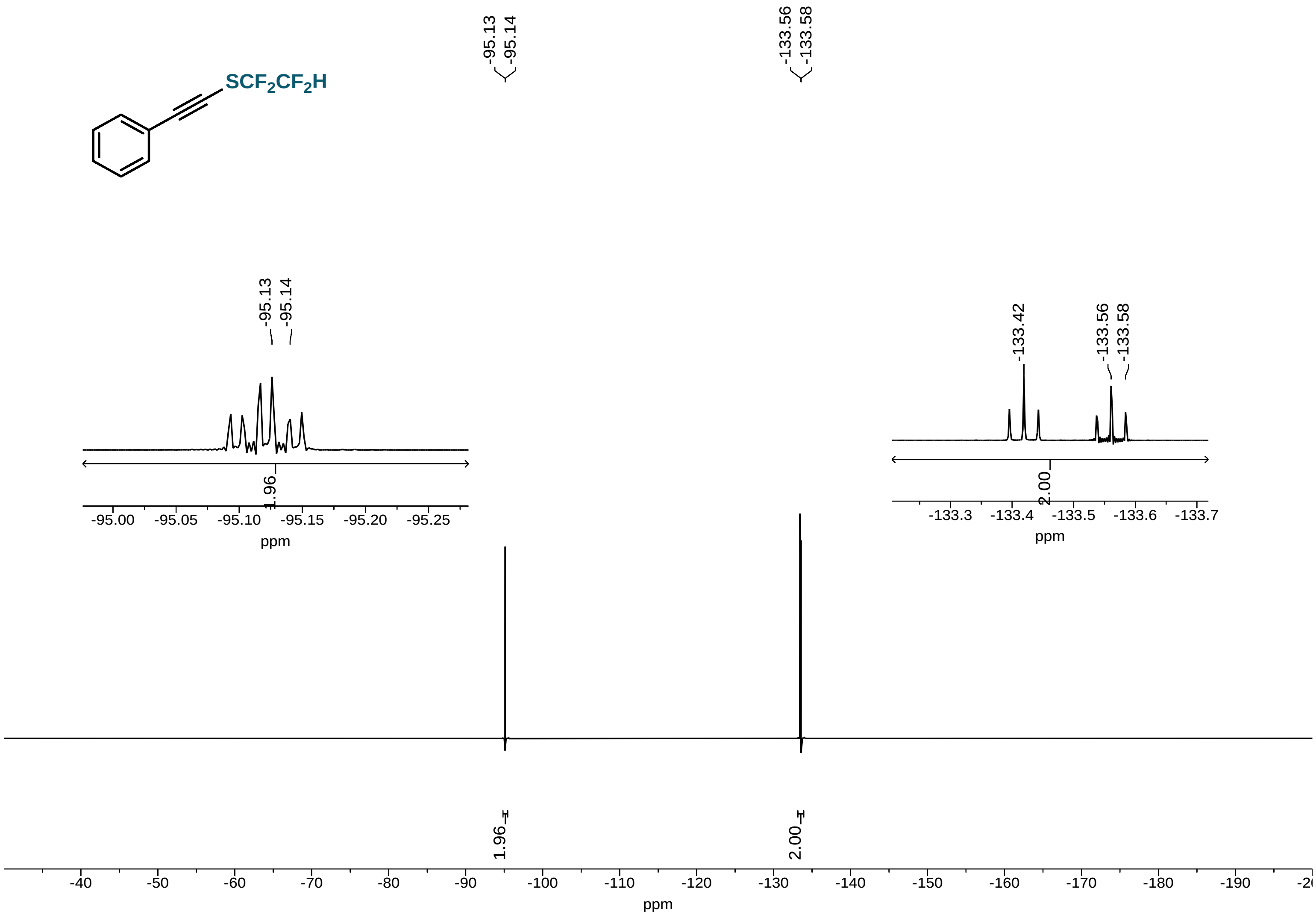

Figure 90. ^{19}F NMR ($CDCl_3$, 376.5 MHz) of **17a**

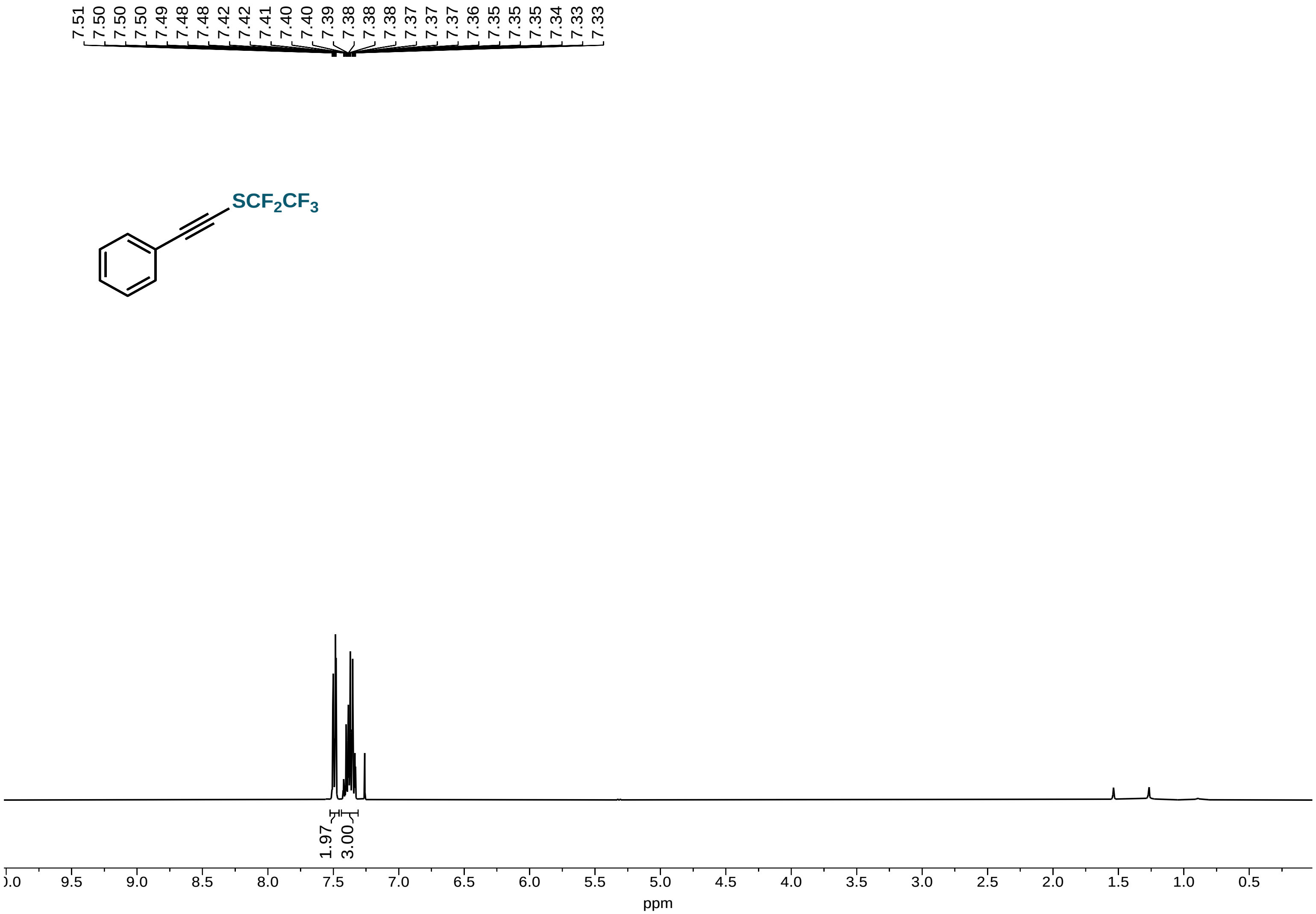

Figure S91. ^{1}H NMR ($CDCl_3$, 400 MHz) of **17b**

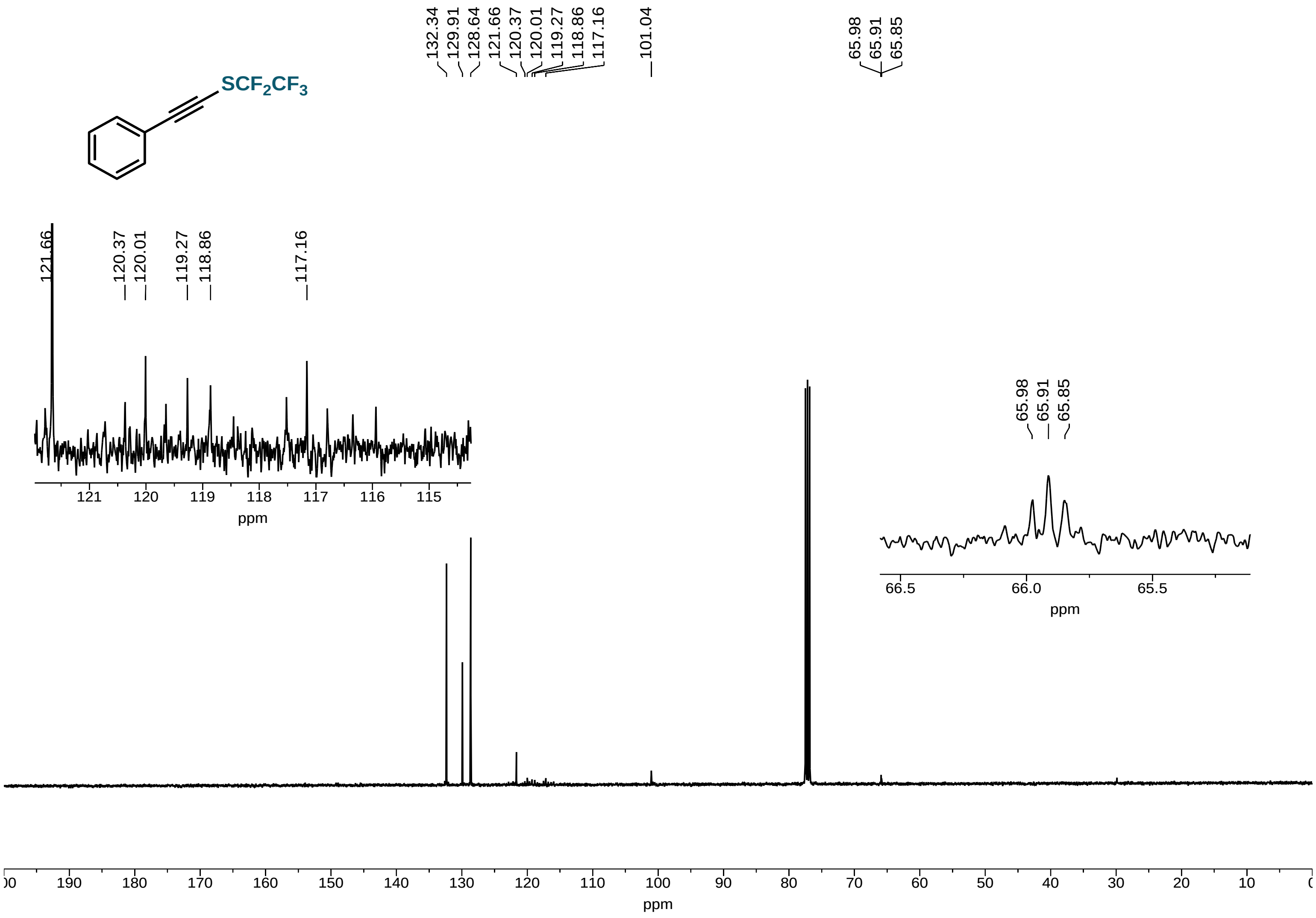

Figure S92. $^{13}C\{^{1}H\}$ NMR ($CDCl_3$, 100.6 MHz) of **17b**

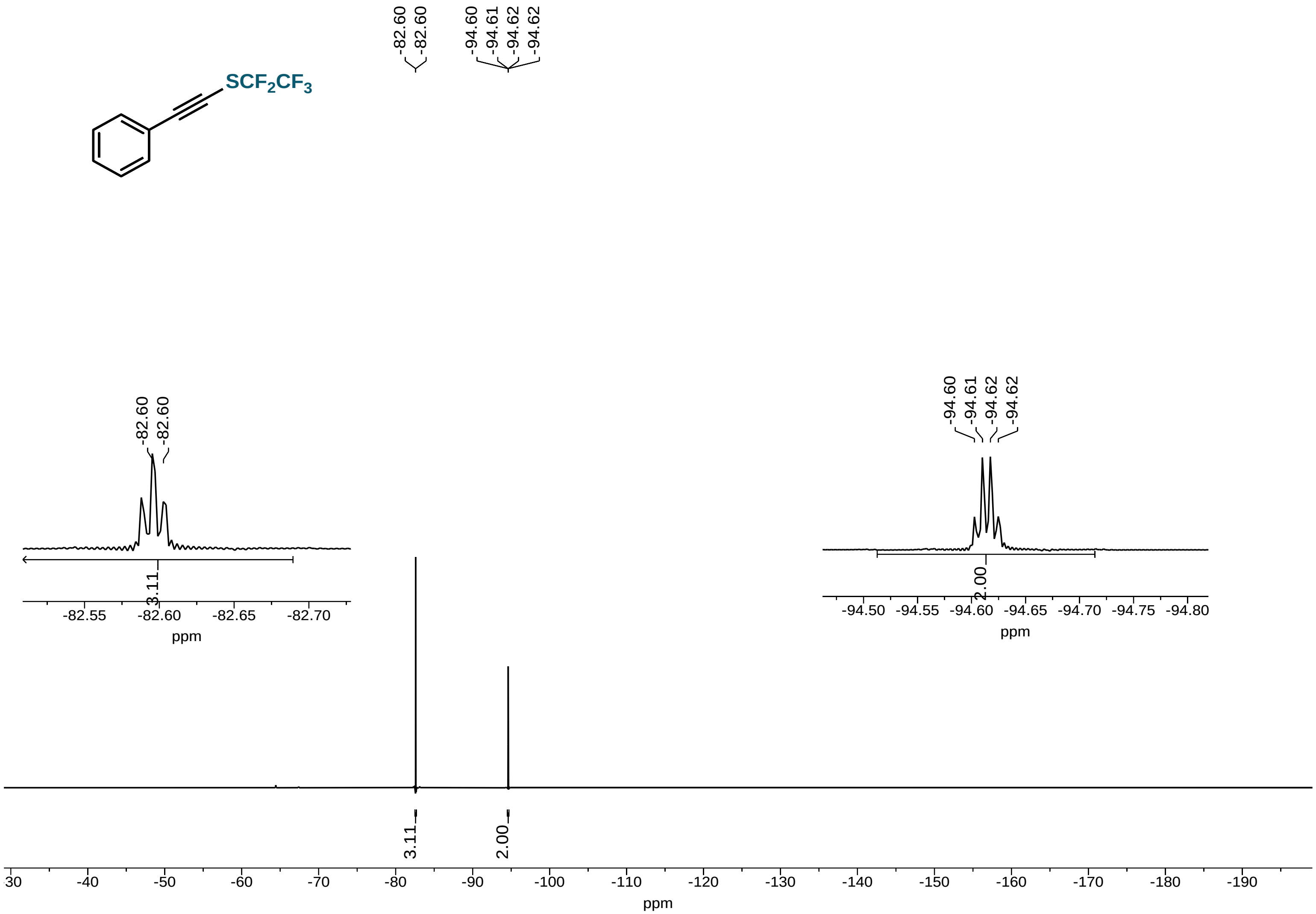

Figure S93 ^{19}F NMR ($CDCl_3$, 376.5 MHz) of **17b**

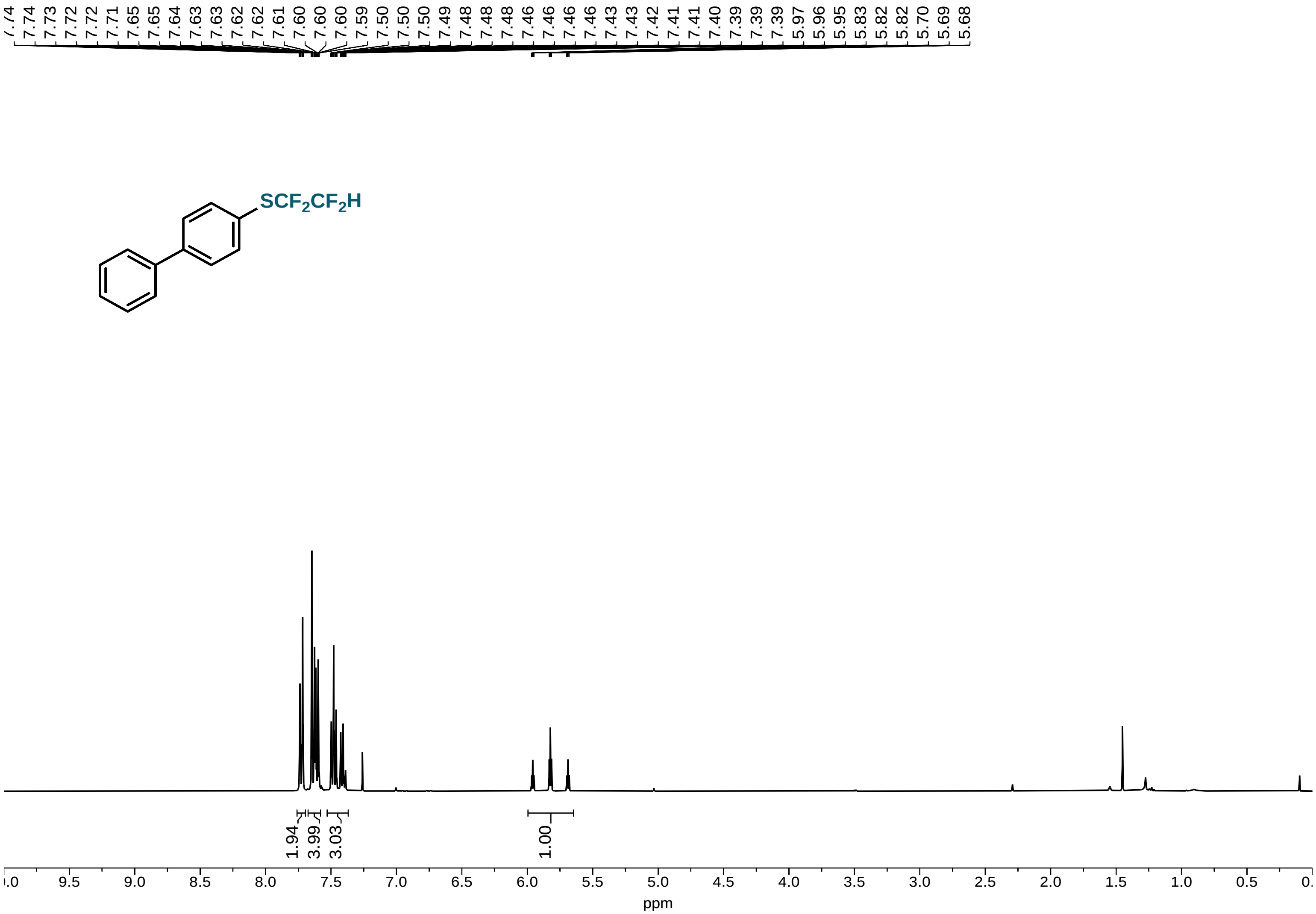

Figure S94. ^{1}H NMR ($CDCl_3$, 400 MHz) of **18a**

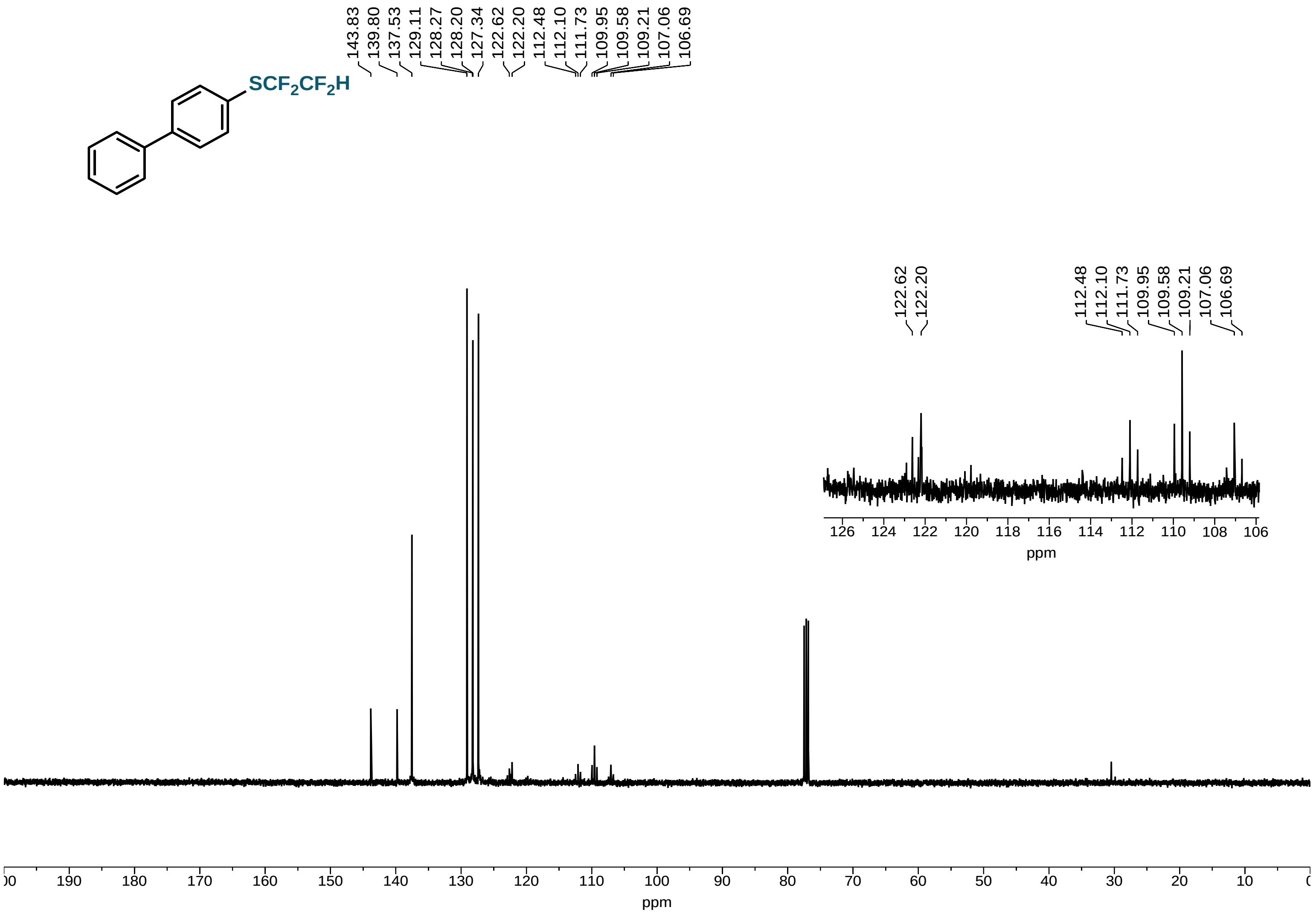

Figure S95. $^{13}C\{^{1}H\}$ NMR ($CDCl_3$, 100.6 MHz) of **18a**

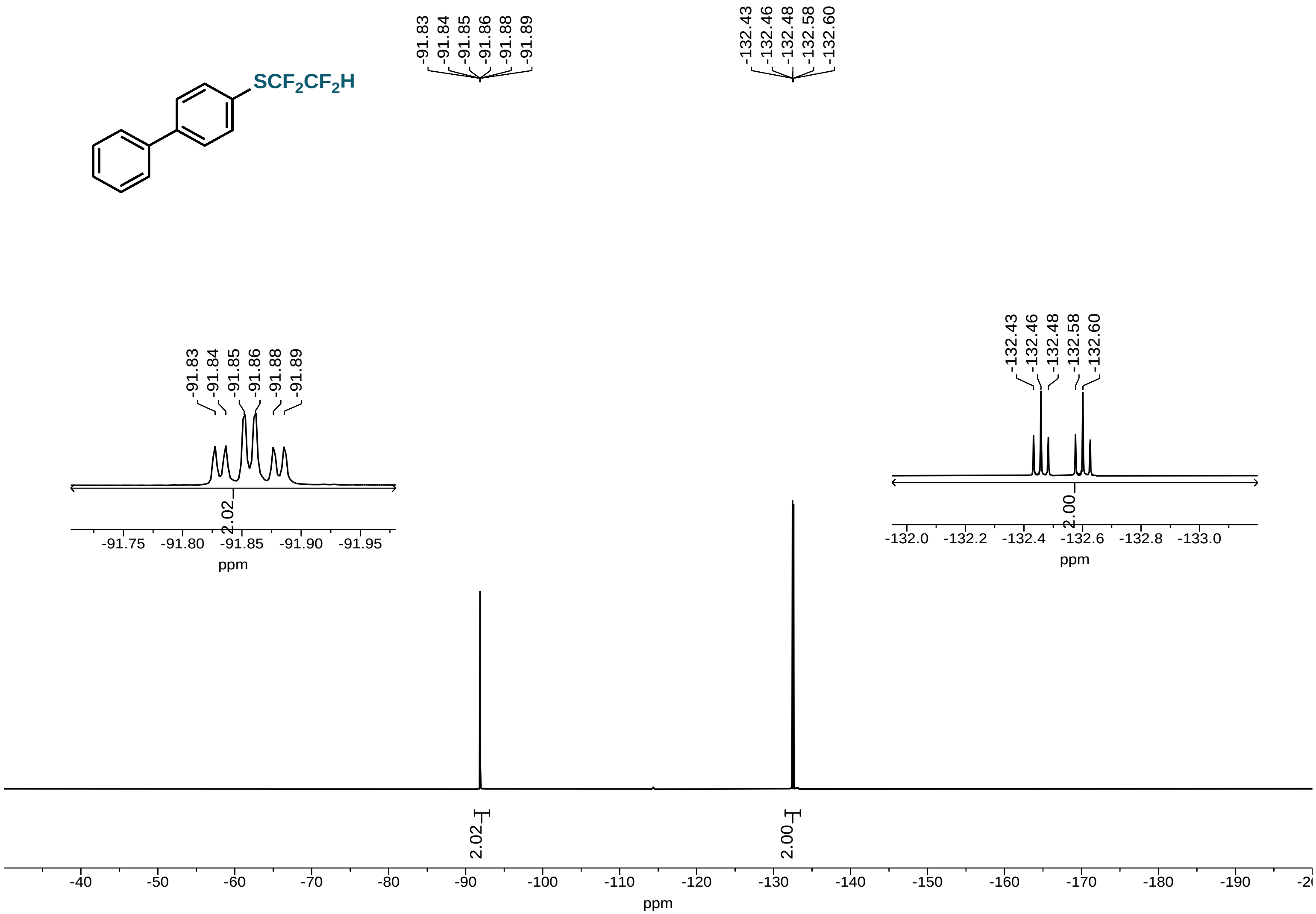

Figure S96. ^{19}F NMR ($CDCl_3$, 376.5 MHz) of **18a**

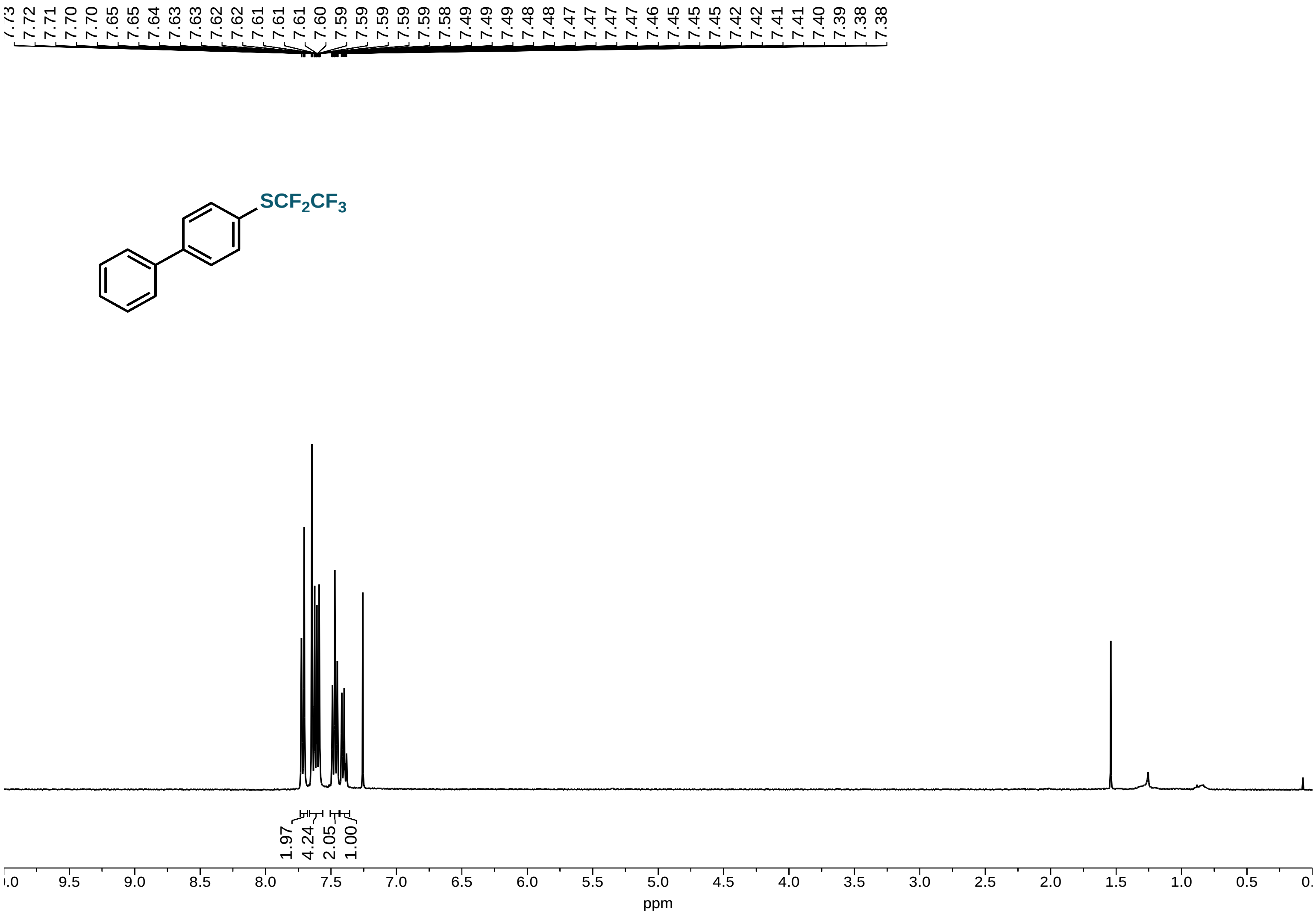

Figure S97. ^{1}H NMR ($CDCl_3$, 400 MHz) of **18b**

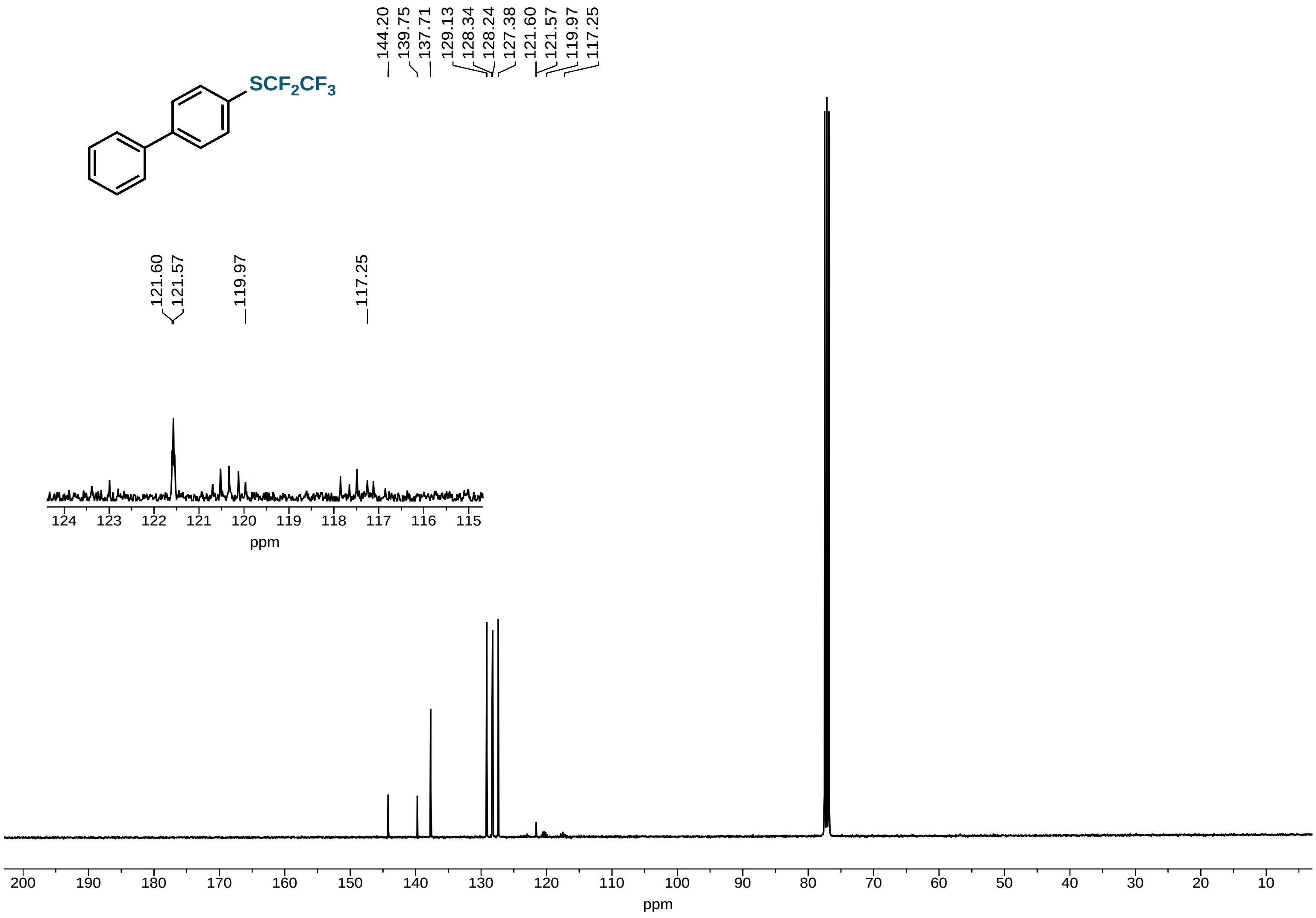

Figure S98. $^{13}C\{^1H\}$ NMR ($CDCl_3$, 100.6 MHz) of **18b**

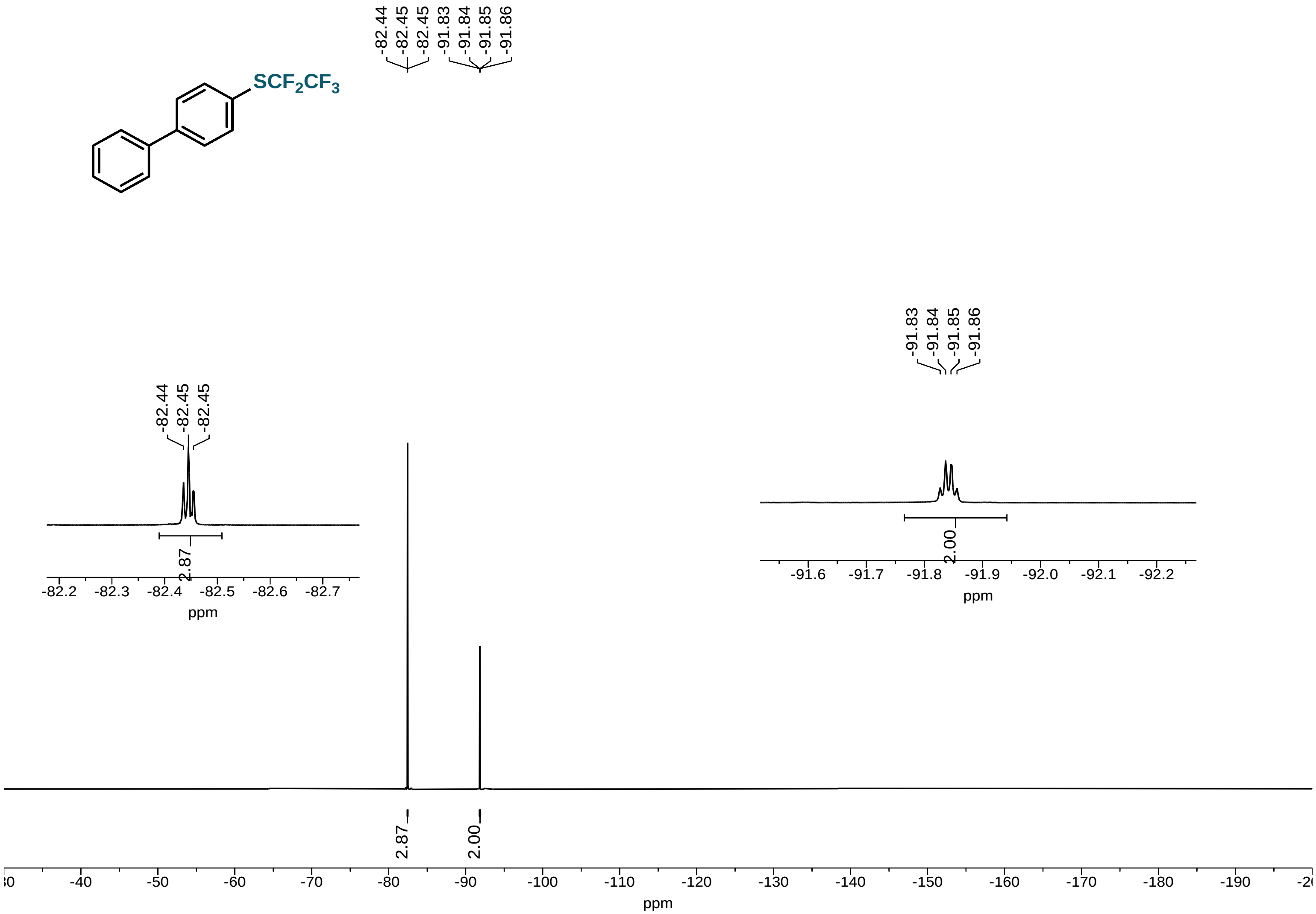

Figure S99. ^{19}F NMR ($CDCl_3$, 376.5 MHz) of **18b**

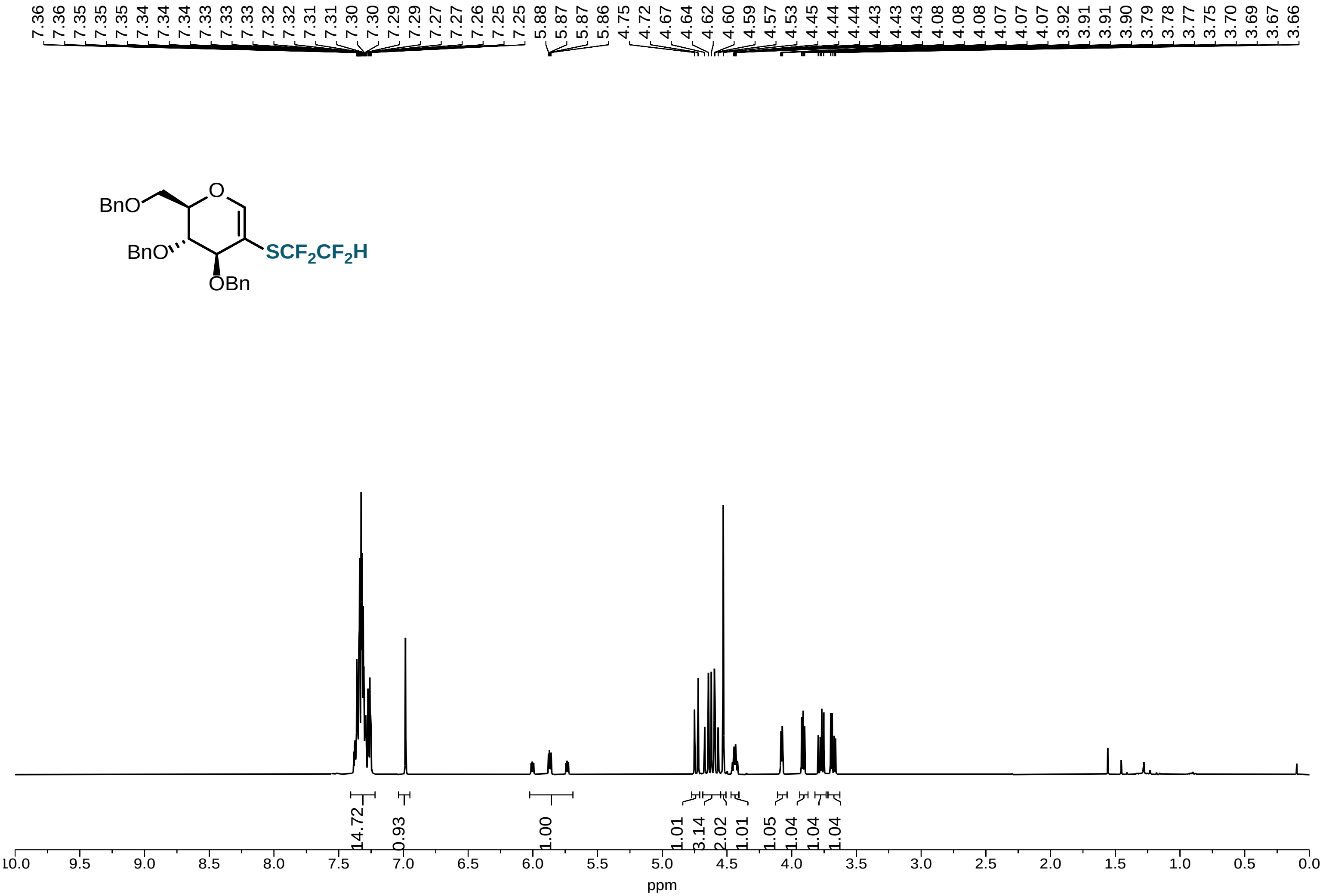

Figure 100. ^{1}H NMR ($CDCl_3$, 400 MHz) of **19a**

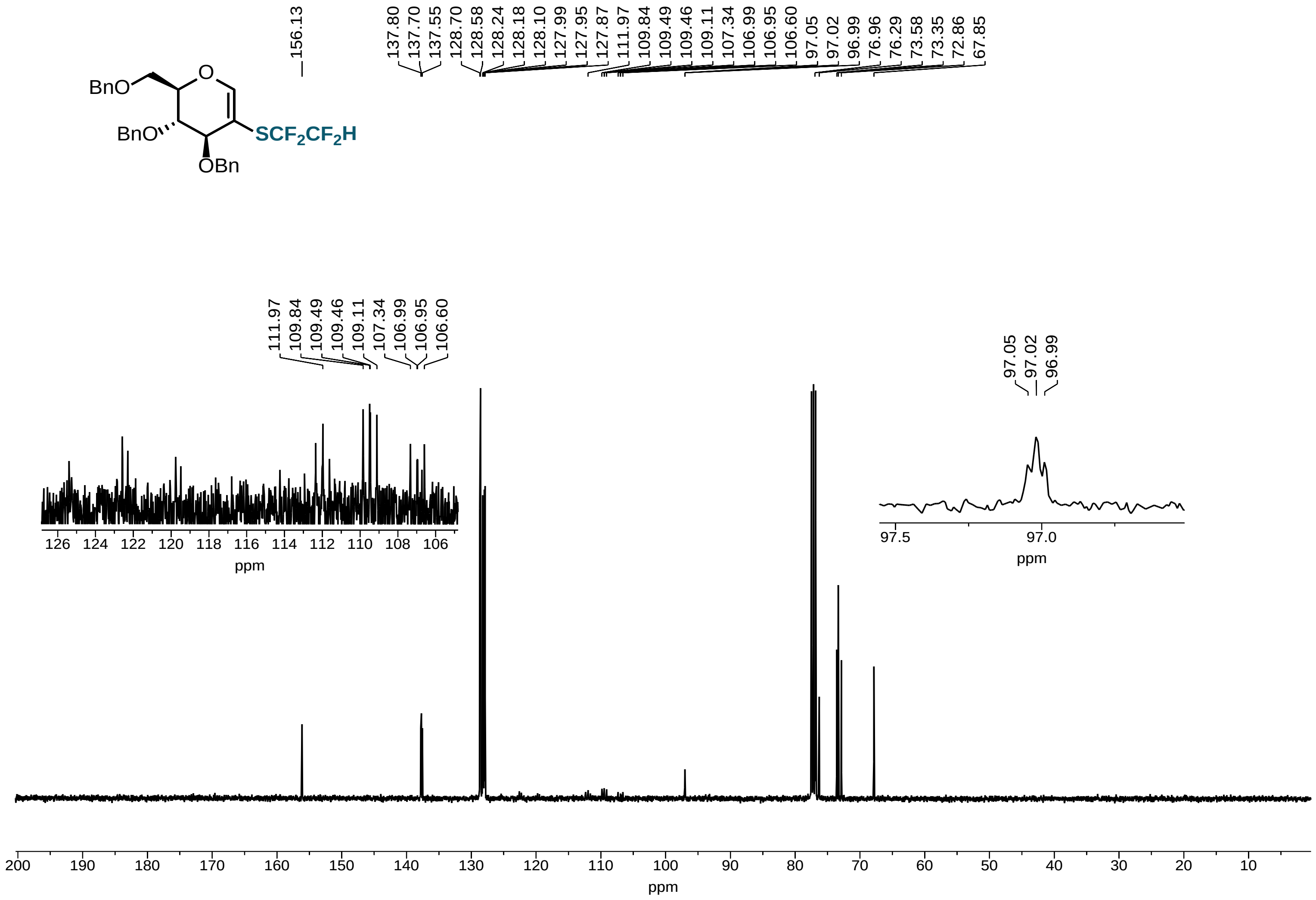

Figure S101. $^{13}C\{^{1}H\}$ NMR ($CDCl_3$, 100.6 MHz) of **19a**

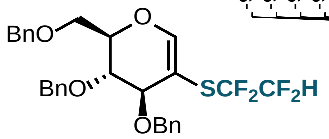

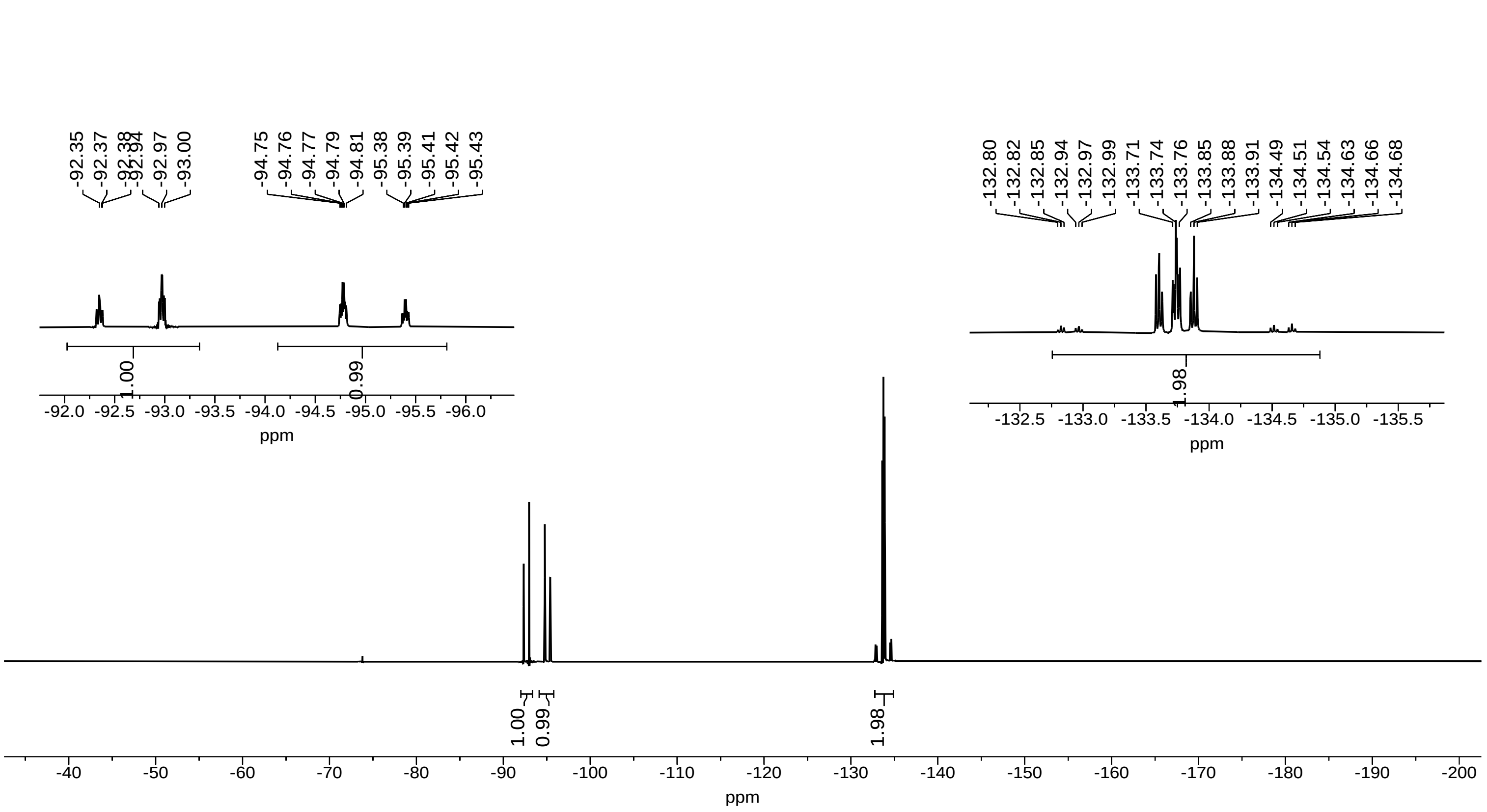

Figure S102. ^{19}F NMR ($CDCl_3$, 376.5 MHz) of **19a**

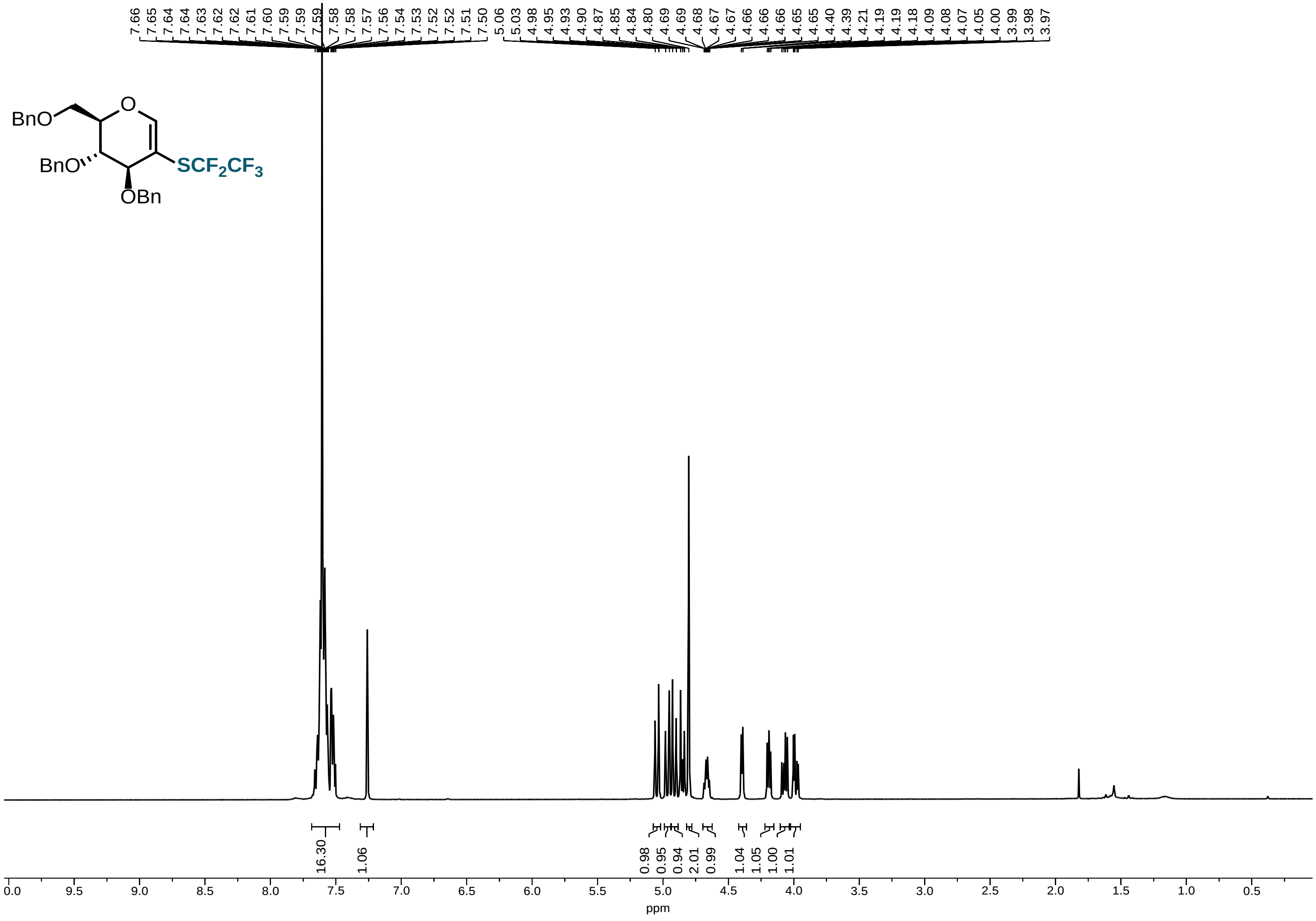

Figure S103. ^{1}H NMR ($CDCl_3$, 400 MHz) of **19b**

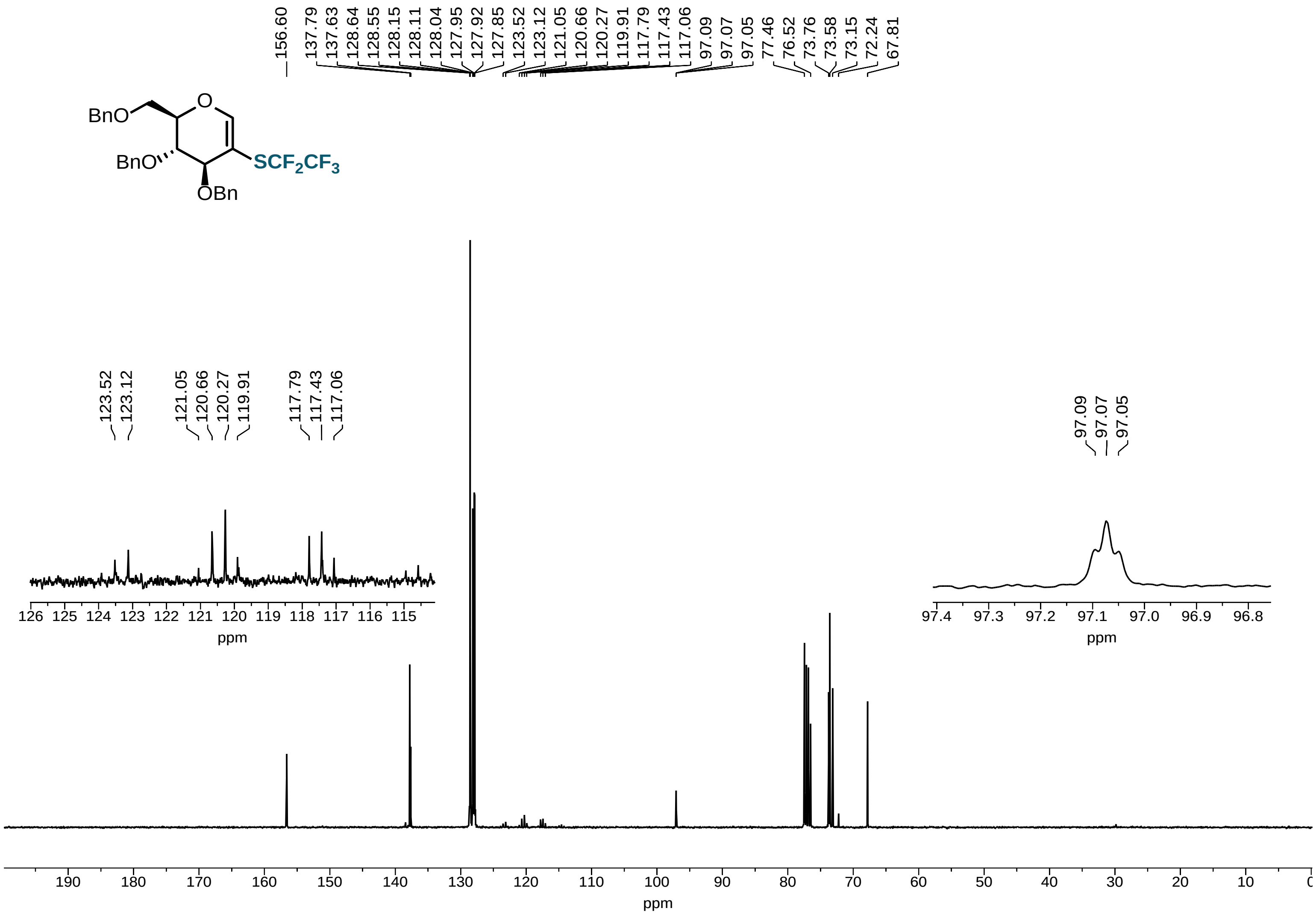

Figure S104. $^{13}C\{^{1}H\}$ NMR ($CDCl_3$, 100.6 MHz) of **19b**

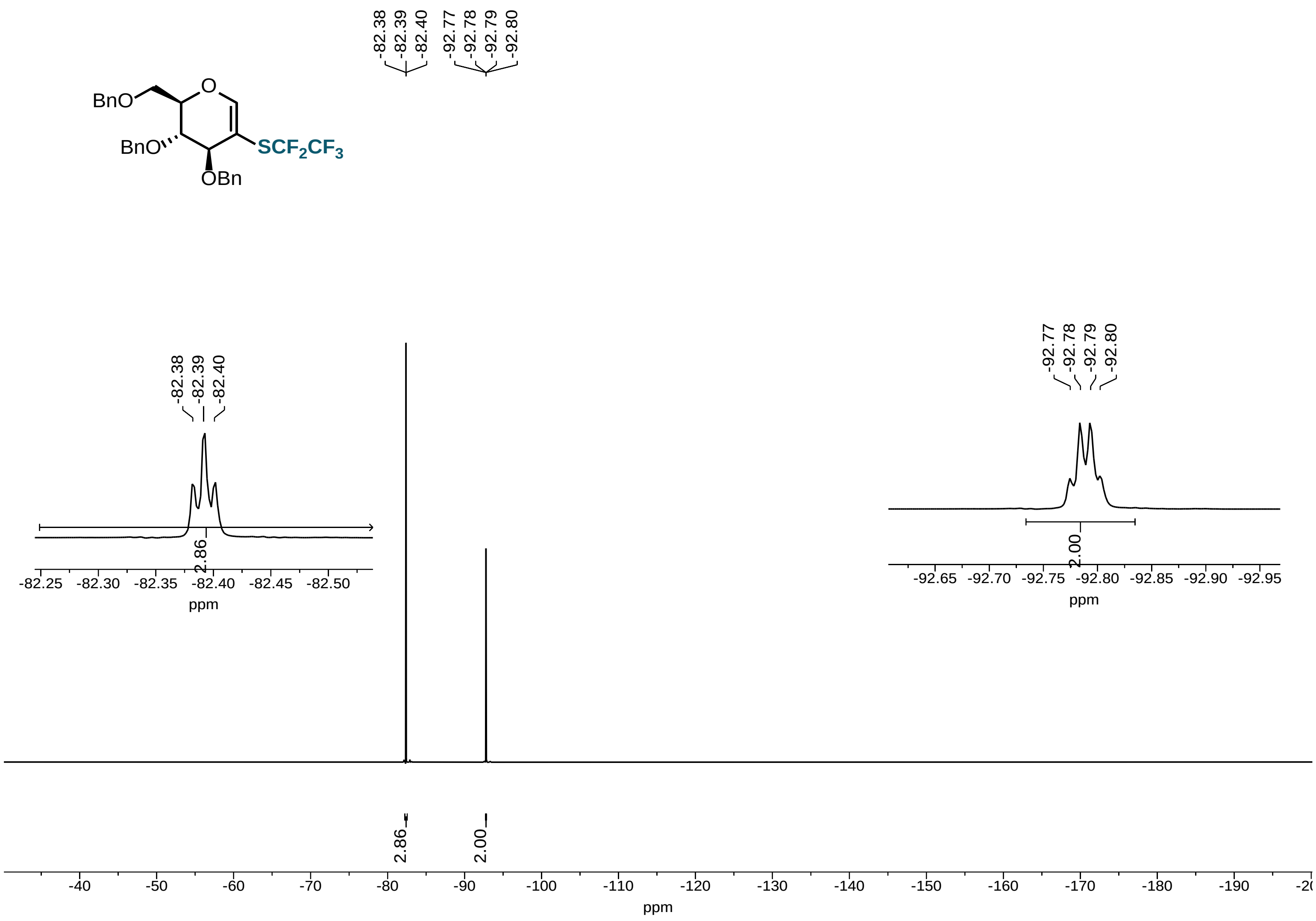

Figure S105. ^{19}F NMR ($CDCl_3$, 376.5 MHz) of **19b**

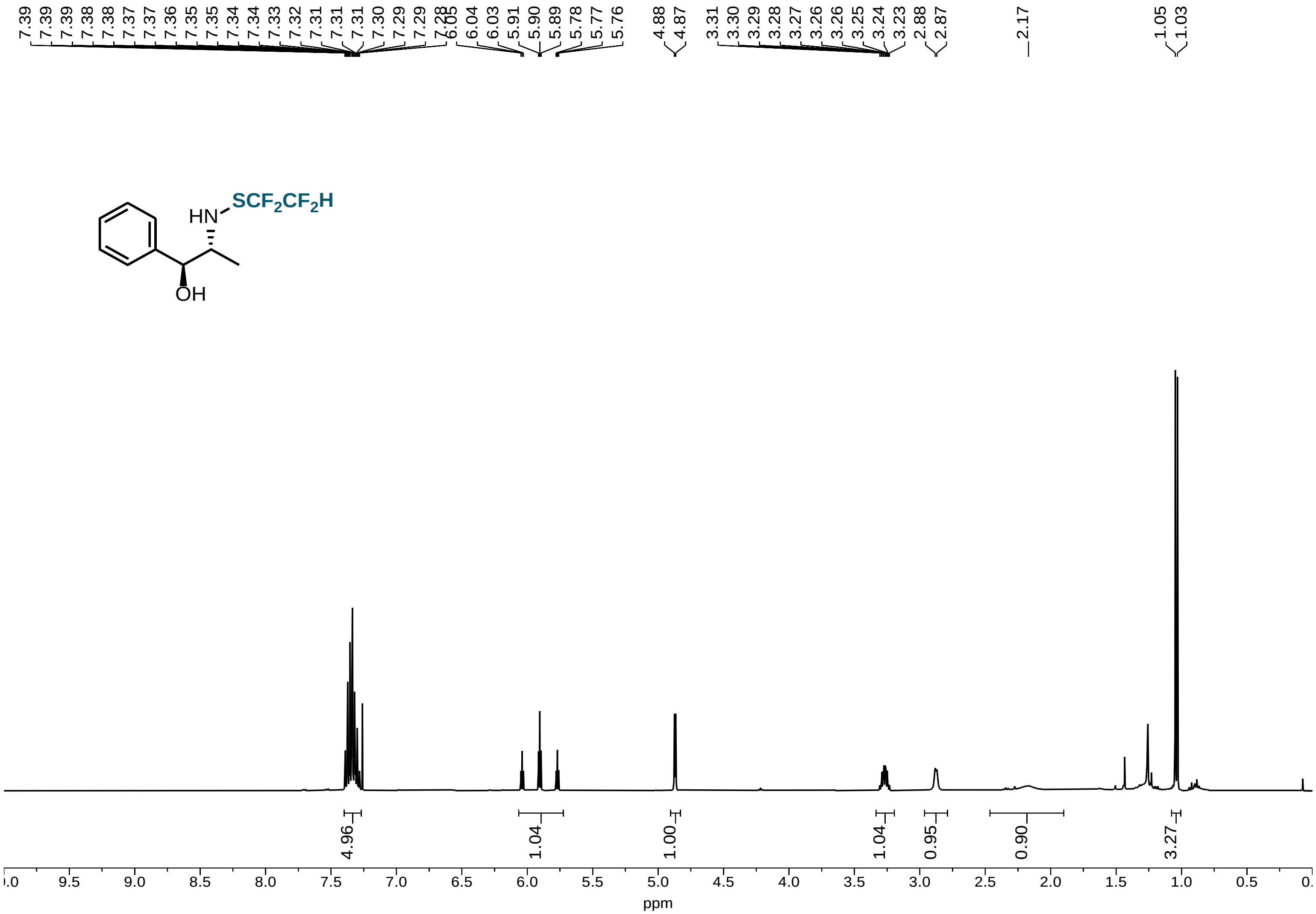

Figure S106. ^{1}H NMR ($CDCl_3$, 400 MHz) of **20a**

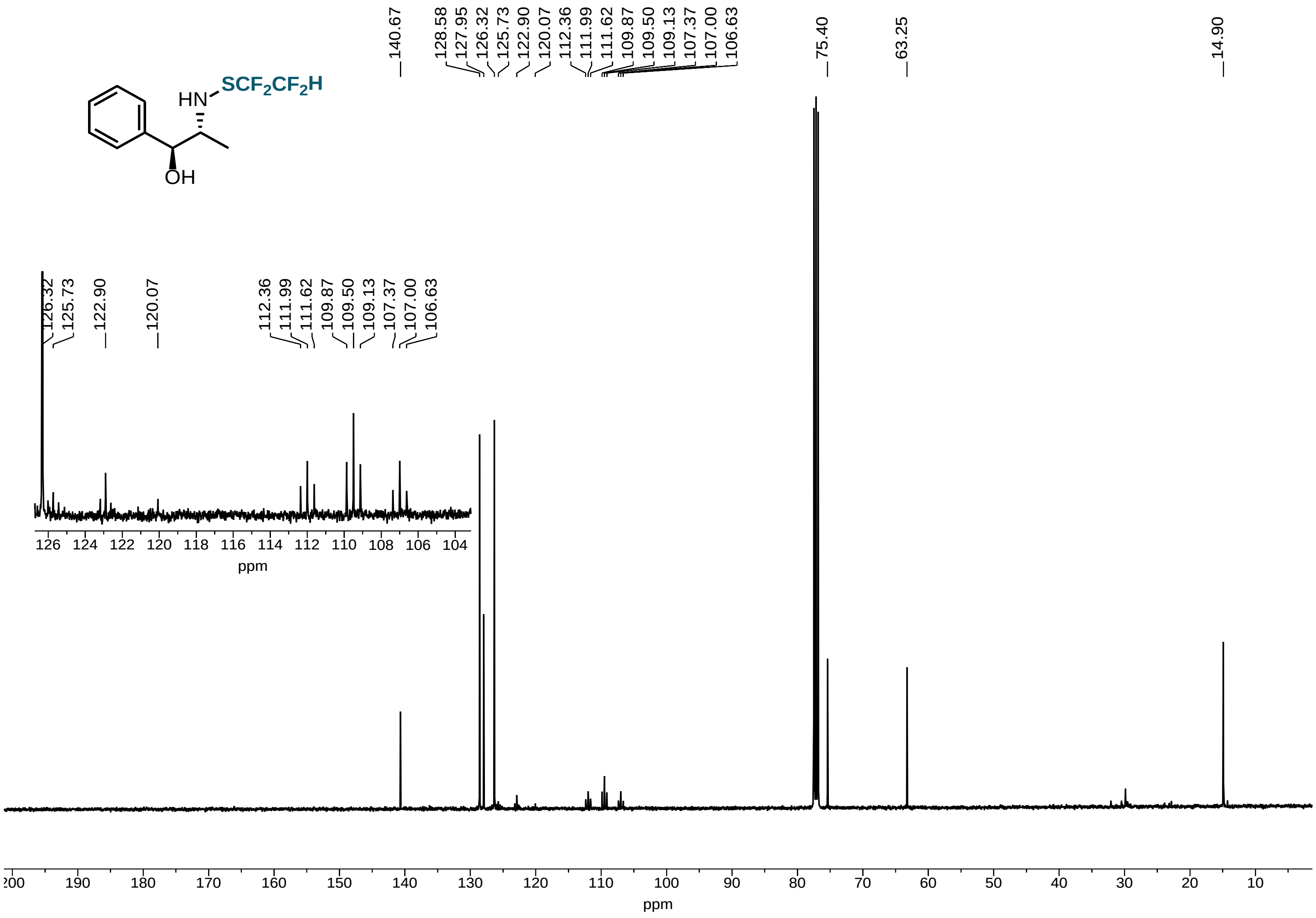

Figure S107. $^{13}C\{^{1}H\}$ NMR ($CDCl_3$, 100.6 MHz) of **20a**

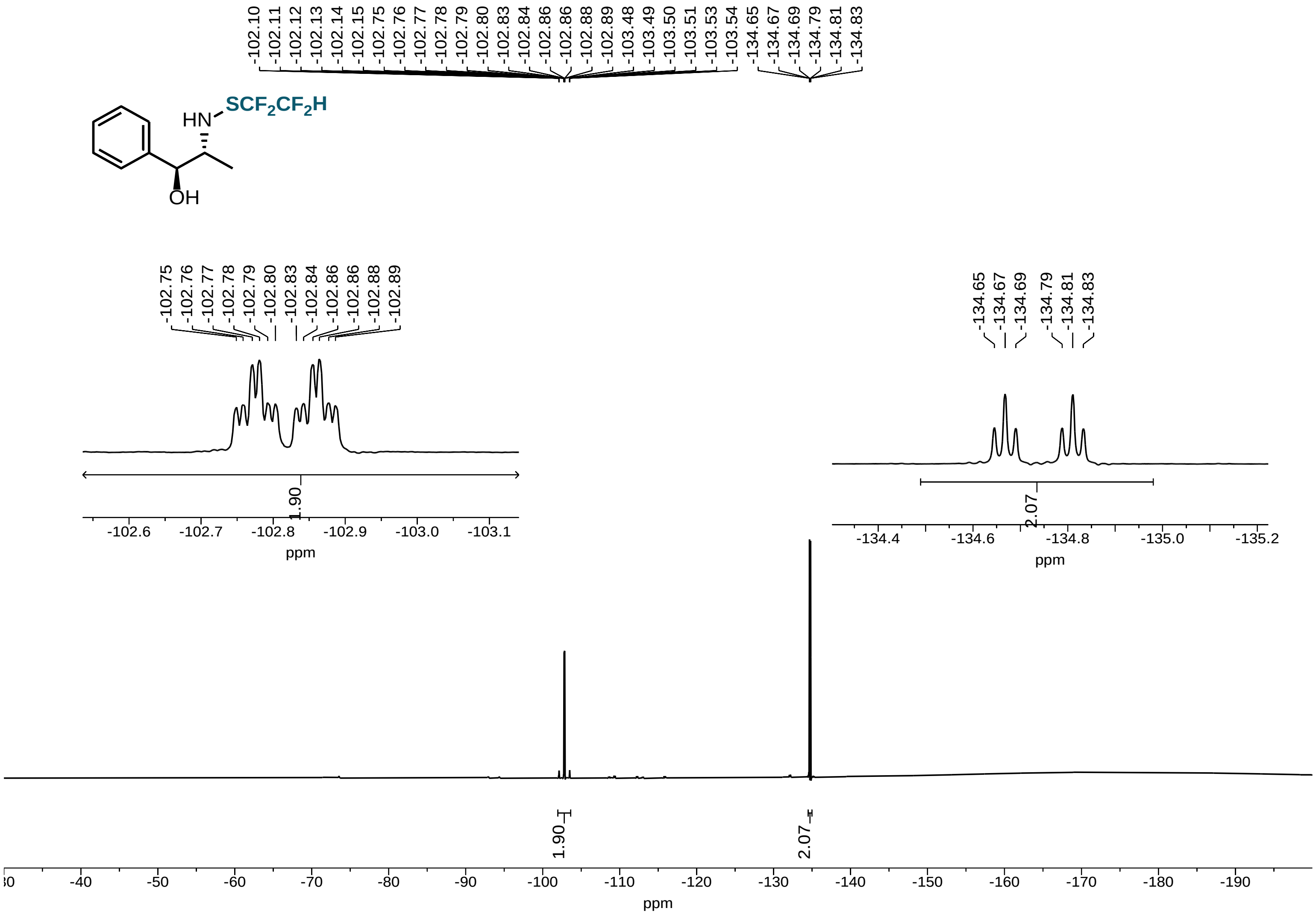

Figure S108. ^{19}F NMR ($CDCl_3$, 376.5 MHz) of **20a**

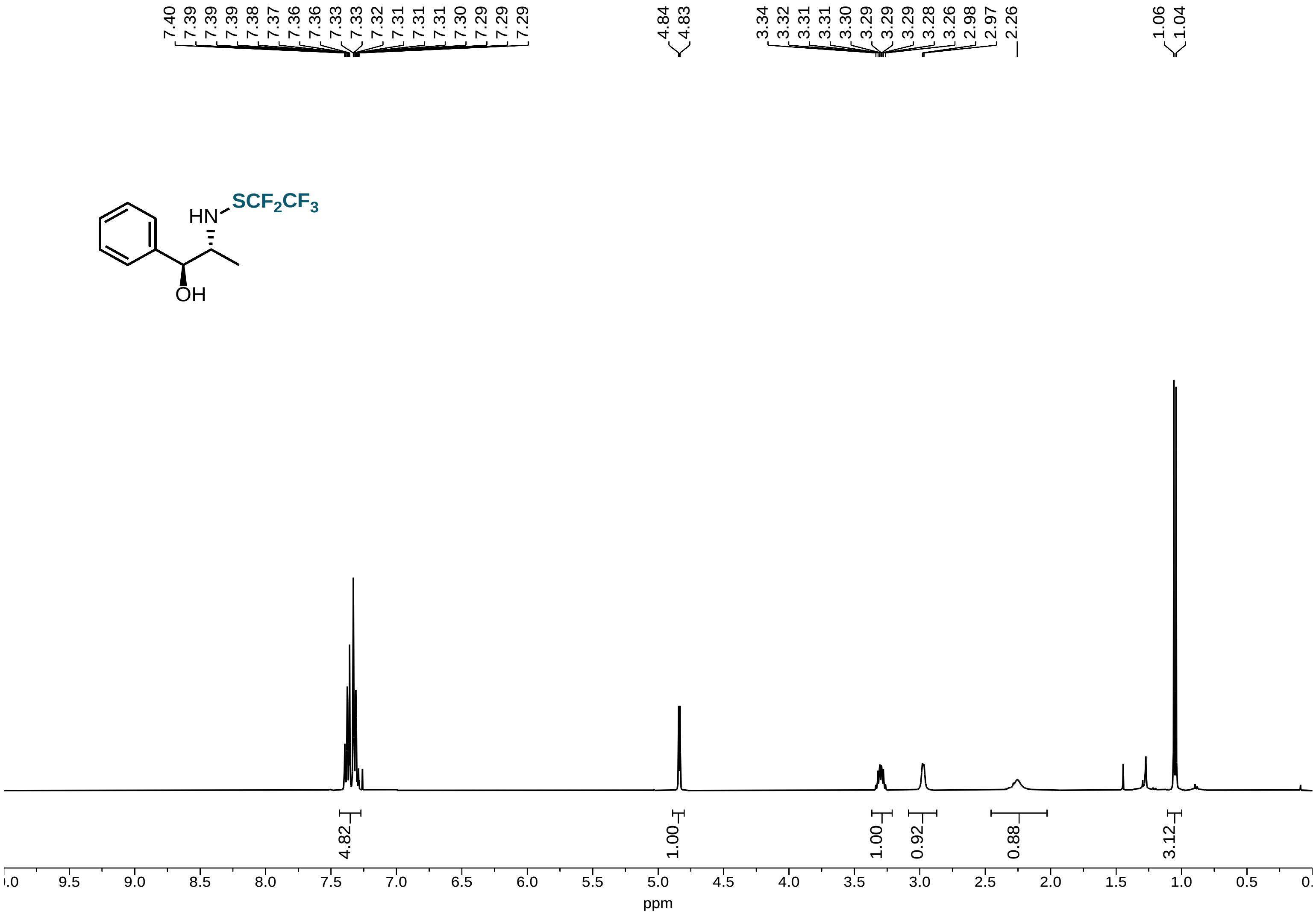

Figure S109. ^{1}H NMR ($CDCl_3$, 400 MHz) of **20b**

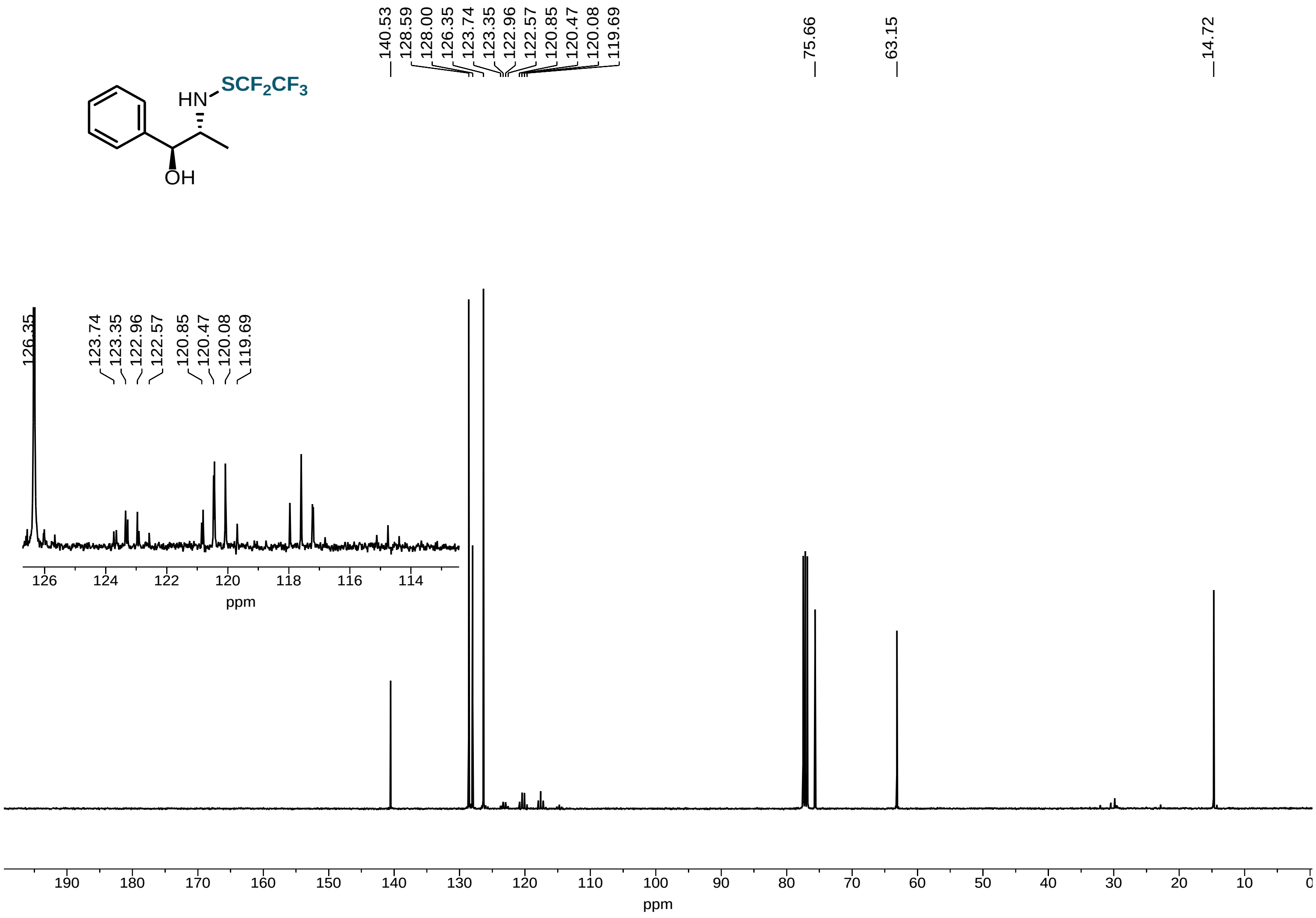

Figure S110. $^{13}C\{^1H\}$ NMR ($CDCl_3$, 100.6 MHz) of **20b**

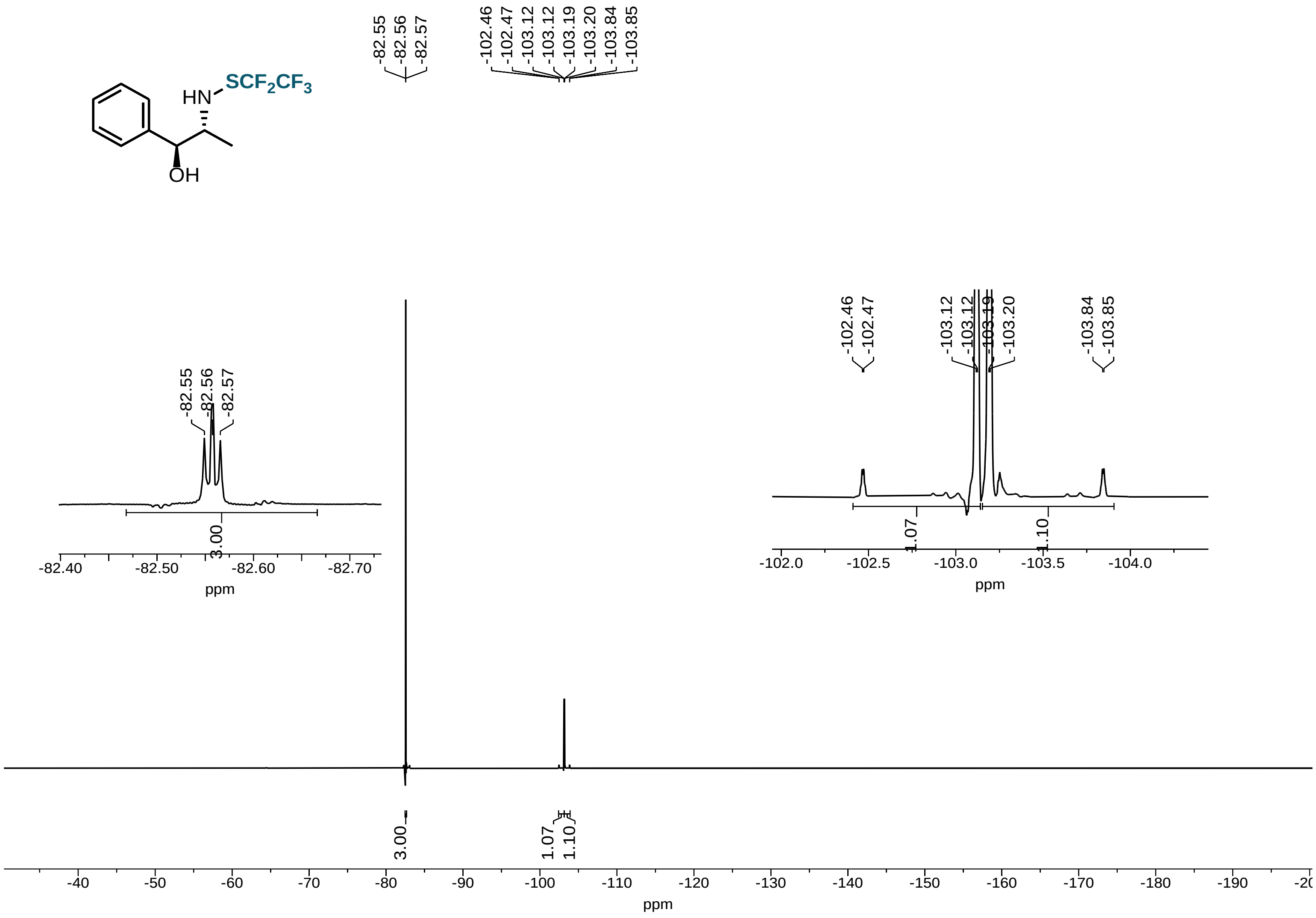

Figure S111. ^{19}F NMR ($CDCl_3$, 376.5 MHz) of **20b**

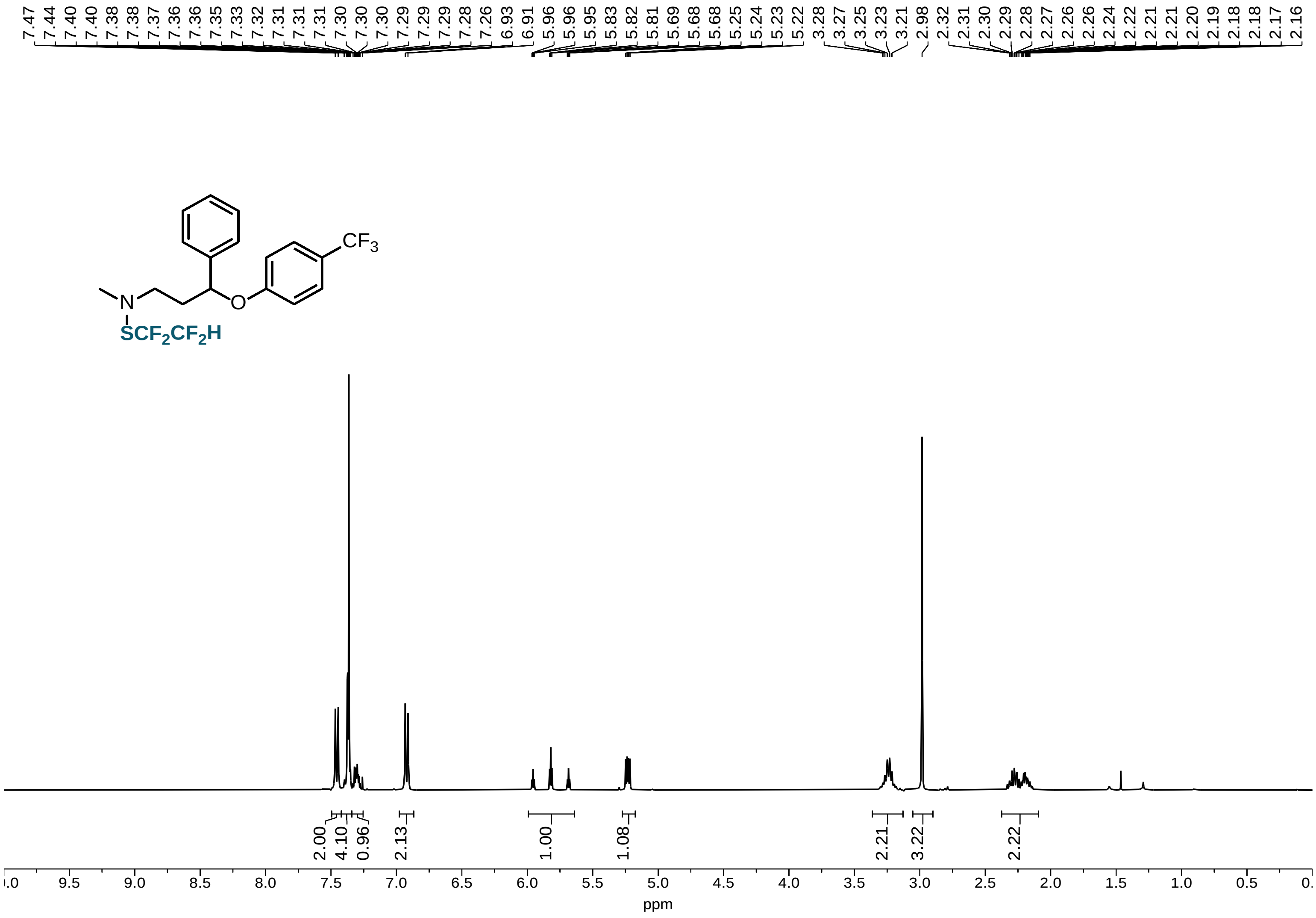

Figure S112. ^{1}H NMR ($CDCl_3$, 400 MHz) of **21a**

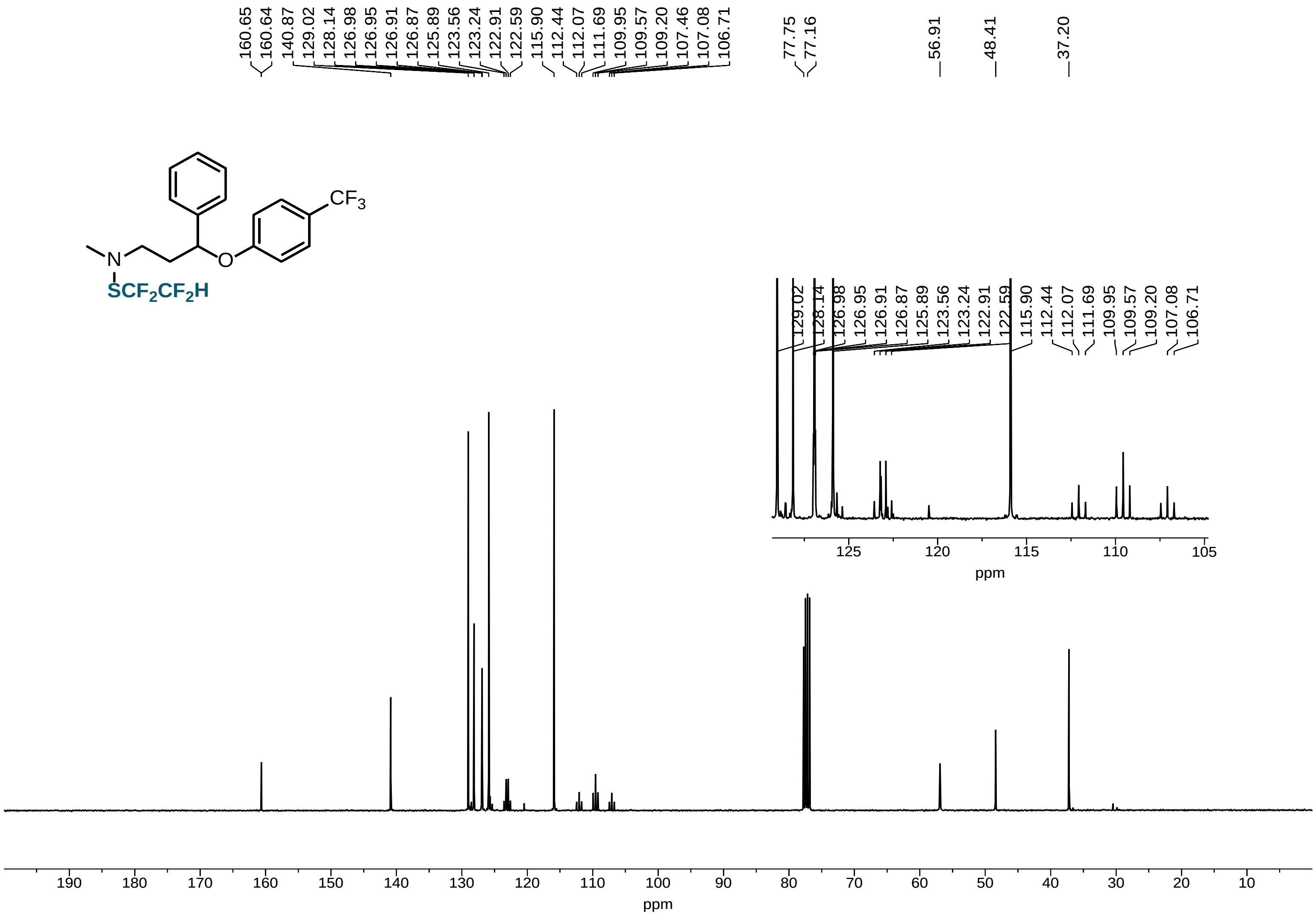

Figure S113. $^{13}C\{^{1}H\}$ NMR ($CDCl_3$, 100.6 MHz) of **21a**

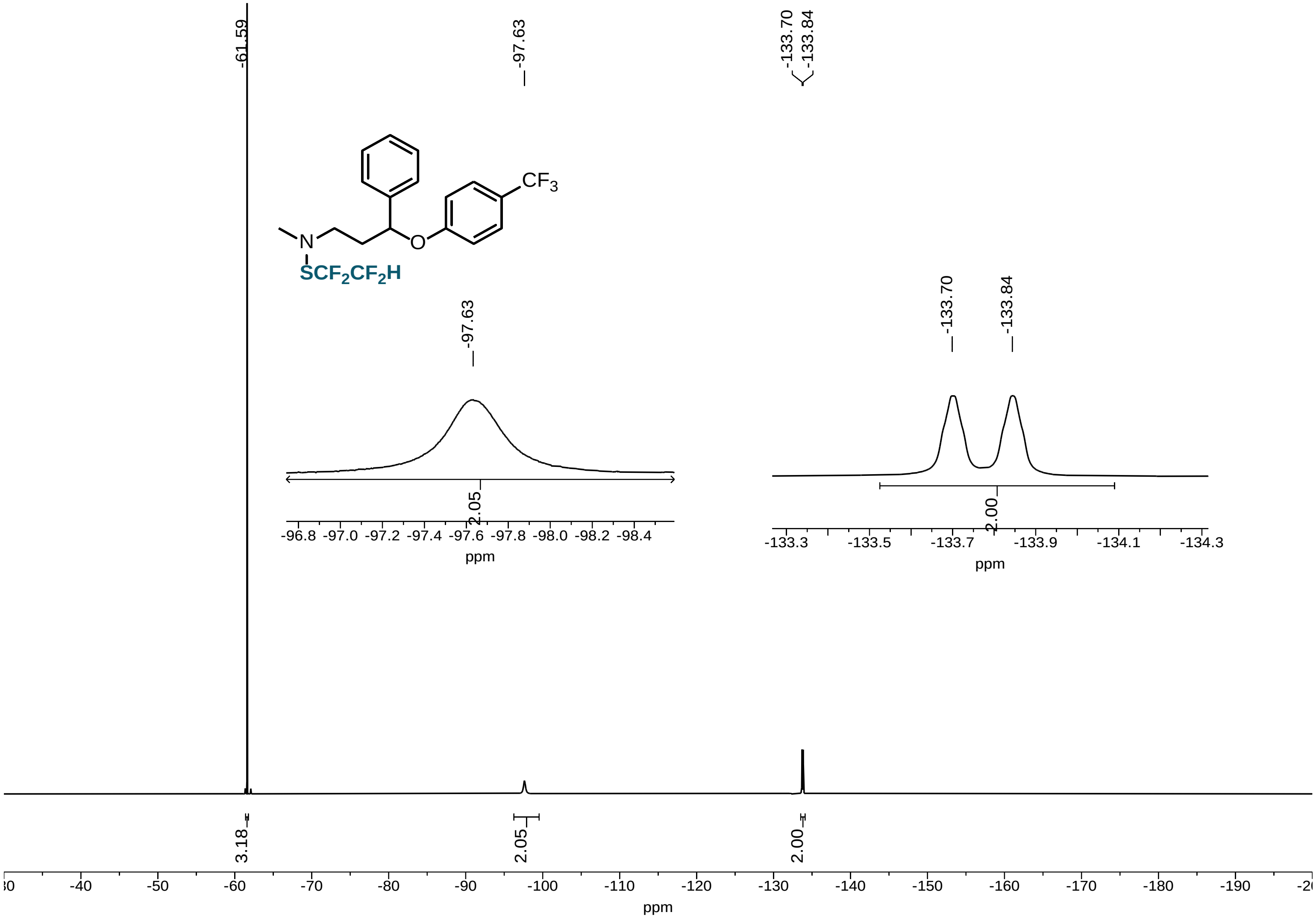

Figure S114. ^{19}F NMR ($CDCl_3$, 376.5 MHz) of **21a**

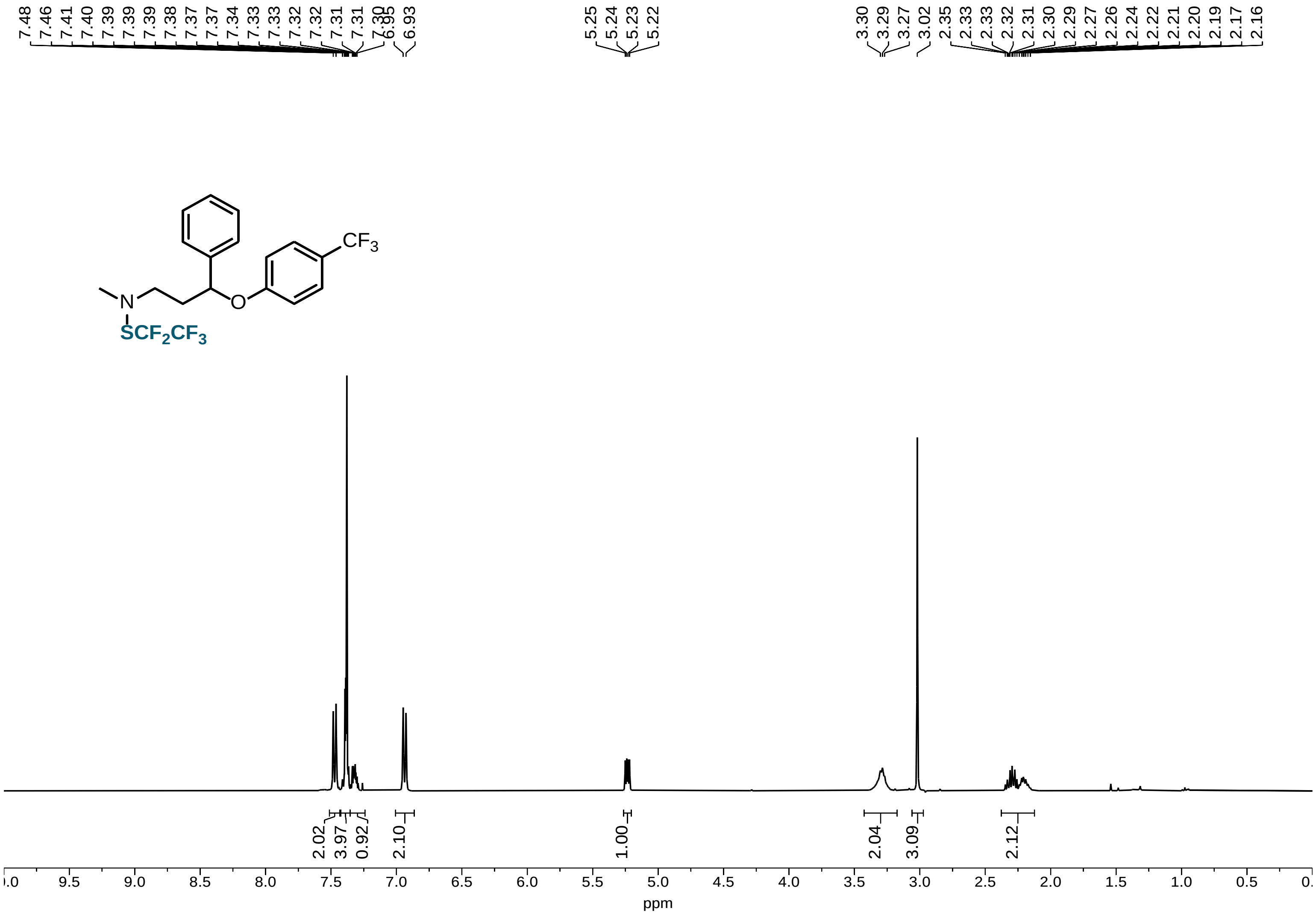

Figure S115. ^{1}H NMR ($CDCl_3$, 400 MHz) of **21b**

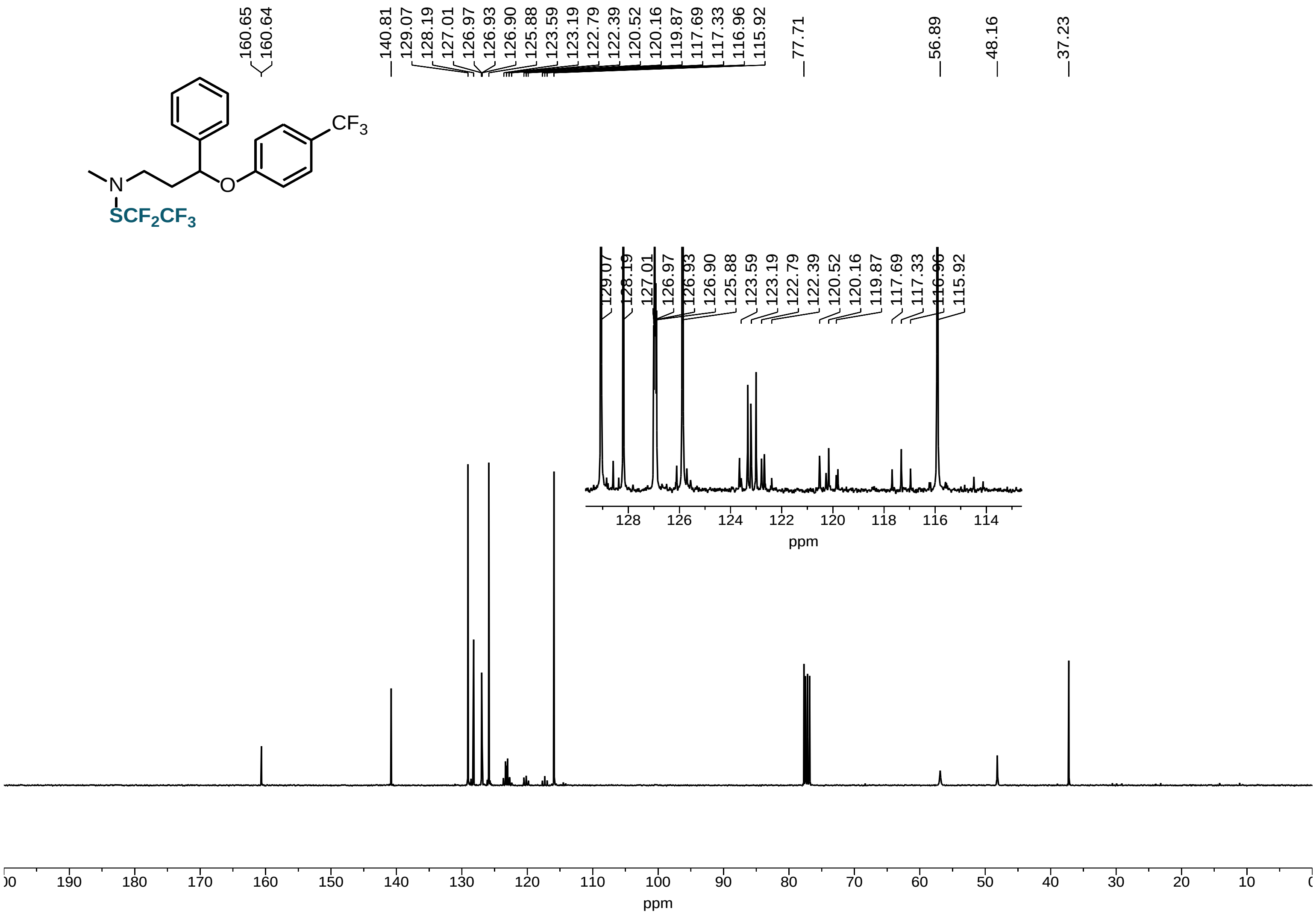

Figure S116. $^{13}C\{^1H\}$ NMR ($CDCl_3$, 100.6 MHz) of **21b**

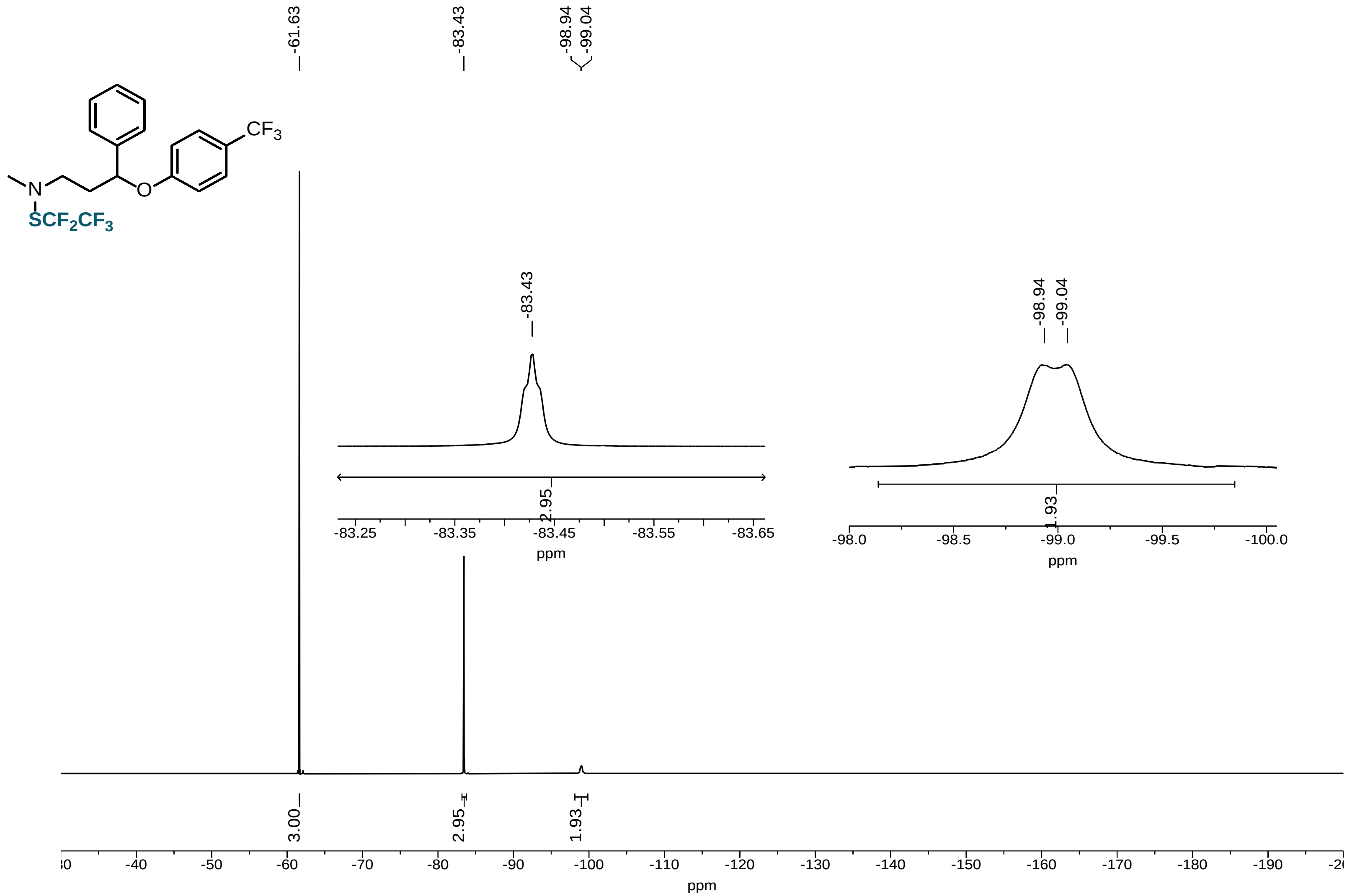

Figure S117. ^{19}F NMR ($CDCl_3$, 376.5 MHz) of **21b**

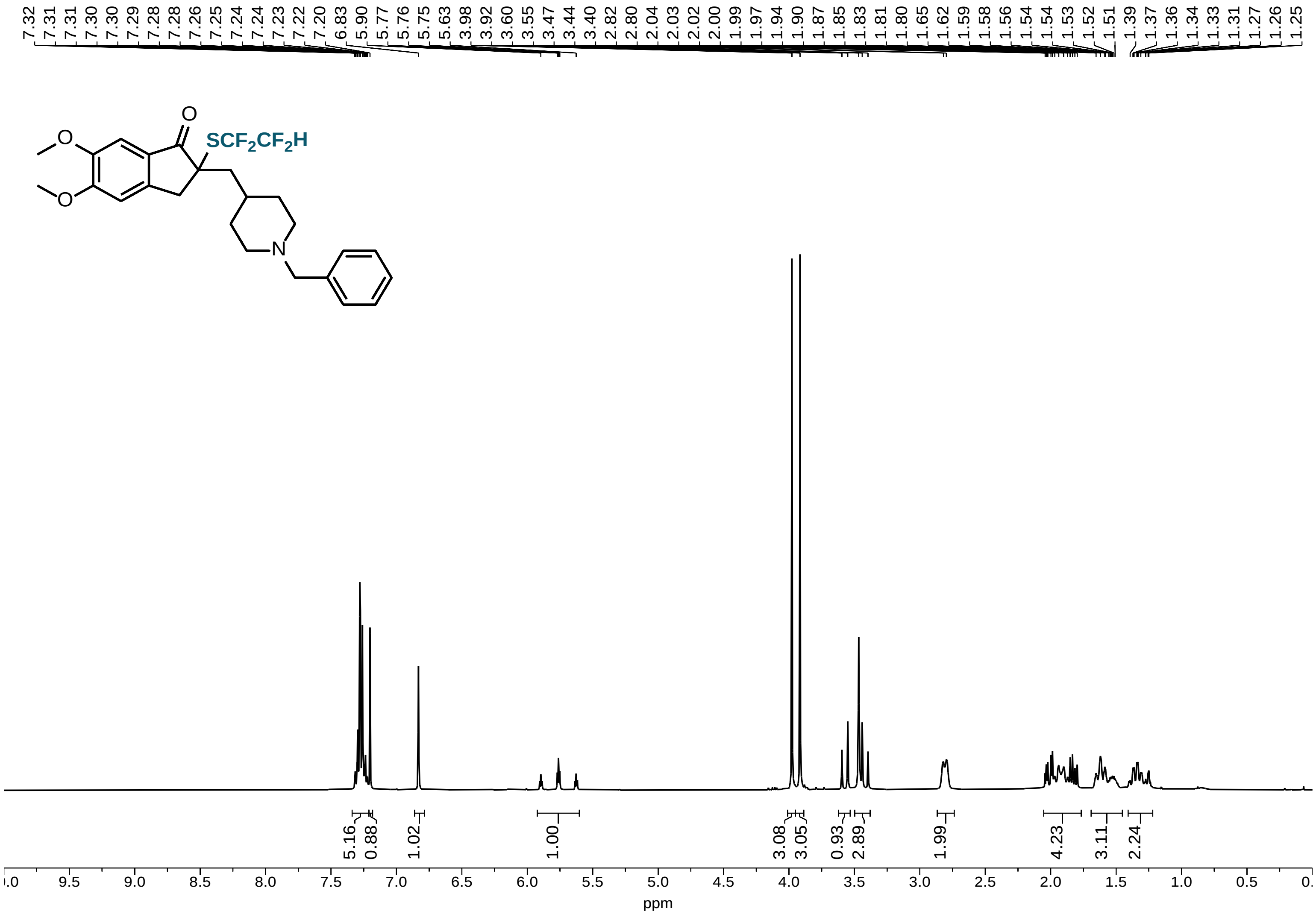

Figure S118. ^{1}H NMR ($CDCl_3$, 400 MHz) of **22a**

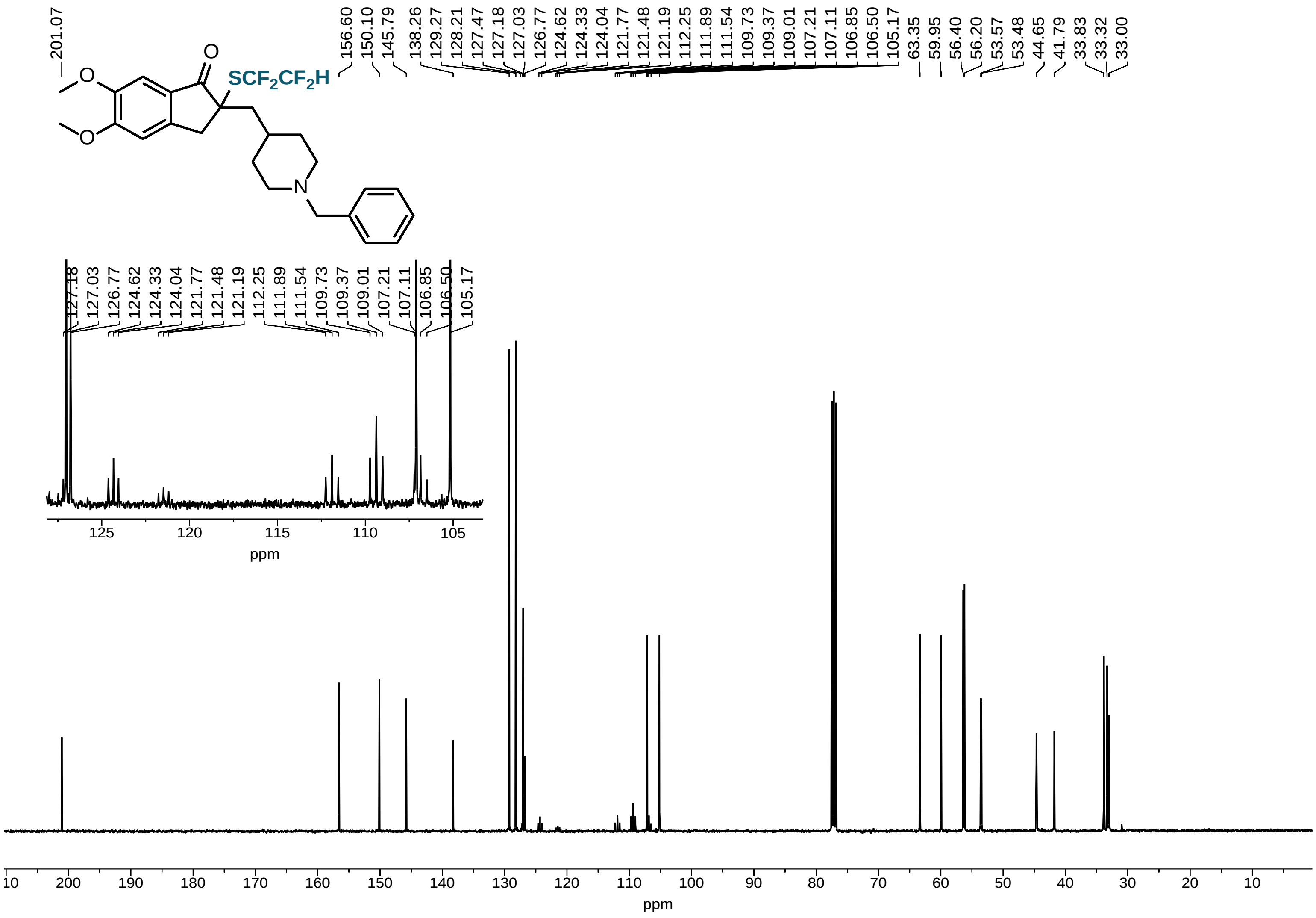

Figure S119. $^{13}C\{^1H\}$ NMR ($CDCl_3$, 100.6 MHz) of **22a**

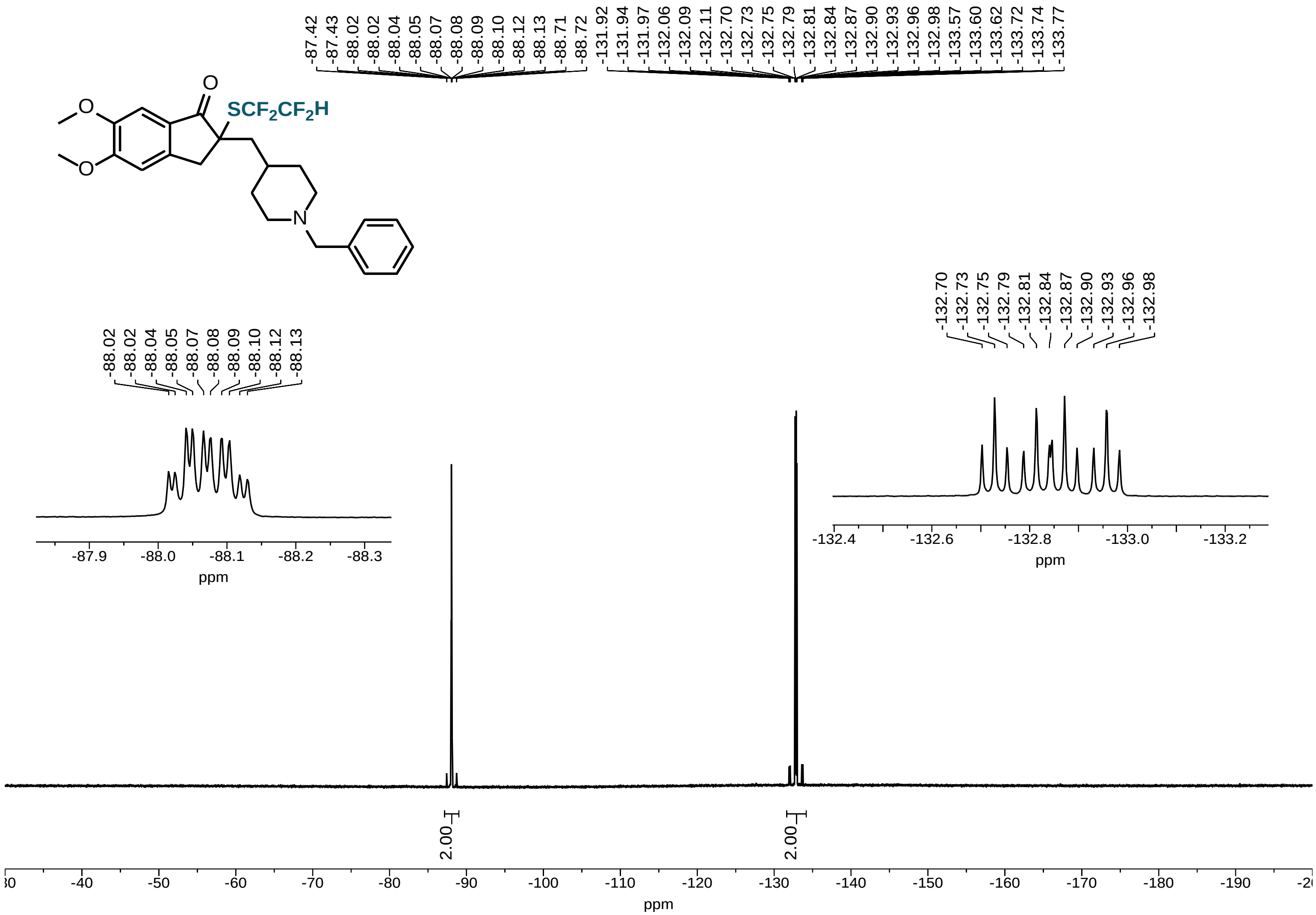

Figure S120. ^{19}F NMR ($CDCl_3$, 376.5 MHz) of **22a**

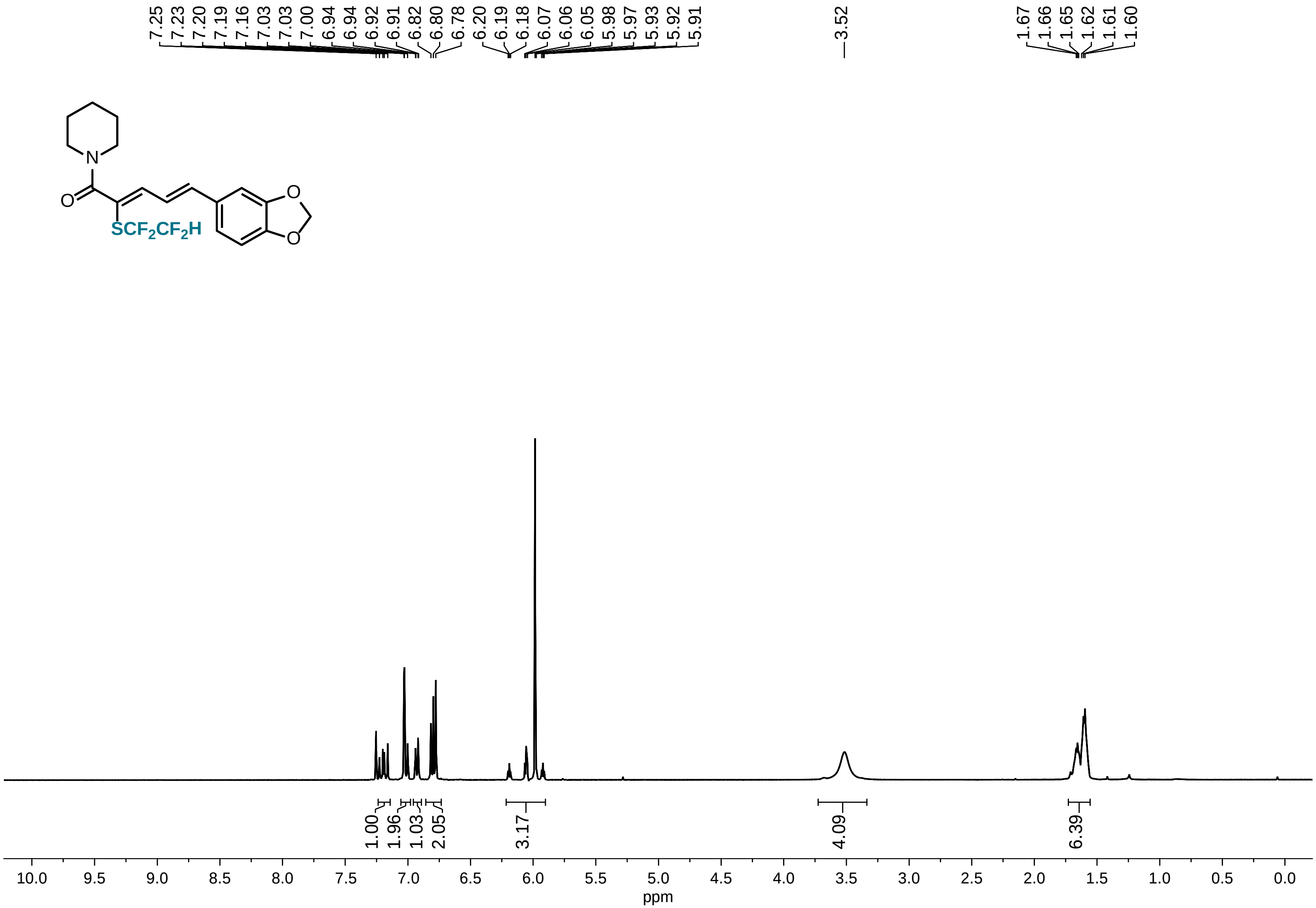

Figure S121. ^{1}H NMR ($CDCl_3$, 400 MHz) of **23a*E***

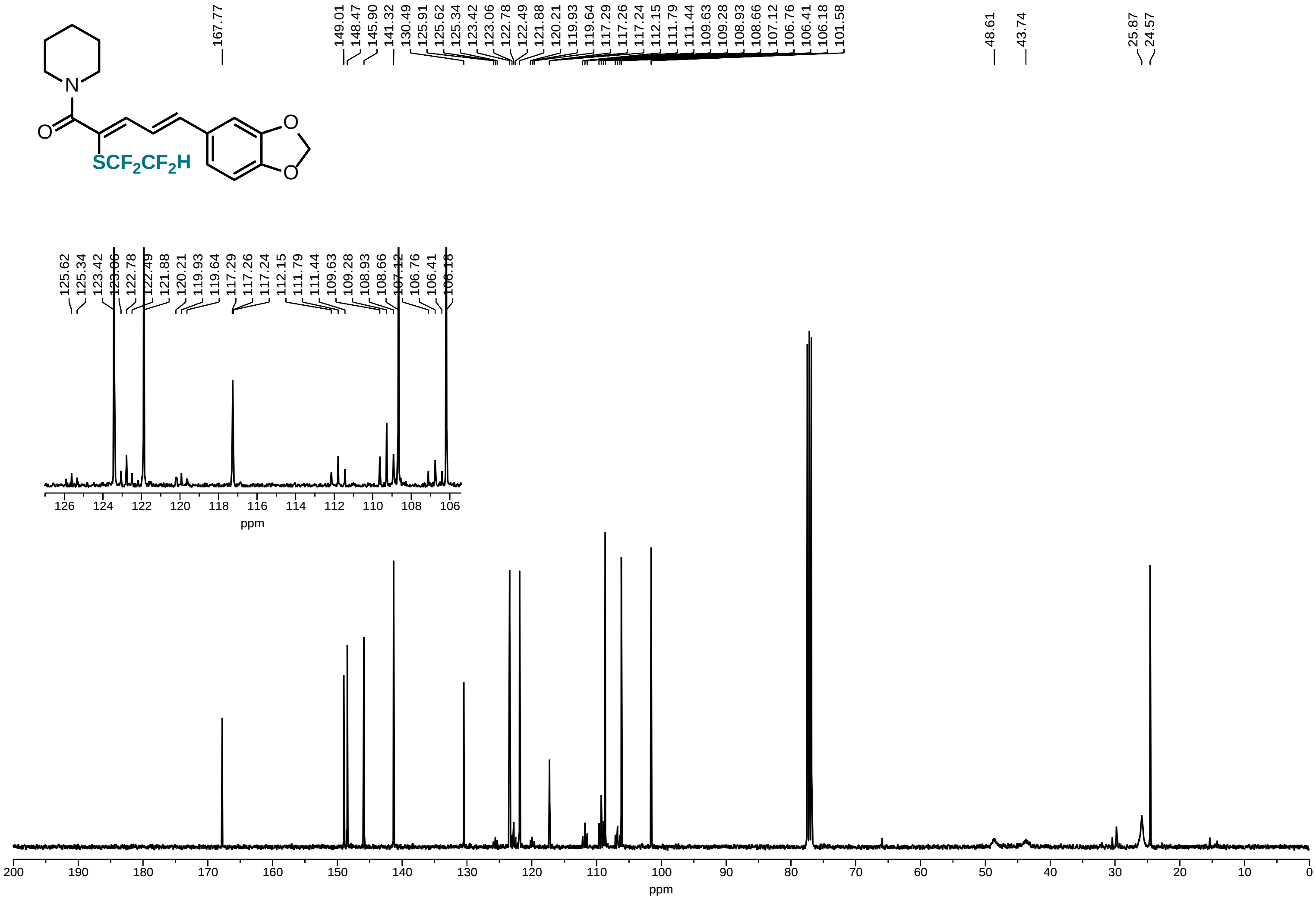

Figure S122. $^{13}C\{^{1}H\}$ NMR ($CDCl_3$, 100.6 MHz) of **23a*E***

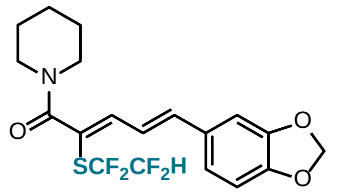

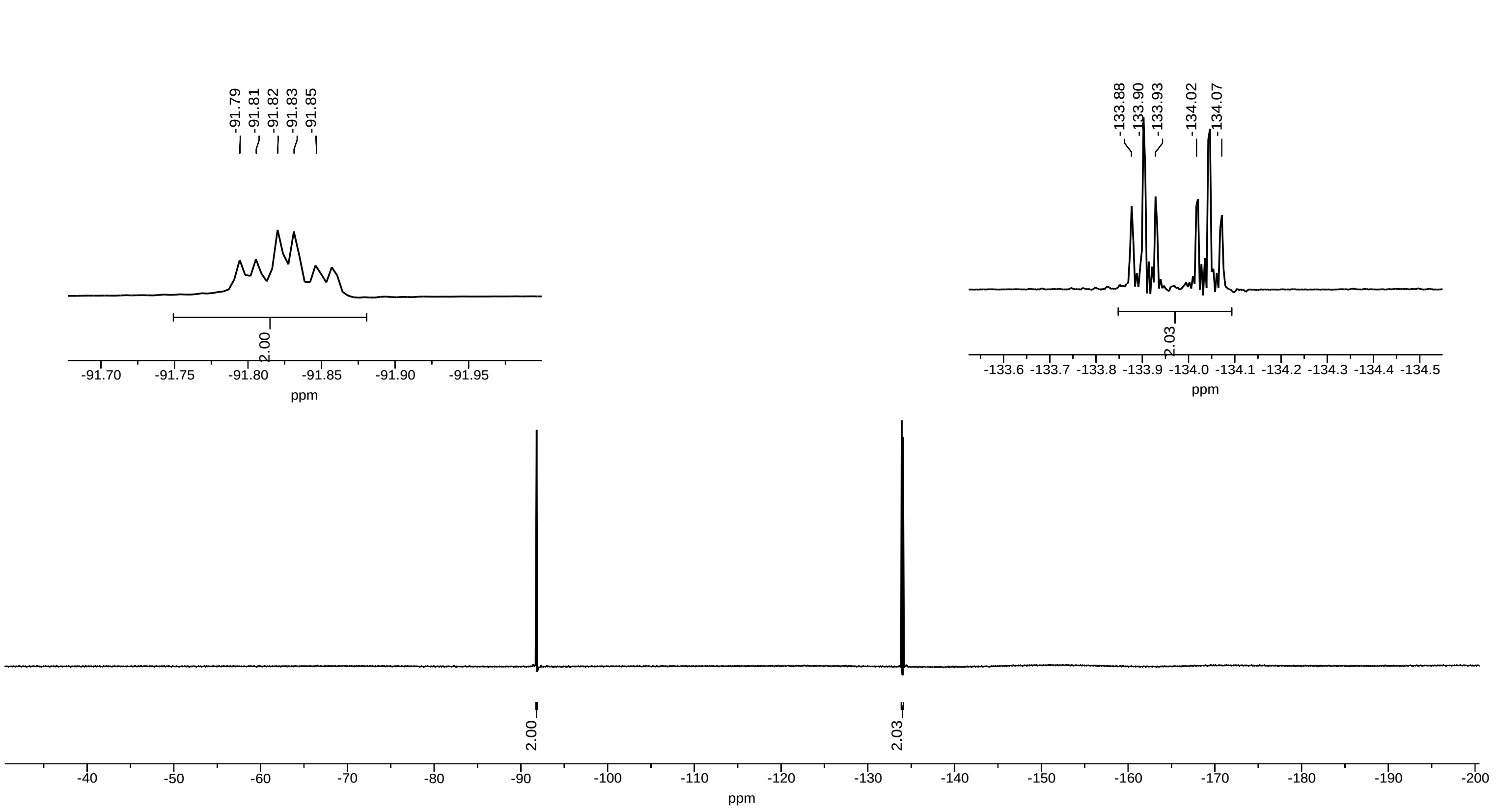

Figure S123. ^{19}F NMR ($CDCl_3$, 376.5 MHz) of **23a*E***

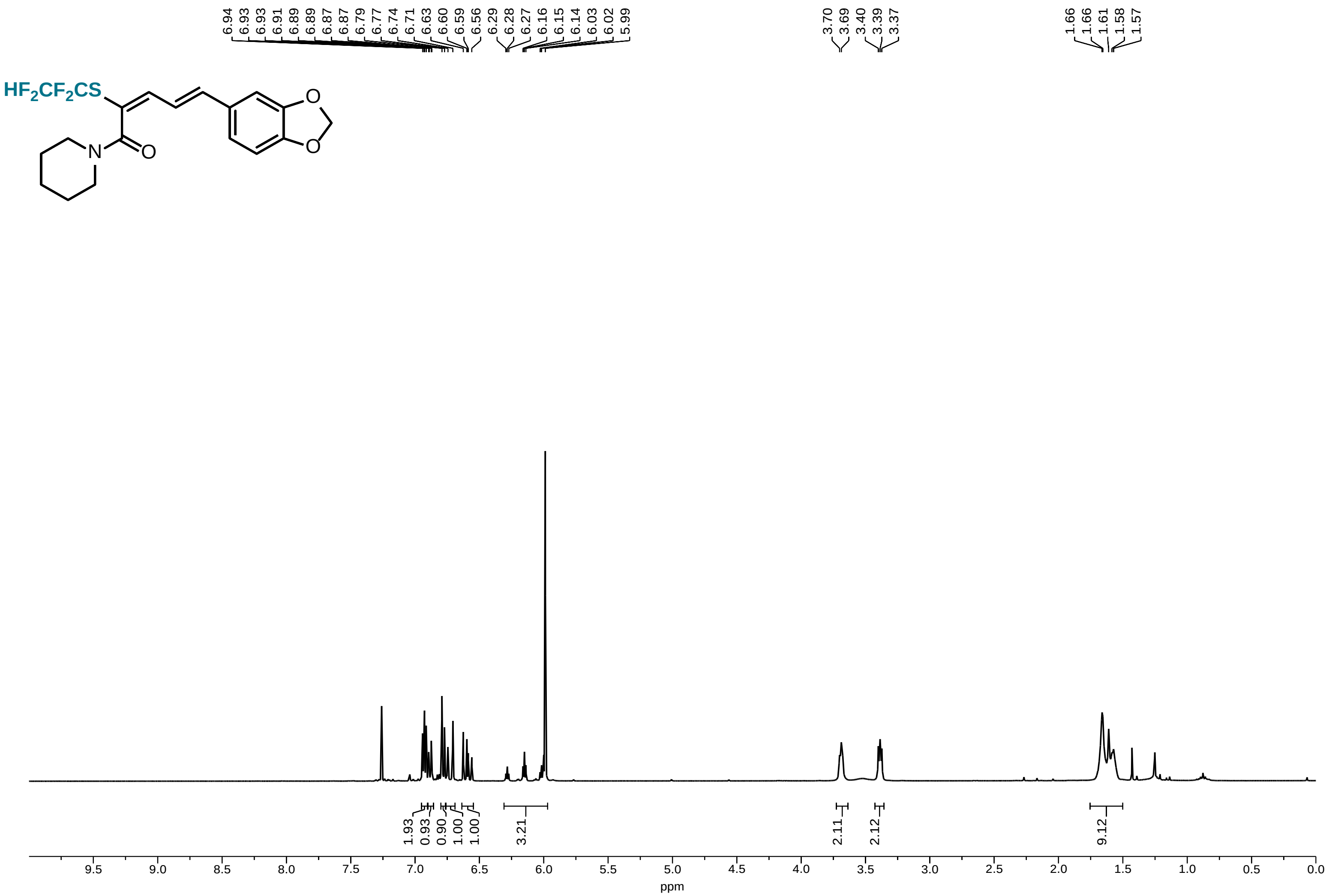

Figure S124. ^{1}H NMR ($CDCl_3$, 400 MHz) of **23a*Z***

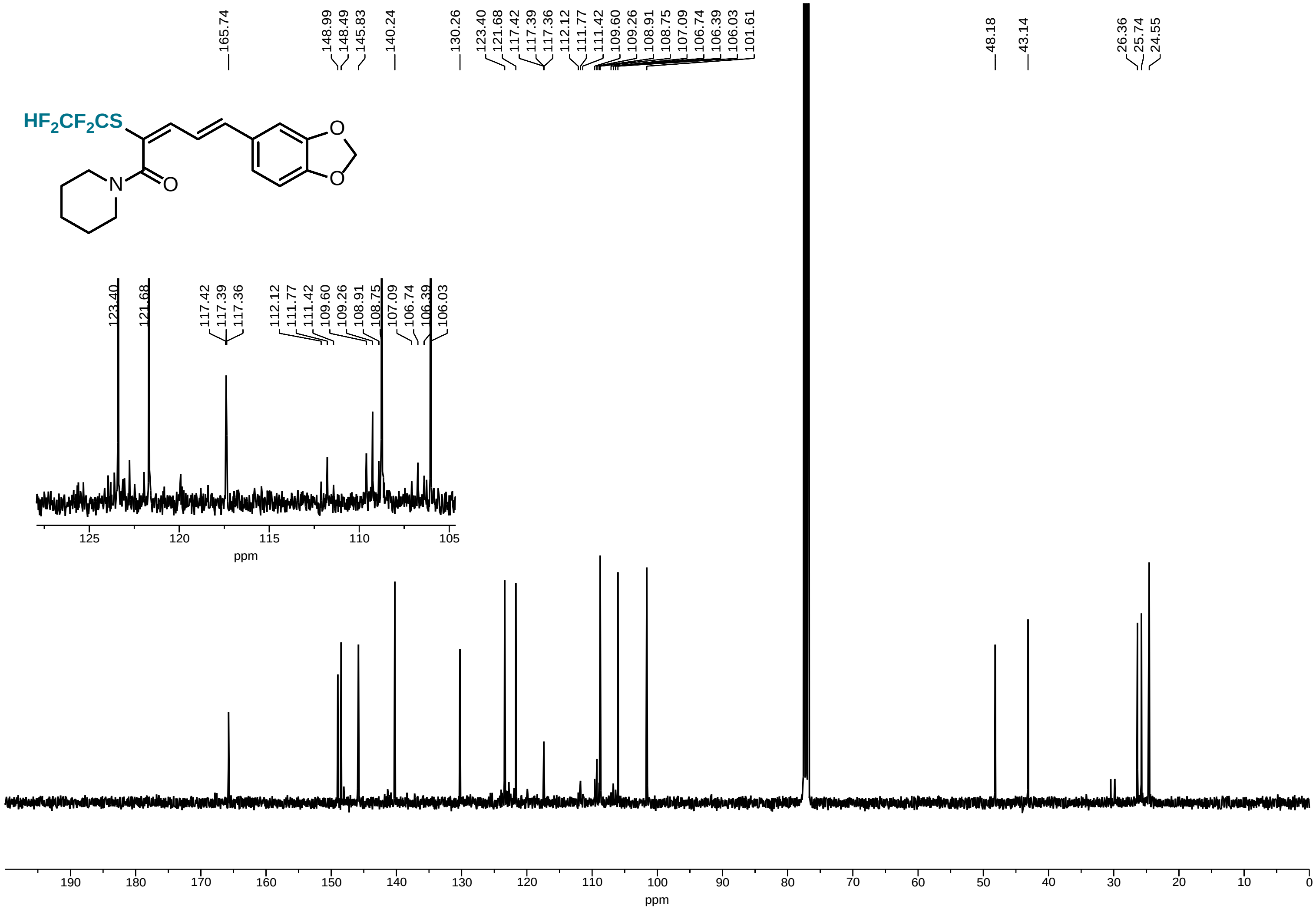

Figure S125. $^{13}C\{^1H\}$ NMR ($CDCl_3$, 100.6 MHz) of **23a*Z***

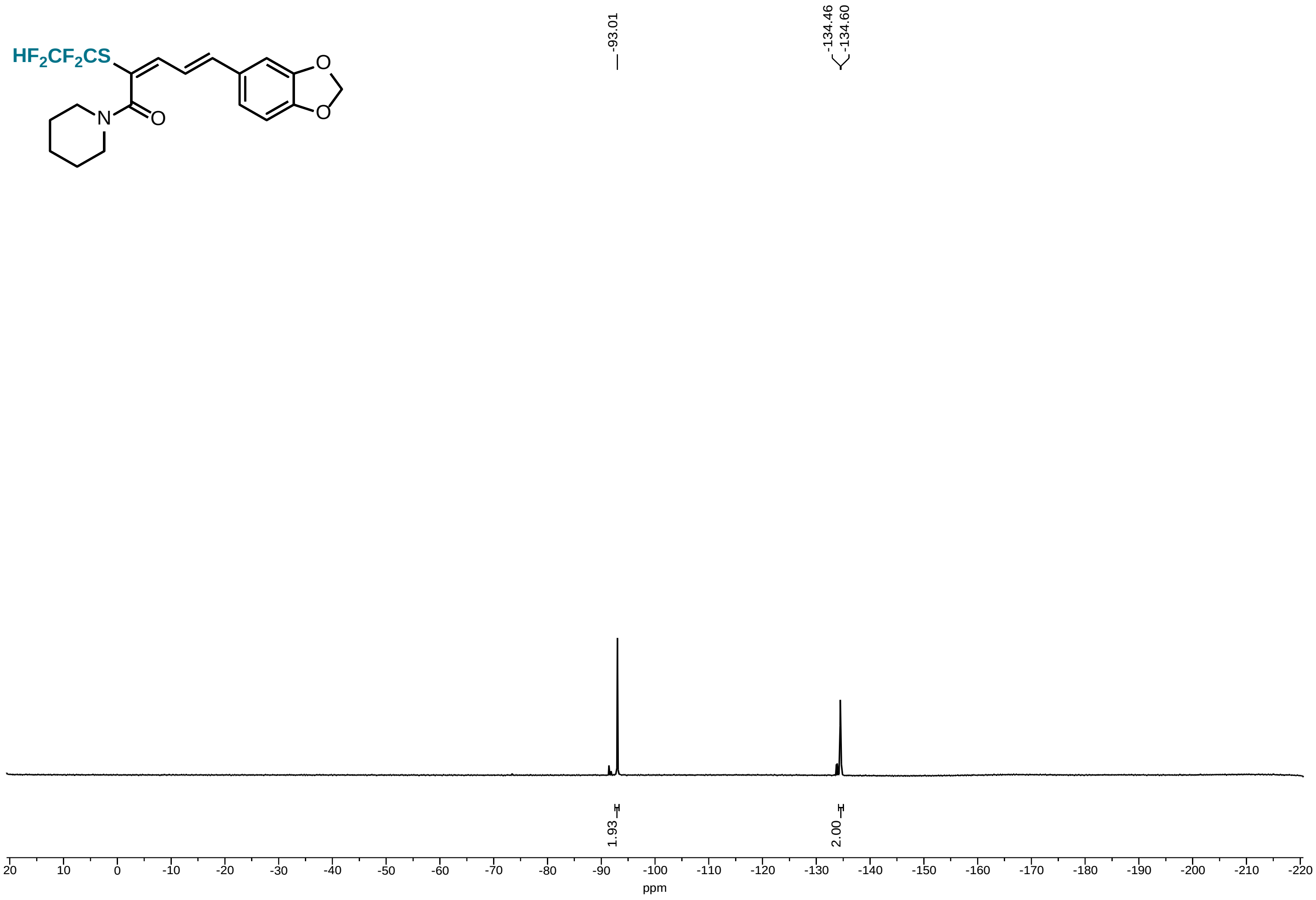

Figure S126. ^{19}F NMR ($CDCl_3$, 376.5 MHz) of **23a*E***

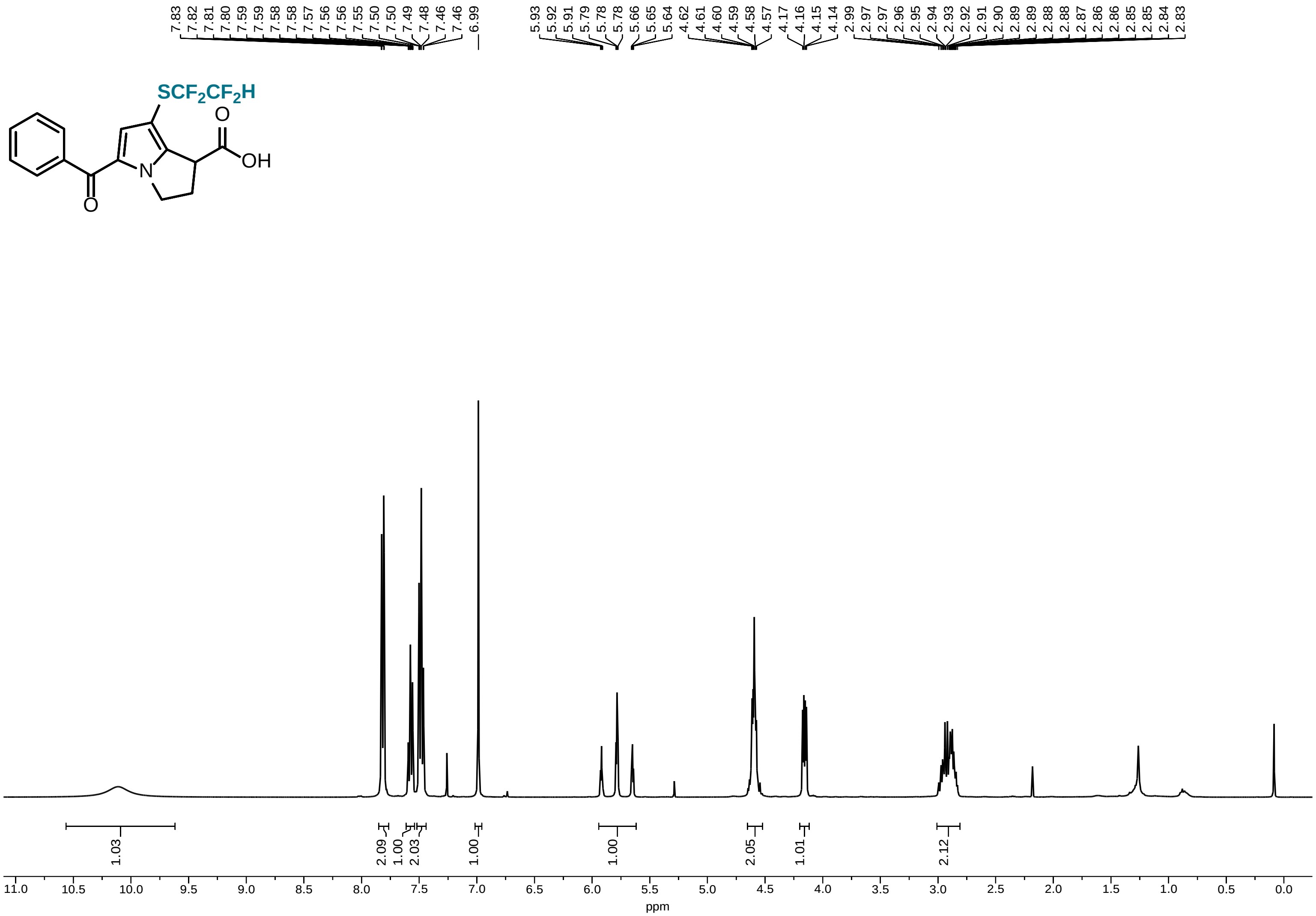

Figure S127. ^{1}H NMR ($CDCl_3$, 400 MHz) of **24a**

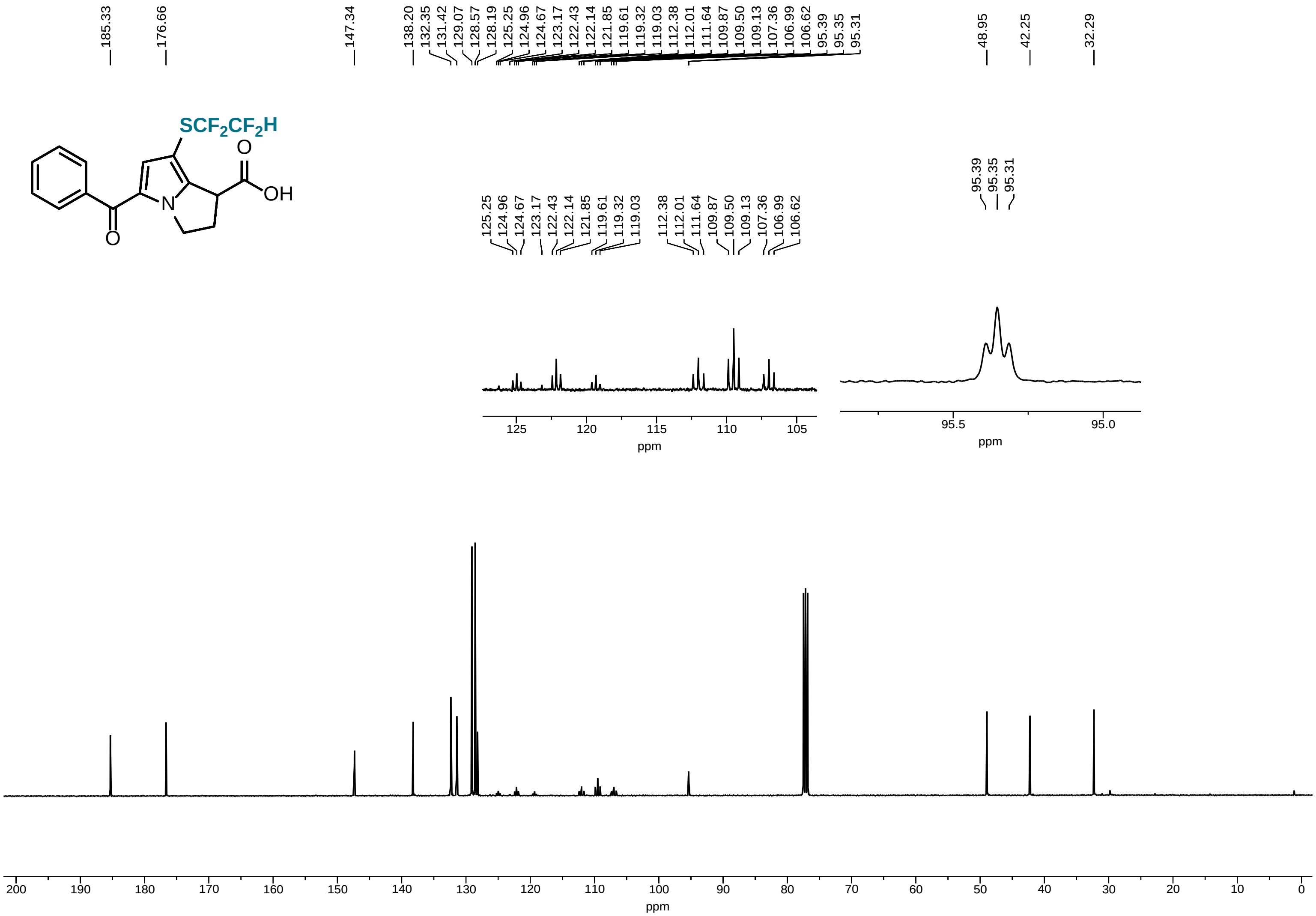

Figure S128. $^{13}C\{^1H\}$ NMR ($CDCl_3$, 100.6 MHz) of **24a**

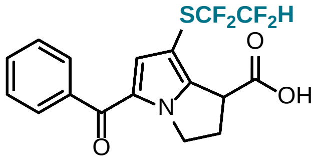

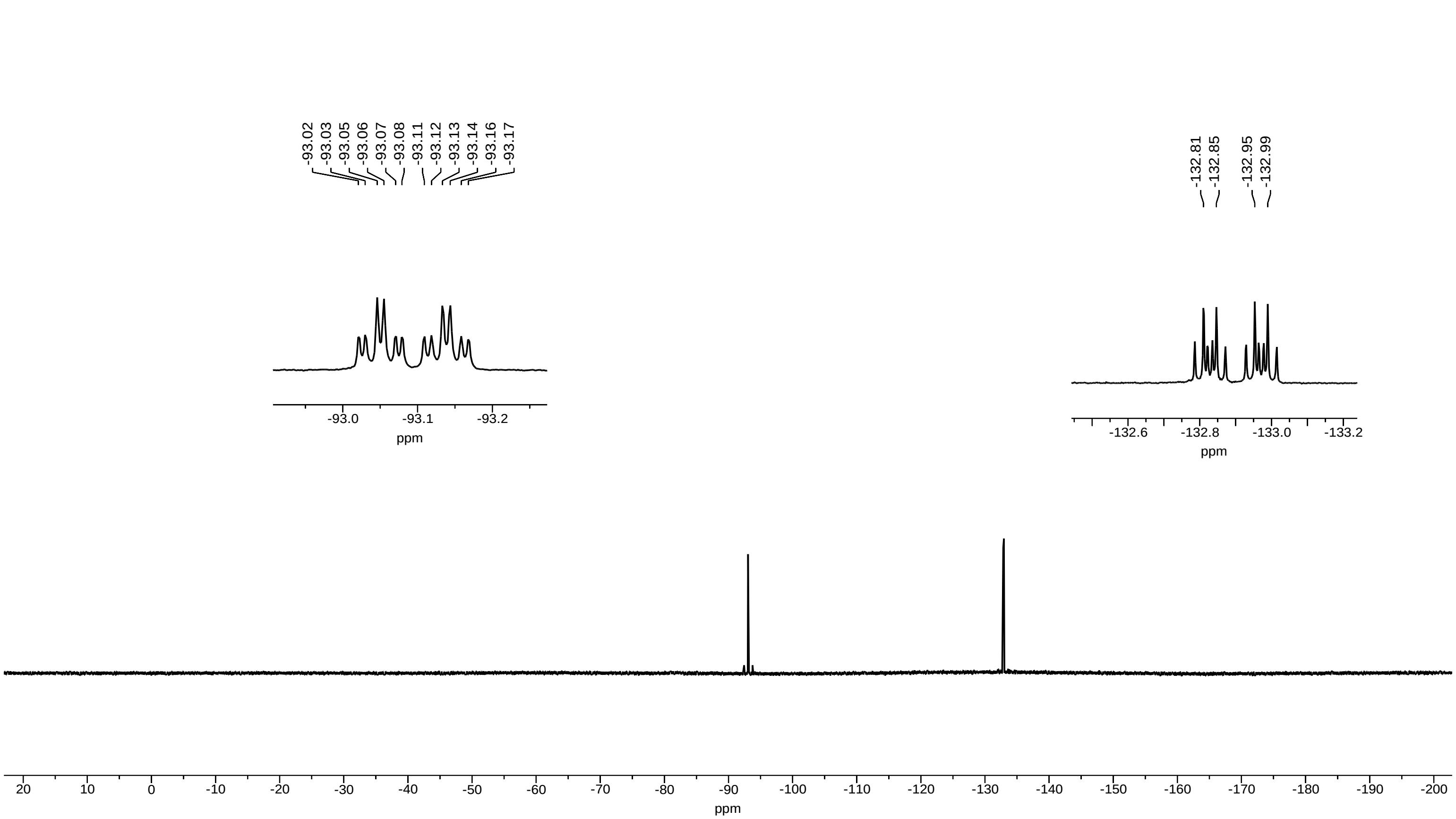

Figure S129. ^{19}F NMR ($CDCl_3$, 376.5 MHz) of **24a**

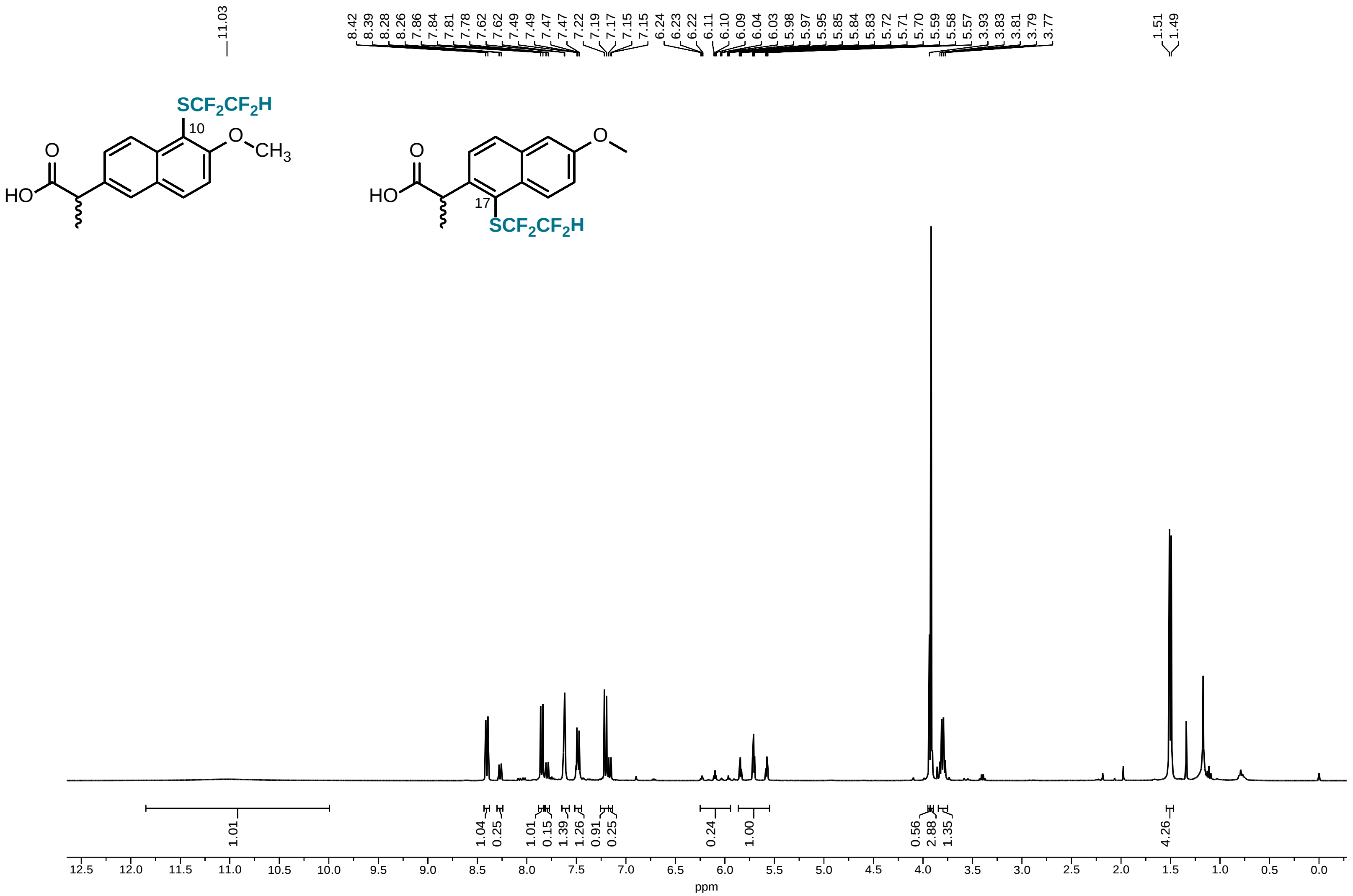

Figure S130. ^{1}H NMR ($CDCl_3$, 400 MHz) of **25a** and **25a'**

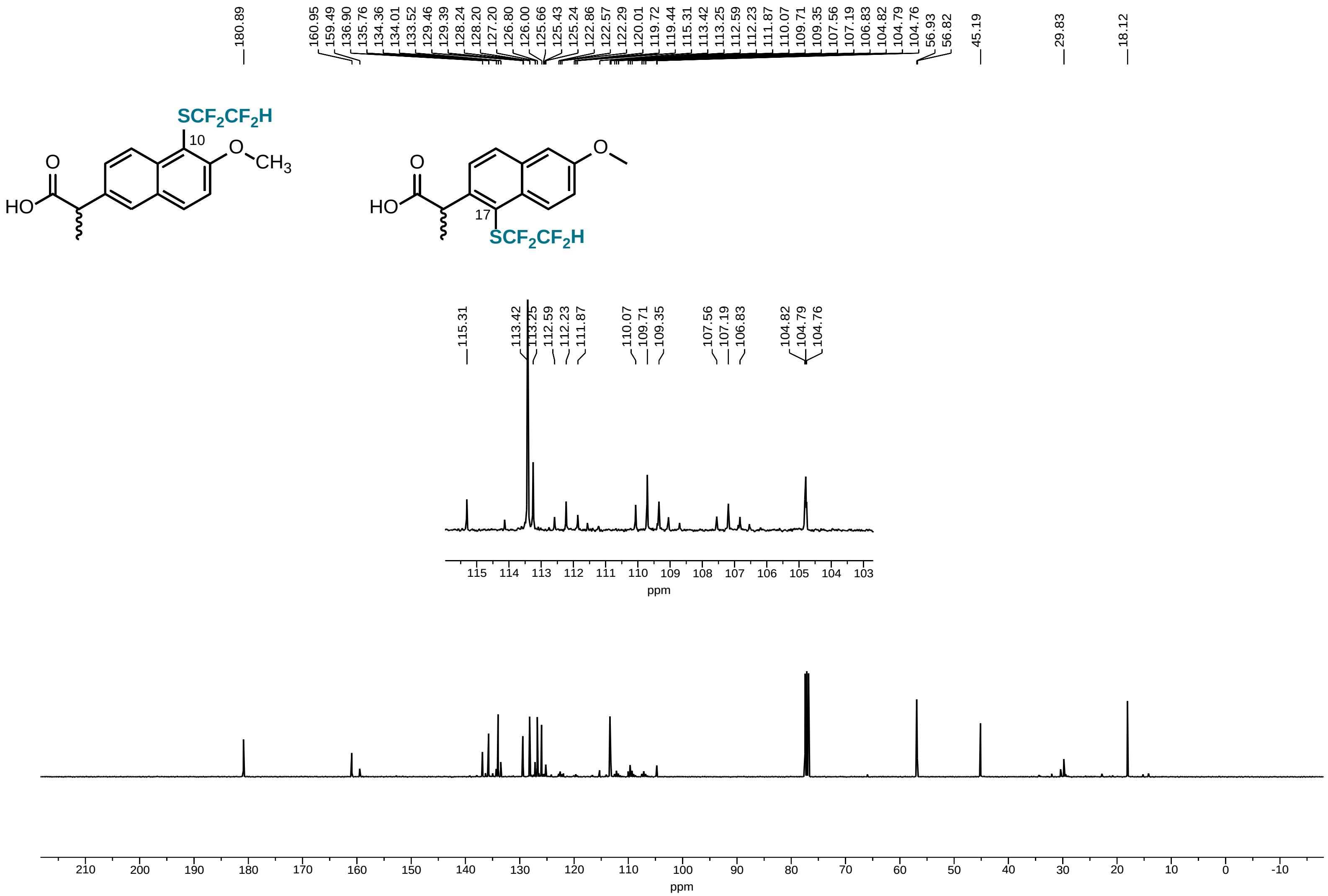

Figure S131. $^{13}C\{^1H\}$ NMR ($CDCl_3$, 100.6 MHz) of **25a** and **25a’**

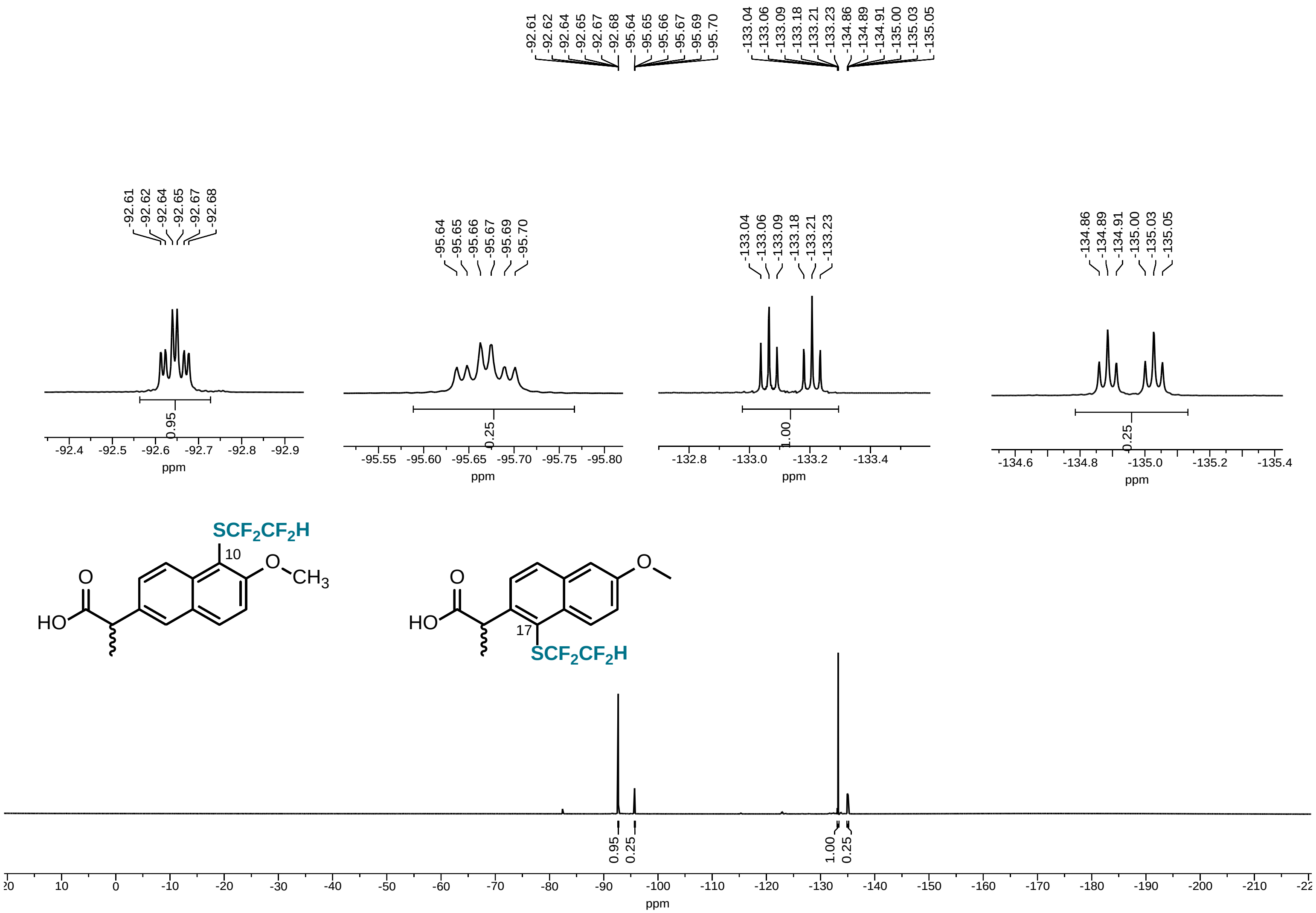

Figure S132. ^{19}F NMR ($CDCl_3$, 376.5 MHz) of **25a** and **25a’**

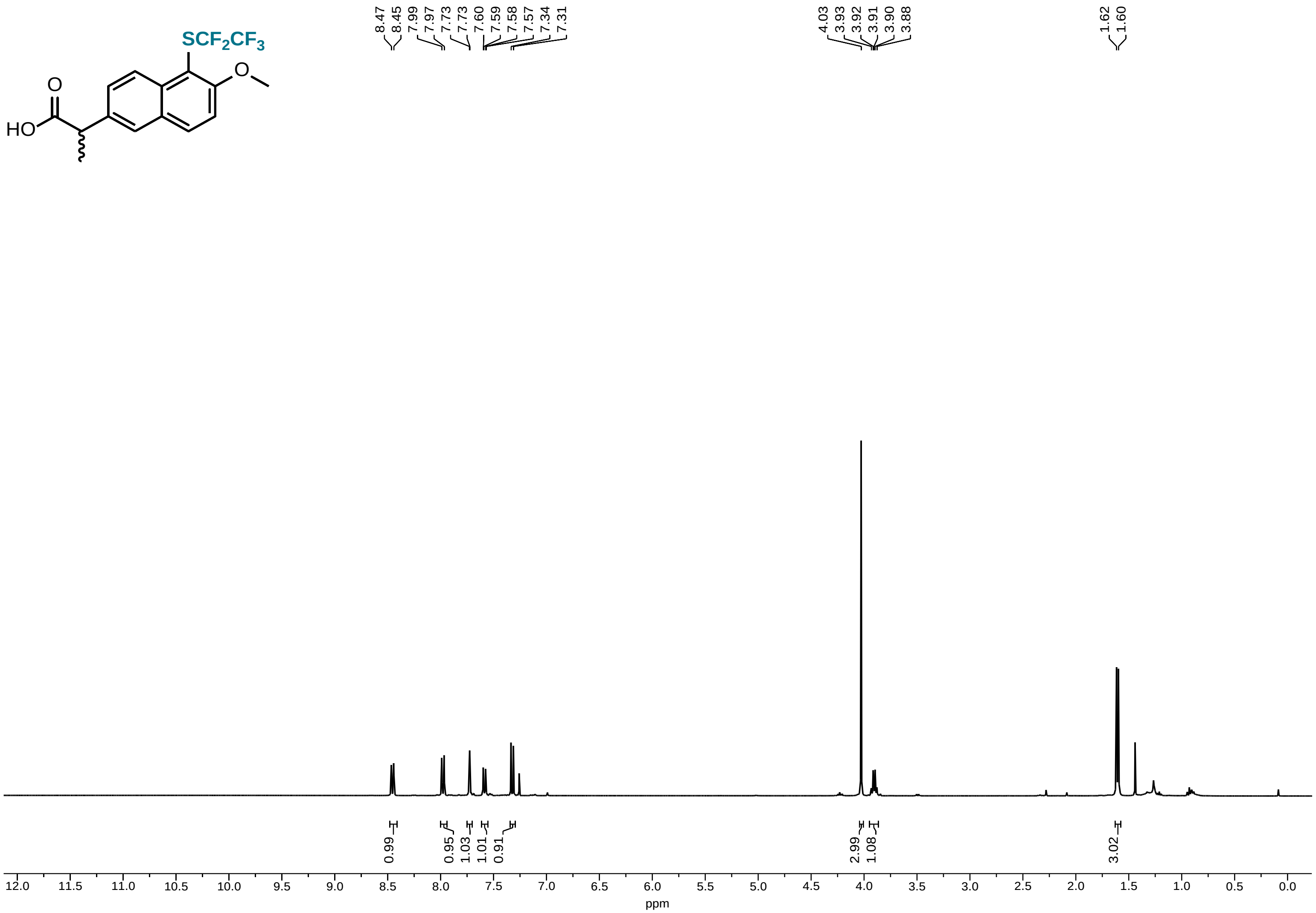

Figure S133. ^{1}H NMR ($CDCl_3$, 400 MHz) of **25b**

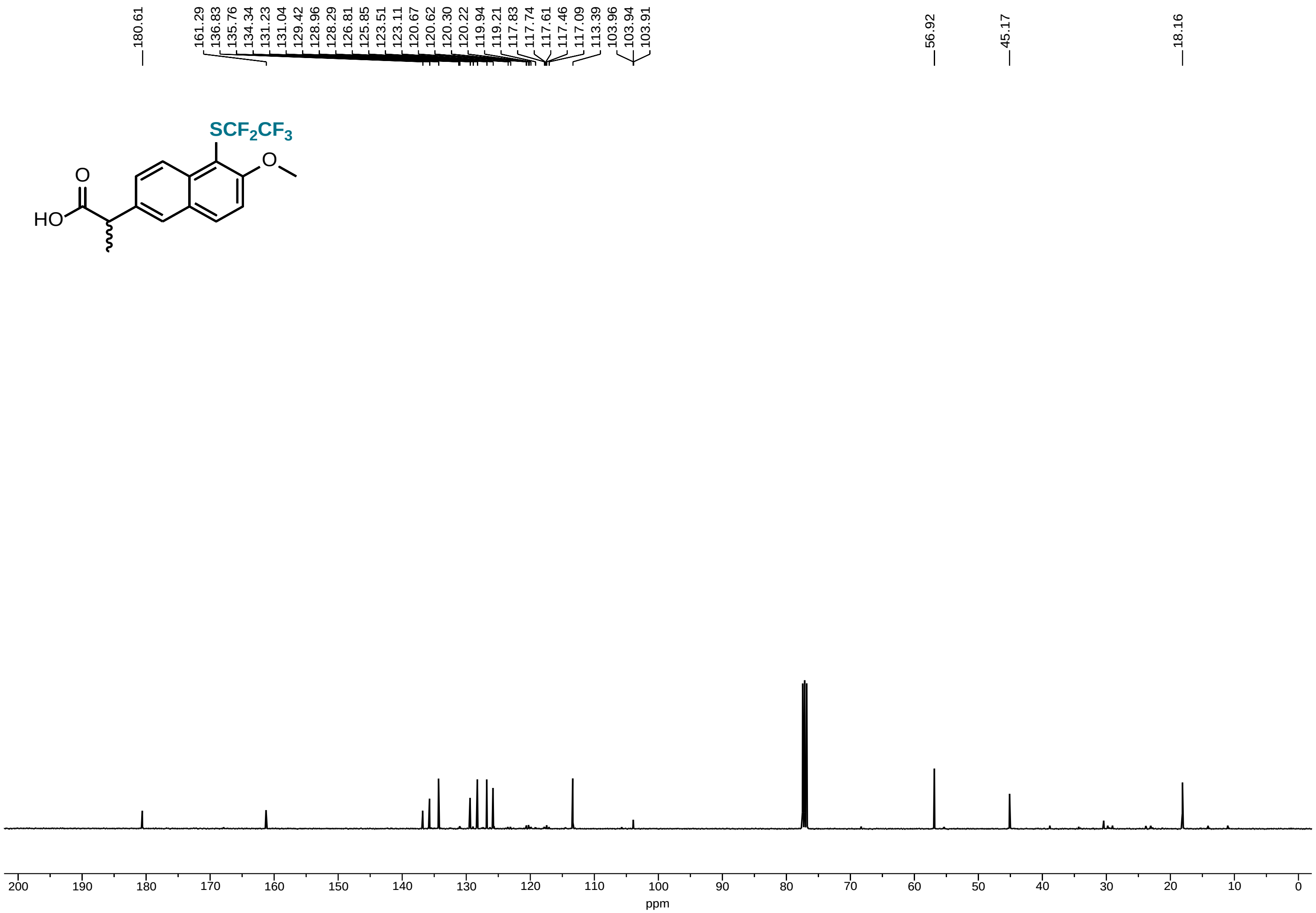

Figure S134. $^{13}C\{^{1}H\}$ NMR ($CDCl_3$, 100.6 MHz) of **25b**

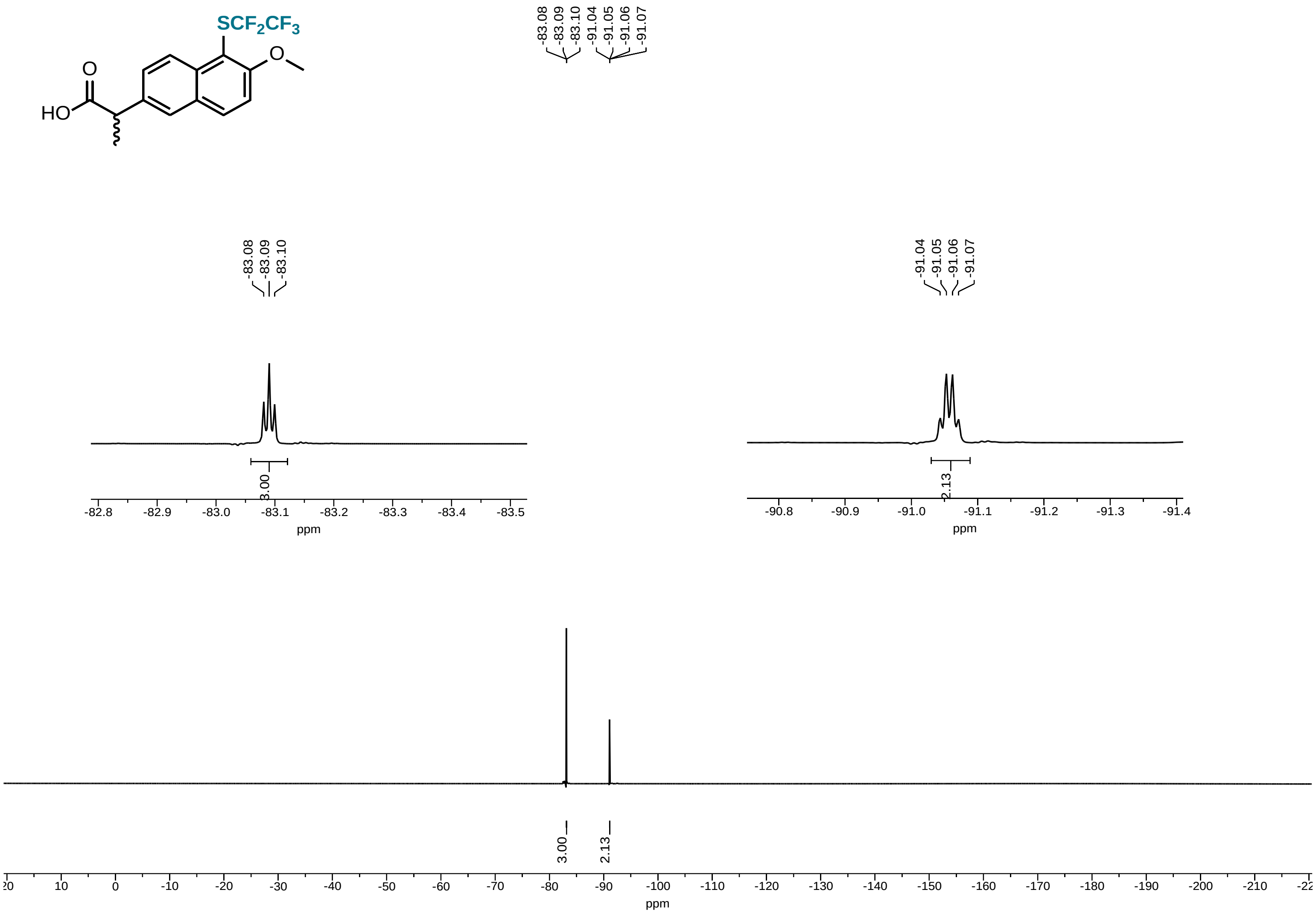

Figure S135. ^{19}F NMR ($CDCl_3$, 376.5 MHz) of **25b**

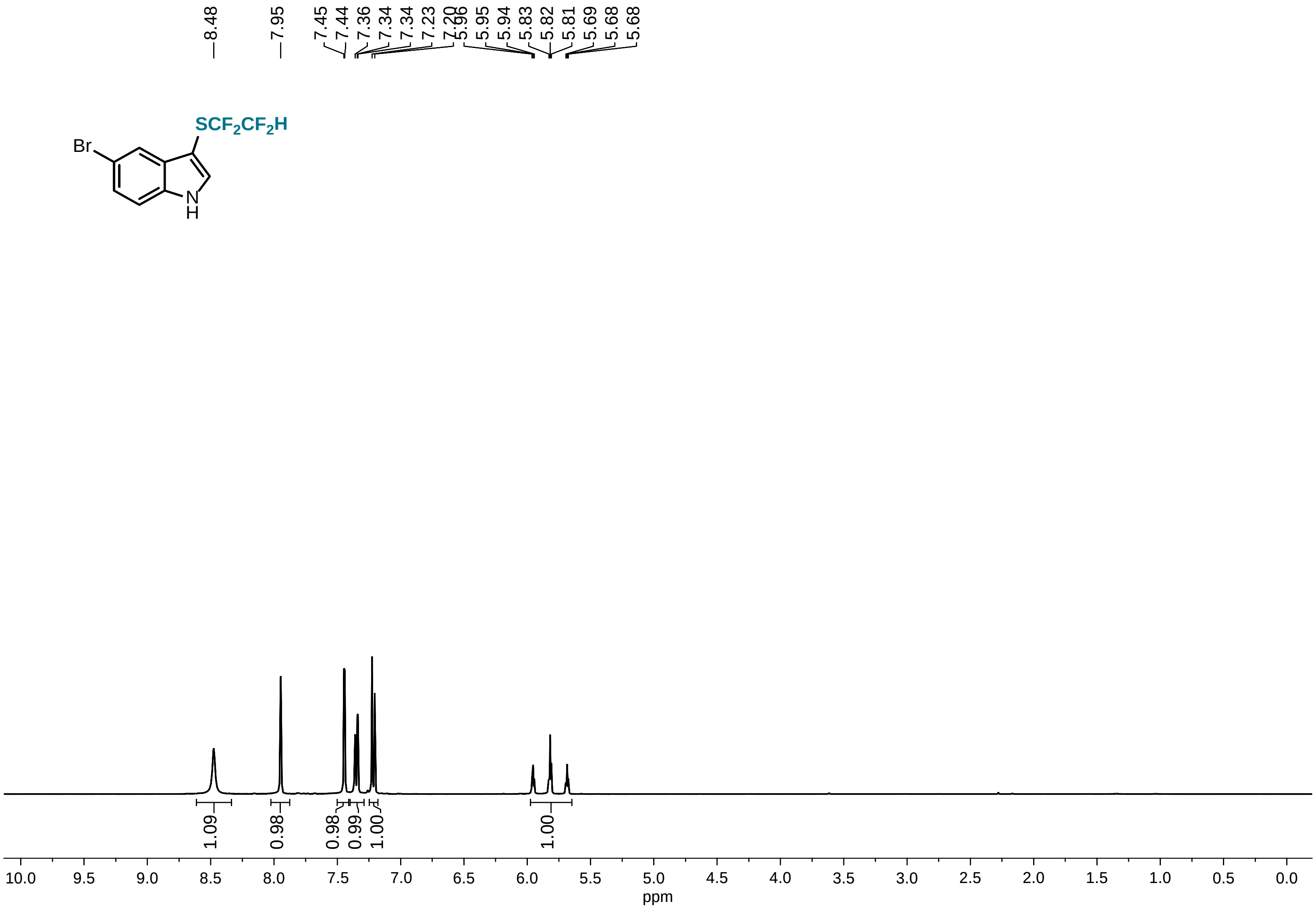

Figure S136. ^{1}H NMR ($CDCl_3$, 400 MHz) of **26a**

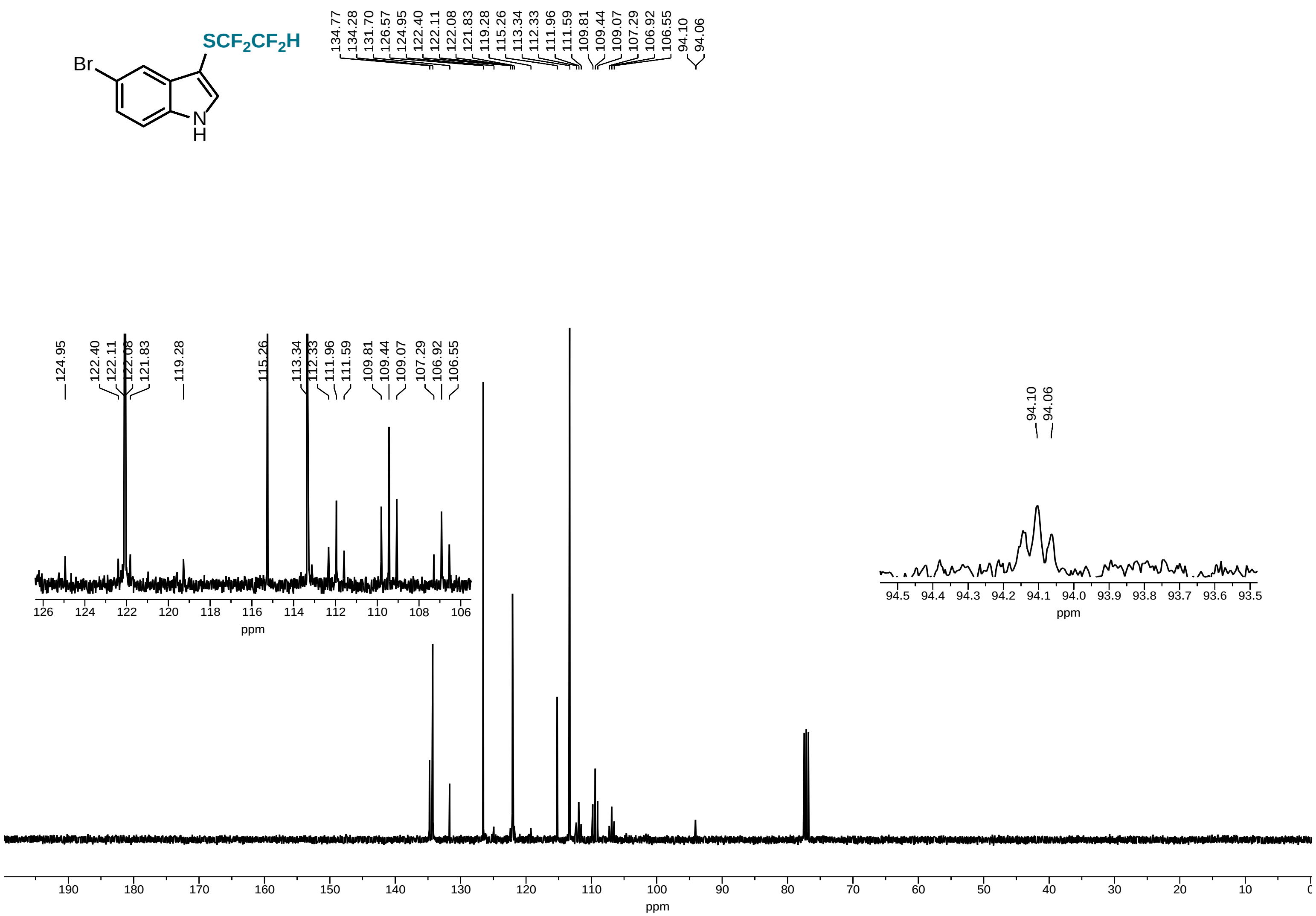

Figure S137. $^{13}C\{^{1}H\}$ NMR ($CDCl_3$, 100.6 MHz) of **26a**

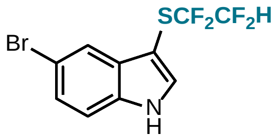

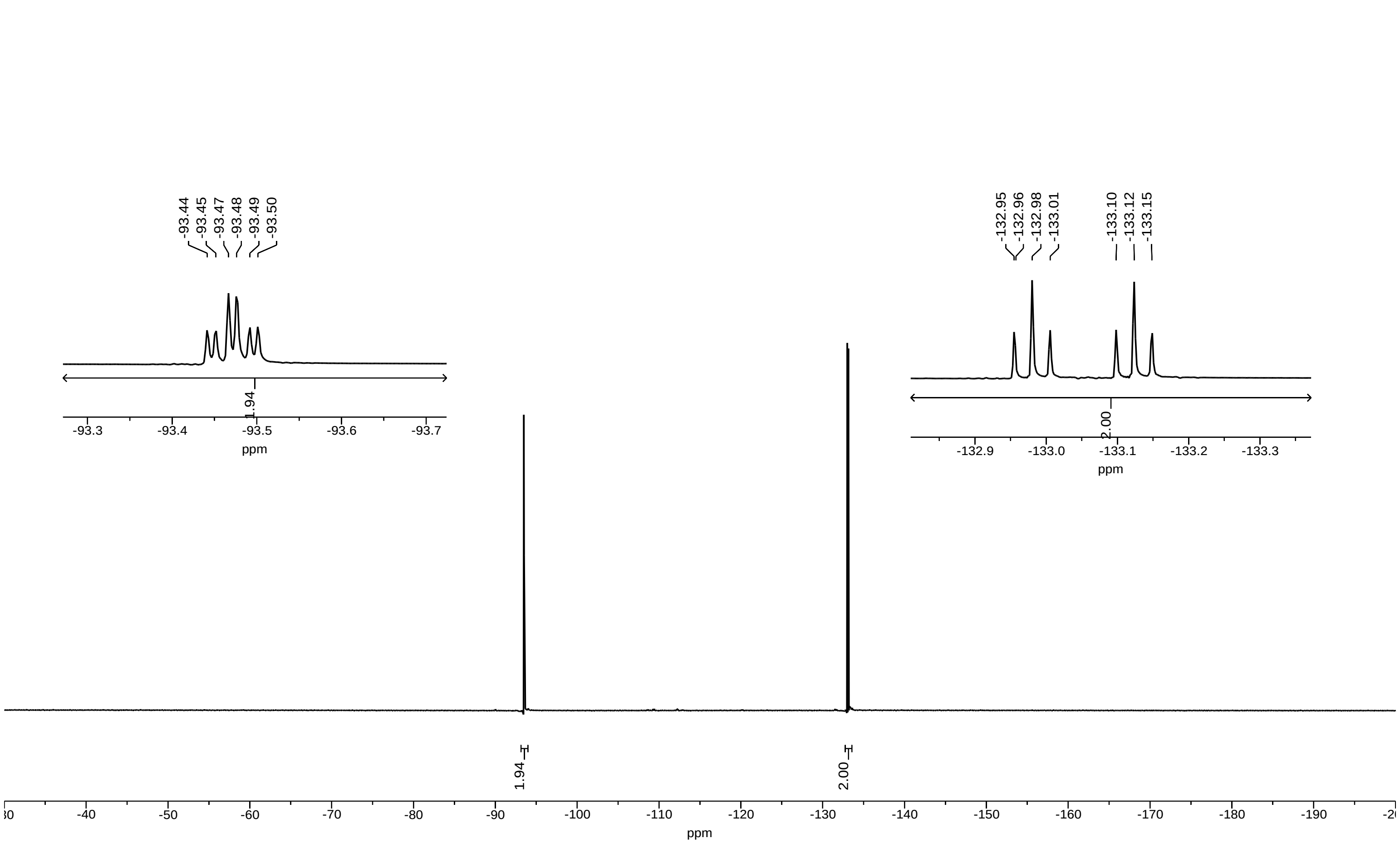

Figure S138. ^{19}F NMR ($CDCl_3$, 376.5 MHz) of **26a**

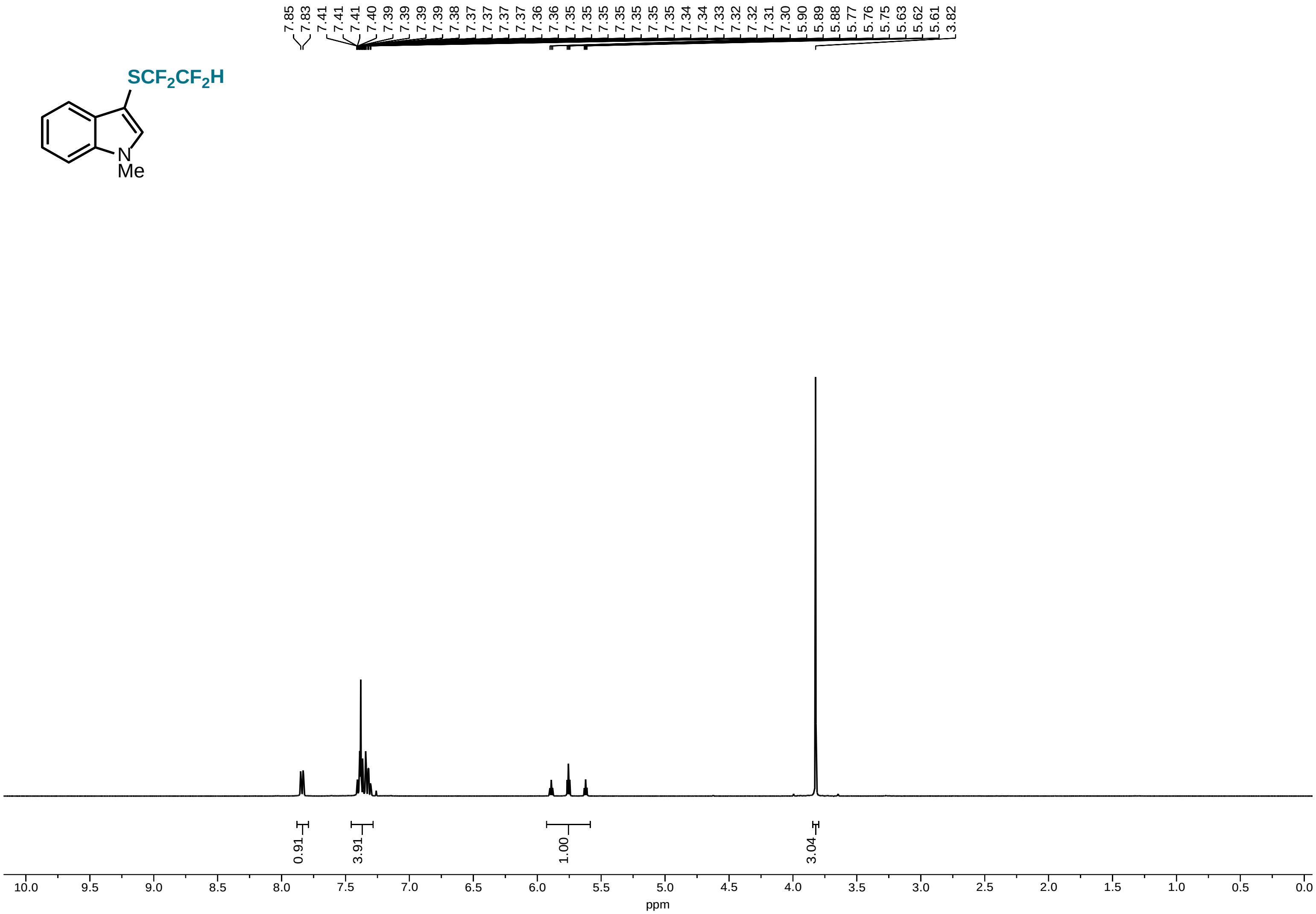

Figure S139. ^{1}H NMR ($CDCl_3$, 400 MHz) of **27a**

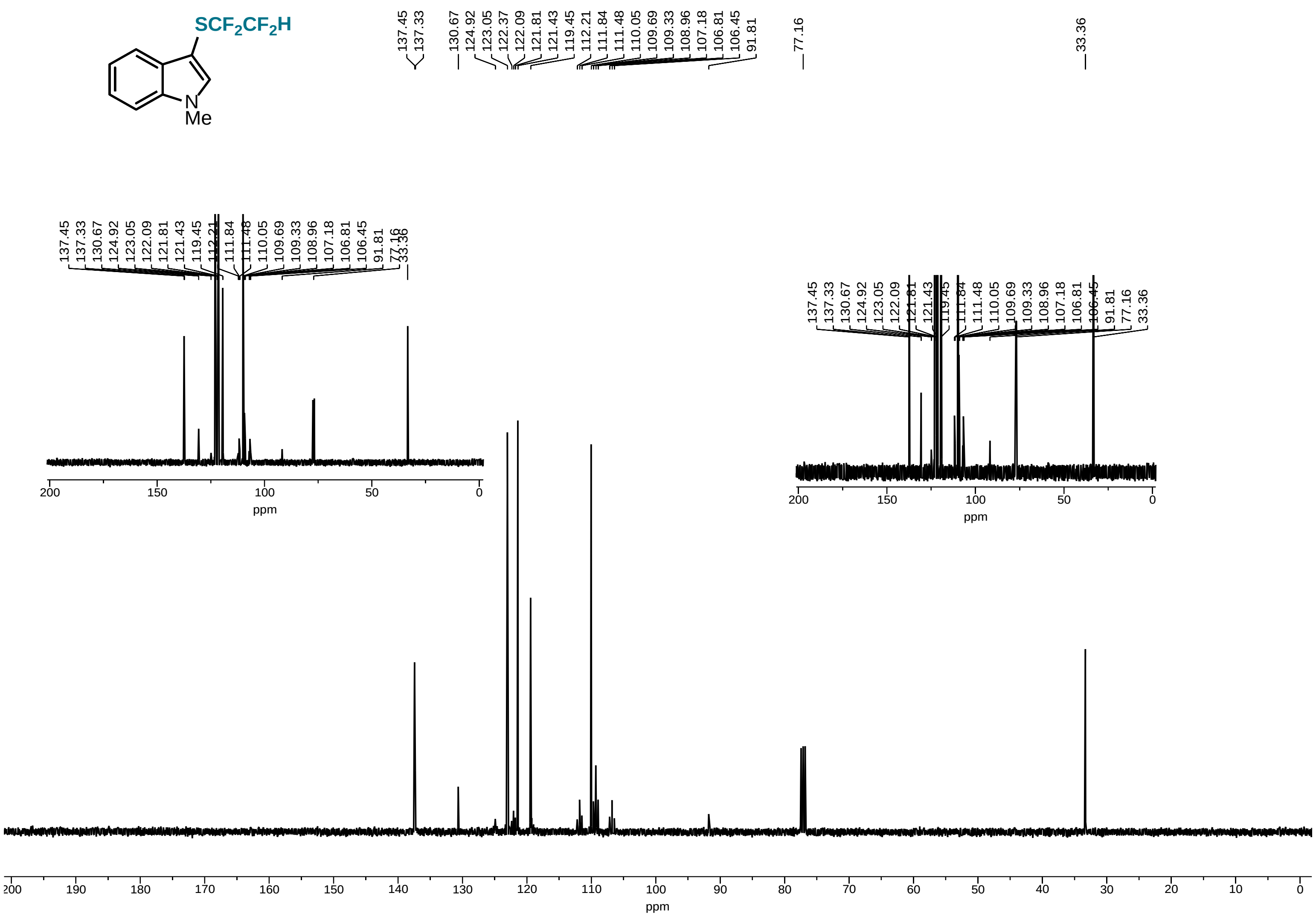

Figure S140. $^{13}C\{^{1}H\}$ NMR ($CDCl_3$, 100.6 MHz) of **27a**

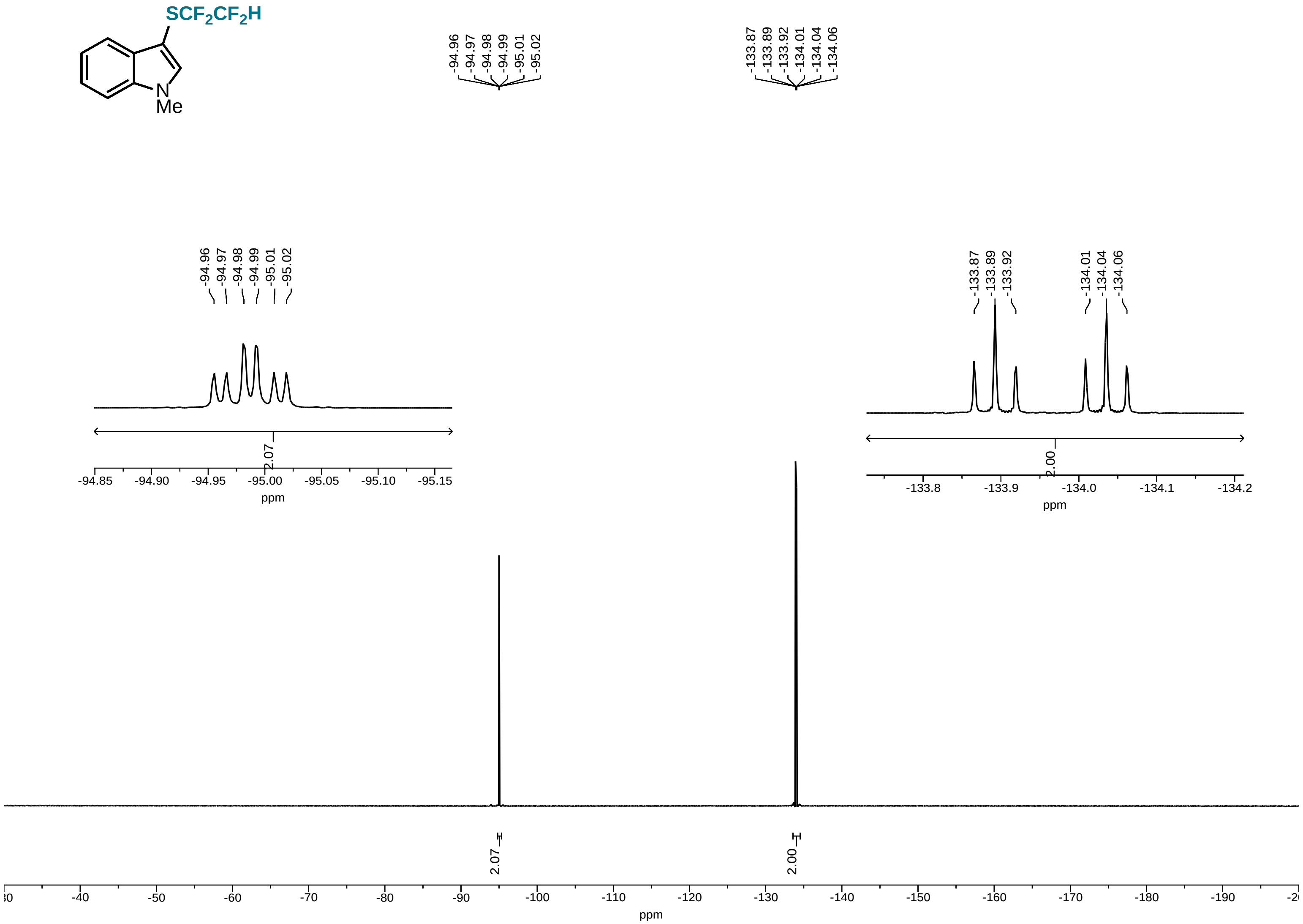

Figure S141. ^{19}F NMR ($CDCl_3$, 376.5 MHz) of **27a**

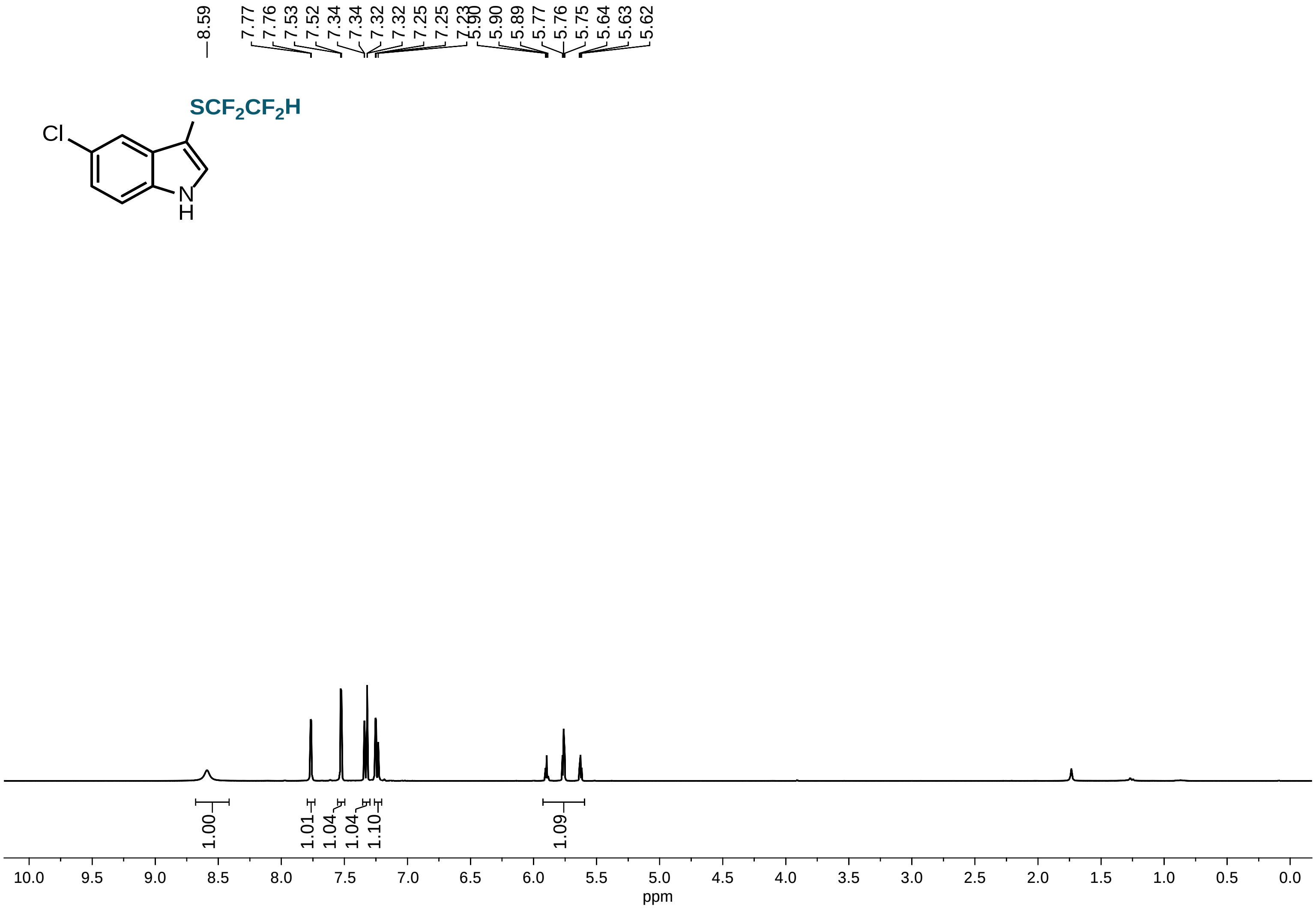

Figure S142. ^{1}H NMR ($CDCl_3$, 400 MHz) of **28a**

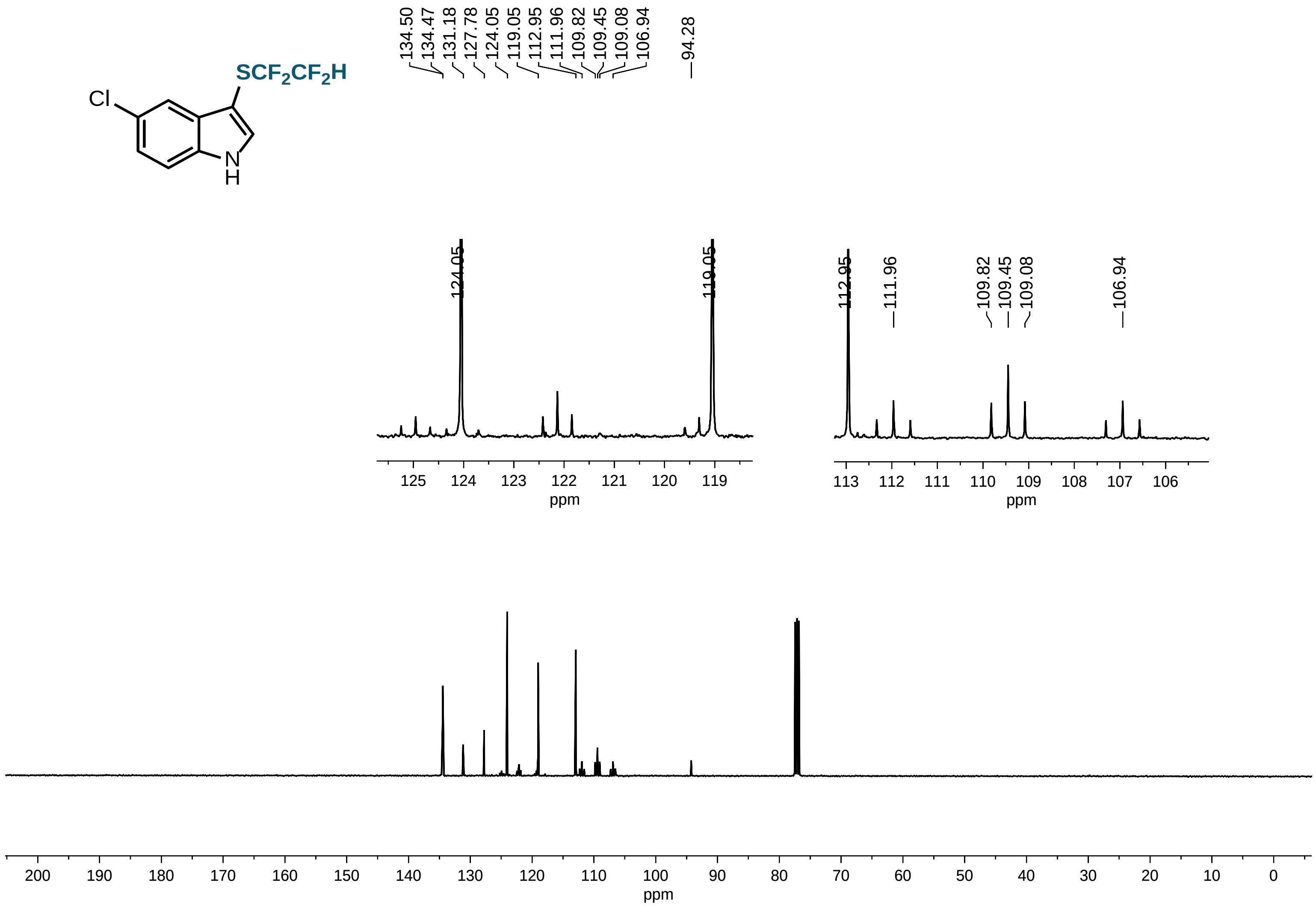

Figure S143. $^{13}C\{^1H\}$ NMR ($CDCl_3$, 100.6 MHz) of **28a**

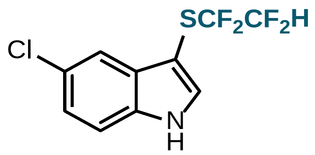

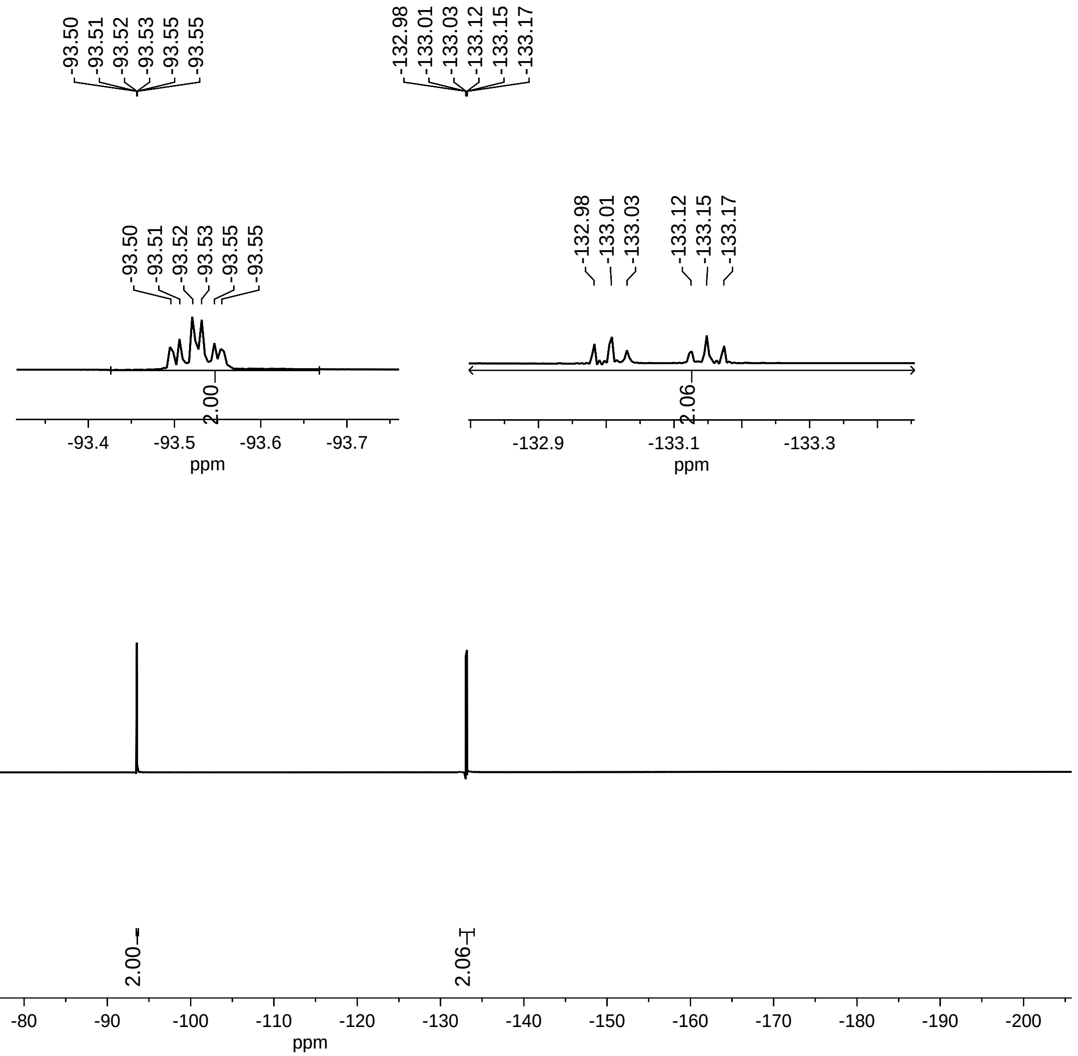

Figure S144. ^{19}F NMR ($CDCl_3$, 376.5 MHz) of **28a**

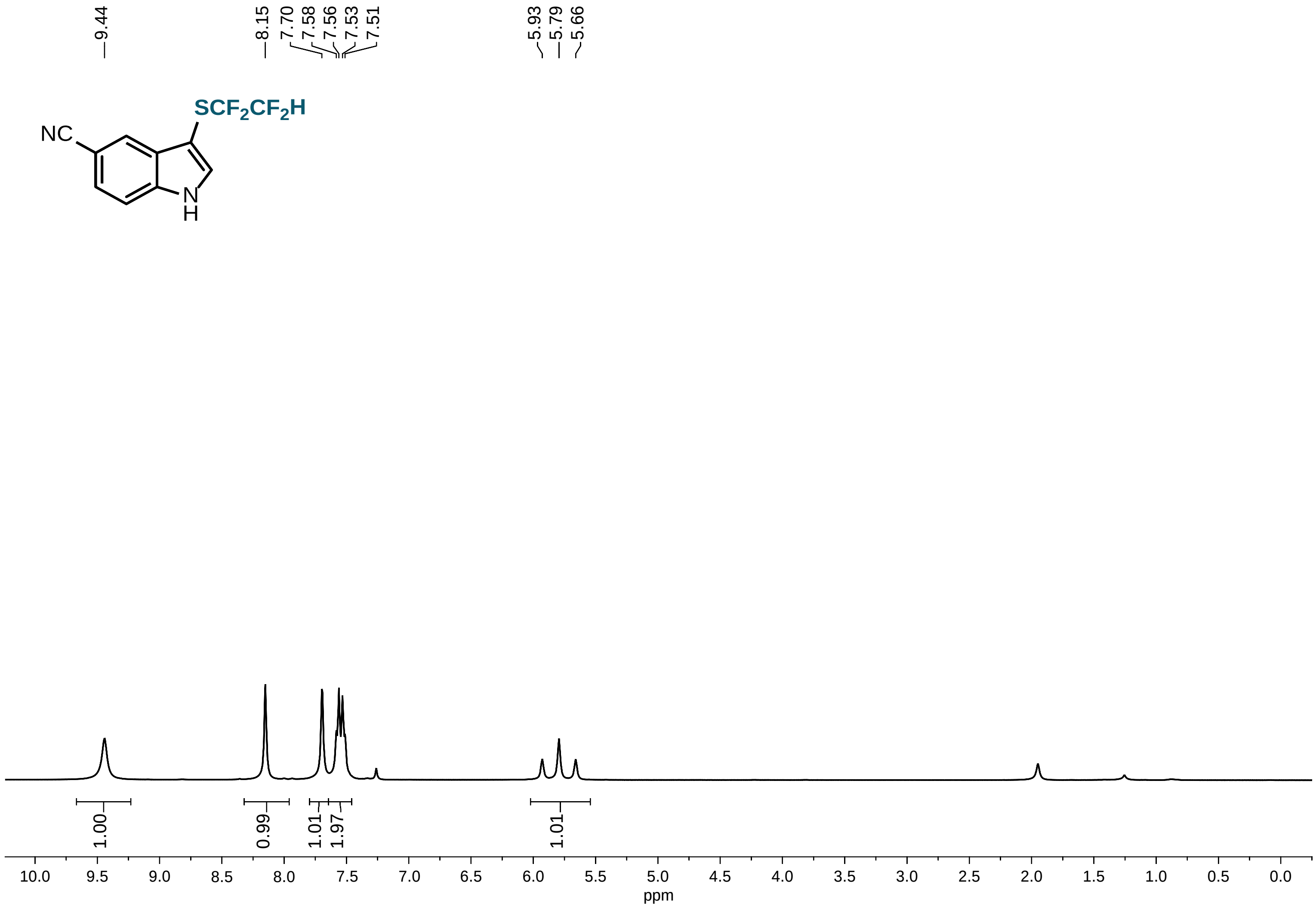

Figure S145. ^{1}H NMR ($CDCl_3$, 400 MHz) of **29a**

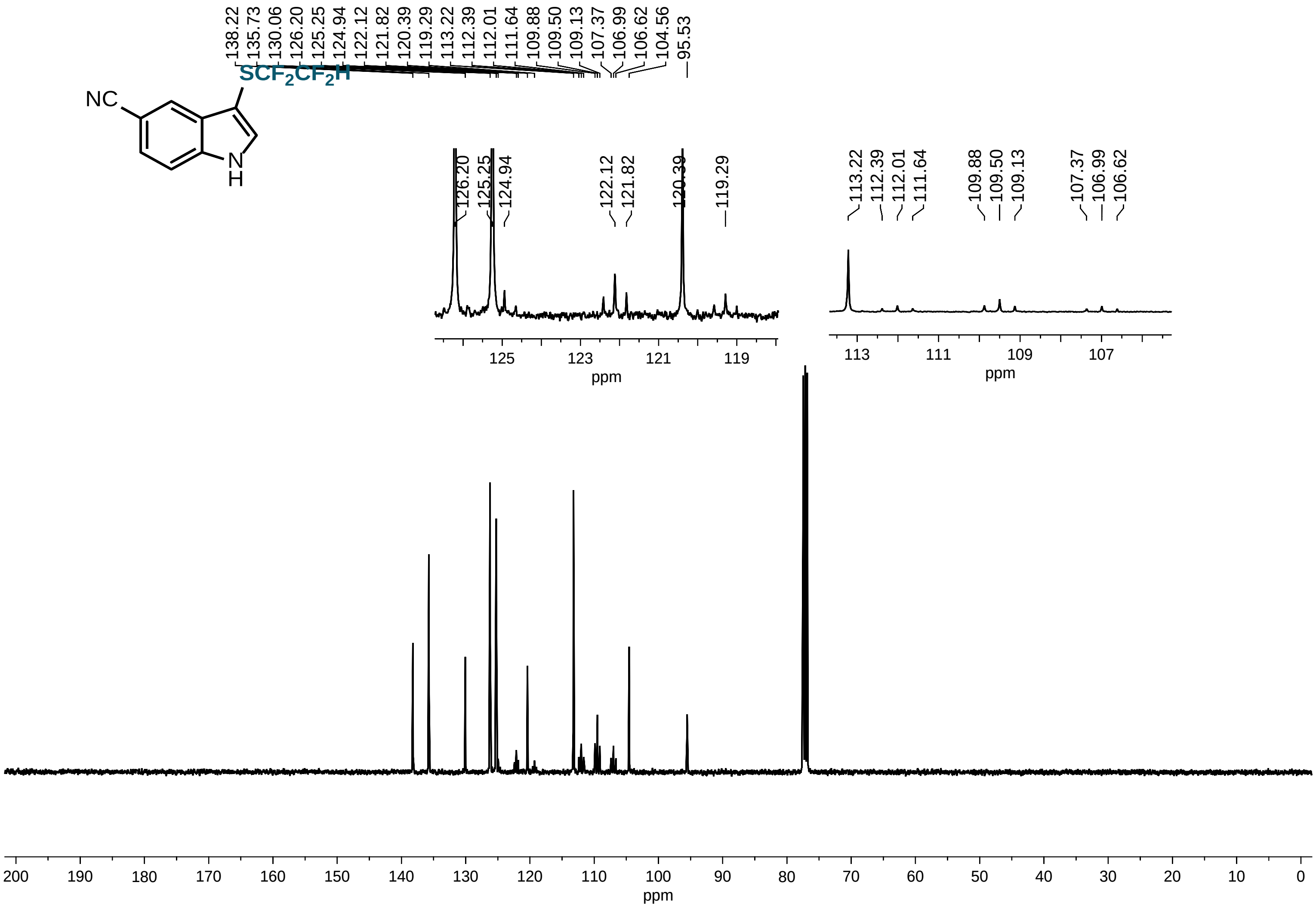

Figure S146. $^{13}C\{^1H\}$ NMR ($CDCl_3$, 100.6 MHz) of **29a**

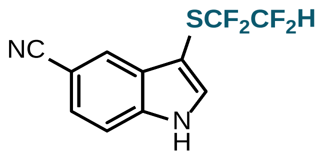

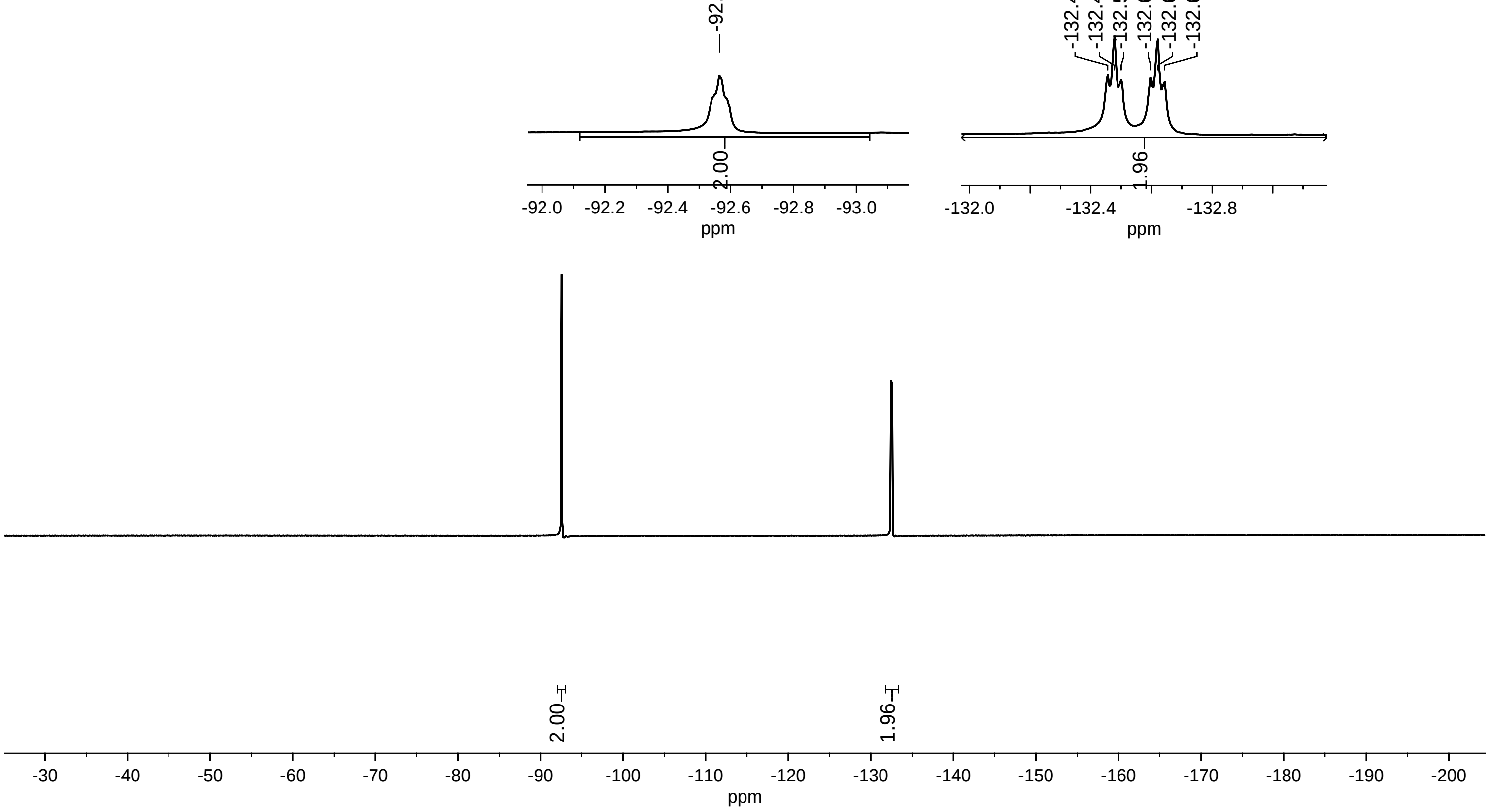

Figure S147. ^{19}F NMR ($CDCl_3$, 376.5 MHz) of **29a**

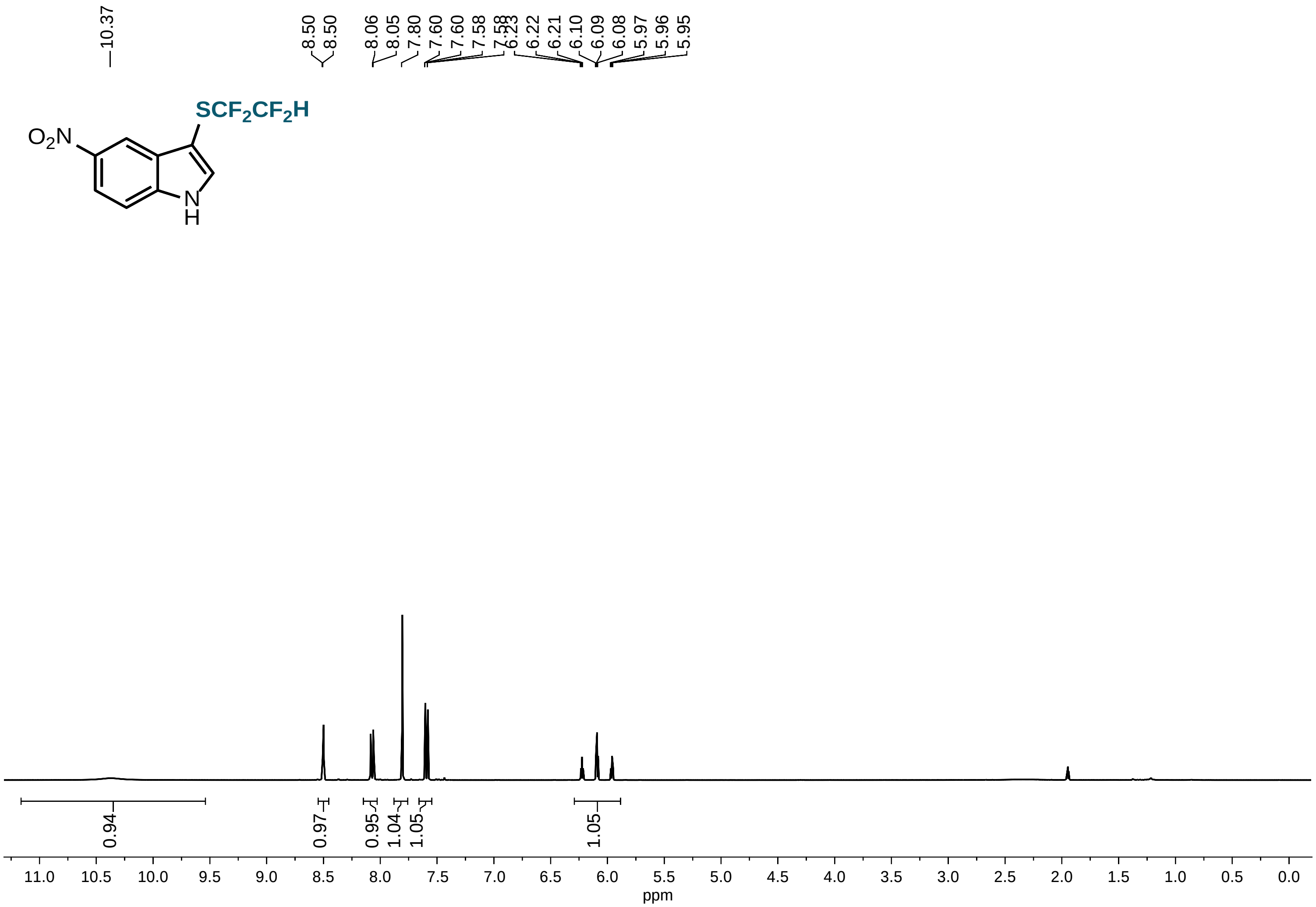

Figure S148. ^{1}H NMR (CD_3CN, 400 MHz) of **30a**

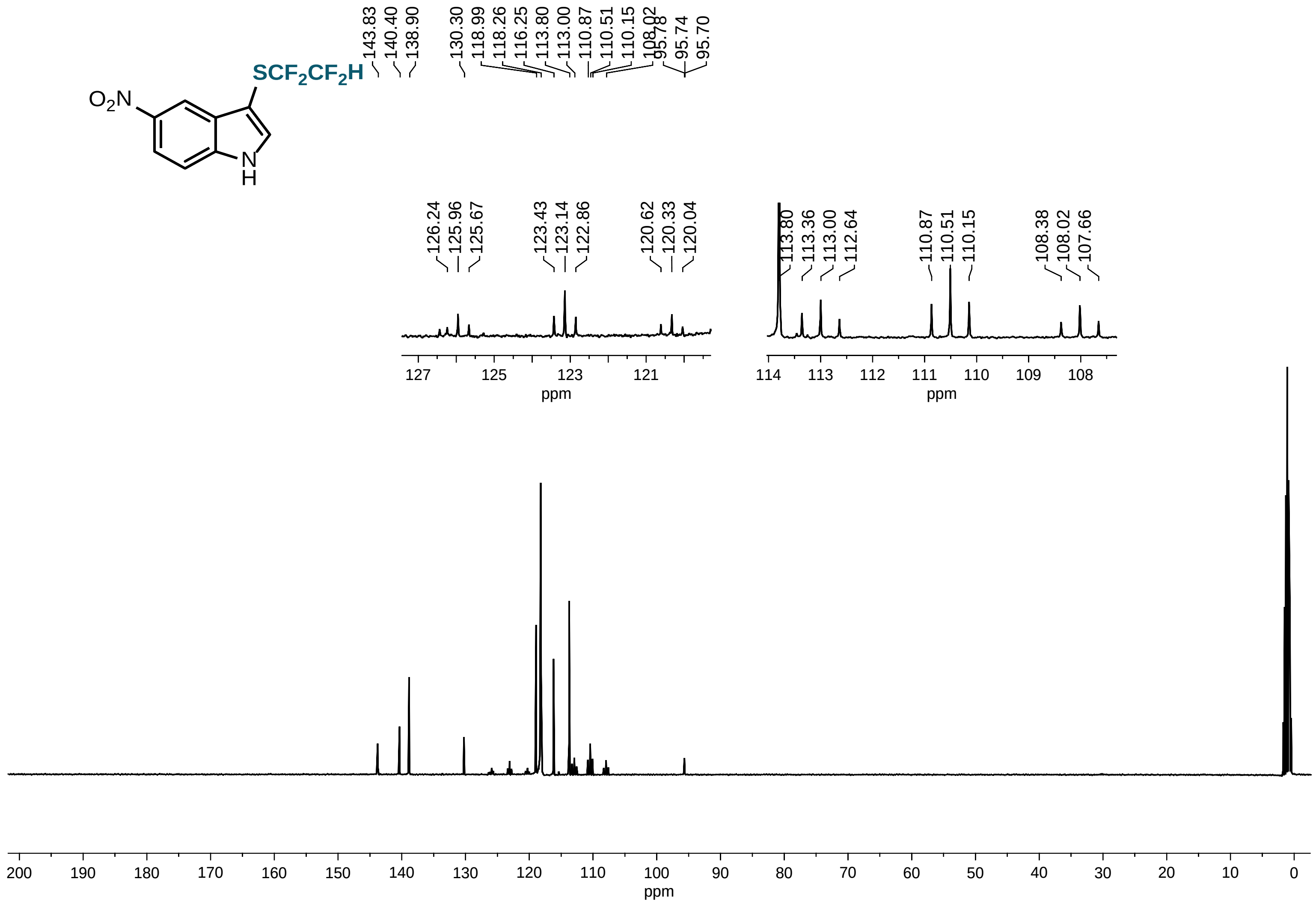

Figure S149. $^{13}C\{^{1}H\}$ NMR (CD_3CN, 100.6 MHz) of **30a**

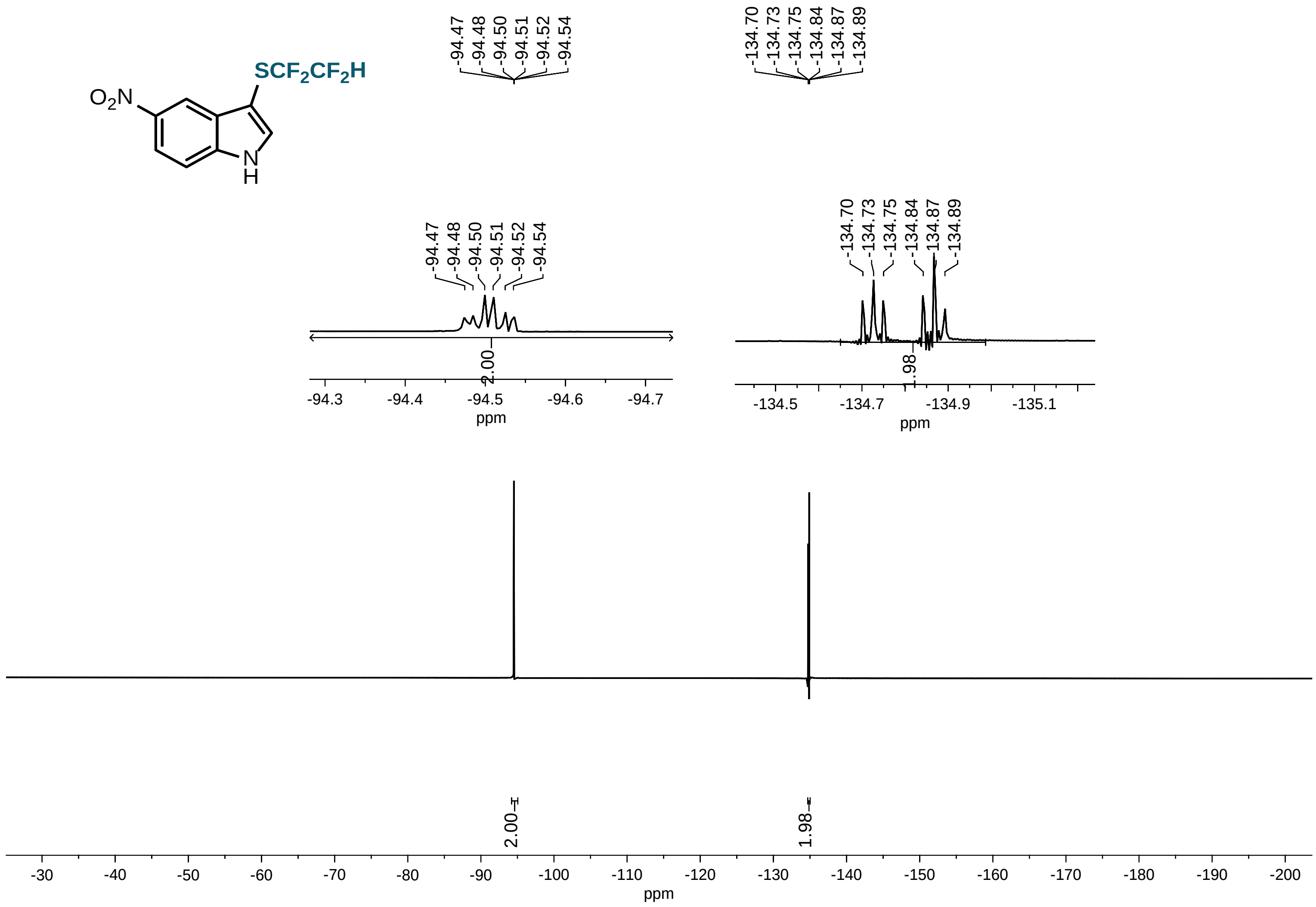

Figure S150. ^{19}F NMR (CD_3CN, 376.5 MHz) of **30a**

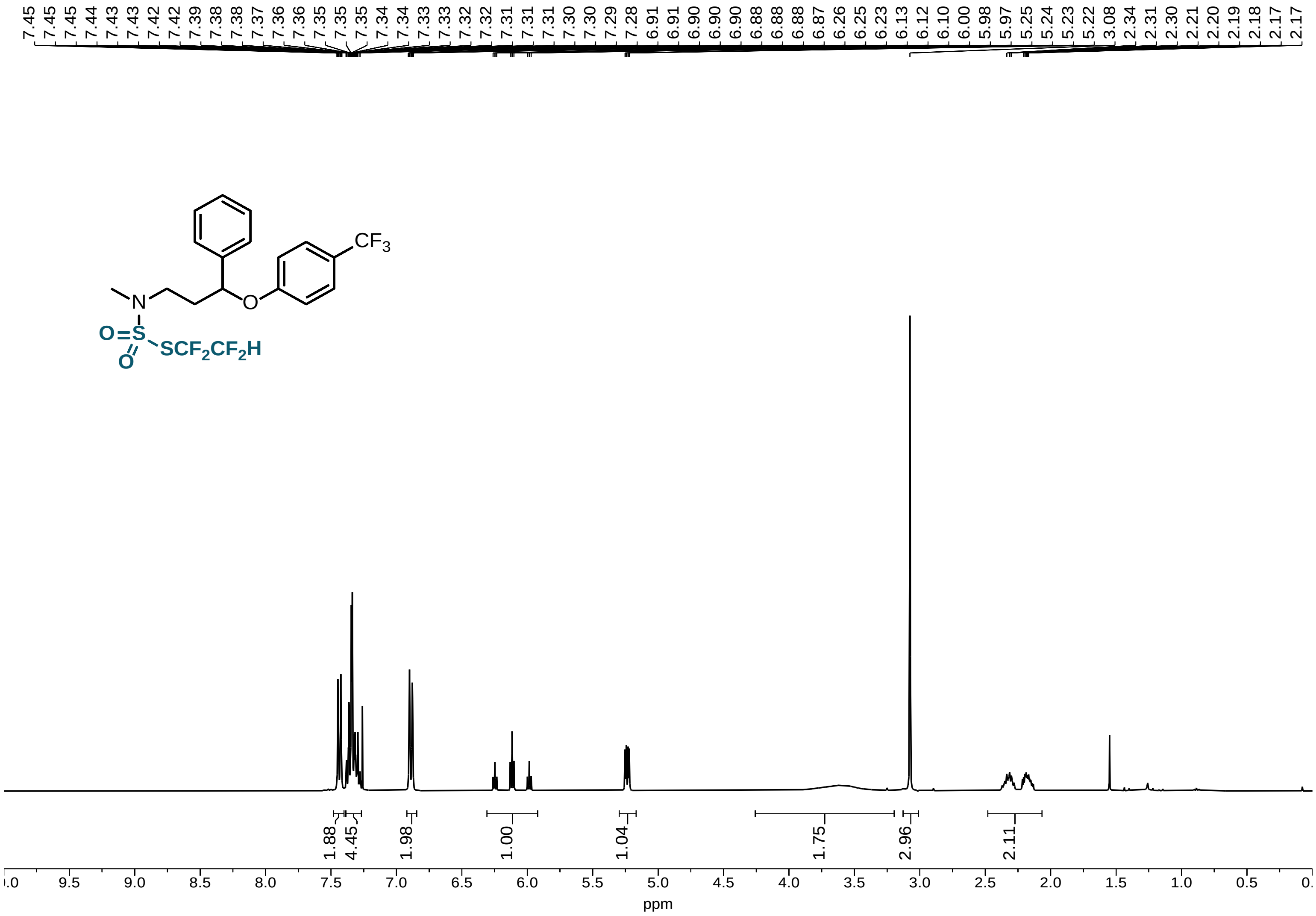

Figure S151. ^{1}H NMR ($CDCl_3$, 400 MHz) of **31a**

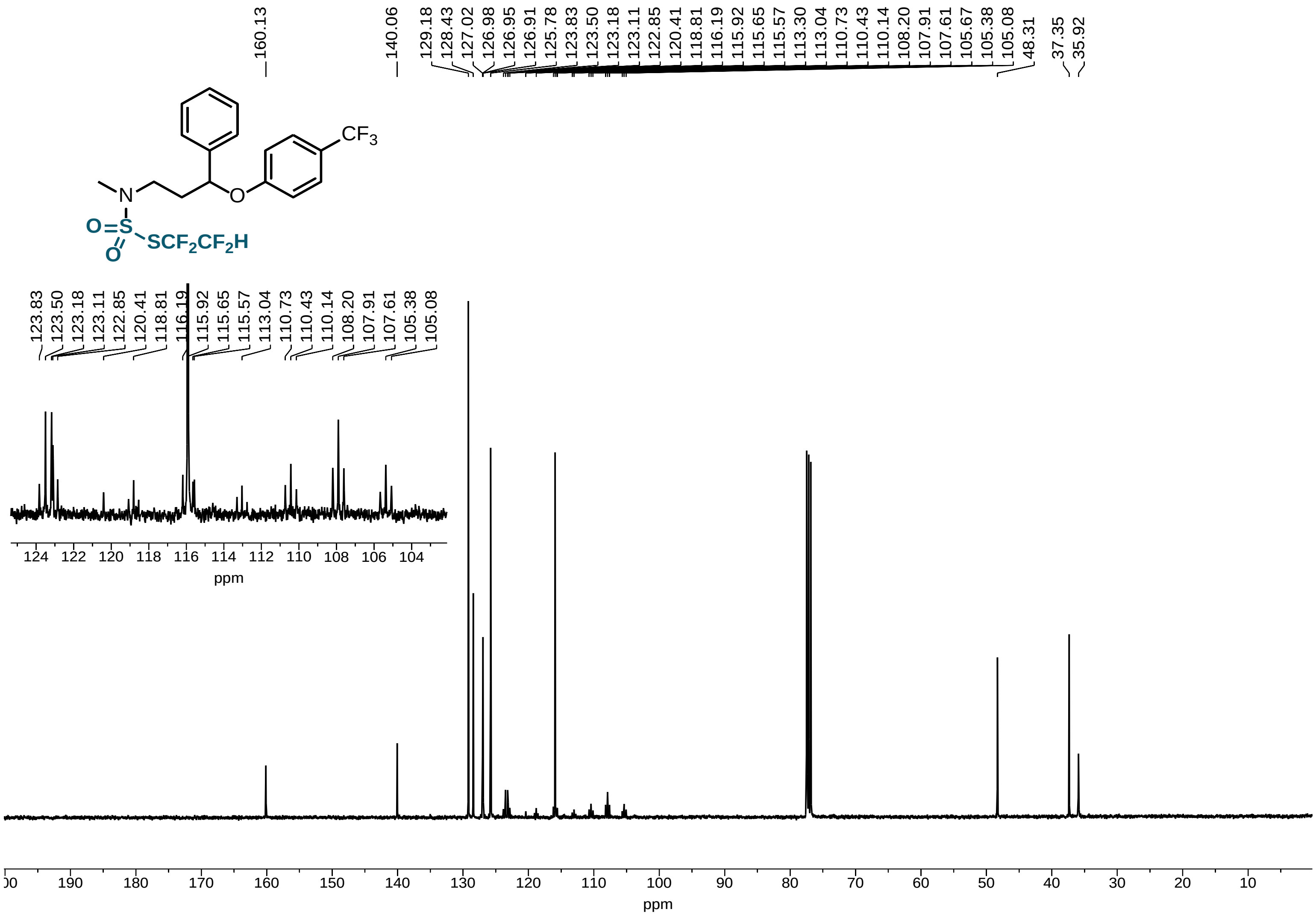

Figure S152. $^{13}C\{^1H\}$ NMR ($CDCl_3$, 100.6 MHz) of **31a**

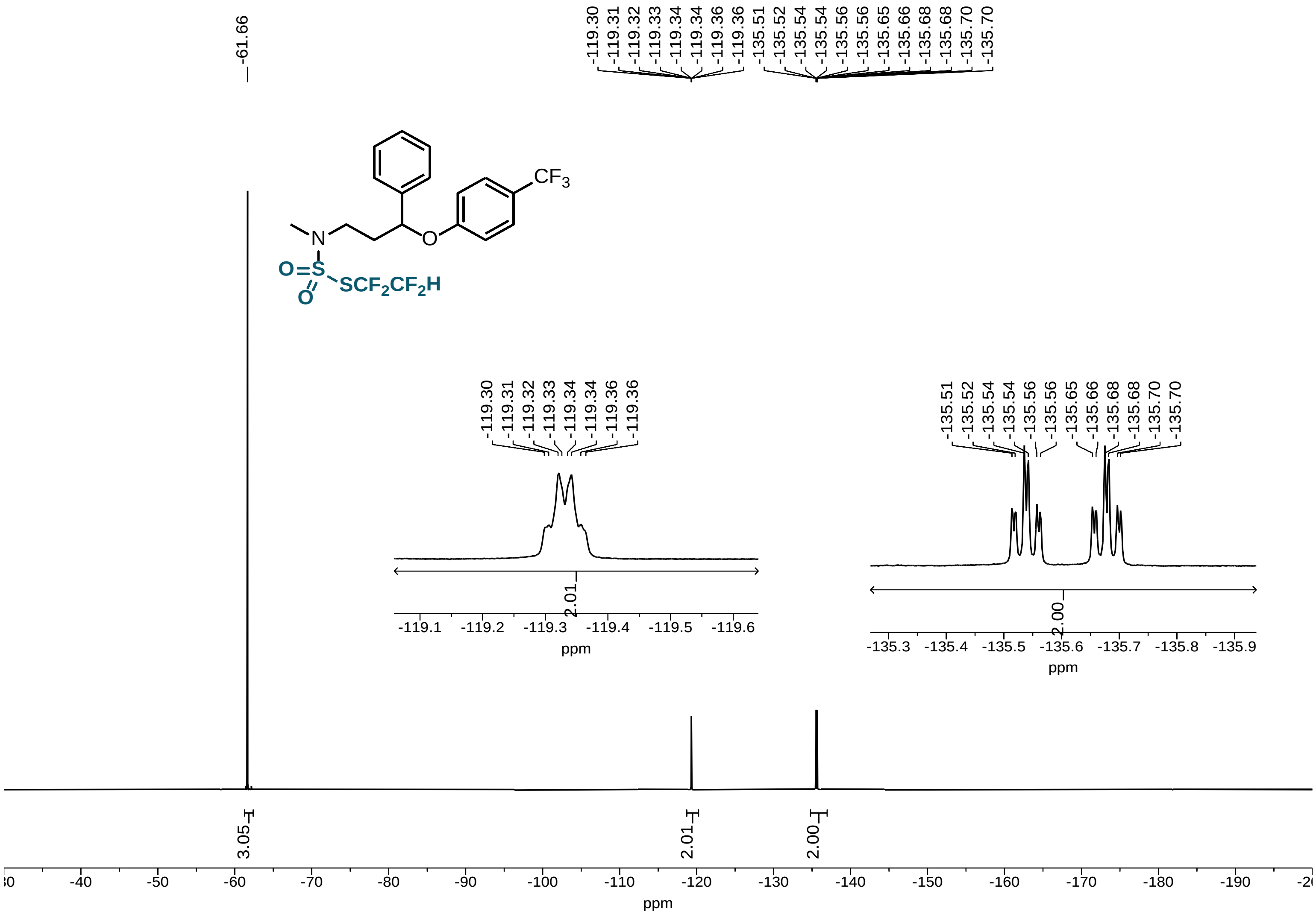

Figure S153. ^{19}F NMR ($CDCl_3$, 376.5 MHz) of **31a**

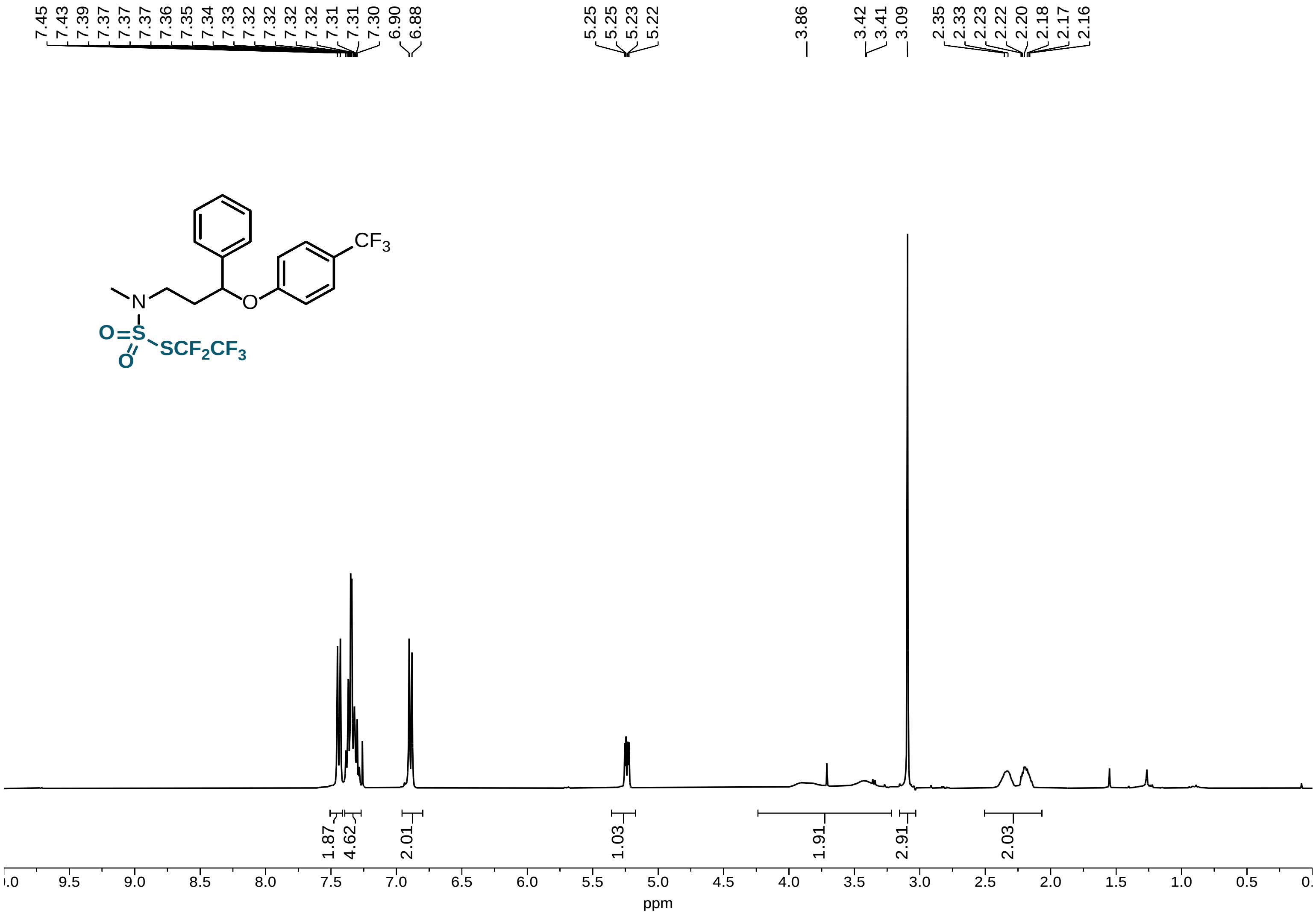

Figure S154. ^{1}H NMR ($CDCl_3$, 400 MHz) of **31b**

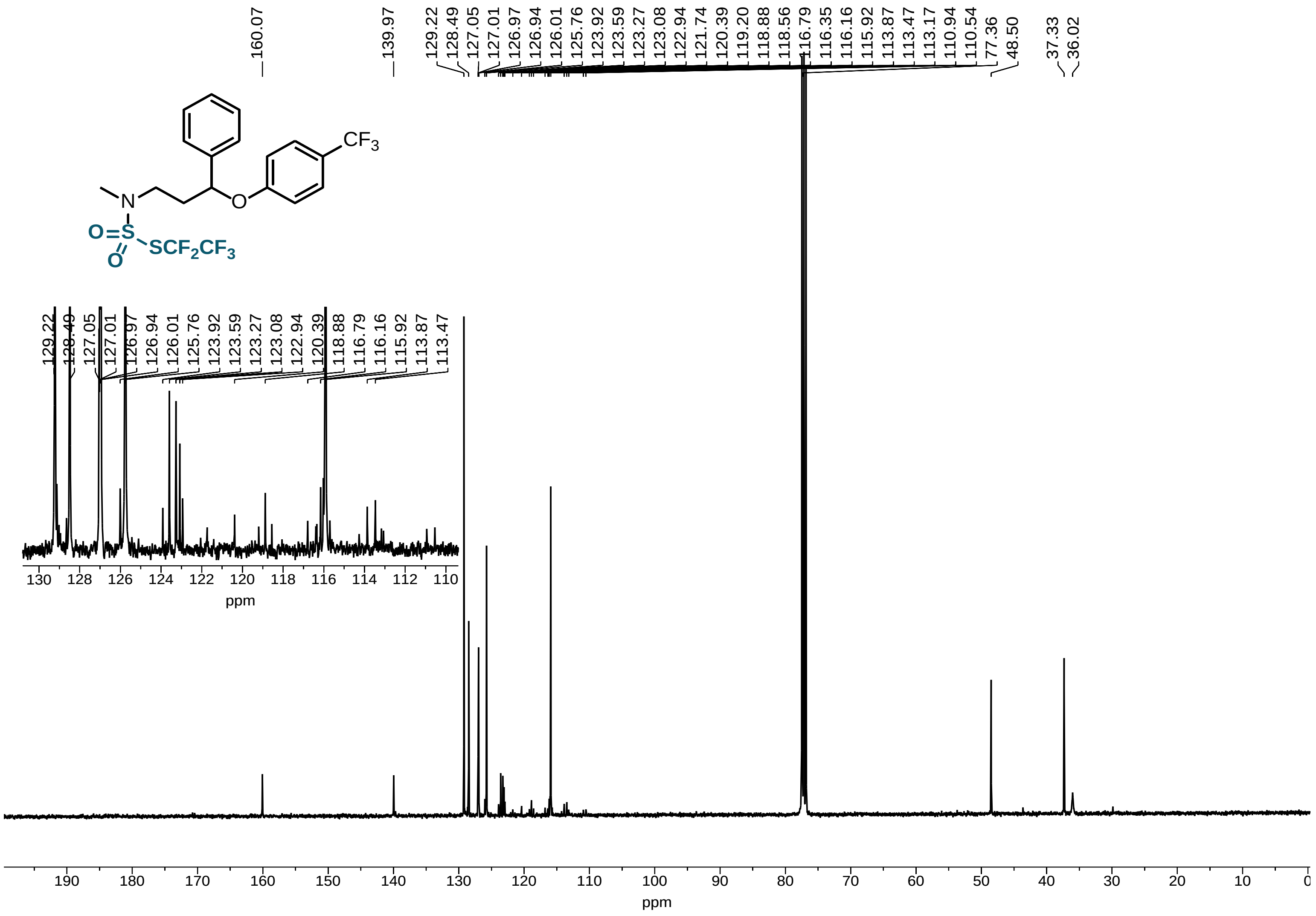

Figure S155. $^{13}C\{^1H\}$ NMR ($CDCl_3$, 100.6 MHz) of **31b**

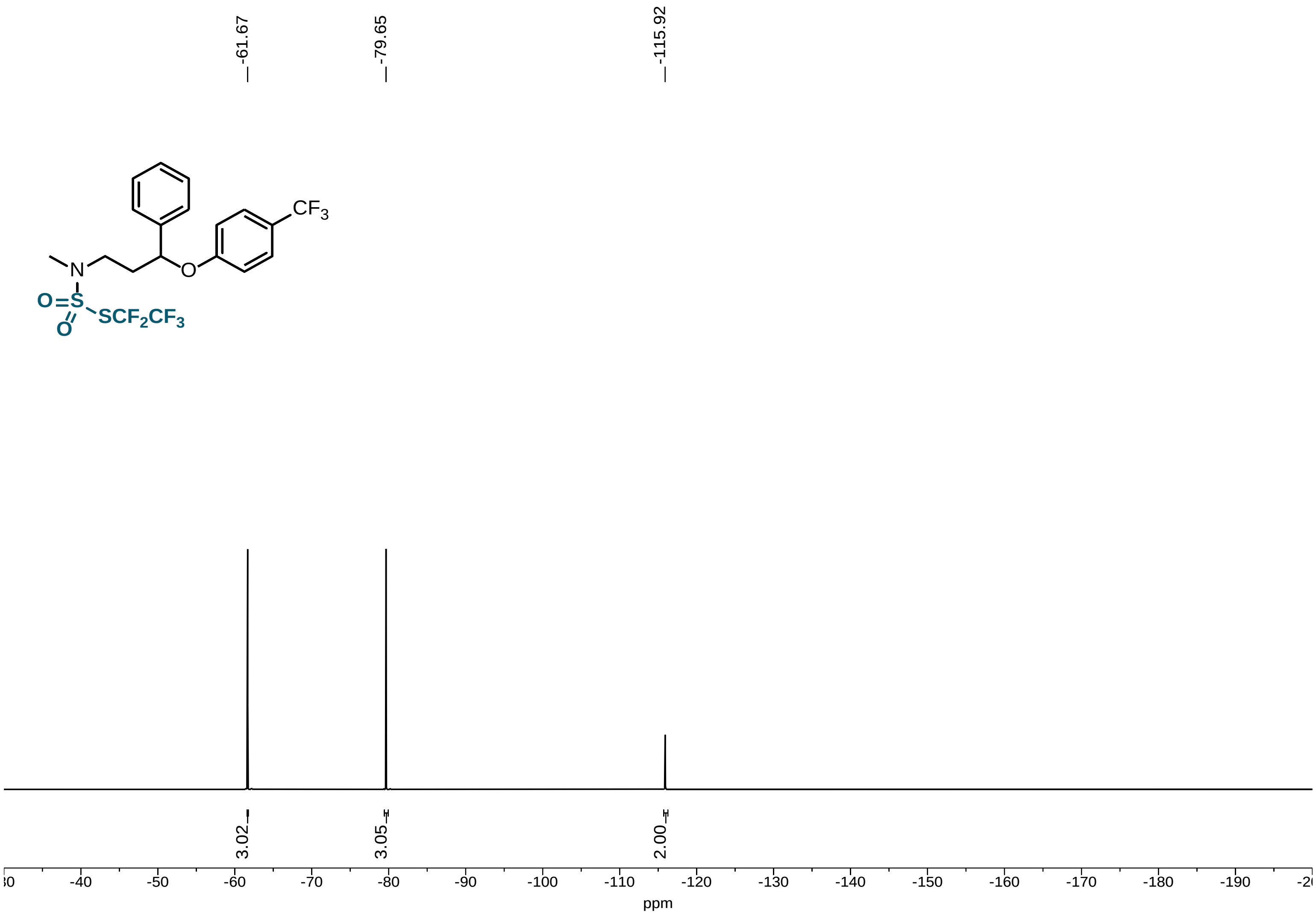

Figure S156. ^{19}F NMR ($CDCl_3$, 376.5 MHz) of **31b**

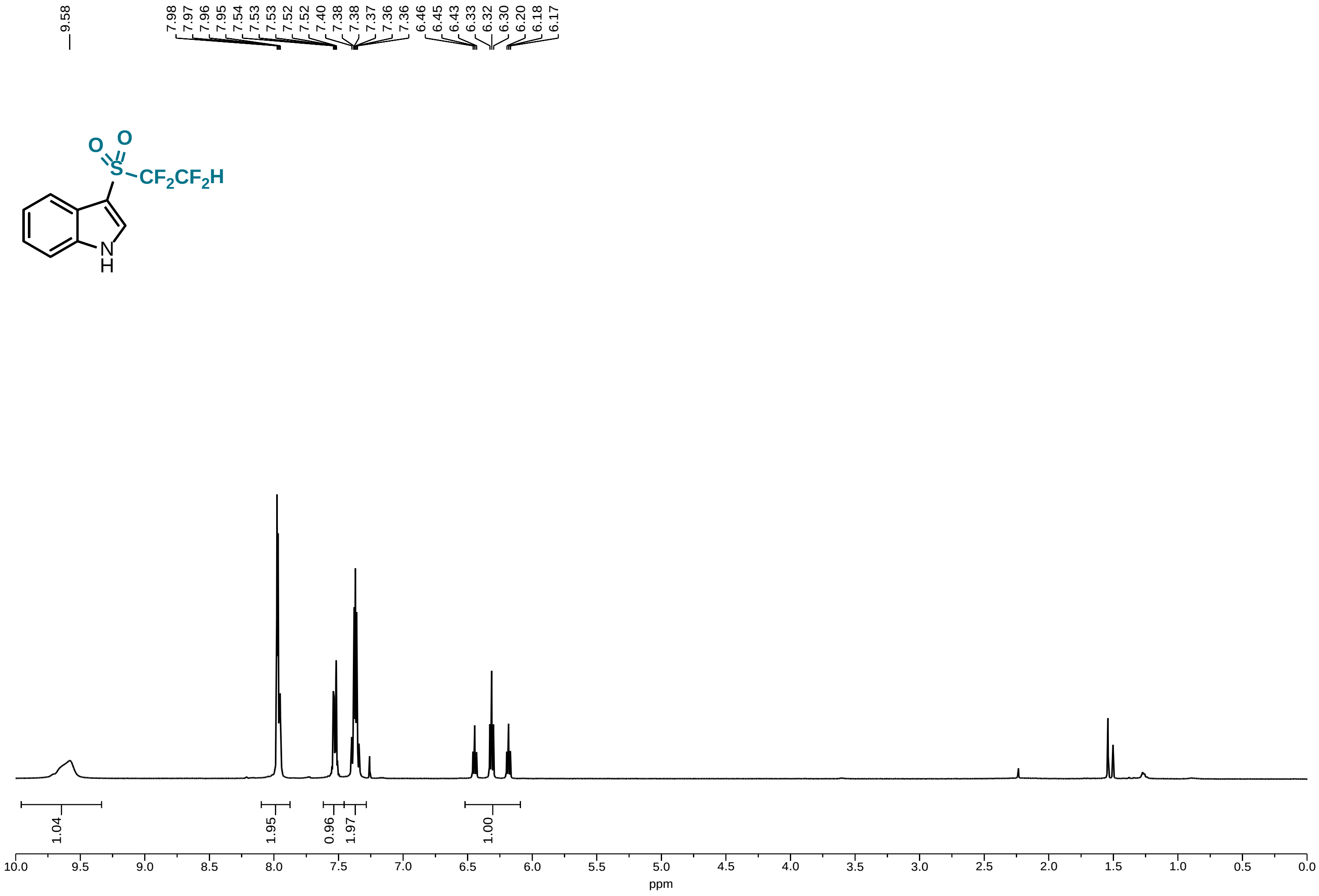

Figure S157. ^{1}H NMR ($CDCl_3$, 400 MHz) of **32a**

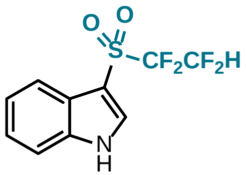

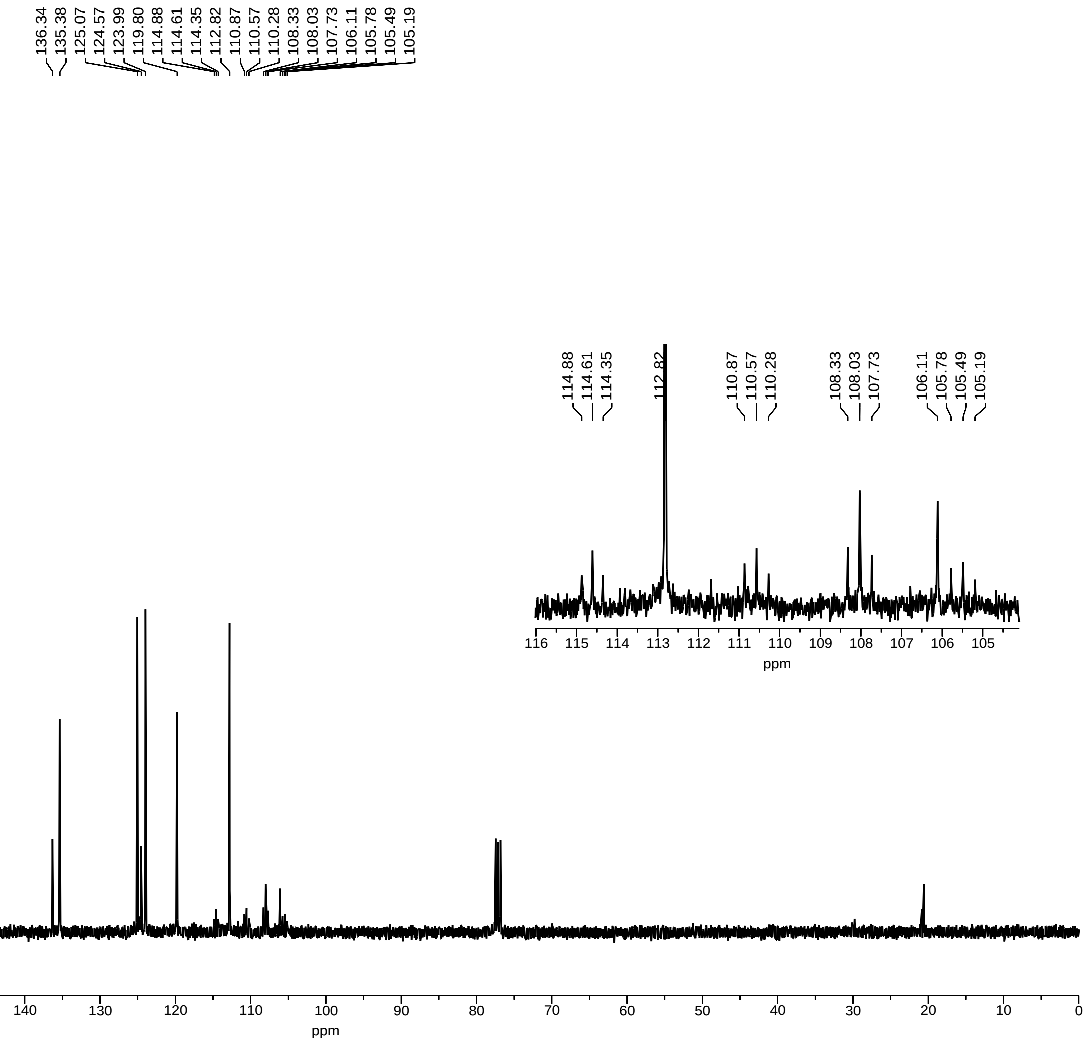

Figure S158. $^{13}C\{^{1}H\}$ NMR ($CDCl_3$, 100.6 MHz) of **32a**

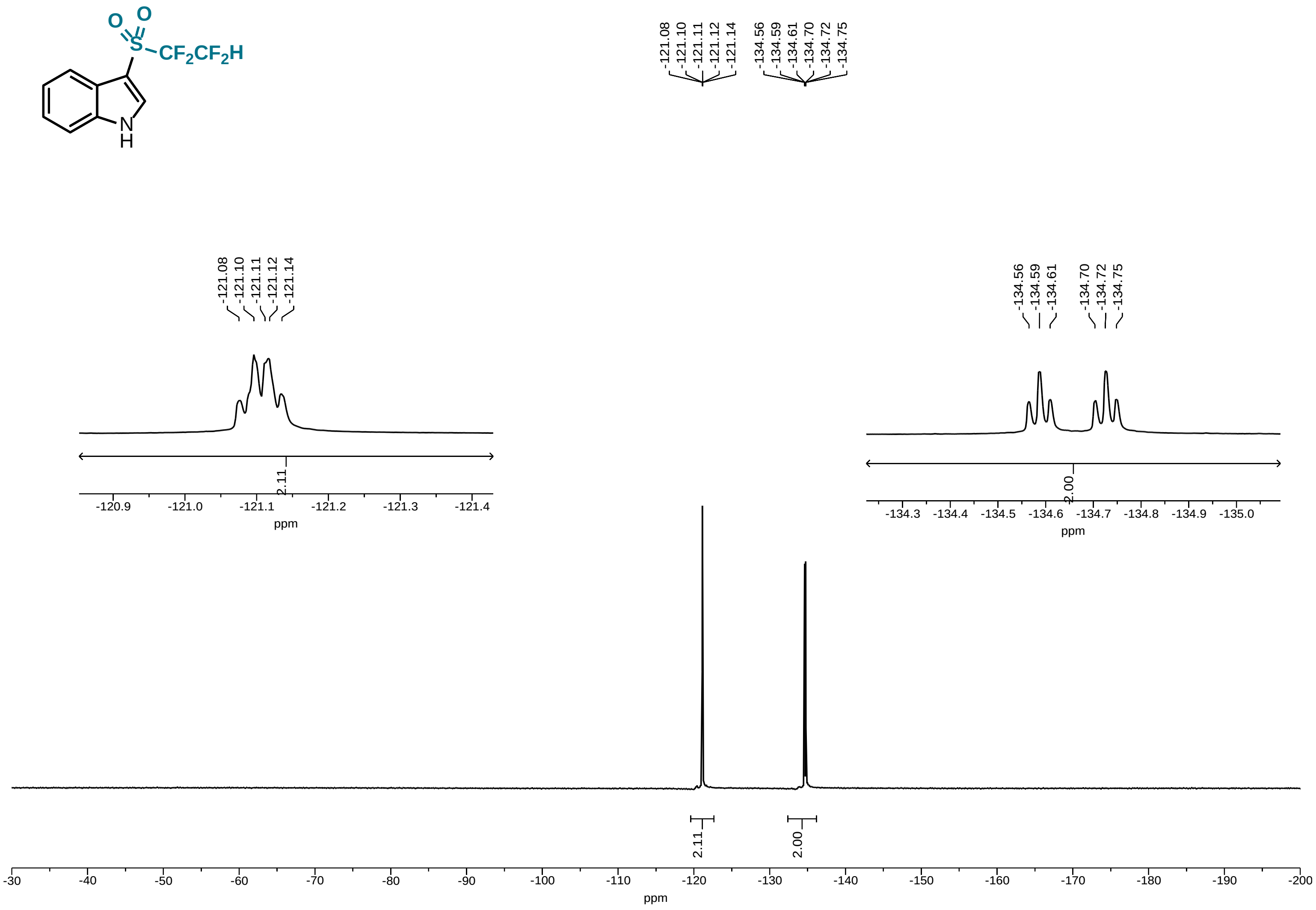

Figure S159. ^{19}F NMR ($CDCl_3$, 376.5 MHz) of **32a**

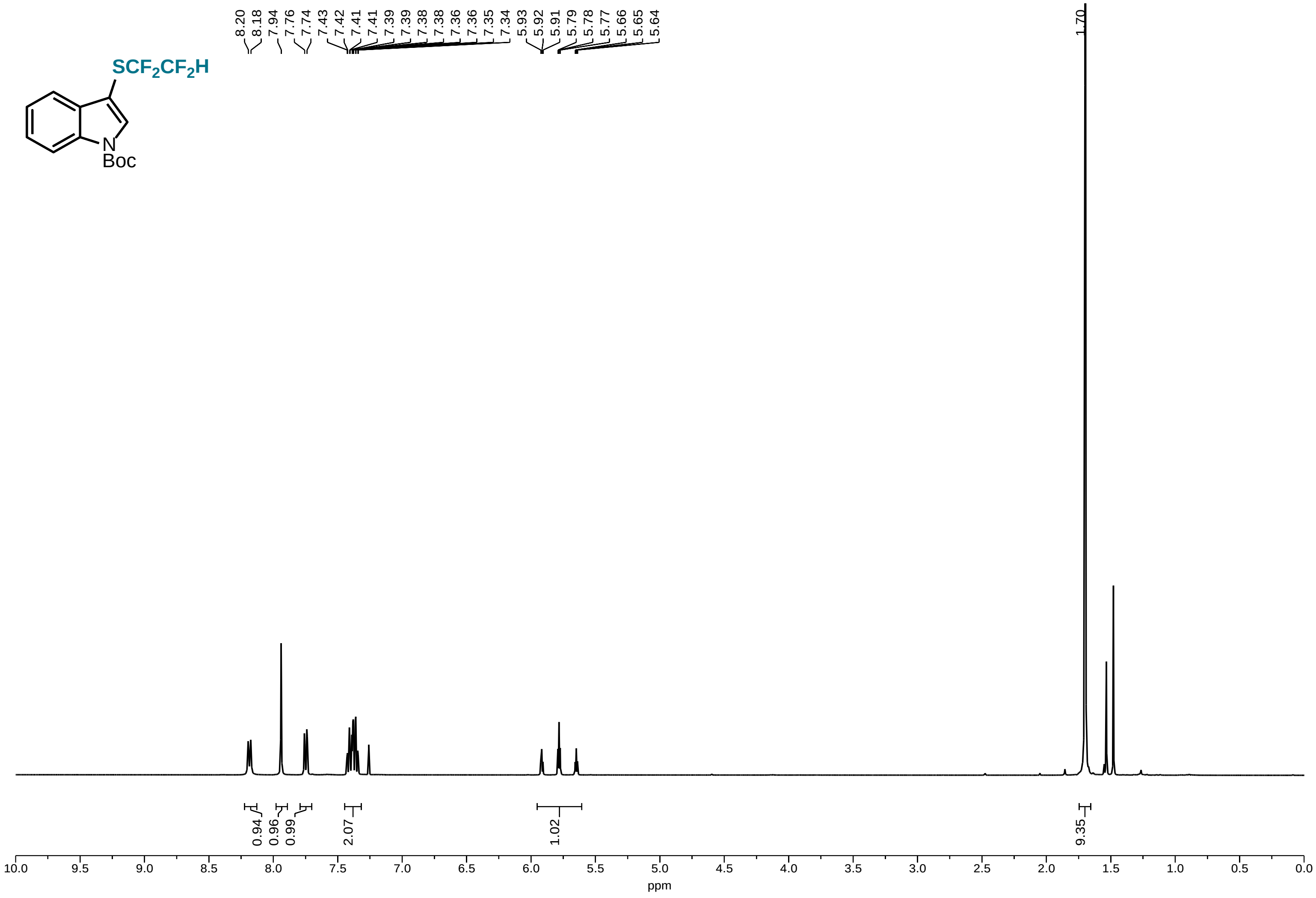

Figure S160. ^{1}H NMR ($CDCl_3$, 400 MHz) of **33a**

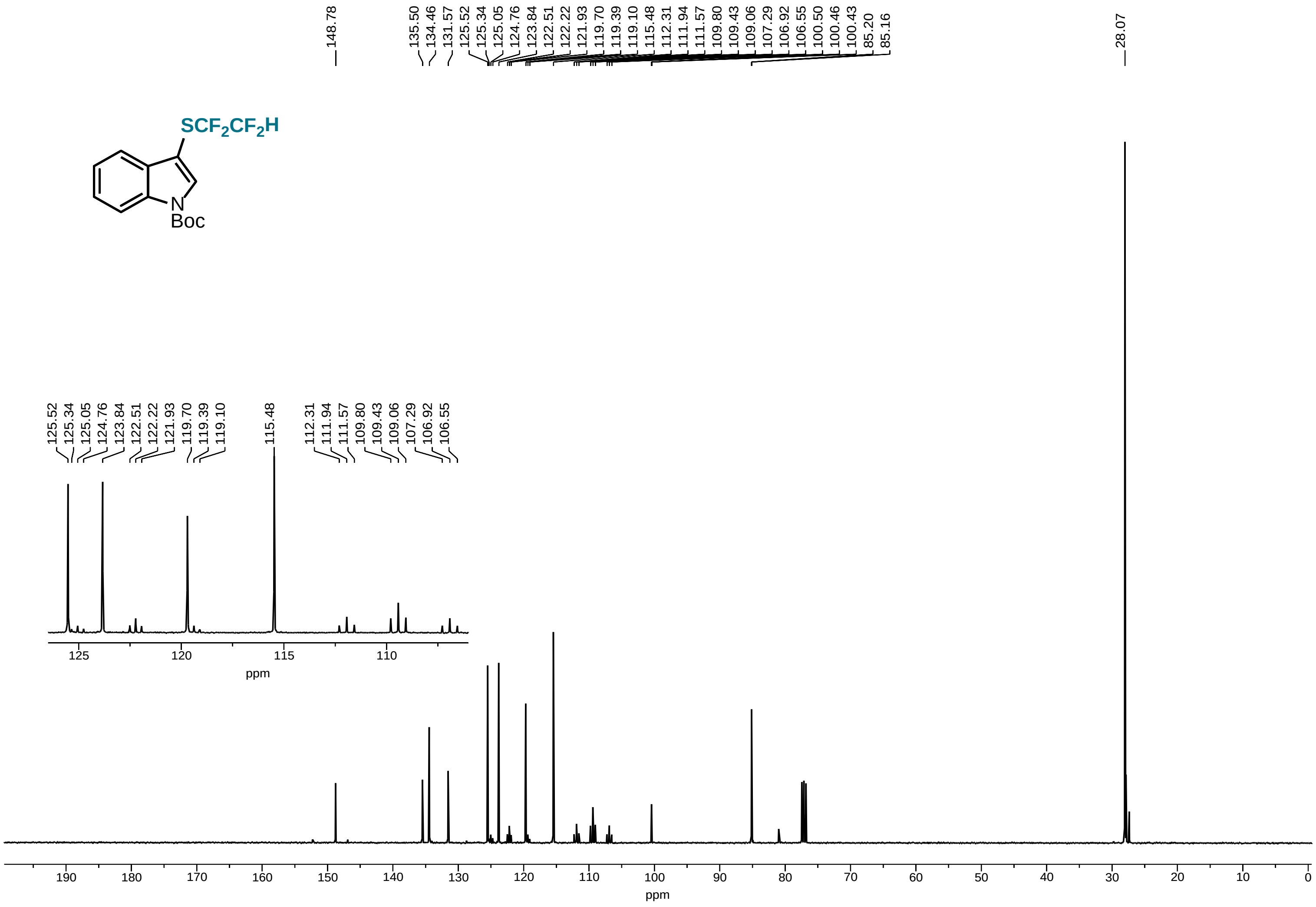

Figure S161. $^{13}C\{^{1}H\}$ NMR ($CDCl_3$, 100.6 MHz) of **33a**

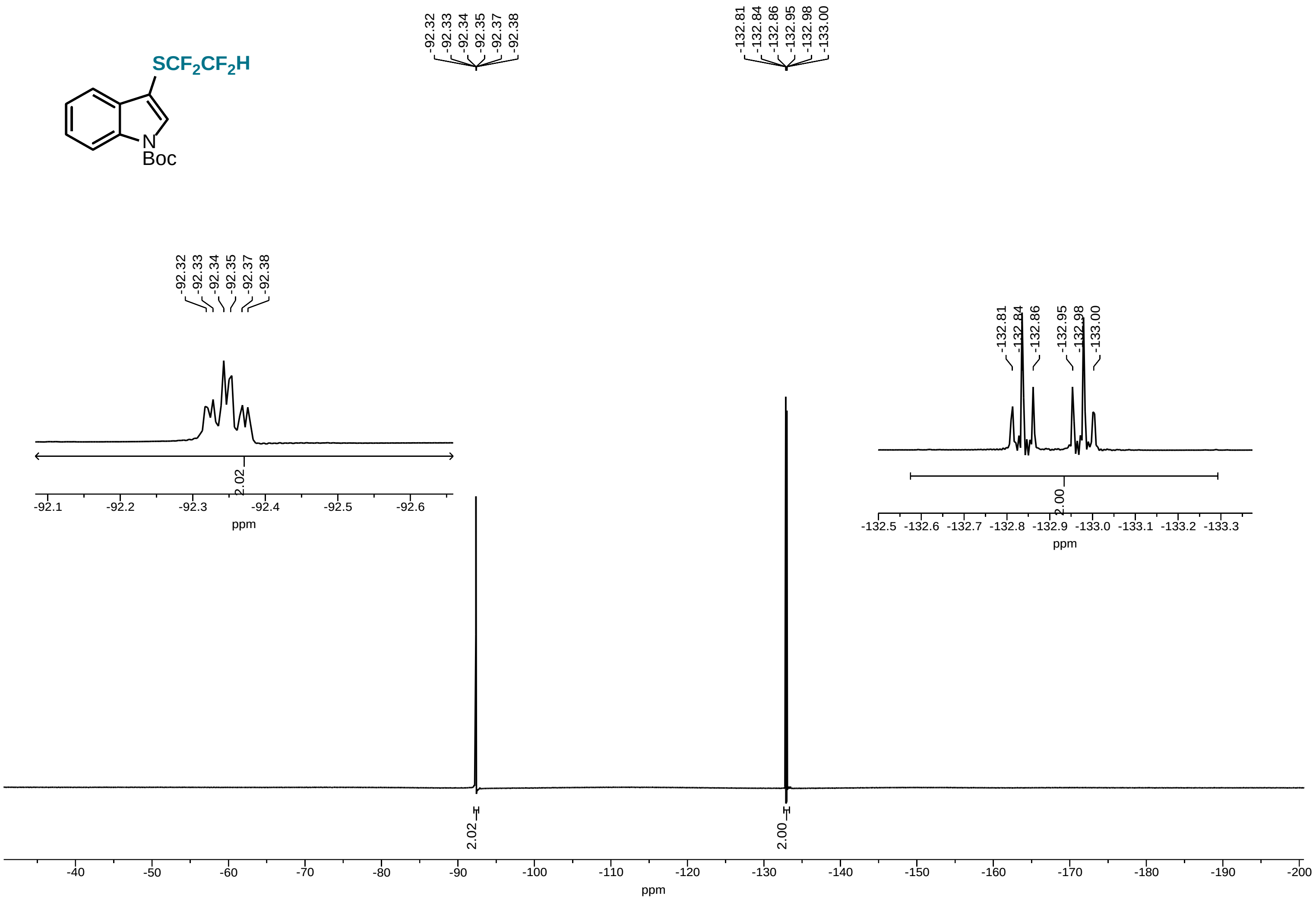

Figure S162. ^{19}F NMR ($CDCl_3$, 376.5 MHz) of **33a**

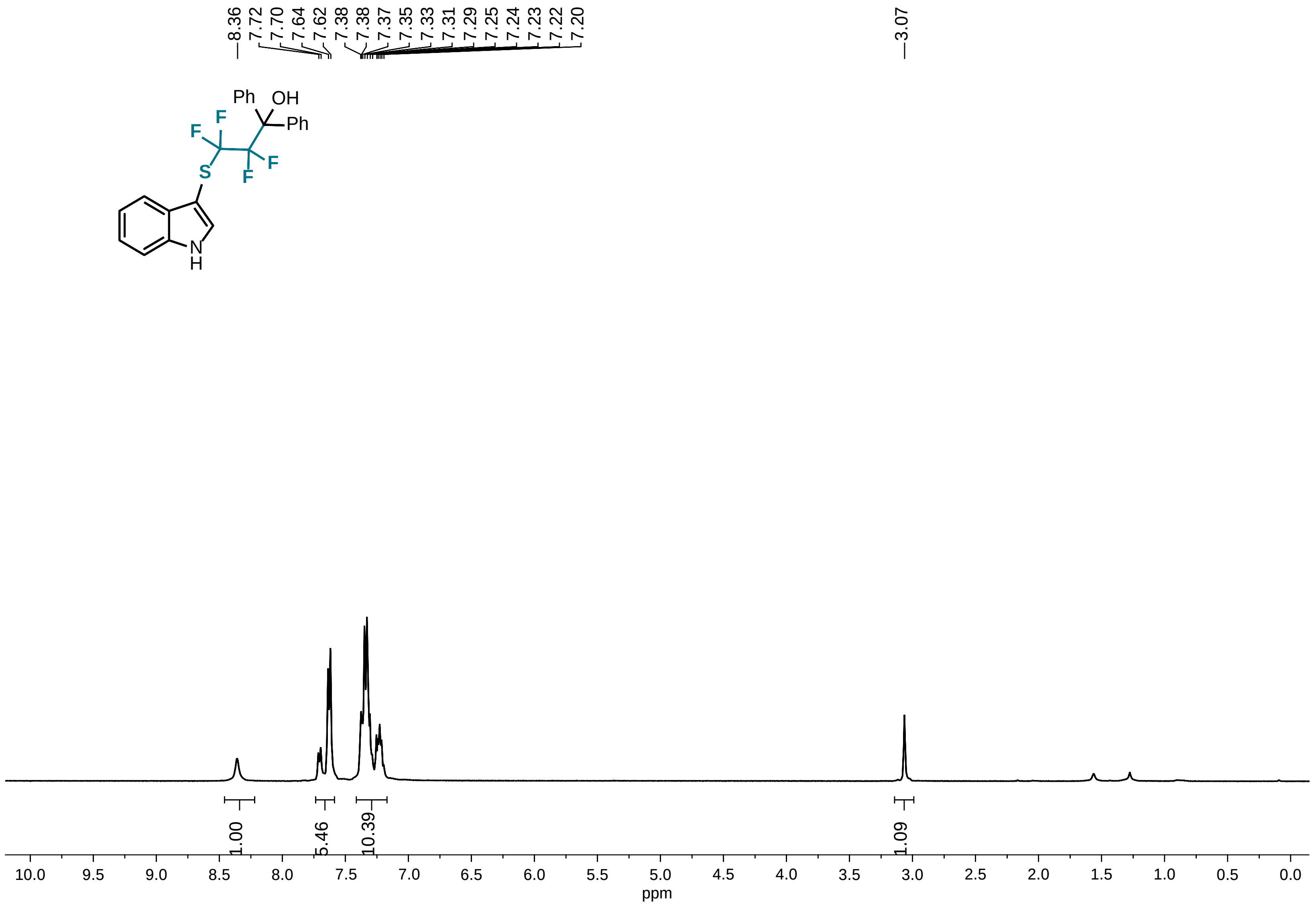

Figure S163. ^{1}H NMR ($CDCl_3$, 400 MHz) of **34a**

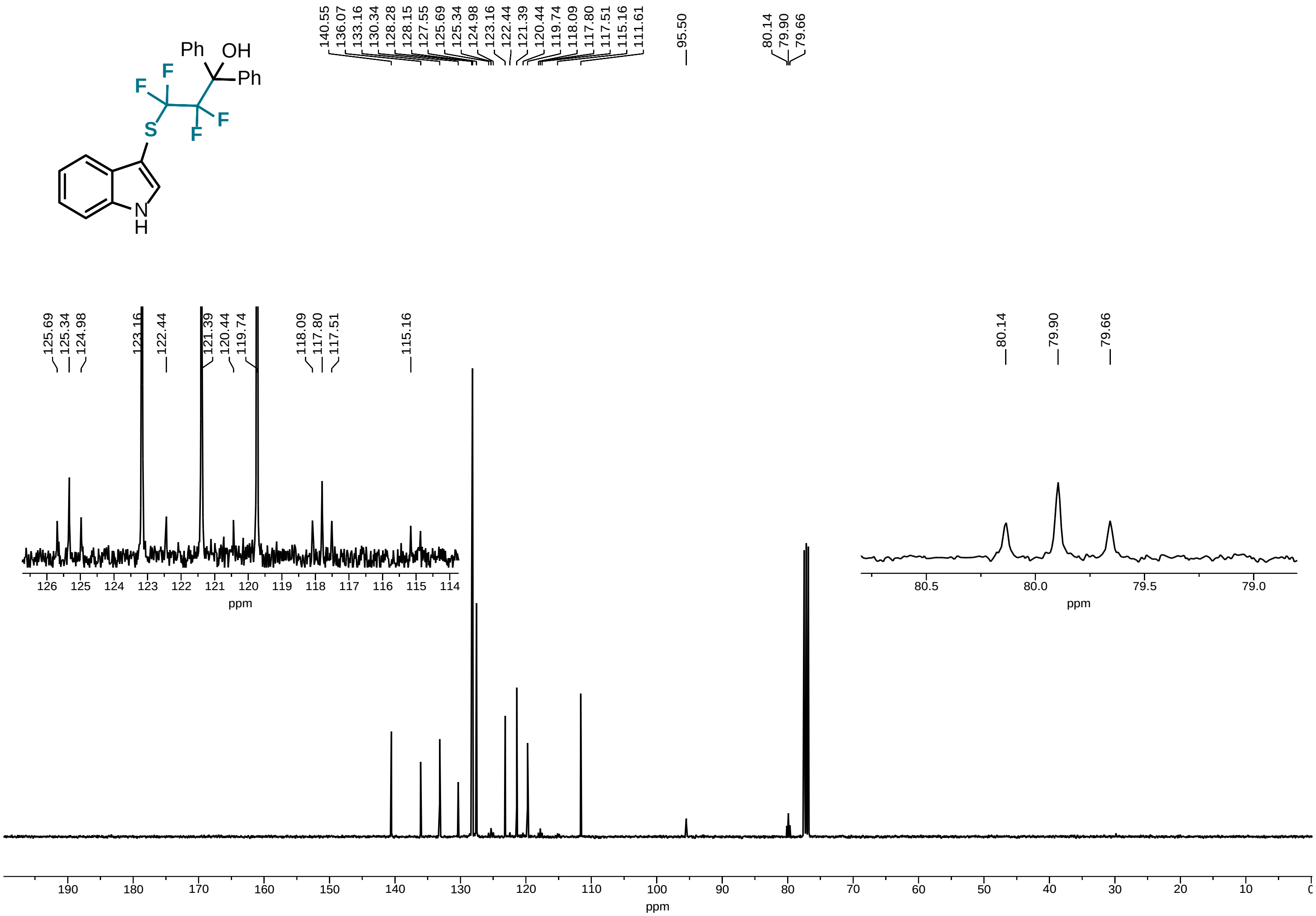

Figure S164. $^{13}C\{^{1}H\}$ NMR ($CDCl_3$, 100.6 MHz) of **34a**

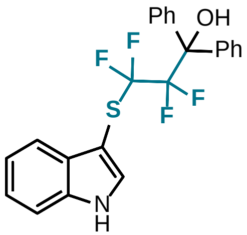

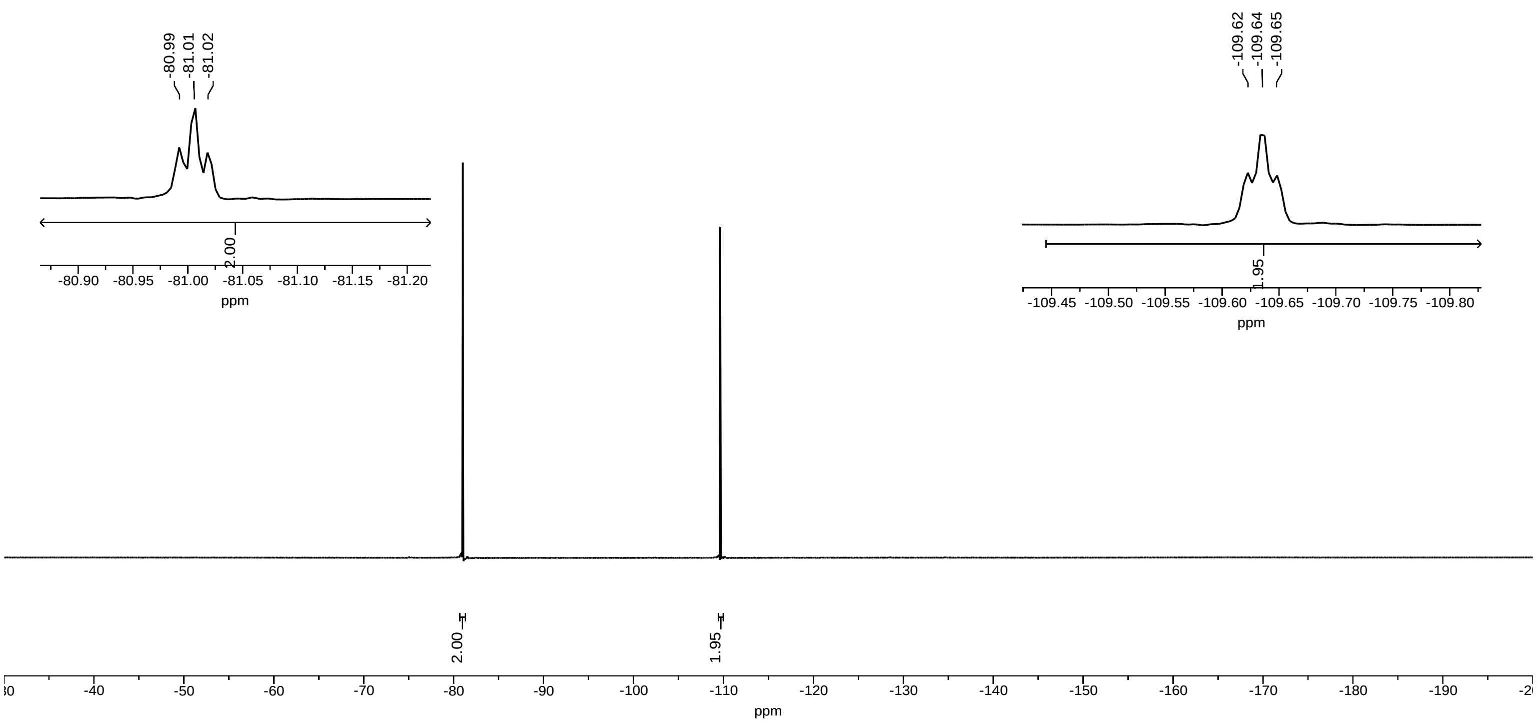

Figure S165. ^{19}F NMR ($CDCl_3$, 376.5 MHz) of **34a**

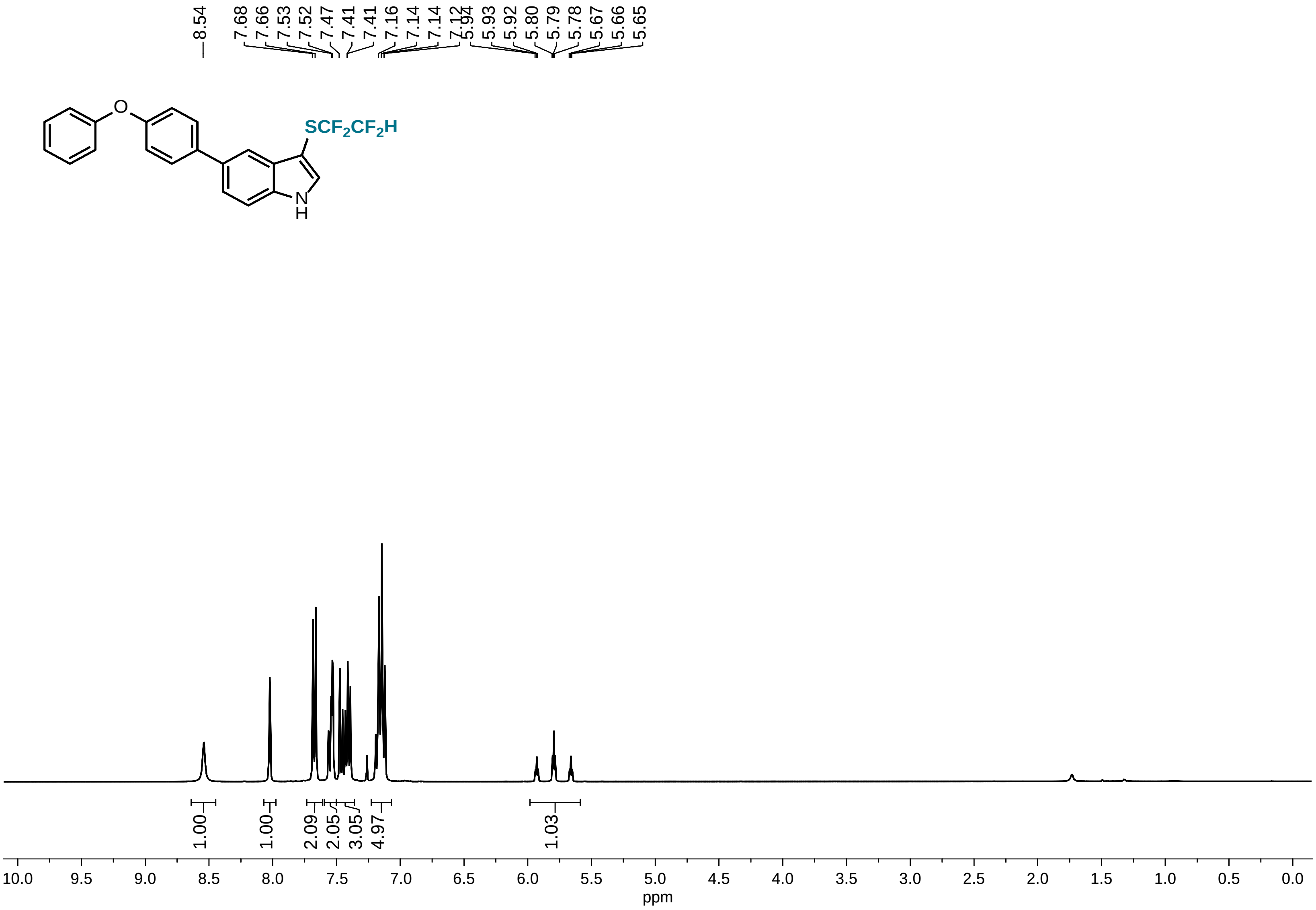

Figure S166. ^{1}H NMR ($CDCl_3$, 400 MHz) of **35a**

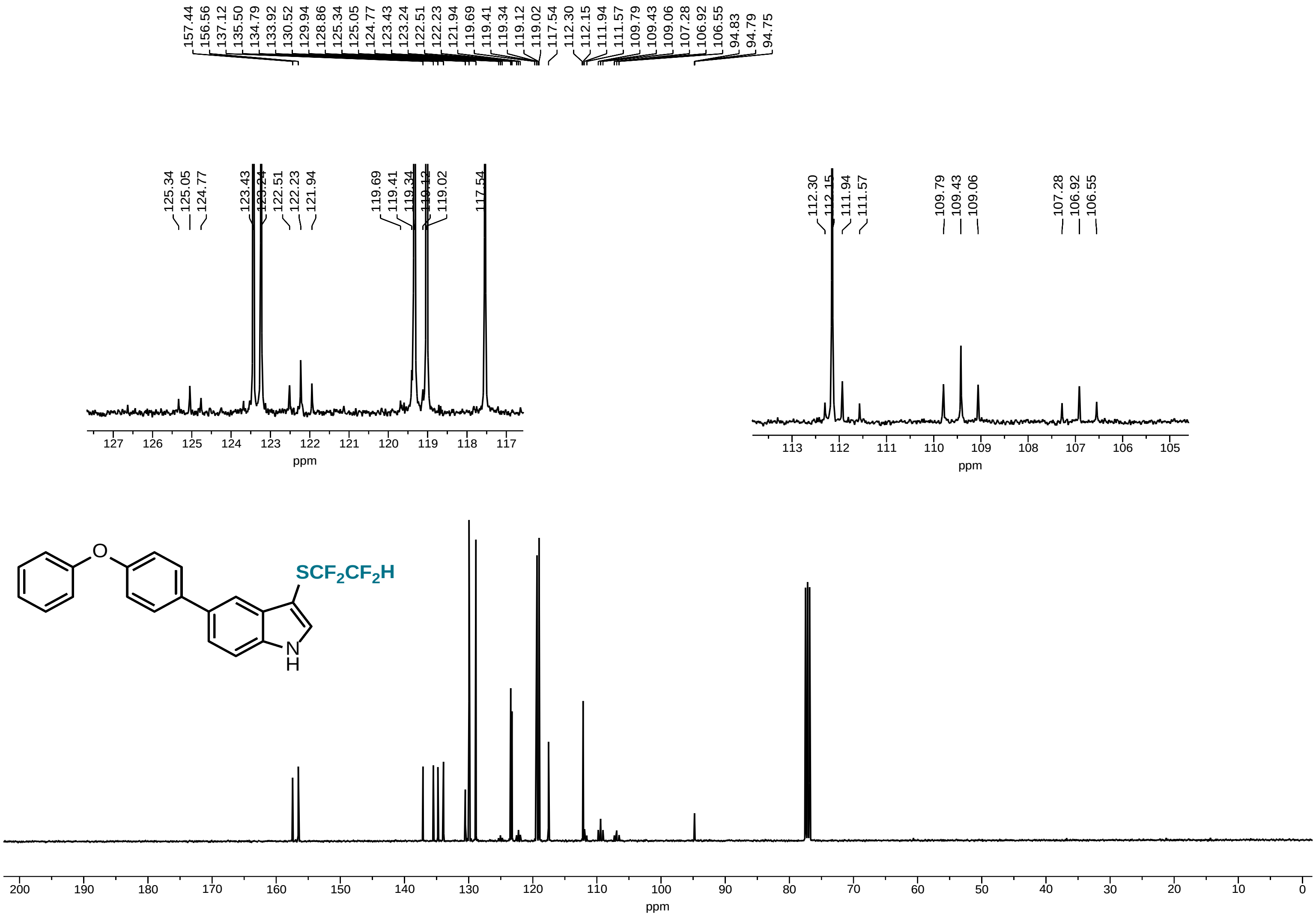

Figure S167. $^{13}C\{^1H\}$ NMR ($CDCl_3$, 100.6 MHz) of **35a**

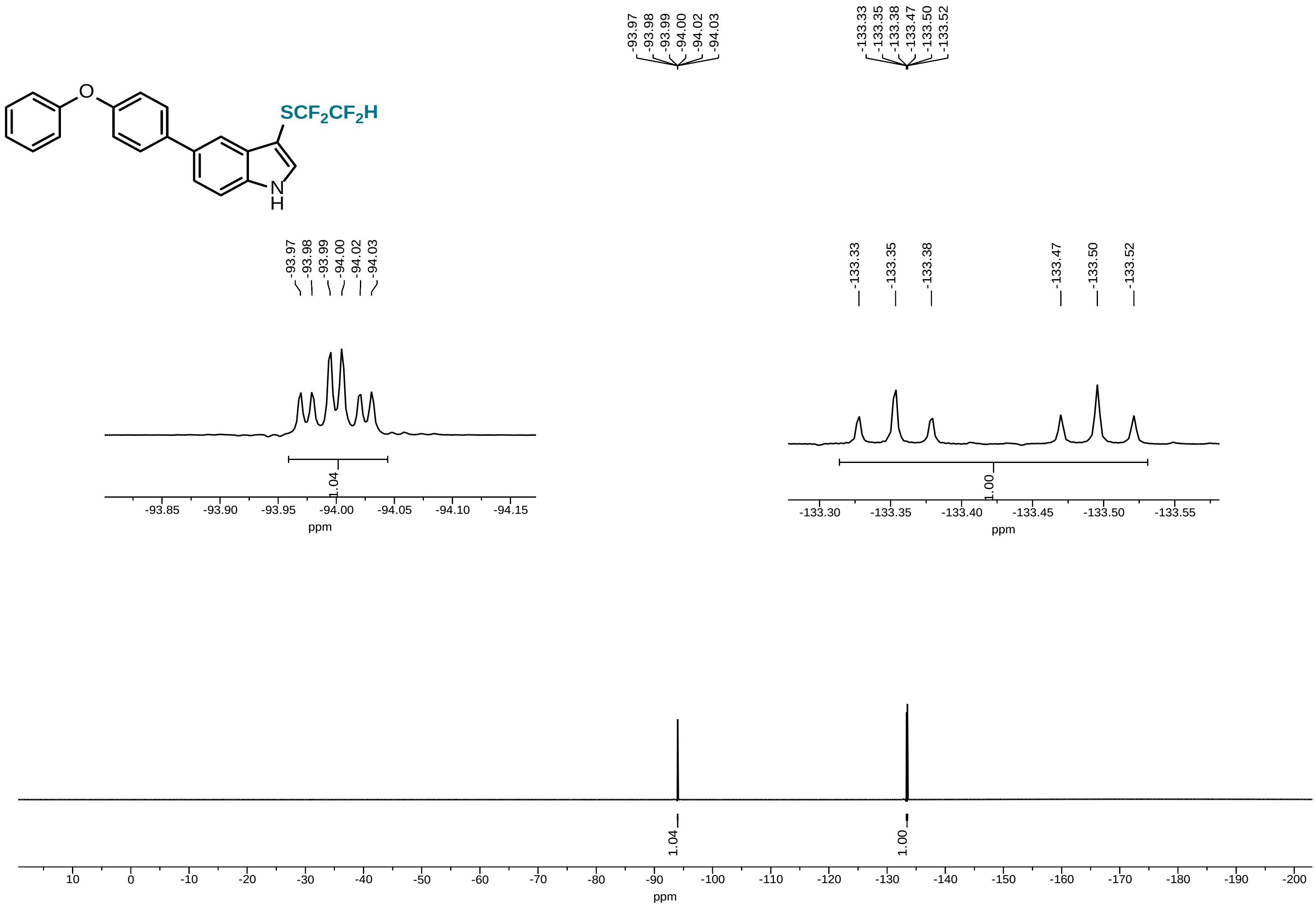

Figure S168. ^{19}F NMR ($CDCl_3$, 376.5 MHz) of **35a**

7. X-ray crystallographic data

Data collection: crystal structure determination was carried out using a Apex DUO Kappa 4-axis goniometer equipped with an APPEX 2 4K CCD area detector, a Microfocus Source E025 IuS using MoKα radiation, Quazar MX multilayer Optics as monochromator and an Oxford Cryosystems low temperature device Cryostream 700 plus (T = –173 °C). Full-sphere data collection was used with ω and φ scans. *Programs used:* Data collection APEX-2,[5] data reduction Bruker Saint[6] V/.60A and absorption correction SADABS.[7]

Structure Solution and Refinement: crystal structure solution was achieved using the computer program SHELXT.[8] Visualization was performed with the program SHELXle.[9] Missing atoms were subsequently located from difference Fourier synthesis and added to the atom list. Least-squares refinement on F^2 using all measured intensities was carried out using the program SHELXL 2015.[10] All non-hydrogen atoms were refined including anisotropic displacement parameters.

Table S2. Compounds reported and characterized by X-ray diffraction

structure	reference
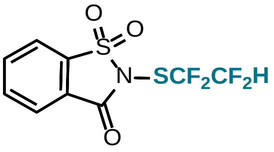	**CCDC**: 2099949<br>Compound **8a**
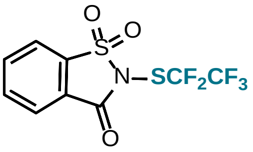	**CCDC**: 2099951<br>Compound **8b**
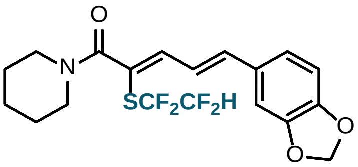	**CCDC**: 2099950<br>Compound **23a*E***
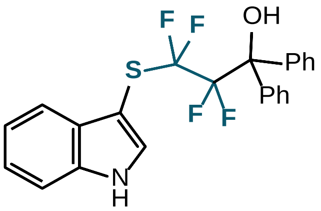	**CCDC**: 2099948<br>Compound **34a**

2-((1,1,2,2-Tetrafluoroethyl)thio)benzo[*d*]isothiazol-3(2*H*)-one 1,1-dioxide (8a).

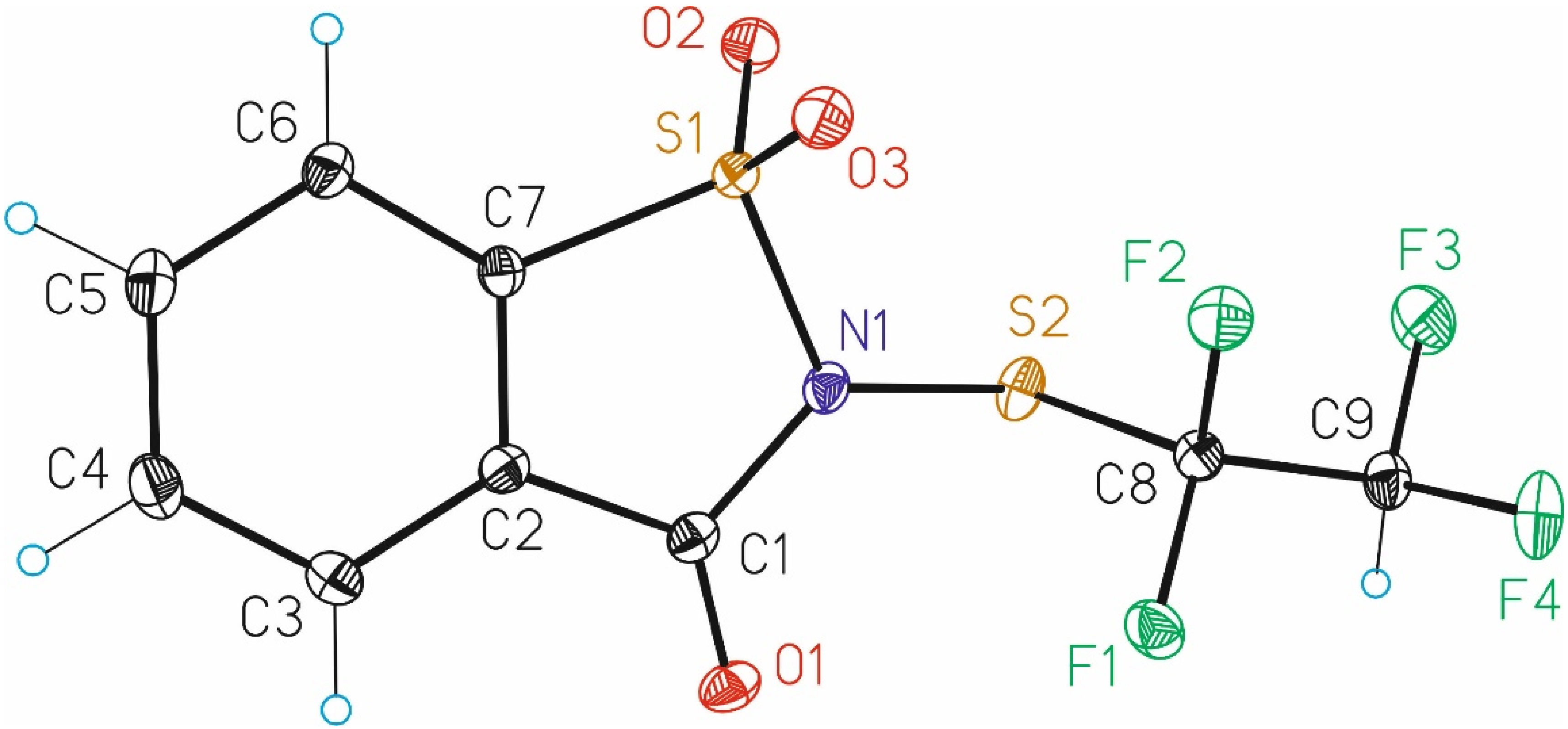

Figure S169. ORTEP diagram of compound **8a** with 50% probability ellipsoid:
Black = carbon, Red = oxygen, Blue = hydrogen, Green = fluorine, Yellow = sulfur.

Table S3. Crystal data and structure refinement for **8a**

CCDC	2099949	
Empirical formula	C9 H5 F4 N O3 S2	
Formula weight	315.26	
Temperature	100(2) K	
Wavelength	0.71073 Å	
Crystal system	monoclinic	
Space group	P 21/n	
Unit cell dimensions	a = 11.0781(5) Å	a = 90°.
	b = 8.5711(4) Å	b = 103.7010(10)°.
	c = 12.5693(6) Å	g = 90°.
Volume	1159.51(9) $Å^3$	
Z	4	
Density (calculated)	1.806 Mg/m^3	
Absorption coefficient	0.515 mm^{-1}	
F(000)	632	
Crystal size	0.200 x 0.100 x 0.100 mm^3	
Theta range for data collection	2.904 to 32.061°.	
Index ranges	-16<=h<=14,-12<=k<=6,-11<=l<=18	
Reflections collected	7908	
Independent reflections	3710[R(int) = 0.0113]	
Completeness to theta =32.061°	91.6%	
Absorption correction	Multi-scan	
Max. and min. transmission	0.74 and 0.71	
Refinement method	Full-matrix least-squares on F^2	
Data / restraints / parameters	3710/ 0/ 172	
Goodness-of-fit on F^2	1.024	
Final R indices [I>2sigma(I)]	R1 = 0.0257, wR2 = 0.0724	
R indices (all data)	R1 = 0.0275, wR2 = 0.0738	
Largest diff. peak and hole	0.665 and -0.315 e.Å	

2-((Perfluoroethyl)thio)benzo[*d*]isothiazol-3(2*H*)-one 1,1-dioxide (8b).

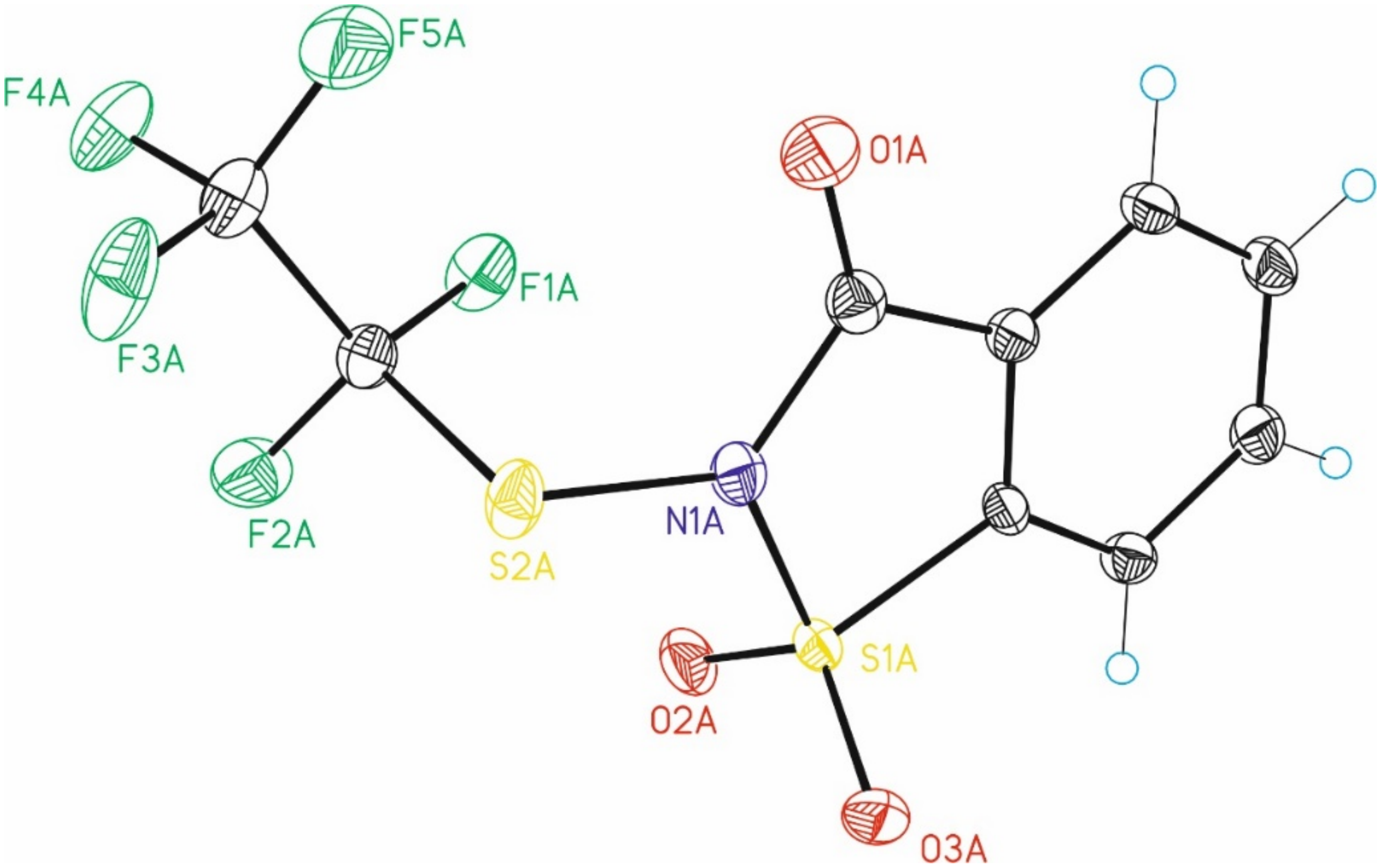

Figure S170. ORTEP diagram of compound **8b** with 50% probability ellipsoid:
Black = carbon, Red = oxygen, Blue = hydrogen, Green = fluorine, Yellow = sulfur.

Table S4. Crystal data and structure refinement for **8b**

CCDC	2099951	
Empirical formula	C18 H8 F10 N2 O6 S4	
Formula weight	666.50	
Temperature	100(2) K	
Wavelength	0.71073 Å	
Crystal system	monoclinic	
Space group	P 21/c	
Unit cell dimensions	a = 16.7878(9) Å	a = 90°.
	b = 15.5639(8) Å	b =
90.1350(10)°.		
	c = 9.1160(5) Å	g = 90°.
Volume	2381.9(2) Å^3	
Z	4	
Density (calculated)	1.859 Mg/m^3	
Absorption coefficient	0.519 mm^{-1}	
F(000)	1328	
Crystal size	0.300 x 0.200 x 0.100 mm^3	
Theta range for data collection	1.784 to 31.659°.	
Index ranges	-21<=h<=24,-15<=k<=22,-13<=l<=13	
Reflections collected	32272	
Independent reflections	7997[R(int) = 0.0352]	
Completeness to theta =31.659°	99.5%	
Absorption correction	Multi-scan	
Max. and min. transmission	0.74 and 0.68	
Refinement method	Full-matrix least-squares on F^2	
Data / restraints / parameters	7997/ 0/ 361	
Goodness-of-fit on F^2	1.007	
Final R indices [I>2sigma(I)]	R1 = 0.0348, wR2 = 0.0830	
R indices (all data)	R1 = 0.0520, wR2 = 0.0915	
Largest diff. peak and hole	0.468 and -0.444 e.Å	

(2Z,4E)-5-(Benzo[d][1,3]dioxol-5-yl)-1-(piperidin-1-yl)-2-((1,1,2,2-tetrafluoroethyl)thio)penta-2,4-dien-1-one (23a*E*).

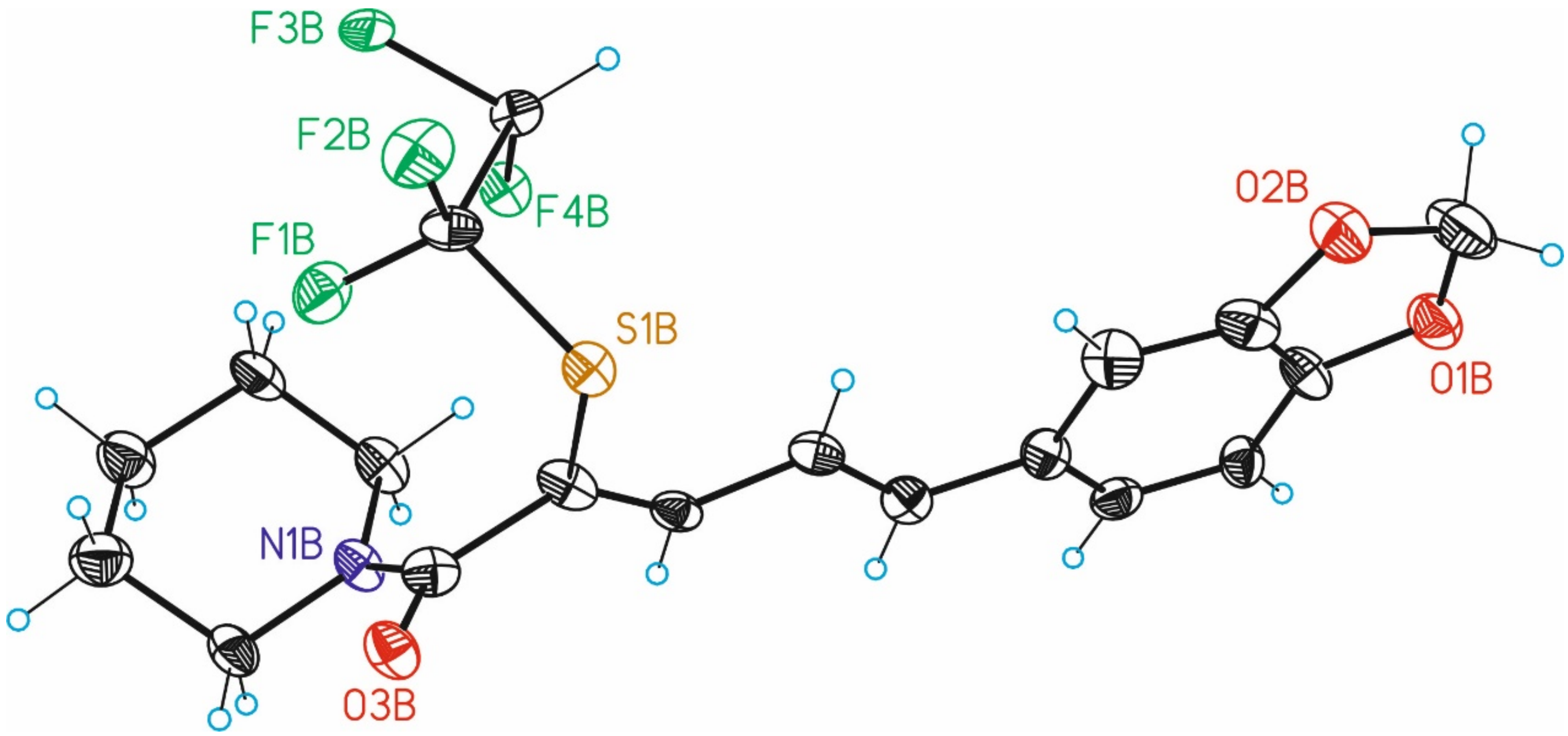

Figure S171. ORTEP diagram of compound **23a*E*** with 50% probability ellipsoid: Black = carbon, Red = oxygen, Blue = hydrogen, Green = fluorine, Yellow = sulfur

Table S5. Crystal data and structure refinement for **23a*E***

CCDC	2099950	
Empirical formula	C19 H19 F4 N O3 S	
Formula weight	417.41	
Temperature	100(2) K	
Wavelength	0.71073 Å	
Crystal system	monoclinic	
Space group	C c	
Unit cell dimensions	a = 35.201(5) Å	a = 90°.
	b = 16.053(2) Å	b = 98.142(3)°.
	c = 13.0624(18) Å	g = 90°.
Volume	7306.8(17) $Å^3$	
Z	16	
Density (calculated)	1.518 Mg/m^3	
Absorption coefficient	0.238 mm^{-1}	
F(000)	3456	
Crystal size	0.500 x 0.400 x 0.030 mm^3	
Theta range for data collection	1.169 to 28.784°.	
Index ranges	-47<=h<=47,-21<=k<=21,-17<=l<=17	
Reflections collected	104557	
Independent reflections	18500[R(int) = 0.0765]	
Completeness to theta =28.784°	97.8%	
Absorption correction	Multi-scan	
Max. and min. transmission	0.74 and 0.43	
Refinement method	Full-matrix least-squares on F^2	
Data / restraints / parameters	18500/ 2694/ 1550	
Goodness-of-fit on F^2	1.170	
Final R indices [I>2sigma(I)]	R1 = 0.0943, wR2 = 0.2554	
R indices (all data)	R1 = 0.0985, wR2 = 0.2602	
Largest diff. peak and hole	0.894 and -3.081 e.$Å^{-3}$	

3-((1*H*-indole-3-yl)thio)-2,2,3,3-tetrafluoro-1,1-diphenylpropan-1-ol (34a).

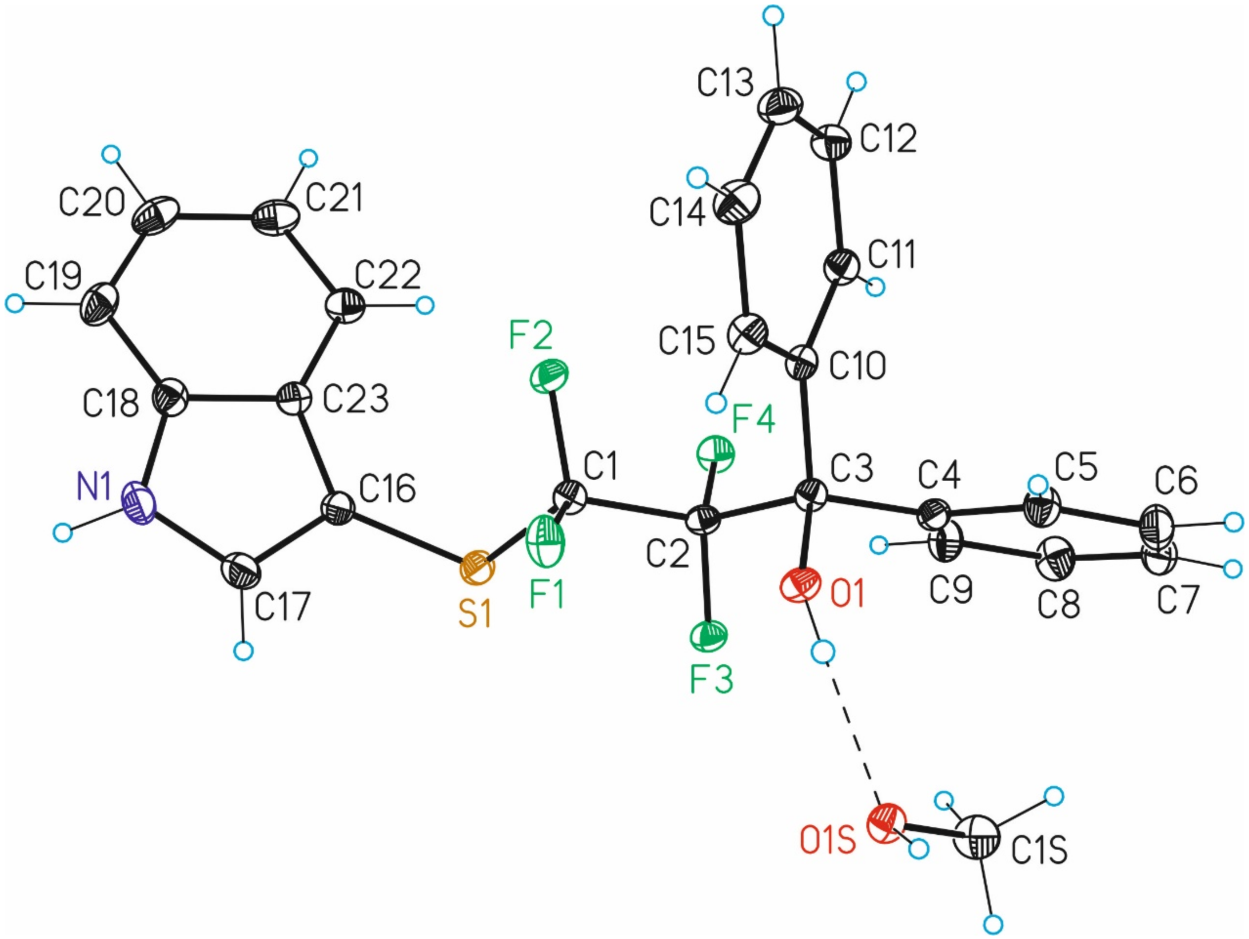

Figure S172. ORTEP diagram of compound **34a** with 50% probability ellipsoid:
Black = carbon, Red = oxygen, Blue = hydrogen, Green = fluorine, Yellow = sulfur

Table S6. Crystal data and structure refinement for **34a**

CCDC	2099948	
Empirical formula	C24 H21 F4 N O2 S	
Formula weight	463.48	
Temperature	100(2)K	
Wavelength	0.71073 Å	
Crystal system	triclinic	
Space group	P -1	
Unit cell dimensions	a = 9.87250(19)Å	a = 111.365(2)°.
	b = 9.96039(19)Å	b = 100.5179(18)°.
	c = 12.9592(3)Å	g = 106.4761(17)°.
Volume	1078.14(4) $Å^3$	
Z	2	
Density (calculated)	1.428 Mg/m^3	
Absorption coefficient	0.206 mm^{-1}	
F(000)	480	
Crystal size	0.400 x 0.300 x 0.080 mm^3	
Theta range for data collection	2.376 to 31.782°.	
Index ranges	-14<=h<=10,-14<=k<=14,-18<=l<=17	
Reflections collected	18656	
Independent reflections	6707[R(int) = 0.0207]	
Completeness to theta =31.782°	91.1%	
Absorption correction	Multi-scan	
Max. and min. transmission	1.00 and 0.83	
Refinement method	Full-matrix least-squares on F^2	
Data / restraints / parameters	6707/ 0/ 299	
Goodness-of-fit on F^2	1.045	
Final R indices [I>2sigma(I)]	R1 = 0.0340, wR2 = 0.0920	
R indices (all data)	R1 = 0.0414, wR2 = 0.0958	
Largest diff. peak and hole	0.576 and -0.210 e.$Å^{-3}$	

8. References

1. Chalmers, A. A.; Hall, R. H. Conformational studies of D-glycals by ^{1}H nuclear magnetic resonance spectroscopy. *J. Chem. Soc.; Perkin Trans. 2*, **1974**, 728–732.
2. Mestre, J.; Lishchynskyi, A.; Castillón, S.; Boutureira, O. Trifluoromethylation of electron-rich alkenyl iodides with fluoroform-derived “ligandless” $CuCF_3$. *J. Org. Chem.* **2018**, *83*, 8150−8160.
3. Cobo, I.; Matheu, M. I.; Castillón, S.; Boutureira, O.; Davis, B. G. Phosphine-free Suzuki–Miyaura cross-coupling in aqueous media enables access to 2-*C*-aryl-glycosides. *Org. Lett.* **2012**, *14*, 1728–1731.
4. Mestre, J.; Castillón, S.; Boutureira, O. “Ligandless” pentafluoroethylation of unactivated (hetero)aryl and alkenyl halides enabled by the controlled self-condensation of $TMSCF_3$-derived $CuCF_3$. *J. Org. Chem.* **2019**, *84*, 15087–15097.
5. Data collection with APEX II version v2013.4-1. Bruker (**2007**). Bruker AXS Inc., Madison, Wisconsin, USA.
6. Data reduction with Bruker SAINT version V8.30c. Bruker (**2007**). Bruker AXS Inc., Madison, Wisconsin, USA.
7. SADABS: V2012/1 Bruker (**2001**). Bruker AXS Inc., Madison, Wisconsin, USA. Blessing, R. H. *Acta Cryst.* **1995**, A51, 33–38.
8. SHELXT; V2014/4 (Sheldrick 2014). Sheldrick, G. M. *Acta Cryst*. **2015**, A71, 3–8.
9. SHELXle; Huebschle, C. B.; Sheldrick, G. M.; Dittrich B. *J. Appl. Cryst.* **2011**, *44*, 1281–1284.
10. SHELXL; SHELXL-2014/7 (Sheldrick 2014). Sheldrick, G. M. *Acta Cryst*. **2015**, C71, 3–8.
